# Mechanism of Thimerosal-Induced Structural Destabilization of a Recombinant Rotavirus P[4] Protein Antigen Formulated as a Multi-Dose Vaccine

**DOI:** 10.1016/j.xphs.2020.11.033

**Published:** 2021-03

**Authors:** Kawaljit Kaur, Jian Xiong, Nishant Sawant, Sanjeev Agarwal, John M. Hickey, David A. Holland, Tarit K. Mukhopadhyay, Joseph R. Brady, Neil C. Dalvie, Mary Kate Tracey, Kerry R. Love, J. Christopher Love, David D. Weis, Sangeeta B. Joshi, David B. Volkin

**Affiliations:** a Department of Pharmaceutical Chemistry, Vaccine Analytics and Formulation Center, University of Kansas, 2030 Becker Drive, Lawrence, Kansas 66047; b Department of Biochemical Engineering, University College London, Bernard Katz Building, Gower Street, London WC1E 6BT, UK; c Department of Chemical Engineering, Koch Institute for Integrative Cancer Research, Massachusetts Institute of Technology, Cambridge, MA 02139; d Department of Chemistry and R.N. Adams Institute of Bioanalytical Chemistry, University of Kansas, Lawrence, Kansas 66045

**Keywords:** Multi-dose, Formulation, Stability, Protein antigen, Vaccine, Preservative, Thimerosal, Rotavirus, HX-MS, Epitope mapping

## Abstract

In a companion paper, a two-step developability assessment is presented to rapidly evaluate low-cost formulations (multi-dose, aluminum-adjuvanted) for new subunit vaccine candidates. As a case study, a non-replicating rotavirus (NRRV) recombinant protein antigen P[4] was found to be destabilized by the vaccine preservative thimerosal, and this effect was mitigated by modification of the free cysteine (C173S). In this work, the mechanism(s) of thimerosal-P[4] protein interactions, along with subsequent effects on the P[4] protein’s structural integrity, are determined. Reversible complexation of ethylmercury, a thimerosal degradation byproduct, with the single cysteine residue of P[4] protein is demonstrated by intact protein mass analysis and biophysical studies. A working mechanism involving a reversible S-Hg coordinate bond is presented based on the literature. This reaction increased the local backbone flexibility of P[4] within the helical region surrounding the cysteine residue and then caused more global destabilization, both as detected by HX-MS. These effects correlate with changes in antibody-P[4] binding parameters and alterations in P[4] conformational stability due to C173S modification. Epitope mapping by HX-MS demonstrated involvement of the same cysteine-containing helical region of P[4] in antibody-antigen binding. Future formulation challenges to develop low-cost, multi-dose formulations for new recombinant protein vaccine candidates are discussed.

## Introduction

To reduce vaccine cost and expand immunization coverage, multi-dose formulations are an important component of global immunization programs.^1^ Multi-dose vaccine formulations offer several cost advantages over single-dose formats including (1) reduced packaging costs since multiple doses are supplied in a single vial, (2) decreased distribution costs since less cold-chain space is required for storage and transportation, and (3) lower disposal costs for medical waste.^1^ These benefits lead to their preferred use in vaccines targeted for use in low and middle-income countries (LMICs), particularly those procured and distributed by UNICEF.^2^ Multi-dose vaccine vials thus play a key role in the success of LMIC’s immunization programs, especially for widely-distributed vaccines used in locations with more limited cold-chain storage space.^3^

Multi-dose formulations of inactivated or recombinant-protein based vaccines contain antimicrobial preservatives (APs) to prevent the growth of microorganisms that may be introduced accidently during multiple drawings from a single container. The most commonly used vaccine preservative is thimerosal, followed by phenoxyethanol and phenol.^4^^,^^5^ Since the 1930s, thimerosal has been used as a preservative in many biological products including vaccines.^6^ Thimerosal is an anionic, ethylmercury-containing organic compound efficient at preventing microbial contamination with a well-documented record of safe use, despite disproven, controversial associations of thimerosal with neurodevelopmental disorders in young children.^7^, ^8^, ^9^ Thimerosal is currently used as a preservative in many pediatric combination vaccines (e.g., Pentabio, Eupenta, and ComBE Five) prequalified by the World Health Organization for use in LMICs.^10^

Despite the need for APs to protect multi-dose formulations against microbial contamination, several studies have reported detrimental effects and incompatibility of APs with certain vaccines. For example, Sawyer *et al.* observed loss of potency of inactivated poliovirus vaccine (IPV) upon its combination with diphtheria-tetanus-pertussis vaccine containing thimerosal,^11^ and others have observed deleterious effects of APs with human papilloma virus (HPV) vaccines as well as with a live attenuated rotavirus vaccine candidate.^12^^,^^13^ Mechanistic studies of AP-induced destabilization of protein-based drugs including monoclonal antibodies have demonstrated direct interactions leading to structural alterations, decreased conformational stability and increased aggregation.^14^, ^15^, ^16^, ^17^ Several studies have probed the nature of the interaction between thimerosal and various model proteins.^18^, ^19^, ^20^, ^21^ Thimerosal rapidly degrades to ethylmercury and thiosalicylate in aqueous media, with the former able to react with the free and surface-exposed cysteine residues of proteins to form protein-ethylmercury adducts.^18^^,^^19^

Recent work in our laboratories demonstrated the incompatibility of two APs (thimerosal and 2-phenoxyethanol) with three different recombinant non-replicating rotavirus (NRRV) fusion protein antigens as part of the analytical and formulation development of a trivalent NRRV vaccine candidate.^22^ As described in the companion paper^23^ and elsewhere,^22^^,^^24^, ^25^, ^26^, ^27^ each recombinant NRRV antigen comprises a truncated rotavirus VP4 surface protein (derived from three target RV strains) linked to the tetanus toxoid universal CD4^+^ T-cell epitope, and are simply referred to as the P[4], P[6], and P[8] protein antigens. The NRRV vaccine candidate has been shown to be safe and immunogenic in infants and toddlers in early clinical trials conducted by PATH in South Africa,^28^^,^^29^ and mid to late stage clinical evaluations are ongoing.^30^ Thus, this NRRV vaccine candidate against rotavirus is a potentially attractive low-cost alternative to the currently-used live-attenuated rotavirus vaccines, especially if it can be combined as part of pediatric multi-dose combination vaccines currently used in LMICs.

As described in a companion paper,^23^ a two-step vaccine formulation developability assessment was used to evaluate a series of NRRV P[4] and P[8] variants produced in *Komagataella phaffii* (*Pichia pastoris*, *Pp*) with an emphasis on their stability in the presence and absence of the AP thimerosal (TH) and the aluminum-adjuvant Alhydrogel (AH). AH-adsorbed NRRV P[4] and P[8] antigens were shown be to incompatible with TH during storage, and the TH-induced destabilization could be mitigated by site-directed mutagenesis of the single cysteine residue. Here, we explore in detail the molecular mechanism(s) by which TH interacts with variants of the NRRV P[4] antigen, using material produced in *E. coli* and *K. phaffii*, including the effects of TH on the protein antigen’s structural integrity and conformational stability (using biophysical methods), antibody-binding properties (with a P[4]-antigen specific mAb using Octet analysis), and local backbone flexibility (using hydrogen exchange-mass spectrometry, HX-MS). We also utilized HX-MS epitope mapping to describe the interactions of an P[4]-antigen specific mAb with the P[4] antigen to better elucidate the mechanisms of the observed thimerosal-induced destabilization effects. These findings are discussed in the context of ongoing and future challenges associated with AP-induced destabilization of recombinant protein antigens when being formulated as new vaccine candidates for use in LMICs.

## Materials and Methods

### Materials

A total of three different NRRV P[4] fusion-protein antigens were used in these studies including *E*. *coli* expressed parent protein P[4], *K. phaffii* (*P. pastoris*) expressed parent protein P[4], and *K. phaffii* (*P. pastoris*) expressed P[4] variant (C173S), which are hereafter referred as *E. coli* P[4], *Pp* P[4] and *Pp* P[4]-C173S, respectively. The detailed description of the design of these P[4] mutants as well as the procedures for their expression and purification are described elsewhere^31^^,^^32^ and in the companion paper.^23^ The purity of these three P[4] samples was estimated to be >90% as determined by SDS-PAGE analysis and densitometry (Supplementary Fig. S1A). Sodium phosphate dibasic heptahydrate and sodium chloride (NaCl) were purchased from Fisher Chemicals (Hampton, NH). Sodium phosphate monobasic monohydrate, 8-anilino-1-naphthalenesulfonic acid (ANS), thimerosal (TH), and dimethyl sulfoxide (DMSO) were purchased from Sigma-Aldrich (St. Louis, MO). Slide-A-Lyzer mini dialysis devices, HPLC vials, LC-MS grade mobile phases and isopropanol were purchased from Thermo Fisher Scientific (Waltham, MA). NRRV P[4] specific mAb was developed by PATH and obtained from Precision Antibody (Columbia, MD) as described elsewhere.^22^^,^^33^

### Sample Preparation

The frozen aliquots of purified P[4] antigens were thawed at room temperature and dialyzed against 150 mM NaCl, 10 mM sodium phosphate, pH 7.2 (phosphate-buffered saline, PBS) buffer using 3.5 kDa molecular weight cut-off (MWCO) Slide-A-Lyzer mini dialysis devices at 4°C. A total of 3 buffer exchanges were performed including one overnight exchange. NanoDrop™ 2000 spectrophotometer (Thermo Fisher Scientific, Waltham, MA) was used to determine the concentration of dialyzed samples as described earlier.^23^ Samples for biophysical characterization were prepared at 0.12 mg/mL by diluting the dialyzed protein stock solution with PBS pH 7.2. For samples containing thimerosal (TH), dialyzed protein stock solution was mixed with 40X TH stock solution (0.4% w/v TH in water) to a final protein concentration of 0.12 mg/mL and TH concentration of 0.01% w/v in PBS pH 7.2. Accumet® XL 25 dual channel pH meter (Fisher Scientific, Hampton, NH) was used to verify the pH of all samples (7.2 ± 0.2).

## Methods

Experimental details of various physicochemical and immunochemical methods used in this study with the P[4] protein antigens, including UV-visible spectroscopy, SDS-PAGE, intact protein MS spectrometry, differential scanning calorimetry (DSC), extrinsic fluorescence spectroscopy, and inhibition ELISA are described in detail elsewhere^22^^,^^26^^,^^27^ and in the companion paper.^23^ The experimental setup for Octet bio-layer interferometry and LC-MS peptide mapping methods are provided in the Supplementary Methods.

### Hydrogen Exchange-Mass Spectrometry (HX-MS)

HX-MS experiments were performed using a LEAP H/DX PAL system (Carrboro, NC) coupled with a quadrupole time-of-flight (QTOF) mass spectrometer (Agilent 6530, Santa Clara, CA) using protocols in line with the well-established HX-MS methodologies. HX-MS workflow for mapping epitopes were carried out as described previously with some adaptations.^34^ Briefly, P[4] samples in PBS were concentrated to 0.16 mg/mL using 3 kDa MWCO spin centrifugal filters. Three microliter of each sample was incubated with 27 μL of deuterated labeling buffer (150 mM NaCl, 10 mM sodium phosphate, pH_read_ 6.8) at 20°C. Triplicate samples were labeled at 6 different time points between 20 and 62,500 s (20, 100, 500, 2500, 12,500 and 62,500 s). For P[4]-TH interaction studies, TH was added at a final concentration of 0.01% w/v (0.25 mM) in each sample prior to incubating with deuterated labeling buffer. For epitope mapping studies, a P[4]-specific mAb and P[4] were mixed at a 2:1 molar ratio, such that the final concentration of P[4]-specific mAb in the sample was 1.2 mg/mL. At each specified time point, the hydrogen exchange was quenched by 1:1 dilution with quenching buffer (200 mM phosphate, 4 M guanidinium chloride, pH 2.5) to the labeled P[4] sample at 0°C. The quenched sample was injected into a liquid chromatography (LC) system for online digestion using an in-house immobilized pepsin column (2.1 × 50 mm),^35^ followed by desalting via a trap (Zorbax 300SB-C8 2.1 × 12.5 mm, 5 μm) and chromatographic separation with the LC column (Zorbax 300SB-C18 2.1 × 50 mm, 3.5 μm). The mobile phase A was composed of water with 0.1% formic acid, and mobile phase B was acetonitrile with 0.1% formic acid. The LC mobile gradient consisted of 1% to 95% mobile phase B over 18 min. Tandem MS (MS/MS) analysis was used to generate peptide maps of the various P[4] samples. To minimize back-exchange, all chromatographic components were refrigerated at 0°C.

The HX data were processed using HDExaminer software (Sierra Analytics, Modesto, CA). For TH interaction (or epitope mapping) studies, HX data for each peptide from all HX times were averaged into a single differential value representing the TH-(or P[4] specific mAb)-bound P[4] minus P[4] alone. These values were then normalized to their theoretical maximal uptake, which is the length of peptide minus two then minus all non-*N*-terminal prolines. Normalized differential HX ($\text{Δ}\bar{\text{HX}})$ was determined as: $\text{Δ}\bar{HX}=\frac{\sum_{t\in \tau }\left({\bar{m}}_{a,t}-{\bar{m}}_{b,t}\right)}{\left|\tau \right|N}$ where, τ is the set of distinct HX labeling times, ${\bar{m}}_{a,t}$ and ${\bar{m}}_{b,t}$ denote the mean peptide masses at labeling time *t* for the two states, *a* and *b*; the notation |τ| denotes the number of HX labeling times, and *N* is the mass of the exchangeable amide hydrogens in the peptide determined by accumulating the mass of non-proline residues starting at the third residue of the peptide. $\text{Δ}\bar{HX}$ represents a unitless fractional HX difference normalized by peptide length. In the absence of back-exchange the value range between +1 and –1 with positive values denoting faster HX by state *a* and negative values denoting slower HX by state *b.* For TH interaction studies, a combination of significance testing and k-means clustering was applied as described elsewhere.^34^ For epitope mapping, the method was modified as follows: Briefly, the calculated $\text{Δ}\bar{\text{HX}}$ values of *E. coli* P[4] with P[4]-specific mAb were rank-ordered from lowest to highest, instead of using *k*-means clustering. The lowest 15% of all the peptides ($\text{Δ}\bar{\text{HX}}$ values ≤ -0.095) were empirically classified as strongly protected peptides, while the next 20% of all the peptides were classified as weakly protected peptides ($\text{Δ}\bar{\text{HX}}$ values between -0.047 and -0.095). The same $\text{Δ}\bar{\text{HX}}$ value thresholds were then applied to *Pp* P[4] and *Pp* P[4]-C173S for epitope identification. For molecular graphics, I-TASSER (zhanglab.ccmb.med.umich.edu/I-TASSER)^36^ was used to generate structural models of P[4] and were displayed using PyMOL Molecular Graphics System Version 2.0.3 (PyMOL Molecular Graphics System; Schrodinger LLC, San Diego, CA) as described previously.^26^ When mapping HX data to the homology models, conflicting results of residues from overlapping peptides were resolved as follows: strongly protected peptides overwrote weakly protected, and weakly protected peptides overwrote insignificant results. In addition, the first two residues of each peptide were ignored during the analysis, since the first two residues normally undergo rapid back-exchange during HX-MS.^37^ Please refer to HX-MS Supplemental Information for details on HX values and deuterium uptake curves.

## Results

### Interaction of Thimerosal (TH) With E. coli P[4]

**Fig 1:**
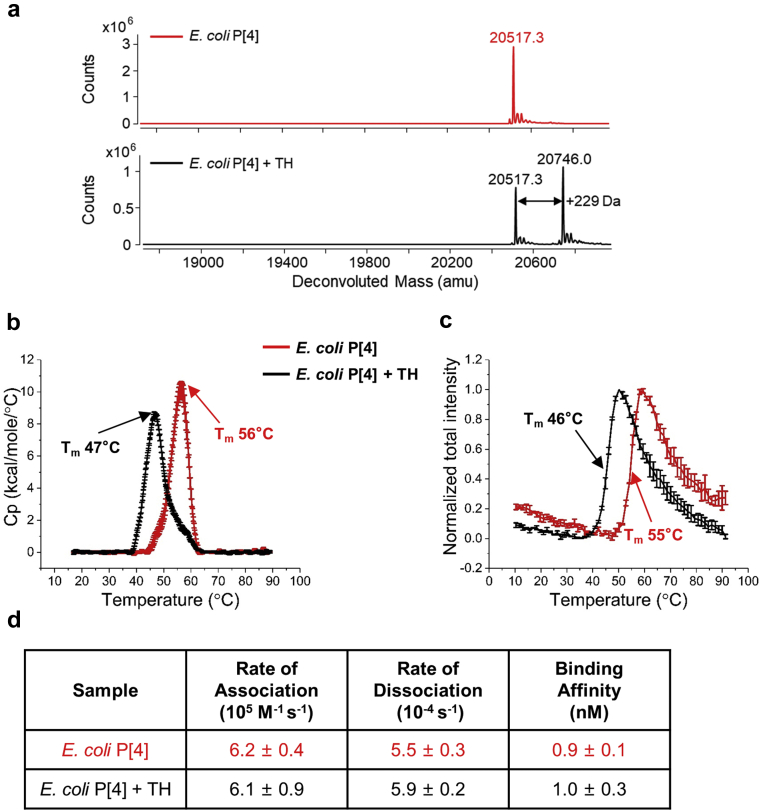
Effect of thimerosal (TH) on the structural integrity, conformational stability and antibody binding properties of *E. coli* expressed NRRV P[4] antigen (*E. coli* P[4]) as measured by: (a) Intact protein mass analysis; (b) Differential scanning calorimetry (DSC); (c) Extrinsic fluorescence spectroscopy; and (d) Octet BLI (*n* = 3, 1 SD). The *E. coli* P[4] samples were prepared in PBS buffer, pH 7.2 in the presence and absence of 0.01% w/v TH. For DSC and extrinsic fluorescence spectroscopy data, error bars represent 1 SD from triplicate measurements and the indicated T_m_ value is the average value.

A combination of analytical techniques was used to examine the effect of TH addition on the primary structure, higher-order structure, conformational stability, and antibody binding of *E. coli* P[4] (Fig. 1). Previous literature reports have observed that ethylmercury, a decomposition product of TH, can interact with the free cysteine residues of proteins.^18^^,^^19^ Intact protein mass analysis of *E. coli* P[4] before and after TH addition showed a +229 Da mass adduct (equivalent to the mass of ethylmercury), consistent with the formation of protein-ethylmercury adduct via the only free cysteine, C173, of P[4] protein (Fig. 1a). Differential scanning calorimetry (DSC) and extrinsic fluorescence spectroscopy were employed to determine the effect of TH on higher-order structural integrity and stability of *E. coli* P[4]. The presence of TH decreased the thermal melting temperature (T_m_) value by 9°C as measured by each method (Fig. 1b and c, respectively). These TH-induced P[4] destabilization results are in agreement with our previous findings with another NRRV antigen, *E. coli* P[8].^22^^,^^23^ Lastly, the *E. coli* P[4] showed low nM binding affinity for P[4]-specific mAb with no notable changes in the kinetic and binding constants upon addition of TH as measured by Octet BLI analysis (Fig. 1d).

**Fig 2:**
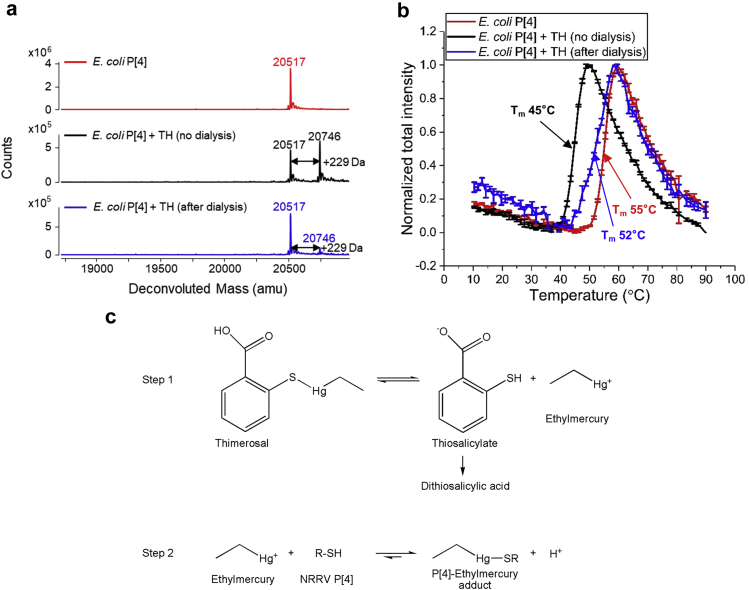
Effect of dialysis on the *E. coli* P[4] protein interaction with thimerosal (TH) and TH-induced changes in conformational stability. Three different P[4] samples were evaluated including: control *E. coli* P[4] (red), *E. coli* P[4] after addition of 0.01% w/v TH (black), and *E. coli* P[4] with 0.01% w/v TH after six rounds of dialysis versus PBS pH 7.2 buffer (blue). Samples were analyzed by (a) Intact protein mass analysis, and (b) Extrinsic fluorescence spectroscopy versus temperature. Error bars represent 1 SD from triplicate measurements and the indicated T_m_ value is the average value. (c) A working mechanistic model (adapted from Trümpler *et al.*^19^) of P[4] protein-TH interactions based on results shown in Fig. 2a and b.

The nature and potential reversibility of TH-P[4] protein interactions were then examined by comparing TH-treated *E. coli* P[4] samples before and after dialysis using a combination of intact protein mass analysis and extrinsic fluorescence spectroscopy (Fig. 2). Upon addition of TH, intact protein mass analysis indicated that *E. coli* P[4] immediately forms a +229 Da mass adduct of P[4] protein with ~60% relative ion abundance (Fig. 2a, black), a value that remained constant irrespective of varying incubation times and TH concentrations (data not shown). Dialysis of *E. coli* P[4]+TH samples (to remove free TH and any degradants) resulted in a substantial decrease in the relative ion abundance of the P[4]-ethylmercury adduct, suggesting reversibility of P[4] and TH interaction (Fig. 2a, blue). Moreover, addition of the alkylating agent iodoacetamide (IAA) interfered with the formation of C173-linked P[4]-ethylmercury adducts as detected by LC-MS peptide mapping analysis (Supplementary Fig. S2). Next, using extrinsic fluorescence spectroscopy, we observed that although the T_m_ values decreased by 10°C upon TH addition, the T_m_ value only decreased by 3°C after dialysis, a result supporting the reversibility of TH interaction with *E. coli* P[4] (Fig. 2b). Interestingly, the *E. coli* P[4] + TH + dialysis sample (blue trace) showed a broader, likely multicomponent thermal transition compared to the sharper, likely single component transition observed with the control samples (*E. coli* P[4] alone (red trace) and *E. coli* P[4]+TH (black trace)). The heterogeneity observed in *E. coli* P[4] + TH + dialysis sample could involve both irreversible structural alterations as well as the presence of native and ethylmercury bound protein (as observed by intact protein mass analysis, Fig. 1a).

Together, these results point toward the “partially reversible” nature of the TH interaction with P[4]. A working mechanism of P[4]-TH interaction adapted from Trümpler *et al.*^19^ and based on our analysis of TH-treated *E. coli* P[4] samples (before and after dialysis) is shown in Fig. 2c. It is a multi-step process involving TH degradation in aqueous solution, and the reversible interaction of the byproduct ethylmercury with the single free cysteine residue in P[4] protein (see Discussion).

### HX-MS analysis of thimerosal (TH)-induced structural alterations to E. coli P[4]

**Fig 3:**
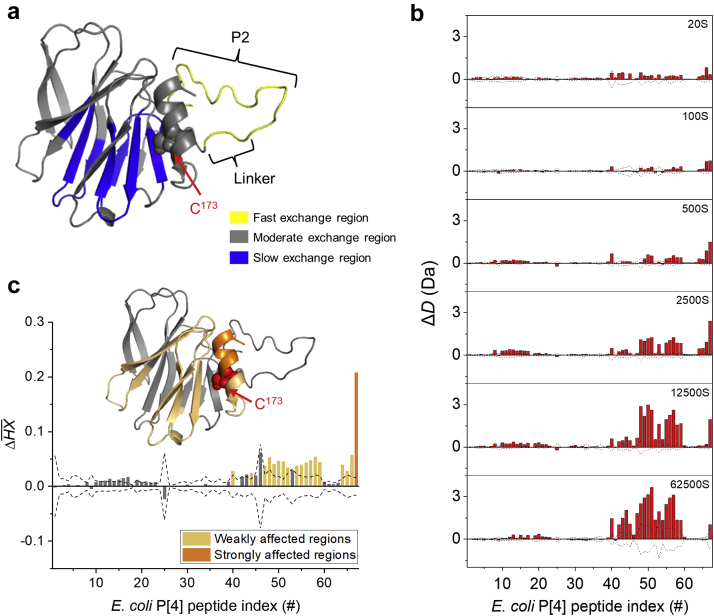
HX-MS analysis of relative inherent flexibility of *E. coli* P[4] protein and the effects of thimerosal (TH) addition. (a) Inherent relative flexibility of *E. coli* P[4] as measured by HX-MS mapped on the homology model of P[4]. The regions of relatively fast (ΔHX >80%, yellow), moderate (20% ≤ ΔHX ≤80%, gray), and slow (ΔHX <20%, blue) hydrogen exchange by the peptic peptides of P[4] (see Supplemental Fig. S3) are color-coded onto the P[4] homology model; (b) Representative Δ*D* plots (*E. coli* P[4] + 0.01% TH vs. *E. coli* P[4] alone) at individual labeling time points between 20 to 62,500 s; and (c) HX-MS *k*-means clustering analysis based on the mean ΔHX of *E. coli* P[4] + 0.01% TH versus *E. coli* P[4] alone. Positive bars in panels (b) and (c) indicate faster hydrogen exchange (i.e., greater local backbone flexibility) of *E. coli* P[4] + 0.01% TH versus *E. coli* P[4] alone. Bars outside the dashed line indicate significantly different values as determined from three technical repeats of the sample. The results of *E. coli* P[4]-TH interaction (panel B, C) have been mapped onto the homology model of P[4] (panel C) with dark orange and light orange indicating strongly and weakly affected regions, respectively. The only cysteine residue in P[4], C173, is labeled. In both (b) and (c) the peptides are indexed from N-terminal to C-terminal as identified in the Supplemental Figure S3.

Next, we utilized hydrogen exchange-mass spectrometry (HX-MS) to better understand the inherent flexibility of *E. coli* P[4] and the structural impact of the interaction of TH with *E. coli* P[4]. A total of 67 peptic peptides were generated and confirmed for *E. coli* P[4] with 100% sequence coverage^38^ (Supplementary Fig. S3A). The inherent flexibility of *E. coli* P[4] was then established by analyzing the HX-MS data across six different labeling time points, and the peptides were rank-ordered into three groups based on the hydrogen exchange after 2500 s of labeling time: fast (ΔHX >80%), moderate (20%≤ ΔHX ≤80%), and slow (ΔHX <20%) exchange regions (Supplementary Fig. S4). These results were mapped onto a previously described homology model^26^ of the P[4] protein (Fig. 3a). As expected, the unstructured *N*-terminal P2 epitope and linker region of *E. coli* P[4] (residues M^1^-L^23^ corresponding to ΔHX rank 62-67 in Supplementary Fig. S4) showed fastest hydrogen exchange and were assigned as the fast exchange region (highlighted in yellow, Fig. 3a). Parts of the central β-sheet core of P[4], including residues Y^38^-L^41^ (ΔHX rank 2), E^52^-I^64^ (ΔHX rank 1, 3, 4, 7, and 9), F^98^-M^100^ (ΔHX rank 6), V^122^-F^134^ (ΔHX rank 5, 8, 10, and 12) and I^154^-F^162^ (ΔHX rank 11, 13, and 14) showed slowest hydrogen exchange (Supplementary Fig. S4) and were assigned as slow exchange regions (highlighted in blue, Fig. 3a). The rest of the P[4], including the helical region containing C173 residue involved in TH interactions (ΔHX rank 18, 50, and 53), were thus found to be in a relatively moderate exchange region of the protein (indicated in gray, Fig. 3a).

Further, HX-MS analysis of *E. coli* P[4] with versus without 0.01% TH was performed. No peptides with ethylmercury bound species were observed during the HX-MS analysis of P[4]-TH interaction, likely due to their lower abundance or interference by quench buffer containing 4M guanidinium chloride. The HX differences between *E. coli* P[4] with versus without the addition of TH at each of the six labeling times between 20 and 62,500 s are shown in Fig. 3b. In this analysis, the P[4] peptides are numbered sequentially from the most *N*-terminal to the most *C*-terminal peptides. The HX difference (ΔD) of each peptide was plotted by using the hydrogen exchange in the presence of 0.01% TH minus hydrogen exchange of *E. coli* P[4] alone. Although no significant differences in uptake were observed at 20 s of labeling time, by 500 s the helical region surrounding C173 (residues Y^163^-L^181^, peptides #64-67) showed significant increase in the HX exchange, indicating increased local flexibility in the helical region induced by TH addition. By 2500 s, additional regions covering the β-sheet core, residues V^122^-S^155^ (peptides #48-51, 53, and 55-59) also showed significant increase in the exchange. HX of these peptides from these two regions of P[4] remained faster in the presence of TH (compared to the no TH sample) through later labeling times, demonstrating increased flexibility of only these peptides induced by TH (Fig. 3b). To further establish that the structural change of *E. coli* P[4] was induced by TH, and not as a function of time over the course of experiments, a separate set of HX-MS experiments were performed. In that experiment, *E. coli* P[4] + TH samples were incubated and HX-MS data were collected every 4 h over a total of 24 h at 20°C and compared with *E. coli* P[4] alone samples with a 120 s labeling time. As shown in Supplementary Fig. S5, no significant HX differences were observed except the region close to the C173 residue, indicating the structural changes of *E. coli* P[4] were derived upon TH addition, and not over the course of experiments.

The exchange differences for each P[4] peptide at individual time points, in the presence versus absence of TH, were then averaged ($\text{Δ}\bar{HX}$ normalized to the theoretical maximal uptake),^39^ and the results were then mapped onto the P[4] homology model for structural visualization (Fig. 3c). The magnitude of altered $\text{Δ}\bar{HX}$ regions of P[4] upon the addition of TH were classified by *k*-means clustering into three categories and color coded accordingly: strong effects (dark orange); weak effects (light orange); insignificant effects (gray). It can be seen that the peptide covering residues *N*^174^-L^181^ (peptide #67) was strongly affected due to the interaction between *E. coli* P[4] and TH, while multiple adjacent peptides covering the surrounding helical region (residues Y^163^-C^173^, peptides #64-66) were weakly affected (Fig. 3c). Another set of weakly affected peptides covered parts of central β-sheet core of P[4], between residues M^100^-L^121^ (peptides #47-52) and V^122^-S^155^ (peptides #54-59).

### Interaction of TH With Pp P[4] and Pp P[4]-C173S

**Fig 4:**
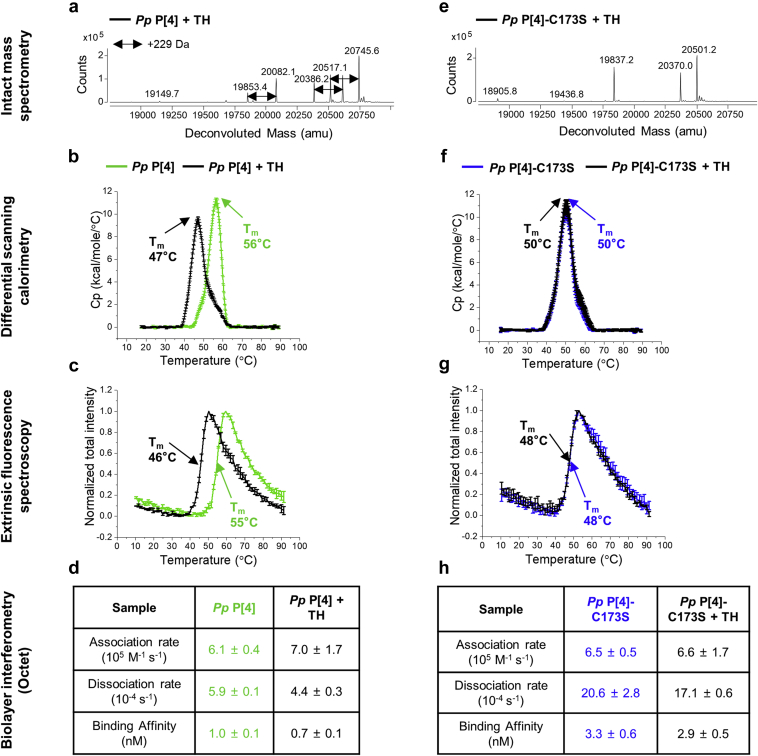
Effect of thimerosal (TH) on the structural integrity, conformational stability and antibody binding properties of *K. phaffii* expressed NRRV P[4] variants. (a) Intact protein mass analysis; (b) DSC; (c) extrinsic fluorescence spectroscopy; and (d) Octet BLI for *Pp* P[4], and (e) Intact protein mass analysis; (f) DSC; (g) extrinsic fluorescence spectroscopy; and (h) Octet BLI for *Pp* P[4]-C173S. Samples were prepared in PBS buffer, pH 7.2 in the presence and absence of 0.01% w/v TH. For DSC and extrinsic fluorescence spectroscopy, error bars represent 1 SD from triplicate measurements and the indicated T_m_ value is the average value.

*Pp* P[4]-C173S was generated with the goals of (1) validating the C173 residue as the site of TH binding, (2) further understanding the mechanisms of TH-induced destabilization of P[4], and (3) evaluating the extent that the *Pp* P[4]-C173S is able to resist TH-induced destabilization. The C173S variant was produced in *K. phaffii* as a part of evaluating the intensified manufacturing platform for low-cost production of recombinant vaccine antigens as described elsewhere.^23^^,^^31^ In order to account for possible host-related variations, parent P[4] protein was also produced in *K. phaffii*, and both P[4] proteins (*Pp* P[4] and *Pp* P[4]-C173S) were analyzed for changes in the primary structure, higher-order structure, conformational stability, and antibody binding upon addition of TH (Fig. 4).

The overall results for interaction of *Pp* P[4] with TH were similar to that observed for *E. coli* P[4], however, the former displayed some unique aspects compared to the latter (see Fig. 1 vs. Fig. 4). Intact protein mass analysis of *Pp* P[4] demonstrated an identical molecular weight for the main species as that of *E. coli* P[4] as well as a similar formation of +229 Da mass adduct in presence of TH (Supplementary Figs. S1B and 4a). In contrast, while the *E. coli* P[4] contained only the full-length P[4] species, *Pp* P[4] showed presence of several, low level *N*-terminal truncated species, 20,386 Da, 19,853 Da, and 18,921 Da, along with the full-length, 20,517 Da P[4] species (identity based on molecular weight values with the average measured masses within 0.2-0.3 Da of the theoretical masses; see Supplementary Fig. S1B). Additionally, upon addition of TH, multiple adducts were observed with +229 Da mass increase (Fig. 4a). DSC and extrinsic fluorescence spectroscopy results showed similar T_m_ values and a similar decrease of 9°C in the T_m_ value for *Pp* P[4] in the presence of TH (Fig. 4b and c). The antibody-binding analysis by Octet BLI showed low nM affinity of *Pp* P[4] to a P[4]-specific mAb with no major changes in the kinetic constants upon addition of TH (Fig. 4d). In summary, full-length *E. coli* P[4] and *Pp* P[4] (containing some low levels of *N*-terminal truncated species) showed similar overall physicochemical and antibody-binding properties as well as sensitivity to the addition of the preservative TH (Fig 1, Fig 4).

A similar set of physicochemical experiments were then performed to evaluate the C173S P[4] variant (*Pp* P[4]-C173S). The *Pp* P[4]-C173S contained full-length species, 20,501 Da, along with several N-terminal truncated species, 20,370 Da, 19,837 Da, and 18,906 Da with average measured masses within 0.3-0.4 Da of the theoretical masses (Supplementary Fig. S1B), thus displaying a similar profile to *Pp* P[4] (both produced in *K. phaffii*). In contrast to the parent protein, intact protein mass analysis of *Pp* P[4]-C173S showed no adduct formation upon addition of TH (Fig. 4e). This observation confirms that C173 of P[4] is the site of interaction of the ethylmercury (present from degradation of TH in solution). DSC results showed a single major endothermic peak with a T_m_ value of 50°C, irrespective of the presence or absence of TH (Fig. 4f). Similarly, extrinsic fluorescence spectroscopy showed the T_m_ value of *Pp* P[4]-C173S (48°C) to be the same in the presence and absence of TH (Fig. 4g). It can be seen, however, that the T_m_ value of *Pp* P[4]-C173S was ~6°C-7°C lower than *E. coli* P[4] and *Pp* P[4]. Based on these results, *Pp* P[4]-C173S is resistant to TH-induced destabilization, but inherently less stable compared to *E. coli* P[4] and *Pp* P[4]. Lastly, mAb-binding studies using Octet BLI indicated comparatively faster dissociation (~3-4X) and consequently, a slightly lower binding affinity (~3 nM vs. 1 nM) for *Pp* P[4]-C173S relative to *E. coli* P[4] and *Pp* P[4], with no notable changes in binding constants observed upon the addition of TH (Fig. 4h).

### HX-MS Analysis of Thimerosal (TH)-Induced Structural Alterations to Pp P[4] and Pp P[4]-C173S

**Fig 5:**
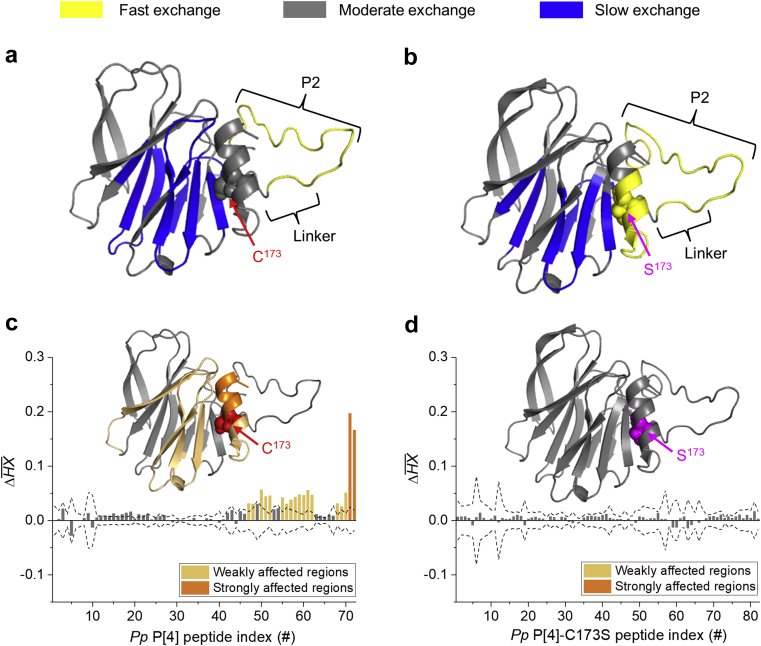
HX-MS analysis of inherent flexibility and thimerosal (TH) interactions with *K. phaffii* expressed NRRV P[4] parent protein (*Pp* P[4]) and the C173S variant (*Pp* P[4]-C173S). Inherent relative flexibility as measured by HX-MS of (a) *Pp* P[4] and (b) *Pp* P[4]-C173S are mapped onto the P[4] homology model. The regions of relatively fast (ΔHX >80%, yellow), moderate (20% ≤ ΔHX ≤80%, gray), and slow (ΔHX <20%, blue) hydrogen exchange by the peptic peptides of each P[4] sample are color-coded onto the P[4] homology model. HX-MS k-means clustering analysis based on the mean ΔHX of (c) *Pp* P[4] + 0.01% TH versus *Pp* P[4] alone, and (d) *Pp* P[4]-C173S + 0.01% TH versus *Pp* P[4]-C173S alone. Bars outside the dashed line indicate significantly different values as determined from three technical repeats of the sample. The results of TH interaction of *Pp* P[4] and *Pp* P[4]-C173S have been mapped onto the P[4] homology model with dark orange and light orange representing strongly and weakly affected regions, respectively. C173 in *Pp* P[4] and S173 in *Pp* P[4]-C173S are labeled.

HX-MS was utilized to further understand the effect of the mutation of cysteine residue to serine on the inherent flexibility of P[4] as well as the nature of the interaction of P[4] with TH. The pepsin digestion patterns and sequence coverage maps of the 2 *K. phaffii* produced P[4] proteins (*Pp* P[4] and *Pp* P[4]-C173S) were overall similar with a total of 72 and 82 peptic peptides, respectively and with 100% sequence coverage (Supplementary Fig. S3B and C), and these results were also similar to that observed with *E. coli* P[4] (see above). The inherent flexibility of these two P[4] molecules was established by analyzing the HX-MS data of *Pp* P[4] and *Pp* P[4]-C173S in same way as described above for the *E. coli* P[4] (Fig. 3), and the results were mapped onto the homology model of P[4] (Fig. 5a and b).

Consistent with *E. coli* P[4], the *Pp* P[4] also showed fast exchange in the region covering residues Y^3^-L^23^ (peptides #4-7, and 9) located in the *N*-terminal P2 epitope and linker region (highlighted in yellow). Sections of the β-sheet core of *Pp* P[4], including residues Y^38^-L^41^ (peptide #20), V^49^-I^64^ (peptides #26-31), F^98^-M^100^ (peptide #41), V^122^-T^143^ (peptides #48-49, 53, 56), and I^154^-F^162^ (peptide #63), were assigned as the slowly exchanging regions (highlighted in blue) (Fig. 5a). Similarly, the *Pp* P[4]-C173S also showed the flexible *N*-terminal P2 epitope and linker regions, including residues Y^3^-L^23^ (peptides #4, 6-9, and 12) as relatively fast exchange (highlighted in yellow) while residues Y^38^-L^41^ (peptide #22), F^59^-I^64^ (peptides #31-34), F^98^-F^101^ (peptides #48-49), V^122^-F^134^ (peptides #57-59, and 63), and I^154^-F^162^ (peptide #73) covering the central β-sheet core of *Pp* P[4]-C173S were shown to be relatively slowly exchanging (highlighted in blue) (Fig. 5b).

In contrast to either *E. coli* P[4] and *Pp* P[4], the *Pp* P[4]-C173S protein also showed another fast exchange region covering residues I^164^-E^175^ (peptide #81) situated in the helical region (Fig. 5b). This result indicates that mutation of the single cysteine residue to serine (C173S) led to an increase in the local backbone flexibility of the helical region surrounding the mutation site. Both *E. coli* P[4] and *Pp* P[4] contained low levels of dimer and other HMW species (≤5%) that consisted of non-native intermolecular disulfide bonds between C173 residues of individual monomers. Although such disulfide bonds between two monomers could potentially also result in increased protection (slower HX) near the disulfide region for *E. coli* P[4] and *Pp* P[4] (compared to *Pp* P[4]-C173S), no peptides containing disulfide cross-linked C173 were identified in the HX-MS analysis, possibly due to their overall low abundance after the pepsin digestion under the quenching condition. To further investigate the effect of C173S mutation of P[4], we also determined the HX uptake difference for each shared peptide between *Pp* P[4] and *Pp* P[4]-C173S (Supplementary Fig. S6). The difference plots highlighted amino acids in the helical region of P[4] surrounding C173S variant (peptides Y^163^-E^170^, Y^163^-L^181^, and Y^176^-L^181^) as the region with significantly greater HX uptake by *Pp* P[4]-C173S at multiple labeling time points (Supplementary Fig. S6). These observations further indicate that C173S change resulted in increased backbone flexibility in the helical region of the P[4] that contains the alteration.

Next, we analyzed and compared the interaction of TH with *Pp* P[4] versus *Pp* P[4]-C173S using HX-MS. The average HX differences of *Pp* P[4] and *Pp* P[4]-C173S in the presence versus absence of 0.01% TH, with results displayed in their corresponding homology models, are shown in Fig. 5c and d, respectively. The *Pp* P[4] showed regions of *N*^174^-L^181^ (peptides #71 and 72), next to C173, as strongly affected by TH (highlighted in dark orange), while residues Y^163^-C^173^ (peptides #68-70), and V^122^-S^155^ (peptides #47-48, 50-52, and 55-62) were affected weakly by TH (highlighted in light orange) (Fig. 5c). Thus, TH-destabilization results are very similar for P[4] produced in *K. phaffii* and *E. coli* (see Fig. 3 vs. Fig. 5). In contrast, the average HX difference plot of *Pp* P[4]-C173S showed no significant differences in local flexibility with and without TH (Fig. 5d), a result indicating that the local backbone flexibility of the helical region of *Pp* P[4]-C173S is not further affected by the addition of TH. Finally, we also compared the HX uptake of *Pp* P[4] in the presence of TH versus the *Pp* P[4]-C173S alone, which interestingly indicated no significant differences during the early labeling times (up to 500 s). At 12,500 s, however, faster exchange by *Pp* P[4] + TH was observed in the β-sheet core region (peptides #47-48, 50-51, and 53-58), and a larger global increase in hydrogen exchange (i.e., overall structural perturbation) was seen by 62,500 s (Supplementary Fig. S7). In summary, these combined results show the modification of the cysteine residue in P[4] protein, in comparison to the parent protein, resulted in increased local backbone flexibility and lower conformational stability, but at the same time, eliminated TH interactions and TH-induced structural destabilization of P[4].

### Characterization of P[4]-Specific mAb Binding to P[4] by HX-MS Epitope Mapping

**Fig 6:**
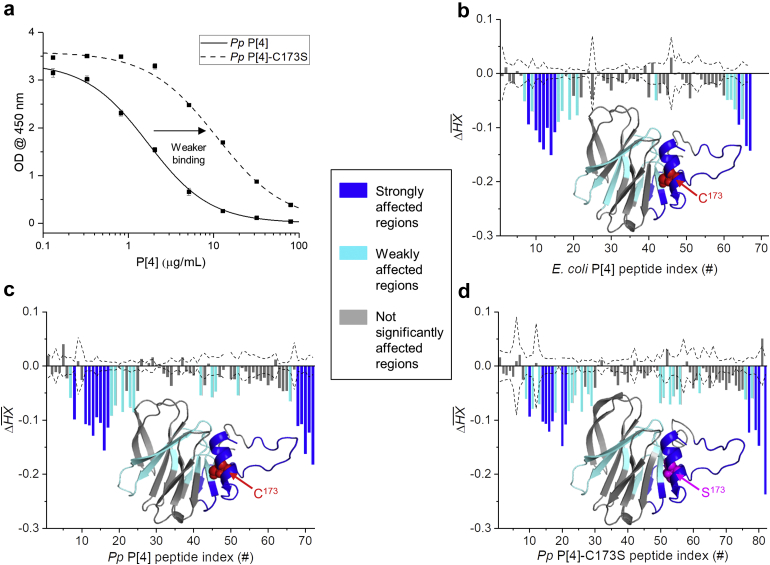
Antibody binding and epitope mapping of interaction sites of P[4]-specific mAb with various P[4] protein samples. (a) Inhibition ELISA results demonstrating relatively weaker antibody binding by *Pp* P[4]-C173S relative to *Pp* P[4]. HX-MS analysis based on the average HX differences of (b) *E. coli* P[4] with P[4]-specific mAb versus *E. coli* P[4] alone, (c) *Pp* P[4] with P[4]-specific mAb versus *Pp* P[4] alone, and (d) *Pp* P[4]-C173S with P[4]-specific mAb versus *Pp* P[4]-C173S alone. Negative bars indicate regions of slower hydrogen exchange (i.e., via the indicated peptic peptides of P[4] during HX analysis) of P[4] protein in presence of bound P[4]-specific mAb compared to P[4] alone. Bars outside the dashed line indicate significantly different values as determined from three technical repeats of the samples. The results of antibody binding have been mapped onto the homology model for each P[4] molecule with blue and cyan indicating strongly and weakly affected regions (that is, sites of protection against deuterium update indicating mAb binding regions to P[4], respectively. The C173 residue in *E. coli* P[4] and *Pp* P[4], and S173 residue in *Pp* P[4]-C173S are labeled.

As described above, *Pp* P[4]-C173S showed faster dissociation, and hence relatively weaker binding affinity for P[4]-specific mAb, when compared to *E. coli* P[4] or *Pp* P[4] as measured by antibody-antigen binding studies using Octet BLI. Complementary inhibition ELISA studies also displayed a clear shift in the OD_450_ curve, further confirming weaker antibody binding by *Pp* P[4]-C173S compared to *Pp* P[4] (Fig. 6a). We then employed HX-MS to better understand this weaker antibody binding and to identify the binding sites(s) of P[4]-specific mAb on P[4]. The application of HX-MS to identify the epitope binding regions between an antigen and antibody has been well-established as described elsewhere.^40^^,^^41^

For *E. coli* P[4], two regions including residues I^11^-W^39^ (peptides #8, and 10-15) and Y^163^-L^181^ (peptides #64, and 66-67) of P[4] protein were strongly affected (i.e., mostly protected) in the presence of P[4]-specific mAb (highlighted in dark blue, Fig. 6b), while four regions comprising residues L^40^-Y^51^ (peptides #19, 21), F^59^-I^64^ (peptide #25), F^101^-L^121^ (peptide #42), and I^156^-F^162^ (peptides #61-63) were more weakly protected (highlighted in light blue, Fig. 6b). Similarly, for *K. phaffii* produced *Pp* P[4], residues I^11^-W^39^ (peptides #8, and 11-17), and Y^163^-L^181^ (peptides #68-72) showed strongest protection (highlighted in dark blue, Fig. 6c), and L^40^-Y^51^ (peptides #21, 23-24), F^98^-G^123^ (peptides #42, 45-46), and I^157^-F^162^ (peptide #66) displayed weaker protection (highlighted in light blue, Fig. 6c).

For the *Pp* P[4]-C173S, residues I^11^-W^39^ (peptides #10, 14-17, and 20-21) and F^162^-L^181^ (peptides #77, 79-80, 82) were strongly affected (highlighted in dark blue, Fig. 6d), and L^40^-Y^51^ (peptides #26, 28-29), F^98^-G^123^ (peptides #50-51, 53-54, and 56), and I^157^-F^162^ (peptide #76) were weakly affected by P[4]-specific mAb (highlighted in light blue, Fig. 6d). These combined results indicate that P[4]-specific mAb recognizes a conformational epitope on P[4] protein that primarily involves the helix, the flexible *N*-terminal region, and some sections of the central β–sheet core. The observation that the helix surrounding C173 plays an important role in P[4]-specific mAb interactions with P[4] can explain the relatively lower binding affinity of P[4]-specific mAb for *Pp* P[4]-C173S compared to *Pp* P[4] and *E. coli* P[4].

## Discussion

Recent work in our laboratories has examined formulation development of a multi-dose, aluminum-salt adjuvanted non-replicating rotavirus (NRRV) subunit vaccine candidate, and demonstrated that the three recombinant protein antigens (P[4], P[6], and P[8]) were destabilized during storage in the presence of the commonly-used vaccine preservative thimerosal.^22^ Since multi-dose vaccines effectively lower costs (see introduction), and since the long-term goal of the development of the NRRV vaccine is to provide the broadest possible coverage in LMICs by combining it with the currently available childhood pentavalent vaccine (which contains thimerosal),^42^ we set out to explore two different approaches to develop a multi-dose NRRV vaccine formulation.

First, as presented in the companion paper in this issue,^23^ we designed a two-step formulation developability assessment approach for rapidly screening recombinant protein antigen candidates, as well as various formulation conditions, for their compatibility with preservatives as well as their storage stability when combined with adjuvants. As a case-study to demonstrate proof-of-concept, we screened eight different NRRV antigen variants, designed to improve developability when produced in *K. phaffii*^31^^,^^32^ and demonstrated incompatibility of the parent NRRV antigens with TH using (1) comparative analytical characterization data generated in a few days using minimal material (~1 mg), and (2) comparative 12 weeks accelerated/real-time stability study with down-selected candidates (~15-20 mg) under various formulation conditions including adsorption to aluminum adjuvants.^23^

The second approach, which is the focus of this work, was to elucidate the molecular mechanism(s) of thimerosal interaction with one of the NRRV antigens P[4], including the site of TH-interaction and subsequent effects on the protein’s structural integrity and conformational stability. We used physicochemical methods to examine TH-induced effects on the primary and higher-order structure of the three P[4] antigens (P[4] parent protein produced in *E. coli* and *K. phaffii* and C173S variant generated in the latter). Moreover, a combination of antibody-binding studies (Octet BLI) and changes in local backbone flexibility (HX-MS) studies were performed to determine site/regional locations within the P[4] where these effects occur.

HX-MS is a well-established higher-resolution technique for characterizing protein dynamics, conformational flexibility, protein-protein interactions including epitope mapping, and analyzing the effects of addition of excipients and engineered point mutations.^40^^,^^41^^,^^43^ We utilized HX-MS in two different ways to explore the structural basis for the conformational destabilization of P[4] antigen induced by thimerosal (TH) including (1) comparisons of the local backbone flexibility of P[4] protein (+/- TH), and (2) epitope-mapping studies of the antibody binding site to P[4] and correlating these results to Octet BLI determined P[4]-antibody binding data (+/- TH).

### Mechanism of TH Interaction With P[4]

Previous studies have shown that TH rapidly degrades to thiosalicylate and ethylmercury in aqueous solution, and that the byproduct ethylmercury reacts with the free thiol group of cysteine residues in proteins to form protein-ethylmercury adducts.^18^^,^^19^ In this work, consistent with the literature, P[4] adducts (+229 Da, equivalent to the mass of ethylmercury from TH) were observed by intact protein mass analysis upon addition of TH to the P[4] parent protein produced in either *E. coli* and *K. phaffii.* No ethylmercury adduct was observed with the C173S variant of P[4]. Additionally, LC-MS peptide mapping analysis identified two peptides, T^133^-Y^176^ and H^135^-Y^176^, bound to ethylmercury through the single cysteine residue of P[4] at C173. Together, these results verify the direct interaction of ethylmercury from TH to the P[4] protein via C173.

Intriguingly, our studies also provide evidence of partial reversibility to the formation of P[4]-ethylmercury adducts. Upon addition of IAA, an alkylating agent that binds covalently with the cysteine thiol groups, C173-linked P[4]-ethylmercury adduct peptides (Δ229 Da) were replaced with carbamidomethylation (Δ57 Da). Second, most of the P[4]-ethylmercury adduct could be removed upon dialysis with PBS buffer, although low level amounts of these adduct species (~5% relative ion abundance) remained even after 6-8 rounds of buffer exchange as detected by intact protein mass analysis. Together, these results indicate reversibility to the formation of P[4]-ethylmercury adduct by addition of competing compounds (IAA) or by concentration gradients (i.e., Le Chatelier’s principle during dilution by dialysis using a physiological buffer, PBS).

A working mechanism for the P[4]-TH interaction is proposed (Fig. 2c) based on results of this work and literature reports.^19^ In aqueous medium, TH degrades to two byproducts namely thiosalicylate and ethylmercury. Thiosalicylate can undergo dimer formation to generate dithiosalicylic acid, while ethylmercury binds to -SH group of the single free cysteine of P[4], C173 via a S-Hg coordinate bond to form a P[4]-ethylmercury adduct. Some free protein may co-exist with the ethylmercury-bound protein likely due to limited surface-exposure and solvent accessibility of the cysteine residue. Finally, due to the weak, reversible nature of the S-Hg coordinate bond, this protein-TH interaction is at least partially reversible upon dialysis due to the concentration gradient of the reactants resulting in additional accumulation of free P[4] protein.

### TH-Induced Structural Alterations of P[4]

TH-induced modification of Cys173 residue of P[4] (i.e., coordination of ethylmercury) resulted in structural destabilization of *E. coli* P[4] and *Pp* P[4] as observed by a 9°C reduction in T_m_ values as measured by DSC and extrinsic fluorescence spectroscopy. After dialysis of the TH-treated P[4] protein, the comparative difference in T_m_ values (vs. untreated P[4] control) was reduced to 3°C. Together, these results indicate reversibility to the formation of P[4]-ethylmercury adduct, with a likelihood that the TH-induced structural alterations within P[4] protein may be at least partially irreversible in nature.

We used HX-MS analysis to examine the structural elements of the P[4] antigen that are perturbed upon addition of TH. For both *E. coli* P[4] and *Pp* P[4], upon addition of TH, an increase in hydrogen exchange around the helical region was observed indicating an increased backbone flexibility of the helix. The middle of this helix is the location of the free cysteine, C173, involved in P[4]-ethylmercury adduct formation. Interestingly, and perhaps more surprisingly, regions of the β-sheet core located far-removed from the ethylmercury coordinated cysteine residue also showed increased hydrogen exchange upon TH addition at longer labeling times. This observation suggests that the structural changes induced by TH were detectable not only at short HX labeling times around the helix region, but also at longer labeling times since the β-sheet core is intrinsically more rigid and thus takes more time to show significant deuterium uptake differences.

These studies demonstrate that TH-induced structural destabilization of P[4] involves increased flexibility in the helical region (containing the ethylmercury modified cysteine residue) as well as more global structural alterations within the P[4] antigen. This global structural destabilization is consistent with the lower conformational stability of the P[4] antigen observed in the presence of TH as measured by DSC and extrinsic fluorescence spectroscopy. Such global structural alterations of the P[4] protein may expose new, otherwise buried, regions of the protein to deleterious interactions that may cause irreversible structural changes in the protein antigen (especially when bound to aluminum adjuvant). In fact, such TH-induced interactions are likely the cause for the instability of the Alhydrogel-adsorbed NRRV P[4] antigen in the presence of TH as described in the companion paper in this issue.^23^ Despite these observations of TH-induced physicochemical alterations within the NRRV P[4] antigen, their impact on *in vivo* immunogenicity remains to be determined in animal models as part of future work, especially since the nature of the neutralizing epitopes remains unknown (i.e., conformational vs linear epitopes). Regardless, a better understanding of preservative-induced destabilization of protein antigens is important to ensure both manufacturing consistency and long-term storage stability of multi-dose recombinant subunit vaccine formulations.

### Effect of C173S Alteration on Structural Stability of the P[4] With and Without TH Addition

Cysteine residues are known to play an important role in the structure, biological activity, and conformational stability of proteins.^44^ When present in protein therapeutics, however, free cysteine residues can be a source of chemical instability, reduced shelf-life, and may induce undesirable immunogenic aggregates.^44^^,^^45^ For this reason, protein-based therapeutic candidates can be designed to avoid the reactivity of the sulfhydryl group of cysteines by altering the free cysteine residue, most commonly with a serine residue.^44^^,^^46^ Cysteine to serine modification has been successfully introduced into several commercial protein therapeutics including Proleukin®, Betaseron® and Neulasta®.^47^ The relatively high hydrophobicity index of cysteine, however, is also an important consideration when the free cysteine residue is buried within the hydrophobic core of protein.^48^ Mutation of a buried free cysteine into serine has often been seen to reduce thermal stability in many proteins.^49^, ^50^, ^51^

In this work, similar to the observations in literature, an engineered P[4] variant (*Pp* P[4]-C173S) displayed lower conformational stability (~6°C-7°C lower T_m_ values) compared to *E. coli* P[4] or *Pp* P[4]. Concomitantly, the *Pp* P[4]-C173S protein displayed improved properties in the presence of TH. Upon TH addition, *Pp* P[4]-C173S did not form ethylmercury adducts, remained resistant to conformational destabilization, and showed no change in local flexibility. Interestingly, the increased flexibility in the helix region of P[4] induced by the cysteine alteration was comparable to the effect of TH modification of the free cysteine residue in *E. coli* P[4] or *Pp* P[4]. However, the P[4] parent protein experienced increased levels of structural perturbation, as revealed by increased HX at longer labeling times, indicating increased flexibility throughout the core of protein due to interaction with TH, an effect lacking in *Pp* P[4]-C173S. This observation likely explains why the P[4] parent proteins in presence of TH (T_m_ value of 47°C) have lower conformational stability than that observed with *Pp* P[4]-C173S protein (T_m_ value of 50°C).

The decreased conformational stability of *Pp* P[4]-C173S correlates with its increased inherent local flexibility as observed by HX-MS analysis. Along with the expected fast exchange flexible P2 linker region (also seen with *E. coli* P[4] and *Pp* P[4]), an additional fast exchange region localized in the helix containing the C173S change was observed with *Pp* P[4]-C173S. It should be pointed out that when making such comparisons between homologous backbone segments of two protein variants with different amino acid sequences, both backbone dynamics and intrinsic exchange rates should be considered.^52^^,^^53^ We thus calculated the theoretical chemical exchange rate for S^168^-A^177^ (a representative peptide in the P[4] helical region covering C173S mutation at pH_read_ 6.8, 20°C) using a chemical hydrogen exchange rate calculator.^54^^,^^55^ The results indicated (1) the S173 of *Pp* P[4]-C173S had a slower chemical exchange rate (11.0 s^-1^) than C173 of *Pp* P[4] (19.6 s^-1^), and (2) the adjacent *N*174 residue also had a slower chemical exchange rate for *Pp* P[4]-C173S versus *Pp* P[4]. These calculations (shown in Supplementary Table S1) indicate, that in the absence of any structural/dynamic effects, *Pp* P[4]-C173S should have had slower HX rate compared to *Pp* P[4] in the helical region comprising the C173S change. Since we experimentally observed the opposite effect (i.e., faster HX) in the helix with *Pp* P[4]-C173S, we can conclude that the observation of faster HX can be attributed to an increase in local backbone flexibility of the helical region due to the cysteine to serine alteration.

Based on these combined data, it can be summarized that the relative conformational stability of the *Pp* P[4]-C173S protein is essentially intermediate between the P[4] parent protein in the absence of TH versus the P[4] parent protein in the presence of TH. Such a rank-ordering was observed in the presence versus absence of TH for the storage stability profiles of Alhydrogel-adsorbed P[4] antigens (parent protein vs. C173S mutant), as described in the companion paper in this issue.^23^ In terms of future work, alternative sequence variants that conserve the hydrophobicity of cysteine, such as, alanine or valine, may be more appropriate than the “go-to” cysteine to serine change when the free cysteine residue is buried deep within the hydrophobic core.^44^ A double mutation approach that combines removal of buried free cysteine and some secondary alterations may also improve the thermal stability of mutant protein,^46^ while maintaining resistance to TH-induced destabilization.

### Interaction of P[4]-Specific mAb With Pp P[4] and Pp P[4]-C173S

HX-MS analysis can also provide peptide-level structural information to identify the epitope(s) for antigen-antibody interactions.^24^^,^^26^^,^^56^ Epitope mapping of P[4]-specific mAb indicated overlapping protected sites (interaction surface) on the P[4] parent protein and the cysteine mutant of P[4] comprised of the flexible *N*-terminal P2 linker region, the helical region with C173 in *Pp* P[4] (S173 in *Pp* P[4]-C173S), and regions of the central β–sheet core. Since protein-protein interactions often occur across large surfaces,^57^ it is not unexpected that a single point mutation in the epitope did not disrupt the binding interface. Differences in the signal strength for HX revealed that the helical region of P[4] became strongly protected when P[4]-specific mAb bound, suggesting that this region has an important role in the P[4]-specific mAb binding. The flexible P2 linker region also became strongly protected, however, the P2 linker region is not likely to be directly involved in P[4]-specific mAb binding because the P[4] variant lacking P2 and linker region showed no loss in mAb binding as measured by Octet (data not shown). The observed protection for the flexible P2 linker region in presence of P[4]-specific mAb is probably due to its close proximity to the helical region, presumably the key mAb epitope, which may cause non-specific interactions or partially occlude an otherwise fully solvent exposed region, resulting in slower hydrogen exchange. Lastly, the weak protection observed in the non-adjacent β–sheet core may be a result of binding-induced conformational changes or weak secondary interactions with P[4]-specific mAb.

We observed the crucial role of the helix encompassing C173 in the binding of P[4]-specific mAb by HX-MS epitope mapping, along with the relatively lower binding affinity of mAb for P[4] upon mutation of C173 by Octet and ELISA. Together, these results support the notion that the cysteine residue of the P[4] plays an important role in stabilizing the P[4] and P[4]-specific mAb complex. Interestingly, similar backbone dynamics was observed for the *Pp* P[4]-C173S protein compared to the P[4] parent protein in the presence of TH (i.e., cysteine adduct with ethylmercury). At the same time, as compared to the P[4] parent protein, the *Pp* P[4]-C173S protein showed weaker binding affinity to the P[4]-specific mAb, however, the TH-modified P[4] parent protein did not display a reduction in P[4]-specific mAb binding affinity. It is possible that this apparent indifference of P[4]-specific mAb binding to P[4] parent protein in the presence of TH may be due to the reversibility observed in TH binding, while changes in the conformation induced by the cysteine to serine mutation may permanently alter certain interactions of P[4] with P[4]-specific mAb, thus, resulting in a relatively weaker binding affinity.

## Conclusions and Future Work

The mechanism of thimerosal (TH) interaction with, and TH-induced destabilization of, the NRRV P[4] recombinant protein antigen was investigated through a combination of physicochemical techniques, antibody-antigen binding studies, and local flexibility changes via HX-MS analysis. The interaction between P[4] and TH occurred via the ethylmercury (an aqueous medium degradant of TH) through the C173 residue of P[4]. It occurs essentially instantaneously via a proposed S-Hg coordinate bond, resulting in enhanced local flexibility in the helical regions of P[4] proximal to the ethylmercury coordinated cysteine, followed by a more global structural alteration over time. Although the formation of ethylmercury adduct to the P[4] protein was partially reversible, its destabilizing impact to the structural integrity and conformational stability of P[4] was partially irreversible. Epitope mapping identified the helical region near C173 as a key binding site for P[4] and P[4]-specific mAb interaction, and thus this antibody-antigen binding analysis can be affected by TH addition or cysteine mutation. The *Pp* P[4]-C173S was not affected by the addition of TH, yet showed somewhat lower inherent conformational stability (T_m_ values decreased ~6°C) and several-fold lower antibody binding compared to the P[4] parent protein. Concomitantly, Alhydrogel-adsorbed *Pp* P[4]-C173S showed improved storage-stability (compared to the parent protein) in the presence of TH as shown in the companion paper.^23^ Future work will include further examining the interaction of P[4] with TH (i.e., nature of the “partial reversibility” of P[4]-ethylmercury adduct formation), as well as the evaluating TH interactions with the other two NRRV antigens, P[6] and P[8]. In addition, the effect of other commonly-used parenteral preservatives will be examined to identify alternatives to TH. Finally, the effect of preservative-antigen interactions on the *in vivo* immunogenicity will ultimately need to be determined with Alhydrogel-adsorbed antigen. Together, such future studies will further our understanding of how different types of preservatives will affect the ability to develop a multi-dose, preservative compatible, aluminum-adjuvant adsorbed NRRV vaccine candidate.

## Appendix A Supplementary Data

Supplemental Information

HX-MS Supplemental Information
