# Supplementary material for: Mechanism of Thimerosal-Induced Structural Destabilization of a Recombinant Rotavirus P[4] Protein Antigen Formulated as a Multi-Dose Vaccine
Source: J Pharm Sci. 2021 Mar;110(3):1054–66. doi: 10.1016/j.xphs.2020.11.033 (PMC7884053; doi:10.1016/j.xphs.2020.11.033)
Supplement: Supplemental Information [file mmc1.docx]

**Supplemental Information**

**Mechanism of thimerosal-induced structural destabilization of a recombinant rotavirus P[4] protein antigen formulated as a multi-dose vaccine**

Kawaljit Kaur ^a,g^, Jian Xiong ^a,f,g^, Nishant Sawant ^a^, Sanjeev Agarwal ^a,e^, John M. Hickey ^a^, David A. Holland ^a^, Tarit K. Mukhopadhyay ^b,f^, Joseph R. Brady ^c^, Neil C. Dalvie ^c^,

Mary Kate Tracey ^c^, Kerry R. Love ^c^, J. Christopher Love ^c^, David D. Weis ^d^,

Sangeeta B. Joshi ^a^, and David B. Volkin ^a,*^

^a^ Department of Pharmaceutical Chemistry, Vaccine Analytics and Formulation Center, University of Kansas, 2030 Becker Drive, Lawrence, KS 66047

^b^ Department of Biochemical Engineering, University College London, Bernard Katz Building, Gower Street, London WC1E 6BT, UK

^c^ Department of Chemical Engineering, Koch Institute for Integrative Cancer Research, Massachusetts Institute of Technology, Cambridge, MA 02139

^d^ Department of Chemistry and R.N. Adams Institute of Bioanalytical Chemistry,

University of Kansas, Lawrence, KS 66045

*Corresponding author.

*E-mail address*: [volkin@ku.edu](mailto:volkin@ku.edu) (D.B. Volkin).

^e^ Current addresses: Amgen, Thousand Oaks, California 91320.

^f^ Current addresses: Merck & Co., West Point, Pennsylvania 19486.

^g^ These authors contributed equally to this work.

**Supplementary Methods**

**Octet biolayer interferometry**

Antibody binding of various P[4] molecules in the presence and absence of thimerosal (TH) was determined with an Octet Red96 Biolayer Interferometry System (Pall Forte Bio LLC, Fremont, CA). P[4]-specific mAb was biotinylated using EZ-Link^TM^ Sulfo-NHS-LC-biotinylation kit following the manufacturer’s instructions, aliquoted, and stored at 4°C. Binding experiments were carried out in 96-well black microplates (Greiner Bio-One) using high precision streptavidin biosensors (Forte Bio, Cat No. 18-5117). Assay kinetics buffer (1X PBS pH 7.2 + 0.5% BSA + 0.05% tween 20) was used in the baseline, dissociation, and reference wells of the 96-well plates as well as for the dilution of mAb and various P[4] samples. For samples with TH, an additional reference well with 0.01% w/v TH alone was also included in the binding experiment. Prior to the run, biosensors were hydrated for ~15 min in the assay kinetics buffer. Binding assay for each sample was performed in triplicate using 1.25 µg/mL of mAb and seven point 1:2 serial dilutions of P[4] (± 0.01% w/v TH) with a starting concentration of 2 µg/mL. Association and dissociation steps were carried out for 300 and 600 sec, respectively, at 1000 rpm. Data analysis was performed using Octet Data Analysis software (v 10.0, Forte Bio). Following data processing (including reference well subtraction), association and dissociation traces of various P[4] samples were fit to a global 1:1 binding model using vendors software. Curve fitting analysis of the kinetics data yielded binding affinity and rate constants of the interaction.

**LC-MS peptide mapping**

*E. coli* P[4] stock was buffer exchanged into 150 mM NaCl, 10 mM phosphate, pH 7.2 buffer using 3 kDa MWCO spin centrifugal filters prior to sample preparation for LC-MS peptide mapping. For sample preparation of *E. coli* P[4] alone, the samples were incubated with 15 mM DTT and 1 mM EDTA for 30 min at 80ºC prior to digestion. For sample preparation of *E. coli* P[4] + TH, 0.01% w/v TH was added to the samples, which were then incubated with 15 mM DTT and 1 mM EDTA for 30 min at 80ºC prior to digestion. And lastly, for *E. coli* P[4] + TH + IAA condition, 0.01% w/v TH was added to the samples, which were then reduced using 15 mM DTT in presence of 1 mM EDTA for 30 min at 80ºC and alkylated with 30 mM iodoacetamide (IAA) for 30 min at room temperature. All samples were digested using chymotrypsin at 1:10 chymotrypsin:P[4] at 37ºC overnight. The proteolysis was quenched using 0.05% trifluoroacetic acid (TFA) and ~15 μg of digested protein was subjected to LC-MS. The peptides from the digested protein solution were separated by a liquid chromatography system (Thermo Scientific, Waltham, MA) prior to analysis. Peptides were injected onto an ACQUITY CSH C18 column (1.7 µm, 2.1 x 150 mm, Waters Corporation) with 45 min 0-30% B gradient (A: H_2_O and 0.05% TFA; B: Acetonitrile and 0.05% TFA; 0.2 mL/min flow rate) for separation. MS was performed using an LTQ-XL ion trap (Thermo Scientific) and the *Xcalibur* 2.0 software (Thermo Scientific). The instrument was also tuned using a standard calibration peptide (Angiotensin II, Sigma) for maximal sensitivity before running any experiments. The mass spectra were acquired in the LTQ over a mass range of m/z 400-1900. For MS^2^ measurements, fragmentation was achieved by collision-induced dissociation (CID). The ion selection threshold was 12,000 counts and the dynamic exclusion duration was 5 s. Raw experimental files were processed using *PepFinder* 2.0 software (Thermo Scientific). Potential Cys ethylmercury adduct from TH, Cys carbamidomethylation, Asn deamidation, and Met oxidation were included during the analysis.

**Supplementary Figures**

**Supplementary Figure S1.** (A) Reduced and non-reduced SDS-PAGE analysis of the three NRRV P[4] protein samples used in the study. Molecular Weight (MW) markers are shown in kDa values with (1) *E. coli* P[4], (2) *Pp* P[4], and (3) *Pp* P[4]-C173S (B) Intact protein mass analysis of *Pp* P[4] and *Pp* P[4]-C173S.


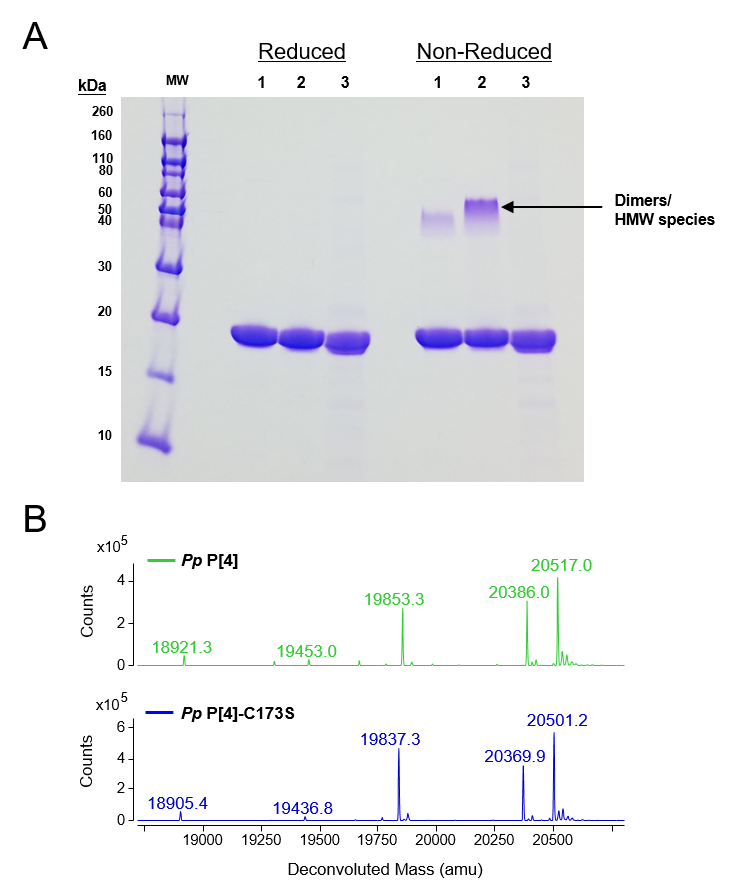


**Supplementary Figure S2.** MS^2^ analysis of the effect of addition of iodoacetamide (IAA) on P[4]-TH interaction and ethylmercury adduct formation of the cysteine-containing peptide identified by LC-MS peptide mapping. Peptide H^135^-Y^176^ indicating the observed ‘b’ and ‘y’ ions from reduced chymotrypsin-digested (1) *E. coli* P[4] incubated with 0.01% TH, (2) *E. coli* P[4] alone, and (3) *E. coli* P[4] incubated with 0.01% TH and alkylated with IAA prior to digestion. The theoretical and observed MH^+3^ precursor *m/z* for the selected peptides were 1661.74 and 1661.71 for *E. coli* P[4]+TH sample, 1585.40 and 1585.42 for *E. coli* P[4] sample, and 1604.40 and 1604.52 for *E. coli* P[4]+TH+IAA sample, respectively. The ethylmercury (from TH) and alkyl group (from IAA) binds to the –SH group of the single cysteine residue in the P[4] protein samples resulting in a mass change of +229 and +57 Da, respectively. Fragment ions shown in blue and red correspond to the ions showing no mass changes and ions showing a change in mass, respectively, following modification by TH or IAA.


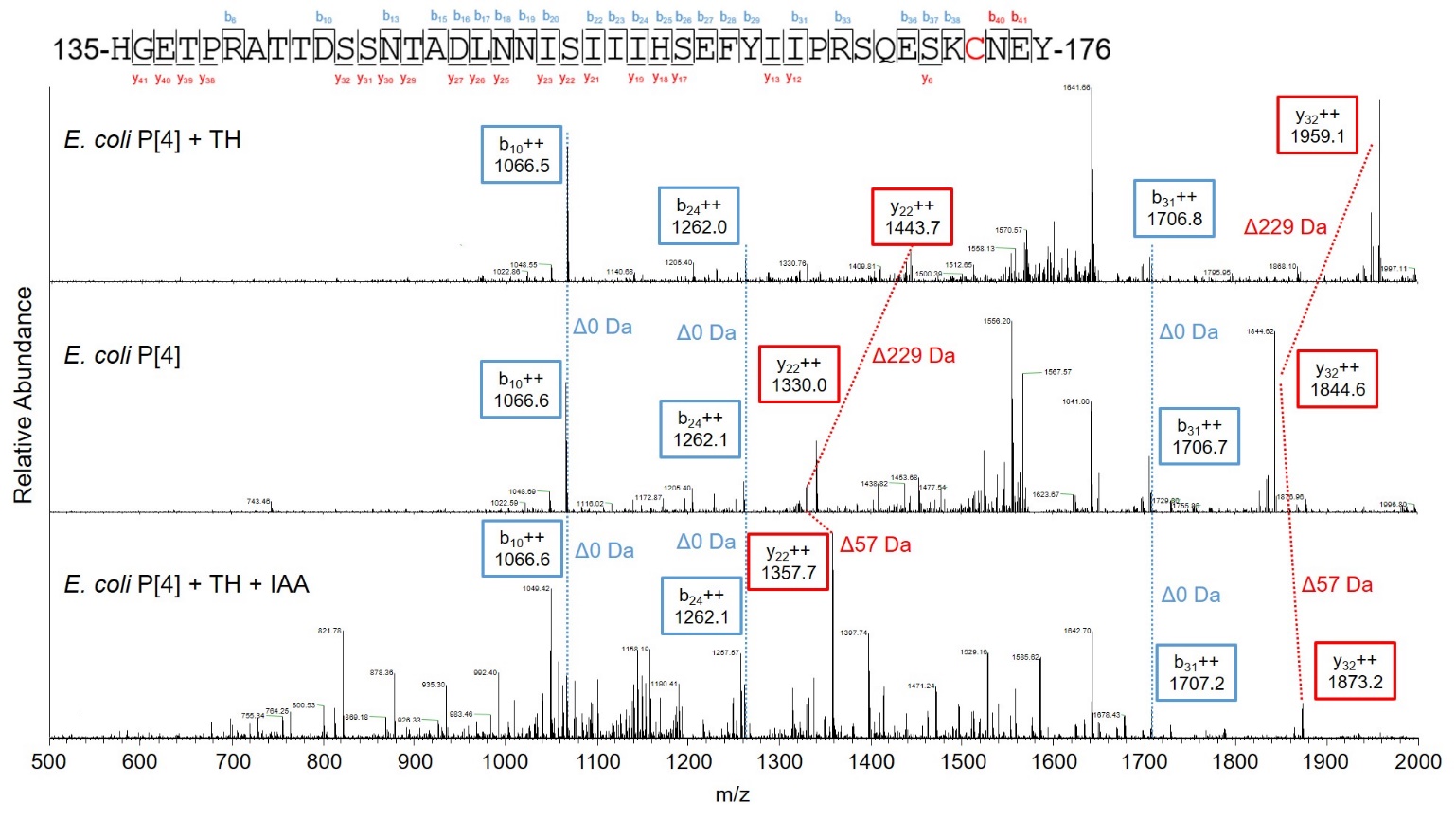


**Supplementary Figure S3.** Peptide coverage from pepsin digestion of the three P[4] samples during HX-MS analysis: (A) *E. coli* P[4], (B) *Pp* P[4], and (C) *Pp* P[4]-C173S. Each of the P[4] protein samples were on-line digested for 3 min by in-house packed pepsin columns. MS-MS data were used to confirm the identity of each peptide.


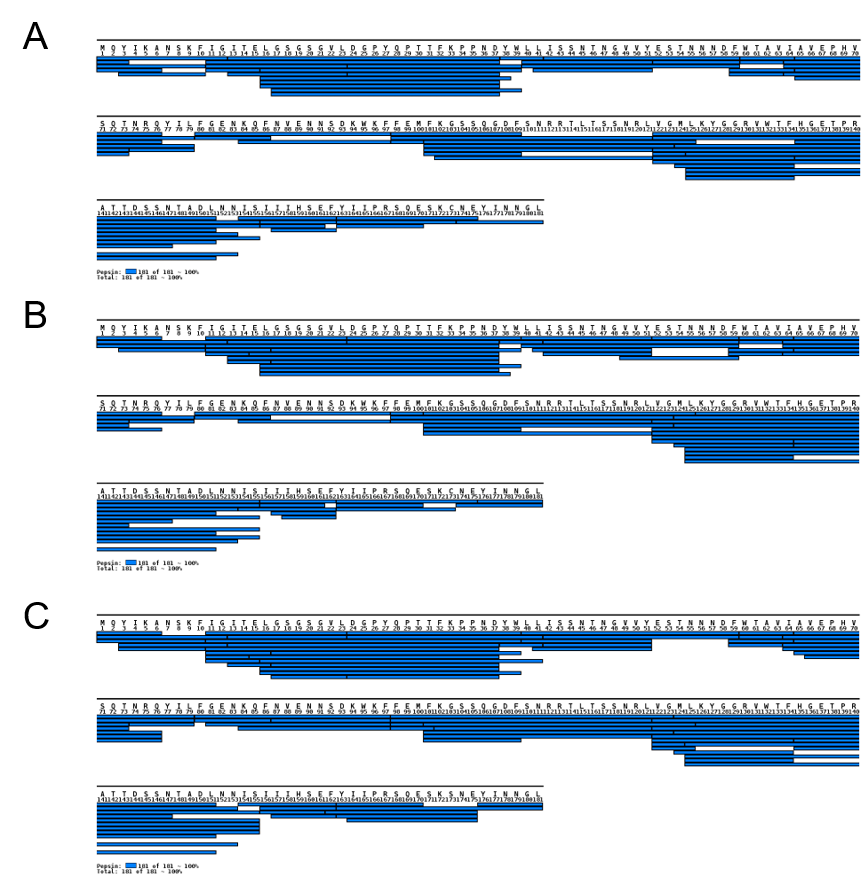


**Supplementary Figure S4.** Relative ranking of HX by peptides generated during HX-MS analysis of *E. coli* P[4]. The relative hydrogen exchange of each of the 67 peptides was rank-ordered from lowest to highest, based on the % HX at 2500 s labeling time. The results were then categorized into fast (> 80% exchange, yellow), moderate (≥ 20% to ≤ 80% exchange, gray), and slow (< 20% exchange, blue) regions. Note that the X-axis is the peptide rank according to HX and is not correlated to amino acid sequence of the P[4] protein.


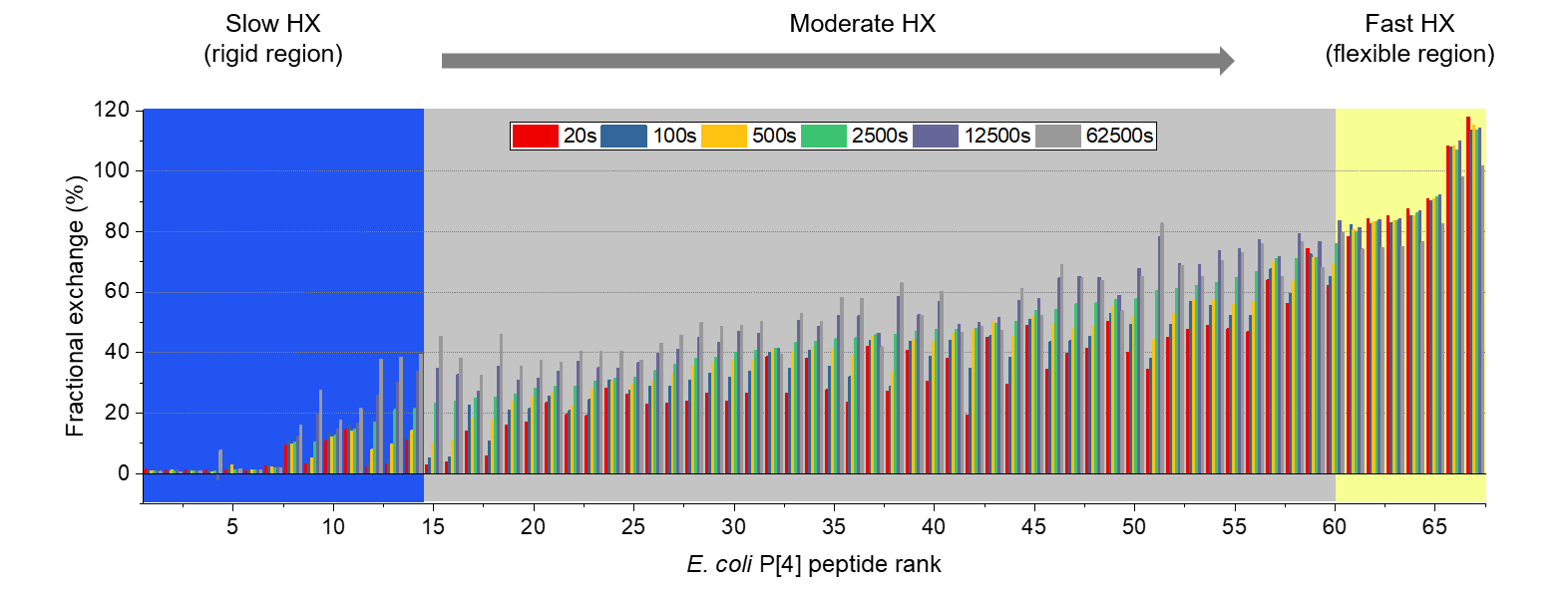


**Supplementary Figure S5.** (A) Comparison of HX between *E. coli* P[4] + TH with *E. coli* P[4] alone over 24 hours at 20°C. HX difference was calculated using the HX values of peptides from *E. coli* P[4] + TH at each incubation time point minus the HX of same peptides from *E. coli* P[4] at time0 (T0). All data were collected with a labeling time of 120 s. (B) Peptide coverage from pepsin digestion of *E. coli* P[4] for the HX-MS stability analysis. The sample was on-line digested for 3 min by in-house packed pepsin columns. MS-MS data were used to confirm the identity of each peptide. Note: This experiment was performed separately and thus has different peptide coverage.

**
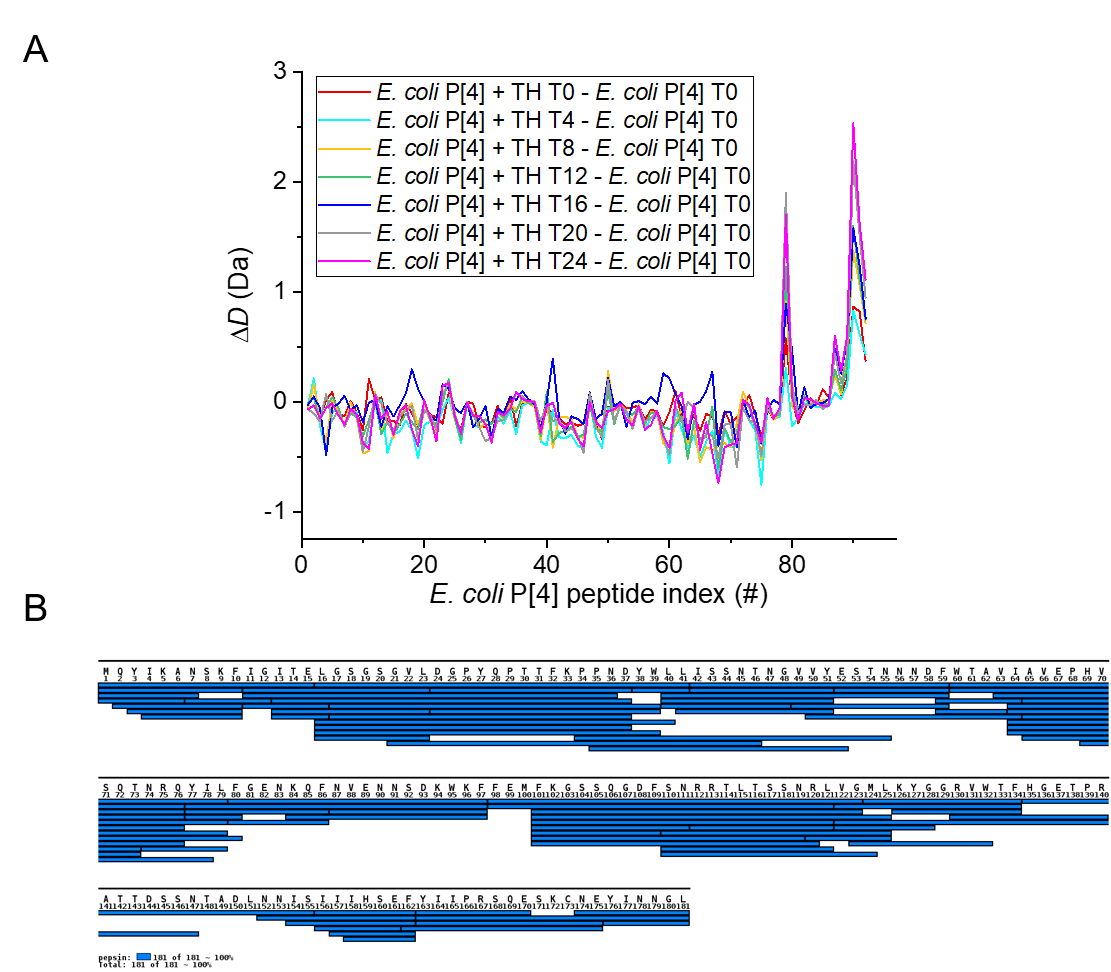
**

**Supplementary Figure S6.** Comparison of HX difference plots between *Pp* P[4] and *Pp* P[4]-C173S (A) Δ*D* (calculated using the HX values of peptides from the *Pp* P[4]-C173S minus the HX of same peptides from *Pp* P[4]) at six different labeling time points between 20 and 62500 s. (B) Averaged Δ*D* values from the individual time points. Positive bars in (A) and (B) indicate faster hydrogen exchange by *Pp* P[4]-C173S, correlated to its greater backbone flexibility, relative to *Pp* P[4]. Bars outside the dashed line indicate significantly different values determined from three technical repeats of the sample.


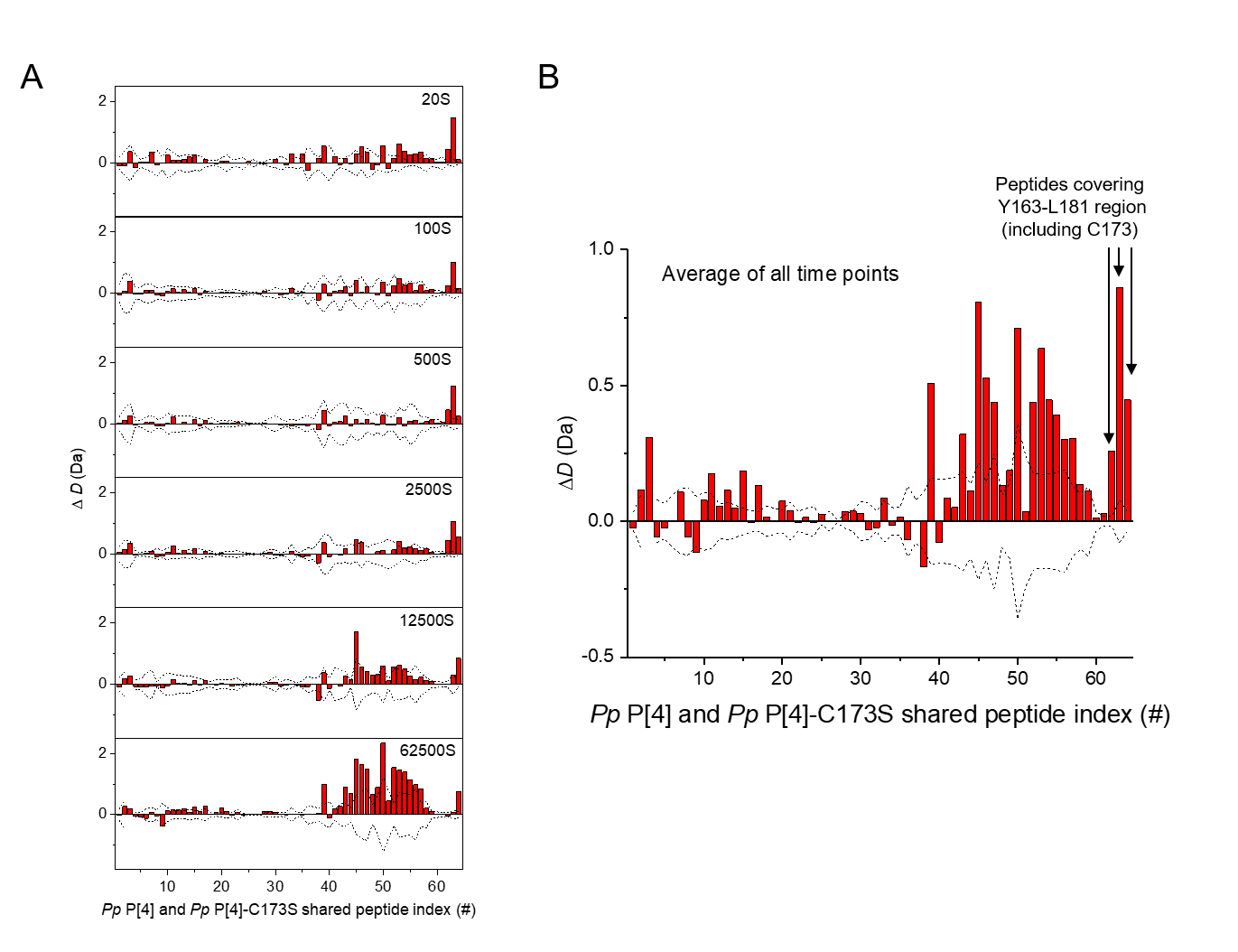


**Supplementary Figure S7.** Comparison of HX difference plots between *Pp* P[4]+TH and *Pp* P[4]-C173S. (A) Δ*D* in Da (calculated using the HX values of peptides from the *Pp* P[4]-C173S minus the HX of same peptides from *Pp* P[4]+TH) at six different labeling time points between 20 and 62500 s. (B) Averaged Δ*D* values in Da from the individual time points. Negative bars in (A) and (B) indicate slower hydrogen exchange by *Pp* P[4]-C173S, correlated to its lower backbone flexibility relative to *Pp* P[4]+TH. Bars outside the dashed line indicate significantly different values determined from three technical repeats of the sample.


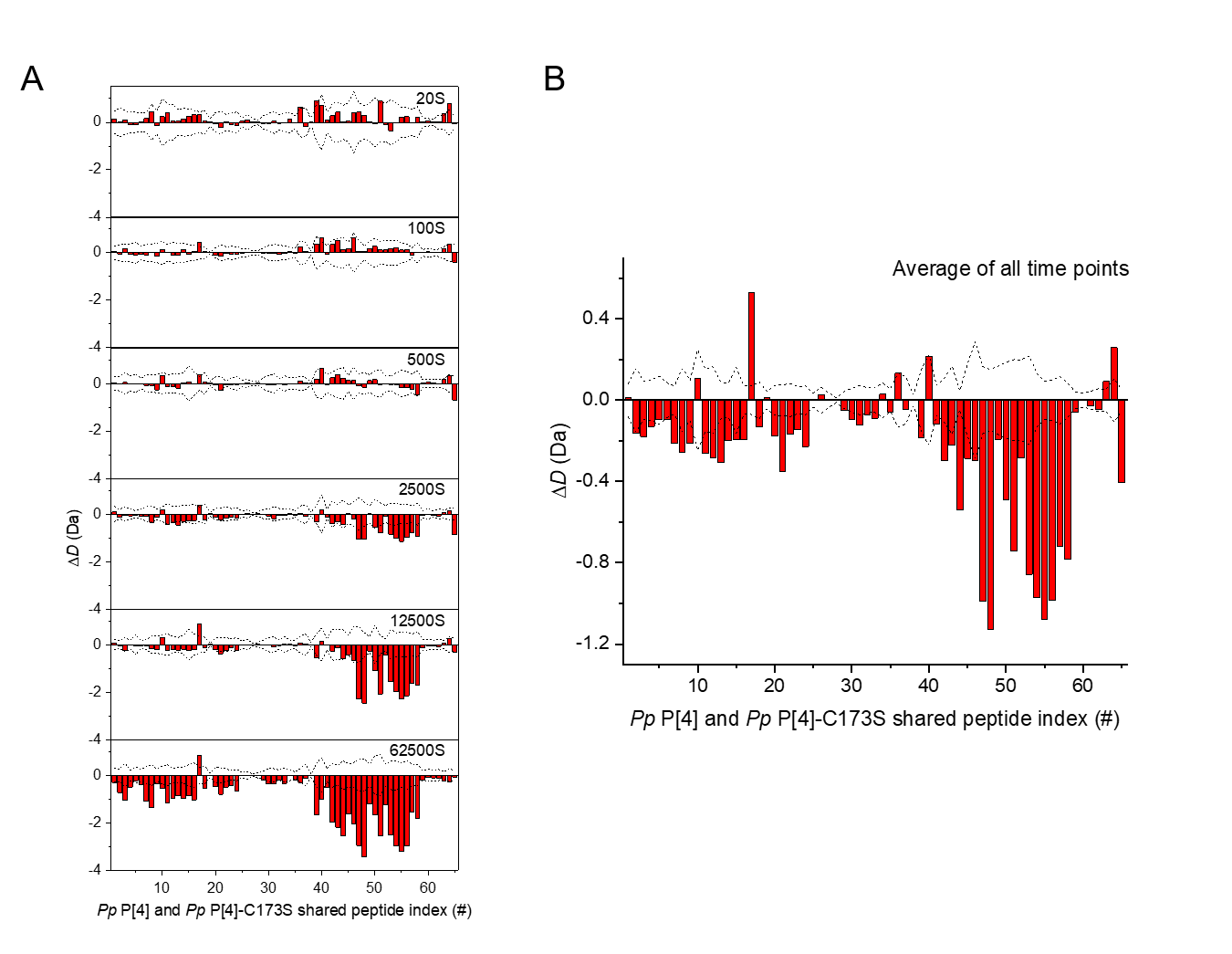


**Supplementary Table S1.** Theoretical calculations of the chemical exchange rate for peptides in the S168-N177 region of *Pp* P[4] and *Pp* P[4]-C173S. The specific peptide segment was selected empirically to cover the mutation site (C173S) and its adjacent residues. Results are based on the current 2018 version of the chemical exchange rate calculator (http://hx2.med.upenn.edu) with labeling conditions set to pH_read_ 6.8 at 20°C. NA for the N-terminal serine (S168) and glutamine (Q169) residues indicate no measurable chemical exchange rate.

| Amino acid | Residue # | *Pp* P[4]  (*k*_ch_, s^-1^) | *Pp* P[4]-C173S  (*k*_ch_, s^-1^) | % Ratio  (*Pp* P[4]-C173S/*Pp* P[4]) |
| --- | --- | --- | --- | --- |
| S | 168 | NA | NA | NA |
| Q | 169 | NA | NA | NA |
| E | 170 | 4.4 | 4.4 | 100 |
| S | 171 | 6.0 | 6.0 | 100 |
| K | 172 | 6.5 | 6.5 | 100 |
| C(S) | 173 | 19.6 | 11.0 | 56 |
| N | 174 | 39.1 | 22.0 | 56 |
| E | 175 | 5.8 | 5.8 | 100 |
| Y | 176 | 1.4 | 1.4 | 100 |
| A | 177 | 0.1 | 0.1 | 100 |
