# Supplementary material for: Mechanism of Thimerosal-Induced Structural Destabilization of a Recombinant Rotavirus P[4] Protein Antigen Formulated as a Multi-Dose Vaccine
Source: J Pharm Sci. 2021 Mar;110(3):1054–66. doi: 10.1016/j.xphs.2020.11.033 (PMC7884053; doi:10.1016/j.xphs.2020.11.033)
Supplement: HX-MS Supplemental Information [file mmc2.pdf]

**HX-MS Supplemental Information**

# Mechanism of thimerosal-induced structural destabilization of a recombinant rotavirus P[4] protein antigen formulated as a multi-dose vaccine

Kawaljit Kaur [a,g], Jian Xiong [a,f,g], Nishant Sawant [a], Sanjeev Agarwal [a,e], John M. Hickey [a], David A. Holland [a], Tarit K. Mukhopadhyay [b,f], Joseph R. Brady [c], Neil C. Dalvie [c], Mary Kate Tracey [c], Kerry R. Love [c], J. Christopher Love [c], David D. Weis [d], Sangeeta B. Joshi [a], and David B. Volkin [a,*]

[a] Department of Pharmaceutical Chemistry, Vaccine Analytics and Formulation Center, University of Kansas, 2030 Becker Drive, Lawrence, KS 66047

[b] Department of Biochemical Engineering, University College London, Bernard Katz Building, Gower Street, London WC1E 6BT, UK

[c] Department of Chemical Engineering, Koch Institute for Integrative Cancer Research, Massachusetts Institute of Technology, Cambridge, MA 02139

[d] Department of Chemistry and R.N. Adams Institute of Bioanalytical Chemistry, University of Kansas, Lawrence, KS 66045

*Corresponding author.

*E-mail address*: volkin@ku.edu (D.B. Volkin).

[e] Current addresses: Amgen, Thousand Oaks, California 91320.

[f] Current addresses: Merck & Co., West Point, Pennsylvania 19486.

[g] These authors contributed equally to this work.

**HX-MS experimental details, % deuterium uptake values and curves for the three NRRV P[4] protein samples used in the study: *E. coli* P[4], *Pp* P[4] (or P[4]-WT, wild type), and *Pp* P[4]-C173S (or P[4]-Cys M, cysteine mutant)**

| Data Set | *E. coli* P[4] | P[4]-WT | P[4]-Cys M |
|---|---|---|---|
| HDX reaction details | 10 mM phosphate buffer, $pD_{read}$ = 6.8, 20 °C | 10 mM phosphate buffer, $pD_{read}$ = 6.8, 20 °C | 10 mM phosphate buffer, $pD_{read}$ = 6.8, 20 °C |
| HDX time course (S) | 20, 100, 500, 2500,12500, 62500 | 20, 100, 500, 2500,12500, 62500 | 20, 100, 500, 2500,12500, 62500 |
| HDX full D control samples | N/A | N/A | N/A |
| Back-exchange (mean / IQR) | N/A | N/A | N/A |
| # of Peptides | 67 | 72 | 82 |
| Sequence coverage overall | 100% | 100% | 100% |
| Average peptide length | 14.8 | 15.1 | 15.6 |
| Replicates (biological or technical) | 3 (technical) | 3 (technical) | 3 (technical) |
| Repeatability | 0.088 (average standard deviation) | 0.074 (average standard deviation) | 0.100(average standard deviation) |
| Significant differences in HDX (delta HDX > X D) | depend on individual peptide | depend on individual peptide | depend on individual peptide |

| State | Start | End | Sequence | Search RT | Charge | Max D | 20s #D | 20s #D | 20s #D | 100s #D | 100s #D | 100s #D | 500s #D | 500s #D | 500s #D | 2500s #D | 2500s #D | 2500s #D | 12500s #D | 12500s #D | 12500s #D | 62500s #D | 62500s #D | 62500s #D |
|---|---|---|---|---|---|---|---|---|---|---|---|---|---|---|---|---|---|---|---|---|---|---|---|---|
| *E. coli* P[4] alone | 1 | 3 | MQY | 4.26 | 1 | 1 | 0.965 | 1.076 | 1.045 | 1.043 | 1.01 | 1.101 | 1.072 | 1.071 | 1.075 | 1.048 | 1.027 | 1.018 | 1.043 | 1.119 | 1.044 | 1.054 | 1.048 | 1.048 |
| *E. coli* P[4] alone | 1 | 6 | MQYIKA | 4.99 | 2 | 4 | 3.04 | 3.099 | 3.205 | 3.247 | 3.18 | 3.216 | 3.244 | 3.192 | 3.203 | 3.222 | 3.272 | 3.242 | 3.287 | 3.284 | 3.266 | 3.325 | 3.269 | 3.304 |
| *E. coli* P[4] alone | 1 | 10 | MQYIKANSKF | 5.88 | 3 | 8 | 5.713 | 5.804 | 5.984 | 5.901 | 5.884 | 5.989 | 5.895 | 5.891 | 5.88 | 5.909 | 6.024 | 5.946 | 6.017 | 6.055 | 5.975 | 6.072 | 5.993 | 6.06 |
| *E. coli* P[4] alone | 1 | 12 | MQYIKANSKFIG | 6.41 | 3 | 10 | 7.249 | 7.341 | 7.472 | 7.421 | 7.414 | 7.519 | 7.441 | 7.425 | 7.39 | 7.447 | 7.575 | 7.503 | 7.595 | 7.613 | 7.525 | 7.633 | 7.597 | 7.646 |
| *E. coli* P[4] alone | 3 | 10 | YIKANSKF | 4.36 | 3 | 6 | 4.194 | 4.07 | 4.323 | 4.526 | 4.311 | 4.506 | 4.424 | 4.489 | 4.411 | 4.392 | 4.518 | 4.453 | 4.481 | 4.531 | 4.457 | 4.601 | 4.447 | 4.592 |
| *E. coli* P[4] alone | 11 | 15 | IGITE | 4.47 | 1 | 3 | 2.749 | 2.782 | 2.864 | 2.855 | 2.85 | 2.897 | 2.884 | 2.842 | 2.837 | 2.883 | 2.934 | 2.886 | 2.943 | 2.934 | 2.955 | 2.949 | 2.921 | 2.962 |
| *E. coli* P[4] alone | 11 | 23 | IGITELGSGSGVL | 8.57 | 2 | 11 | 8.337 | 8.2 | 8.492 | 8.348 | 8.36 | 8.351 | 8.392 | 8.376 | 8.266 | 8.332 | 8.552 | 8.469 | 8.542 | 8.599 | 8.491 | 8.628 | 8.585 | 8.586 |
| *E. coli* P[4] alone | 11 | 37 | IGITELGSGSGVLDGPYQPTTFKPPND | 8.8 | 2 | 21 | 10.86 | 10.896 | 11.054 | 11.78 | 11.793 | 11.889 | 12.479 | 12.469 | 12.378 | 13.654 | 13.868 | 13.753 | 15.221 | 15.252 | 15.143 | 15.853 | 15.717 | 15.875 |
| *E. coli* P[4] alone | 13 | 23 | ITELGSGSGVL | 7.1 | 1 | 9 | 5.89 | 6.027 | 6.264 | 6.391 | 6.387 | 6.302 | 6.436 | 6.236 | 6.166 | 6.491 | 6.344 | 6.312 | 6.616 | 6.39 | 6.324 | 6.395 | 6.375 | 6.661 |
| *E. coli* P[4] alone | 13 | 37 | ITELGSGSGVLDGPYQPTTFKPPND | 8.07 | 2 | 19 | 9.09 | 8.875 | 9.196 | 9.862 | 9.834 | 10.109 | 10.668 | 10.639 | 10.538 | 11.822 | 12.055 | 11.871 | 13.297 | 13.354 | 13.221 | 13.996 | 13.898 | 14.038 |
| *E. coli* P[4] alone | 16 | 37 | LGSGSGVLDGPYQPTTFKPPND | 7.6 | 2 | 16 | 6.251 | 6.376 | 6.537 | 7.193 | 7.226 | 7.363 | 7.904 | 7.916 | 7.845 | 9.055 | 9.315 | 9.185 | 10.663 | 10.7 | 10.594 | 11.319 | 11.234 | 11.35 |
| *E. coli* P[4] alone | 16 | 38 | LGSGSGVLDGPYQPTTFKPPNDY | 8.24 | 2 | 17 | 6.14 | 6.169 | 6.321 | 6.994 | 7.012 | 7.1 | 7.641 | 7.719 | 7.648 | 8.706 | 9.012 | 8.853 | 10.443 | 10.35 | 10.295 | 11.078 | 10.966 | 11.114 |
| *E. coli* P[4] alone | 16 | 39 | LGSGSGVLDGPYQPTTFKPPNDYW | 9.8 | 3 | 18 | 5.648 | 5.794 | 5.966 | 6.536 | 6.578 | 6.697 | 7.131 | 7.186 | 7.151 | 8.172 | 8.432 | 8.359 | 9.577 | 9.709 | 9.654 | 10.465 | 10.312 | 10.387 |
| *E. coli* P[4] alone | 17 | 37 | GSGSGVLDGPYQPTTFKPPND | 7.32 | 2 | 15 | 5.742 | 5.884 | 6.001 | 6.781 | 6.778 | 6.886 | 7.436 | 7.526 | 7.389 | 8.665 | 8.854 | 8.767 | 10.291 | 10.212 | 10.161 | 10.838 | 10.792 | 10.903 |
| *E. coli* P[4] alone | 17 | 39 | GSGSGVLDGPYQPTTFKPPNDYW | 9.61 | 2 | 17 | 5.504 | 5.582 | 5.778 | 6.362 | 6.42 | 6.46 | 7.067 | 6.971 | 7.085 | 8.116 | 8.333 | 8.239 | 9.558 | 9.627 | 9.547 | 10.423 | 10.139 | 10.25 |
| *E. coli* P[4] alone | 24 | 37 | DGPYQPTTFKPPND | 6.14 | 2 | 8 | 2.453 | 2.57 | 2.556 | 2.743 | 2.739 | 2.774 | 3.228 | 3.227 | 3.198 | 4.308 | 4.435 | 4.398 | 5.677 | 5.722 | 5.685 | 6.357 | 6.304 | 6.363 |
| *E. coli* P[4] alone | 24 | 39 | DGPYQPTTFKPPNDYW | 9.19 | 2 | 10 | 2.366 | 2.347 | 2.448 | 2.584 | 2.599 | 2.651 | 3.107 | 3.077 | 3.079 | 4.122 | 4.231 | 4.192 | 5.353 | 5.329 | 5.407 | 6.101 | 6.093 | 6.044 |
| *E. coli* P[4] alone | 38 | 41 | YWLL | 11.1 | 1 | 2 | 0.04 | 0.035 | 0.031 | 0.036 | 0.034 | 0.035 | 0.033 | 0.032 | 0.027 | 0.036 | 0.033 | 0.029 | 0.028 | 0.031 | 0.027 | 0.03 | 0.025 | 0.034 |
| *E. coli* P[4] alone | 40 | 51 | LLISSNTNGVVY | 7.65 | 2 | 10 | 3.477 | 3.433 | 3.61 | 3.753 | 3.681 | 3.711 | 3.843 | 3.85 | 3.91 | 4.003 | 4.065 | 4.021 | 4.479 | 4.476 | 4.501 | 5.263 | 4.985 | 5.085 |
| *E. coli* P[4] alone | 40 | 59 | LLISSNTNGVVYESTNNNDF | 8.18 | 2 | 18 | 3.412 | 3.572 | 3.625 | 3.854 | 3.896 | 3.946 | 3.994 | 4.037 | 4.044 | 4.371 | 4.474 | 4.421 | 5.104 | 5.203 | 5.144 | 6.12 | 5.949 | 5.997 |
| *E. coli* P[4] alone | 41 | 51 | LISSNTNGVVY | 6.17 | 1 | 9 | 3.396 | 3.372 | 3.407 | 3.775 | 3.748 | 3.835 | 3.822 | 3.757 | 3.776 | 3.918 | 3.991 | 4.036 | 4.379 | 4.48 | 4.413 | 4.929 | 4.81 | 4.88 |
| *E. coli* P[4] alone | 42 | 59 | ISSNTNGVVYESTNNNDF | 6.82 | 2 | 16 | 3.546 | 3.641 | 3.704 | 3.882 | 3.962 | 3.983 | 4.072 | 4.108 | 4.082 | 4.379 | 4.547 | 4.497 | 5.129 | 5.227 | 5.144 | 5.849 | 5.727 | 5.786 |
| *E. coli* P[4] alone | 52 | 59 | ESTNNNDF | 4.28 | 1 | 6 | 0.187 | 0.217 | 0.183 | 0.207 | 0.241 | 0.197 | 0.319 | 0.323 | 0.323 | 0.63 | 0.644 | 0.613 | 1.136 | 1.112 | 1.114 | 1.626 | 1.581 | 1.623 |
| *E. coli* P[4] alone | 59 | 63 | FWTAV | 8.1 | 1 | 3 | 0.036 | 0.05 | 0.037 | 0.045 | 0.017 | 0.03 | 0.04 | 0.044 | 0.034 | 0.05 | 0.022 | 0.035 | 0.047 | 0.025 | 0.025 | 0.044 | 0.026 | 0.052 |
| *E. coli* P[4] alone | 59 | 64 | FWTAVI | 9.72 | 2 | 4 | 0.316 | 0.704 | 0.38 | 0.483 | 0.341 | 0.115 | 0.565 | 0.469 | 0.336 | 0.406 | 0.275 | 0.292 | 0.251 | 0.552 | 0.45 | 0.154 | 0.288 | 0.396 |
| *E. coli* P[4] alone | 60 | 63 | WTAV | 5.58 | 1 | 2 | 0.027 | 0.029 | 0.028 | 0.022 | 0.028 | 0.027 | 0.023 | 0.028 | 0.019 | 0.023 | 0.03 | 0.022 | 0.033 | 0.022 | 0.019 | 0.02 | 0.019 | 0.027 |
| *E. coli* P[4] alone | 60 | 64 | WTAVI | 7.81 | 1 | 3 | 0.046 | 0.049 | 0.048 | 0.043 | 0.037 | 0.048 | 0.037 | 0.038 | 0.034 | 0.037 | 0.035 | 0.031 | 0.043 | 0.034 | 0.03 | 0.041 | 0.04 | 0.033 |
| *E. coli* P[4] alone | 64 | 73 | IAVEPHVSQT | 4.1 | 2 | 7 | 1.479 | 1.502 | 1.547 | 1.942 | 1.914 | 1.949 | 2.262 | 2.255 | 2.269 | 2.409 | 2.475 | 2.438 | 2.875 | 2.865 | 2.862 | 3.387 | 3.336 | 3.386 |
| *E. coli* P[4] alone | 64 | 76 | IAVEPHVSQTNRQ | 3.65 | 2 | 10 | 2.601 | 2.667 | 2.748 | 3.405 | 3.421 | 3.474 | 3.96 | 3.976 | 3.955 | 4.269 | 4.373 | 4.281 | 5.119 | 5.156 | 5.102 | 5.877 | 5.739 | 5.884 |
| *E. coli* P[4] alone | 64 | 79 | IAVEPHVSQTNRQYIL | 7.05 | 3 | 13 | 2.421 | 2.555 | 2.577 | 3.444 | 3.549 | 3.549 | 4.17 | 4.276 | 4.228 | 4.535 | 4.666 | 4.595 | 5.368 | 5.361 | 5.301 | 6.008 | 5.894 | 6.01 |
| *E. coli* P[4] alone | 65 | 73 | AVEPHVSQT | 3.14 | 2 | 6 | 1.464 | 1.434 | 1.476 | 1.923 | 1.938 | 1.941 | 2.291 | 2.281 | 2.25 | 2.42 | 2.453 | 2.469 | 2.847 | 2.891 | 2.907 | 3.403 | 3.353 | 3.394 |
| *E. coli* P[4] alone | 65 | 76 | AVEPHVSQTNRQ | 2.89 | 3 | 9 | 2.638 | 2.709 | 2.817 | 3.489 | 3.535 | 3.565 | 4.043 | 4.05 | 4.07 | 4.354 | 4.476 | 4.388 | 5.258 | 5.29 | 5.218 | 6.082 | 5.916 | 6.051 |
| *E. coli* P[4] alone | 65 | 79 | AVEPHVSQTNRQYIL | 6.64 | 3 | 12 | 2.555 | 2.741 | 2.705 | 3.583 | 3.616 | 3.641 | 4.331 | 4.351 | 4.305 | 4.682 | 4.75 | 4.613 | 5.843 | 5.572 | 5.384 | 6.045 | 5.957 | 6.141 |
| *E. coli* P[4] alone | 74 | 79 | NRQYIL | 6.48 | 2 | 4 | 0.787 | 0.777 | 0.805 | 1.53 | 1.403 | 1.422 | 1.846 | 1.784 | 1.777 | 1.858 | 1.923 | 1.873 | 1.95 | 1.926 | 1.892 | 1.967 | 1.895 | 2.007 |
| *E. coli* P[4] alone | 80 | 86 | FGENKQF | 5.21 | 2 | 5 | 1.221 | 1.244 | 1.283 | 1.376 | 1.363 | 1.394 | 1.372 | 1.39 | 1.4 | 1.423 | 1.467 | 1.426 | 1.591 | 1.576 | 1.562 | 2.042 | 1.983 | 2.039 |
| *E. coli* P[4] alone | 80 | 97 | FGENKQFNVENNSDKWKF | 8.1 | 4 | 16 | 2.302 | 2.484 | 2.51 | 2.712 | 2.698 | 2.713 | 3.067 | 3.052 | 3.031 | 4.045 | 4.027 | 3.973 | 5.305 | 5.2 | 5.129 | 6.115 | 5.919 | 6.187 |
| *E. coli* P[4] alone | 84 | 97 | KQFNVENNSDKWKF | 7.49 | 4 | 12 | 1.067 | 1.217 | 1.255 | 1.402 | 1.421 | 1.387 | 1.711 | 1.636 | 1.566 | 2.588 | 2.461 | 2.387 | 3.845 | 4.163 | 4.057 | 4.61 | 4.794 | 4.853 |
| *E. coli* P[4] alone | 98 | 100 | FEM | 6.24 | 1 | 1 | 0.021 | 0.029 | 0.03 | 0.023 | 0.031 | 0.026 | 0.024 | 0.031 | 0.027 | 0.025 | 0.021 | 0.029 | 0.027 | 0.024 | 0.027 | 0.028 | 0.027 | 0.028 |
| *E. coli* P[4] alone | 98 | 109 | FEMFKGSSQGDF | 8.35 | 2 | 10 | 3.522 | 3.605 | 3.682 | 3.755 | 3.791 | 3.899 | 3.883 | 3.995 | 3.994 | 3.948 | 4.093 | 4.08 | 4.08 | 4.187 | 4.158 | 4.204 | 4.18 | 4.162 |
| *E. coli* P[4] alone | 98 | 121 | FEMFKGSSQGDFSNRRTLTSSNRL | 7.18 | 3 | 22 | 5.349 | 6.042 | 6.464 | 6.793 | 6.837 | 7.105 | 7.232 | 7.303 | 7.467 | 7.532 | 7.812 | 8.073 | 8.442 | 8.394 | 7.885 | 8.723 | 8.051 | 8.731 |
| *E. coli* P[4] alone | 101 | 109 | FKGSSQGDF | 4.88 | 2 | 7 | 3.768 | 3.869 | 3.946 | 4.279 | 4.214 | 4.235 | 4.412 | 4.394 | 4.391 | 4.433 | 4.543 | 4.486 | 4.588 | 4.567 | 4.553 | 4.669 | 4.572 | 4.705 |
| *E. coli* P[4] alone | 101 | 121 | FKGSSQGDFSNRRTLTSSNRL | 5.31 | 3 | 19 | 6.519 | 7.098 | 7.186 | 8.085 | 8.046 | 8.25 | 8.686 | 8.73 | 8.583 | 8.99 | 9.181 | 8.917 | 9.802 | 9.574 | 9.419 | 9.973 | 9.414 | 10.246 |
| *E. coli* P[4] alone | 101 | 123 | FKGSSQGDFSNRRTLTSSNRLVG | 5.49 | 4 | 21 | 6.074 | 6.612 | 6.762 | 7.74 | 7.521 | 7.827 | 8.305 | 8.25 | 8.185 | 8.63 | 8.558 | 8.532 | 9.54 | 9.107 | 9.063 | 9.774 | 9.158 | 10.052 |
| *E. coli* P[4] alone | 101 | 125 | FKGSSQGDFSNRRTLTSSNRLVGML | 7.76 | 4 | 23 | 6.059 | 6.51 | 6.669 | 7.127 | 7.363 | 7.45 | 7.814 | 7.996 | 7.748 | 8.305 | 8.153 | 8.233 | 8.4 | 8.616 | 8.58 | 9.312 | 9.182 | 9.052 |
| *E. coli* P[4] alone | 102 | 121 | KGSSQGDFSNRRTLTSSNRL | 4.92 | 4 | 18 | 6.014 | 6.408 | 6.47 | 7.473 | 7.46 | 7.686 | 8.036 | 7.967 | 7.961 | 8.465 | 8.569 | 8.319 | 9.194 | 8.736 | 8.834 | 9.289 | 8.617 | 9.644 |
| *E. coli* P[4] alone | 122 | 124 | VGM | 2.87 | 1 | 1 | -0.002 | 0.108 | 0.012 | 0.043 | 0.095 | -0.039 | 0.003 | -0.02 | 0.052 | 0.06 | -0.001 | 0.021 | -0.004 | -0.045 | -0.007 | 0.23 | 0.152 | 0.135 |
| *E. coli* P[4] alone | 122 | 134 | VGMLKYGGRVWTF | 9.52 | 3 | 11 | 0.959 | 0.98 | 1.011 | 1.318 | 1.227 | 1.093 | 1.31 | 1.184 | 1.112 | 1.377 | 1.29 | 1.191 | 1.693 | 1.501 | 1.428 | 2.64 | 1.982 | 2.615 |
| *E. coli* P[4] alone | 122 | 147 | VGMLKYGGRVWTFHGETPRATTDSSN | 7.37 | 4 | 23 | 1.447 | 1.948 | 2.229 | 2.731 | 2.696 | 2.55 | 3.451 | 3.148 | 2.949 | 3.709 | 3.332 | 3.23 | 4.306 | 4.26 | 4.68 | 7.251 | 5.726 | 7.767 |
| *E. coli* P[4] alone | 122 | 151 | VGMLKYGGRVWTFHGETPRATTDSSNTADL | 7.85 | 4 | 27 | 3.424 | 3.84 | 3.899 | 4.896 | 4.764 | 4.816 | 5.714 | 5.657 | 5.65 | 6.372 | 6.319 | 6.252 | 7.849 | 7.457 | 7.389 | 10.084 | 8.703 | 10.231 |
| *E. coli* P[4] alone | 122 | 153 | VGMLKYGGRVWTFHGETPRATTDSSNTADLNN | 7.48 | 4 | 29 | 5.388 | 5.575 | 5.55 | 6.667 | 6.44 | 6.484 | 7.486 | 7.3 | 7.393 | 8.272 | 8.162 | 8.136 | 9.567 | 9.413 | 9.402 | 12.434 | 10.807 | 12.519 |
| *E. coli* P[4] alone | 122 | 155 | VGMLKYGGRVWTFHGETPRATTDSSNTADLNNIS | 7.78 | 4 | 31 | 5.46 | 5.718 | 5.855 | 6.971 | 7.13 | 7.199 | 8.219 | 8.26 | 8.16 | 8.766 | 9.001 | 8.959 | 10.363 | 10.414 | 10.351 | 13.118 | 11.956 | 13.011 |
| *E. coli* P[4] alone | 124 | 134 | MLKYGGRVWTF | 9.2 | 3 | 9 | 0.897 | 0.999 | 1.041 | 1.368 | 1.164 | 1.053 | 1.268 | 1.209 | 1.073 | 1.401 | 1.261 | 1.142 | 1.624 | 1.418 | 1.444 | 2.413 | 1.911 | 2.456 |
| *E. coli* P[4] alone | 124 | 151 | MLKYGGRVWTFHGETPRATTDSSNTADL | 7.49 | 4 | 25 | 2.988 | 3.579 | 3.612 | 4.95 | 4.329 | 4.522 | 5.631 | 5.572 | 5.341 | 6.302 | 5.953 | 5.876 | 7.84 | 7.171 | 7.077 | 9.593 | 8.132 | 10.802 |
| *E. coli* P[4] alone | 125 | 134 | LKYGGRVWTF | 8.88 | 3 | 8 | 1.096 | 1.244 | 1.217 | 1.426 | 1.266 | 1.26 | 1.438 | 1.329 | 1.219 | 1.378 | 1.377 | 1.245 | 1.673 | 1.584 | 1.555 | 2.421 | 2.054 | 2.487 |
| *E. coli* P[4] alone | 125 | 151 | LKYGGRVWTFHGETPRATTDSSNTADL | 7.17 | 4 | 24 | 3.773 | 3.953 | 3.934 | 4.999 | 4.87 | 4.906 | 5.82 | 5.837 | 5.748 | 6.63 | 6.541 | 6.498 | 8.014 | 7.653 | 7.617 | 10.017 | 8.746 | 10.197 |
| *E. coli* P[4] alone | 125 | 153 | LKYGGRVWTFHGETPRATTDSSNTADLNN | 6.72 | 4 | 26 | 5.334 | 5.825 | 5.781 | 6.681 | 6.789 | 6.866 | 7.705 | 7.784 | 7.597 | 8.563 | 8.533 | 8.392 | 9.977 | 9.861 | 9.773 | 12.278 | 11.086 | 12.351 |
| *E. coli* P[4] alone | 125 | 155 | LKYGGRVWTFHGETPRATTDSSNTADLNNIS | 7.15 | 4 | 28 | 5.834 | 6.13 | 6.104 | 7.36 | 7.577 | 7.657 | 8.472 | 8.737 | 8.513 | 9.18 | 9.387 | 9.331 | 10.68 | 10.715 | 10.686 | 13.631 | 12.092 | 13.209 |
| *E. coli* P[4] alone | 135 | 151 | HGETPRATTDSSNTADL | 4.1 | 3 | 14 | 2.793 | 2.981 | 2.982 | 4.018 | 4.033 | 4.102 | 5.128 | 5.033 | 5.037 | 5.793 | 5.853 | 5.804 | 6.97 | 6.934 | 6.972 | 8.697 | 7.83 | 8.697 |
| *E. coli* P[4] alone | 135 | 155 | HGETPRATTDSSNTADLNNIS | 4.78 | 3 | 18 | 4.501 | 4.651 | 4.42 | 6.065 | 5.88 | 6.023 | 7.39 | 7.457 | 7.458 | 8.015 | 8.436 | 8.288 | 9.275 | 9.521 | 9.469 | 11.282 | 10.461 | 11.475 |
| *E. coli* P[4] alone | 154 | 162 | ISIIIHSEF | 8.4 | 2 | 7 | 0.145 | 0.16 | 0.143 | 0.202 | 0.21 | 0.208 | 0.438 | 0.435 | 0.449 | 0.969 | 1.025 | 0.98 | 1.464 | 1.509 | 1.494 | 2.257 | 2.261 | 2.23 |
| *E. coli* P[4] alone | 156 | 161 | IIIHSE | 3.79 | 2 | 4 | 0.091 | 0.12 | 0.089 | 0.175 | 0.189 | 0.179 | 0.362 | 0.355 | 0.381 | 0.848 | 0.895 | 0.881 | 1.227 | 1.285 | 1.247 | 1.637 | 1.597 | 1.622 |
| *E. coli* P[4] alone | 156 | 162 | IIIHSEF | 6.9 | 2 | 5 | 0.13 | 0.127 | 0.123 | 0.183 | 0.19 | 0.191 | 0.407 | 0.434 | 0.425 | 0.904 | 0.95 | 0.941 | 1.304 | 1.287 | 1.267 | 1.689 | 1.707 | 1.69 |
| *E. coli* P[4] alone | 157 | 162 | IIHSEF | 5.6 | 2 | 4 | 0.114 | 0.123 | 0.117 | 0.169 | 0.17 | 0.189 | 0.388 | 0.379 | 0.388 | 0.858 | 0.884 | 0.88 | 1.174 | 1.19 | 1.152 | 1.432 | 1.387 | 1.413 |
| *E. coli* P[4] alone | 163 | 170 | YIIPRSQE | 4.82 | 2 | 5 | 2.009 | 2.096 | 2.15 | 2.389 | 2.415 | 2.448 | 2.51 | 2.512 | 2.506 | 2.729 | 2.795 | 2.743 | 3.242 | 3.239 | 3.221 | 3.38 | 3.315 | 3.359 |
| *E. coli* P[4] alone | 163 | 173 | YIIPRSQESKC | 4.63 | 2 | 8 | 2.96 | 3.183 | 3.13 | 3.694 | 3.675 | 3.888 | 3.621 | 3.846 | 3.813 | 3.984 | 4.216 | 4.069 | 4.604 | 4.636 | 4.543 | 4.763 | 4.625 | 4.669 |
| *E. coli* P[4] alone | 163 | 175 | YIIPRSQESKCNE | 4.57 | 3 | 10 | 2.947 | 3.122 | 3.205 | 3.945 | 4.006 | 4.112 | 4.313 | 4.387 | 4.376 | 4.599 | 4.877 | 4.736 | 5.621 | 5.65 | 5.588 | 5.939 | 5.766 | 5.829 |
| *E. coli* P[4] alone | 174 | 181 | NEYINNGL | 6.32 | 1 | 6 | 0.324 | 0.325 | 0.361 | 0.612 | 0.586 | 0.591 | 1.01 | 1.069 | 0.99 | 1.421 | 1.398 | 1.387 | 2.597 | 2.562 | 2.498 | 4.053 | 2.701 | 4.005 |
| *E. coli* P[4] with Thimerosal | 1 | 3 | MQY | 4.26 | 1 | 1 | 1.055 | 1.073 | 0.991 | 1.021 | 1.057 | 1.053 | 1.014 | 1.027 | 1.058 | 1.065 | 1.062 | 1.075 | 1.054 | 1.034 | 1.118 | 1.045 | 1.035 | 1.057 |
| *E. coli* P[4] with Thimerosal | 1 | 6 | MQYIKA | 4.99 | 2 | 4 | 3.139 | 3.215 | 3.186 | 3.157 | 3.215 | 3.209 | 3.212 | 3.222 | 3.224 | 3.24 | 3.246 | 3.242 | 3.282 | 3.294 | 3.281 | 3.293 | 3.284 | 3.269 |
| *E. coli* P[4] with Thimerosal | 1 | 10 | MQYIKANSKF | 5.88 | 3 | 8 | 5.841 | 6.025 | 5.937 | 5.821 | 5.936 | 5.938 | 5.936 | 5.936 | 5.914 | 5.983 | 5.998 | 5.956 | 5.988 | 6.061 | 6.082 | 6.018 | 6.057 | 5.99 |
| *E. coli* P[4] with Thimerosal | 1 | 12 | MQYIKANSKFIG | 6.41 | 3 | 10 | 7.409 | 7.559 | 7.467 | 7.38 | 7.518 | 7.468 | 7.453 | 7.473 | 7.471 | 7.544 | 7.556 | 7.522 | 7.567 | 7.67 | 7.664 | 7.694 | 7.63 | 7.58 |
| *E. coli* P[4] with Thimerosal | 3 | 10 | YIKANSKF | 4.36 | 3 | 6 | 4.148 | 4.192 | 4.269 | 4.346 | 4.457 | 4.372 | 4.455 | 4.492 | 4.354 | 4.479 | 4.436 | 4.49 | 4.516 | 4.613 | 4.594 | 4.495 | 4.5 | 4.445 |
| *E. coli* P[4] with Thimerosal | 11 | 15 | IGITE | 4.47 | 1 | 3 | 2.803 | 2.889 | 2.876 | 2.816 | 2.85 | 2.879 | 2.888 | 2.821 | 2.896 | 2.899 | 2.91 | 2.894 | 2.923 | 2.947 | 2.97 | 2.932 | 2.948 | 2.928 |
| *E. coli* P[4] with Thimerosal | 11 | 23 | IGITELGSGSGVL | 8.57 | 2 | 11 | 8.167 | 8.42 | 8.297 | 8.238 | 8.409 | 8.34 | 8.482 | 8.379 | 8.387 | 8.478 | 8.511 | 8.506 | 8.542 | 8.595 | 8.646 | 8.584 | 8.571 | 8.534 |
| *E. coli* P[4] with Thimerosal | 11 | 37 | IGITELGSGSGVLDGPYQPTTFKPPND | 8.8 | 2 | 21 | 10.879 | 11.134 | 11.074 | 11.718 | 11.938 | 11.907 | 12.636 | 12.636 | 12.647 | 14.096 | 14.139 | 14.106 | 15.389 | 15.45 | 15.485 | 15.844 | 15.871 | 15.855 |
| *E. coli* P[4] with Thimerosal | 13 | 23 | ITELGSGSGVL | 7.1 | 1 | 9 | 6.064 | 6.244 | 6.175 | 6.143 | 6.206 | 6.2 | 6.199 | 6.177 | 6.32 | 6.325 | 6.428 | 6.271 | 6.425 | 6.472 | 6.515 | 6.414 | 6.386 | 6.385 |
| *E. coli* P[4] with Thimerosal | 13 | 37 | ITELGSGSGVLDGPYQPTTFKPPND | 8.07 | 2 | 19 | 8.955 | 9.282 | 9.126 | 9.914 | 10.051 | 9.98 | 10.833 | 10.781 | 10.764 | 12.23 | 12.257 | 12.157 | 13.6 | 13.544 | 13.67 | 14.076 | 14.017 | 14.001 |
| *E. coli* P[4] with Thimerosal | 16 | 37 | LGSGSGVLDGPYQPTTFKPPND | 7.6 | 2 | 16 | 6.398 | 6.636 | 6.565 | 7.237 | 7.345 | 7.358 | 8.088 | 8.113 | 8.078 | 9.53 | 9.522 | 9.479 | 10.843 | 10.87 | 10.926 | 11.326 | 11.381 | 11.313 |
| *E. coli* P[4] with Thimerosal | 16 | 38 | LGSGSGVLDGPYQPTTFKPPNDY | 8.24 | 2 | 17 | 6.16 | 6.385 | 6.368 | 6.977 | 7.134 | 7.148 | 7.833 | 7.834 | 7.838 | 9.266 | 9.264 | 9.222 | 10.532 | 10.621 | 10.689 | 11.222 | 11.229 | 11.166 |
| *E. coli* P[4] with Thimerosal | 16 | 39 | LGSGSGVLDGPYQPTTFKPPNDYW | 9.8 | 3 | 18 | 5.827 | 6.025 | 6 | 6.591 | 6.728 | 6.757 | 7.387 | 7.412 | 7.357 | 8.656 | 8.732 | 8.674 | 9.934 | 9.984 | 10.102 | 10.665 | 10.738 | 10.637 |
| *E. coli* P[4] with Thimerosal | 17 | 37 | GSGSGVLDGPYQPTTFKPPND | 7.32 | 2 | 15 | 5.946 | 6.088 | 6.045 | 6.719 | 6.892 | 6.895 | 7.651 | 7.614 | 7.64 | 9.08 | 9.038 | 9.089 | 10.427 | 10.421 | 10.455 | 10.914 | 10.93 | 10.824 |
| *E. coli* P[4] with Thimerosal | 17 | 39 | GSGSGVLDGPYQPTTFKPPNDYW | 9.61 | 2 | 17 | 5.58 | 5.851 | 5.776 | 6.464 | 6.538 | 6.553 | 7.187 | 7.261 | 7.22 | 8.532 | 8.609 | 8.535 | 9.842 | 9.838 | 9.909 | 10.55 | 10.546 | 10.538 |
| *E. coli* P[4] with Thimerosal | 24 | 37 | DGPYQPTTFKPPND | 6.14 | 2 | 8 | 2.49 | 2.545 | 2.55 | 2.73 | 2.797 | 2.772 | 3.394 | 3.365 | 3.381 | 4.679 | 4.678 | 4.671 | 5.874 | 5.886 | 5.88 | 6.414 | 6.42 | 6.445 |
| *E. coli* P[4] with Thimerosal | 24 | 39 | DGPYQPTTFKPPNDYW | 9.19 | 2 | 10 | 2.386 | 2.45 | 2.441 | 2.642 | 2.655 | 2.668 | 3.249 | 3.196 | 3.243 | 4.443 | 4.457 | 4.465 | 5.632 | 5.615 | 5.677 | 6.361 | 6.344 | 6.338 |
| *E. coli* P[4] with Thimerosal | 38 | 41 | YWLL | 11.1 | 1 | 2 | 0.032 | 0.034 | 0.041 | 0.031 | 0.031 | 0.03 | 0.029 | 0.028 | 0.026 | 0.026 | 0.032 | 0.031 | 0.035 | 0.035 | 0.036 | 0.059 | 0.059 | 0.059 |
| *E. coli* P[4] with Thimerosal | 40 | 51 | LLISSNTNGVVY | 7.65 | 2 | 10 | 3.462 | 3.486 | 3.492 | 3.769 | 3.805 | 3.78 | 3.922 | 3.942 | 3.87 | 4.227 | 4.143 | 4.099 | 4.709 | 4.765 | 4.78 | 5.252 | 5.318 | 5.517 |
| *E. coli* P[4] with Thimerosal | 40 | 59 | LLISSNTNGVVYESTNNNDF | 8.18 | 2 | 18 | 3.609 | 3.65 | 3.669 | 3.862 | 3.933 | 3.889 | 4.082 | 4.091 | 4.089 | 4.581 | 4.568 | 4.585 | 5.436 | 5.47 | 5.514 | 6.313 | 6.431 | 6.352 |
| *E. coli* P[4] with Thimerosal | 41 | 51 | LISSNTNGVVY | 6.17 | 1 | 9 | 3.339 | 3.433 | 3.466 | 3.629 | 3.75 | 3.757 | 3.835 | 3.818 | 3.839 | 4.027 | 4.054 | 4.009 | 4.614 | 4.623 | 4.616 | 5.071 | 5.061 | 5.038 |
| *E. coli* P[4] with Thimerosal | 42 | 59 | ISSNTNGVVYESTNNNDF | 6.82 | 2 | 16 | 3.608 | 3.735 | 3.687 | 3.926 | 4.017 | 3.96 | 4.157 | 4.174 | 4.146 | 4.592 | 4.593 | 4.563 | 5.301 | 5.344 | 5.377 | 5.939 | 5.972 | 5.9 |
| *E. coli* P[4] with Thimerosal | 52 | 59 | ESTNNNDF | 4.28 | 1 | 6 | 0.188 | 0.252 | 0.192 | 0.214 | 0.246 | 0.237 | 0.327 | 0.335 | 0.299 | 0.705 | 0.67 | 0.669 | 1.181 | 1.209 | 1.233 | 1.739 | 1.75 | 1.719 |
| *E. coli* P[4] with Thimerosal | 59 | 63 | FWTAV | 8.1 | 1 | 3 | 0.041 | 0.025 | 0.014 | 0.033 | 0.036 | 0.045 | 0.038 | 0.029 | 0.024 | 0.043 | 0.03 | 0.043 | 0.027 | 0.036 | 0.036 | 0.056 | 0.046 | 0.049 |
| *E. coli* P[4] with Thimerosal | 59 | 64 | FWTAVI | 9.72 | 2 | 4 | 0.309 | 0.381 | 0.514 | 0.295 | 0.33 | 0.49 | 0.156 | 0.33 | 0.219 | 0.267 | 0.252 | 0.097 | -0.009 | 0.252 | 0.405 | 0.365 | 0.104 | 0.272 |
| *E. coli* P[4] with Thimerosal | 60 | 63 | WTAV | 5.58 | 1 | 2 | 0.026 | 0.029 | 0.028 | 0.026 | 0.022 | 0.023 | 0.022 | 0.021 | 0.019 | 0.024 | 0.022 | 0.023 | 0.026 | 0.025 | 0.024 | 0.032 | 0.032 | 0.03 |
| *E. coli* P[4] with Thimerosal | 60 | 64 | WTAVI | 7.81 | 1 | 3 | 0.048 | 0.043 | 0.022 | 0.043 | 0.043 | 0.043 | 0.042 | 0.04 | 0.032 | 0.034 | 0.032 | 0.037 | 0.031 | 0.04 | 0.034 | 0.04 | 0.041 | 0.042 |
| *E. coli* P[4] with Thimerosal | 64 | 73 | IAVEPHVSQT | 4.1 | 2 | 7 | 1.47 | 1.527 | 1.543 | 1.907 | 1.942 | 1.933 | 2.271 | 2.281 | 2.263 | 2.464 | 2.446 | 2.432 | 2.861 | 2.883 | 2.902 | 3.366 | 3.381 | 3.342 |
| *E. coli* P[4] with Thimerosal | 64 | 76 | IAVEPHVSQTNRQ | 3.65 | 2 | 10 | 2.619 | 2.761 | 2.751 | 3.404 | 3.431 | 3.449 | 3.977 | 4.012 | 3.963 | 4.355 | 4.368 | 4.297 | 5.158 | 5.178 | 5.252 | 5.78 | 5.84 | 5.708 |
| *E. coli* P[4] with Thimerosal | 64 | 79 | IAVEPHVSQTNRQYIL | 7.05 | 3 | 13 | 2.546 | 2.636 | 2.619 | 3.479 | 3.536 | 3.543 | 4.29 | 4.262 | 4.31 | 4.677 | 4.638 | 4.634 | 5.428 | 5.5 | 5.472 | 5.923 | 6.012 | 5.882 |
| *E. coli* P[4] with Thimerosal | 65 | 73 | AVEPHVSQT | 3.14 | 2 | 6 | 1.457 | 1.482 | 1.511 | 1.926 | 1.915 | 1.924 | 2.286 | 2.257 | 2.253 | 2.439 | 2.46 | 2.43 | 2.877 | 2.877 | 2.861 | 3.357 | 3.381 | 3.356 |
| *E. coli* P[4] with Thimerosal | 65 | 76 | AVEPHVSQTNRQ | 2.89 | 3 | 9 | 2.683 | 2.818 | 2.835 | 3.481 | 3.505 | 3.53 | 4.066 | 4.098 | 4.055 | 4.431 | 4.5 | 4.416 | 5.298 | 5.281 | 5.352 | 5.946 | 6.005 | 5.894 |
| *E. coli* P[4] with Thimerosal | 65 | 79 | AVEPHVSQTNRQYIL | 6.64 | 3 | 12 | 2.689 | 2.82 | 2.752 | 3.612 | 3.612 | 3.661 | 4.346 | 4.363 | 4.353 | 4.768 | 4.718 | 4.743 | 5.48 | 5.569 | 5.562 | 5.985 | 6.038 | 5.885 |
| *E. coli* P[4] with Thimerosal | 74 | 79 | NRQYIL | 6.48 | 2 | 4 | 0.782 | 0.867 | 0.857 | 1.554 | 1.412 | 1.497 | 1.816 | 1.855 | 1.822 | 1.904 | 1.898 | 1.936 | 1.886 | 1.915 | 1.994 | 1.925 | 1.94 | 1.947 |
| *E. coli* P[4] with Thimerosal | 80 | 86 | FGENKQF | 5.21 | 2 | 5 | 1.236 | 1.269 | 1.301 | 1.364 | 1.374 | 1.393 | 1.394 | 1.412 | 1.393 | 1.459 | 1.444 | 1.43 | 1.586 | 1.612 | 1.618 | 2.034 | 2.077 | 2.004 |
| *E. coli* P[4] with Thimerosal | 80 | 97 | FGENKQFNVENNSDKWKF | 8.1 | 4 | 16 | 2.485 | 2.552 | 2.484 | 2.638 | 2.701 | 2.653 | 3.087 | 3.034 | 2.97 | 4.061 | 3.995 | 3.996 | 5.233 | 5.247 | 5.238 | 6.155 | 6.159 | 6.003 |

| | | | | | | | | | | | | | | | | | | | | | | | | |
|---|---|---|---|---|---|---|---|---|---|---|---|---|---|---|---|---|---|---|---|---|---|---|---|---|
| *E. coli* P[4] with Thimerosal | 84 | 97 | KQFNVENNSDKWKF | 7.49 | 4 | 12 | 1.225 | 1.252 | 1.189 | 1.278 | 1.331 | 1.371 | 1.576 | 1.617 | 1.658 | 2.433 | 2.362 | 2.579 | 3.877 | 4.086 | 4.081 | 4.617 | 4.658 | 4.877 |
| *E. coli* P[4] with Thimerosal | 98 | 100 | FEM | 6.24 | 1 | 1 | 0.035 | 0.031 | 0.023 | 0.026 | 0.028 | 0.027 | 0.023 | 0.02 | 0.025 | 0.02 | 0.022 | 0.024 | 0.026 | 0.03 | 0.024 | 0.036 | 0.037 | 0.035 |
| *E. coli* P[4] with Thimerosal | 98 | 109 | FEMFKGSSQGDF | 8.35 | 2 | 10 | 3.622 | 3.69 | 3.741 | 3.833 | 3.905 | 3.932 | 4.066 | 4.072 | 4.034 | 4.17 | 4.097 | 4.095 | 4.206 | 4.243 | 4.209 | 4.257 | 4.338 | 4.263 |
| *E. coli* P[4] with Thimerosal | 98 | 121 | FEMFKGSSQGDFSNRRTLTSSNRL | 7.18 | 3 | 22 | 6.423 | 6.684 | 6.061 | 7.168 | 7.339 | 7.217 | 8.213 | 7.94 | 7.83 | 8.623 | 8.073 | 8.15 | 8.846 | 8.8 | 8.827 | 10.015 | 10.068 | 9.776 |
| *E. coli* P[4] with Thimerosal | 101 | 109 | FKGSSQGDF | 4.88 | 2 | 7 | 3.895 | 4.035 | 3.969 | 4.176 | 4.268 | 4.242 | 4.417 | 4.429 | 4.397 | 4.513 | 4.489 | 4.476 | 4.564 | 4.604 | 4.6 | 4.567 | 4.598 | 4.508 |
| *E. coli* P[4] with Thimerosal | 101 | 121 | FKGSSQGDFSNRRTLTSSNRL | 5.31 | 3 | 19 | 7.218 | 7.551 | 7.289 | 8.022 | 8.18 | 8.212 | 8.77 | 8.704 | 8.55 | 9.419 | 9.204 | 9.176 | 9.987 | 9.961 | 10.002 | 10.961 | 11.036 | 10.695 |
| *E. coli* P[4] with Thimerosal | 101 | 123 | FKGSSQGDFSNRRTLTSSNRLVG | 5.49 | 4 | 21 | 6.745 | 7.058 | 6.991 | 7.504 | 7.745 | 7.749 | 8.584 | 8.234 | 8.269 | 9.1 | 8.862 | 8.831 | 9.637 | 9.691 | 9.779 | 11.252 | 11.215 | 10.93 |
| *E. coli* P[4] with Thimerosal | 101 | 125 | FKGSSQGDFSNRRTLTSSNRLVGML | 7.76 | 4 | 23 | 6.368 | 6.543 | 6.387 | 7.1 | 7.53 | 7.422 | 8.049 | 7.977 | 7.87 | 8.872 | 8.466 | 8.53 | 9.225 | 9.277 | 9.291 | 11.103 | 11.497 | 11.109 |
| *E. coli* P[4] with Thimerosal | 102 | 121 | KGSSQGDFSNRRTLTSSNRL | 4.92 | 4 | 18 | 6.559 | 6.896 | 6.636 | 7.182 | 7.711 | 7.85 | 8.167 | 7.97 | 8.177 | 8.712 | 8.462 | 8.541 | 9.35 | 9.496 | 9.354 | 10.395 | 10.404 | 9.997 |
| *E. coli* P[4] with Thimerosal | 122 | 124 | VGM | 2.87 | 1 | 1 | 0.106 | -0.021 | 0.013 | -0.019 | 0.036 | -0.036 | -0.038 | 0.035 | 0.023 | 0.013 | 0.057 | 0.079 | 0.077 | 0.109 | 0.04 | 0.455 | 0.479 | 0.495 |
| *E. coli* P[4] with Thimerosal | 122 | 134 | VGMLKYGGRVWTF | 9.52 | 3 | 11 | 1.027 | 1.05 | 1.011 | 1.15 | 1.153 | 1.112 | 1.22 | 1.193 | 1.198 | 1.41 | 1.414 | 1.372 | 2.22 | 2.175 | 2.199 | 4.134 | 4.348 | 4.227 |
| *E. coli* P[4] with Thimerosal | 122 | 147 | VGMLKYGGRVWTFHGETPRATTDSSN | 7.37 | 4 | 23 | 2.386 | 2.402 | 2.236 | 2.856 | 3.289 | 2.508 | 3.495 | 2.969 | 3.279 | 4.596 | 4.719 | 4.221 | 7.106 | 7.794 | 6.97 | 9.247 | 9.78 | 9.221 |
| *E. coli* P[4] with Thimerosal | 122 | 151 | VGMLKYGGRVWTFHGETPRATTDSSNTADL | 7.85 | 4 | 27 | 3.911 | 4.009 | 3.937 | 4.84 | 4.969 | 4.922 | 5.987 | 6.002 | 5.94 | 7.32 | 7.293 | 7.281 | 9.683 | 9.802 | 9.794 | 12.623 | 12.67 | 12.508 |
| *E. coli* P[4] with Thimerosal | 122 | 153 | VGMLKYGGRVWTFHGETPRATTDSSNTADLNN | 7.48 | 4 | 29 | 5.737 | 5.786 | 5.628 | 6.655 | 6.774 | 6.717 | 8.112 | 7.822 | 7.997 | 9.22 | 9.146 | 9.686 | 12.53 | 12.438 | 12.331 | 15.119 | 15.014 | 15.047 |
| *E. coli* P[4] with Thimerosal | 122 | 155 | VGMLKYGGRVWTFHGETPRATTDSSNTADLNNIS | 7.78 | 4 | 31 | 5.829 | 6.064 | 5.926 | 7.352 | 7.395 | 7.461 | 8.761 | 8.685 | 8.737 | 10.144 | 10.203 | 10.151 | 12.908 | 12.98 | 13.092 | 16.369 | 16.521 | 16.146 |
| *E. coli* P[4] with Thimerosal | 124 | 134 | MLKYGGRVWTF | 9.2 | 3 | 9 | 1.013 | 1.03 | 1.021 | 1.091 | 1.121 | 1.081 | 1.203 | 1.147 | 1.165 | 1.378 | 1.339 | 1.349 | 2.065 | 1.993 | 2.035 | 3.637 | 3.647 | 3.533 |
| *E. coli* P[4] with Thimerosal | 124 | 151 | MLKYGGRVWTFHGETPRATTDSSNTADL | 7.49 | 4 | 25 | 3.665 | 3.817 | 3.642 | 4.484 | 4.66 | 4.591 | 5.68 | 5.597 | 5.486 | 6.889 | 6.754 | 6.762 | 8.935 | 8.965 | 9.019 | 11.833 | 11.442 | 11.071 |
| *E. coli* P[4] with Thimerosal | 125 | 134 | LKYGGRVWTF | 8.88 | 3 | 8 | 1.233 | 1.222 | 1.168 | 1.238 | 1.247 | 1.208 | 1.298 | 1.25 | 1.237 | 1.482 | 1.39 | 1.43 | 2.245 | 2.182 | 2.052 | 3.755 | 3.612 | 3.593 |
| *E. coli* P[4] with Thimerosal | 125 | 151 | LKYGGRVWTFHGETPRATTDSSNTADL | 7.17 | 4 | 24 | 4.007 | 4.104 | 4.051 | 4.929 | 5.085 | 5.039 | 6.219 | 6.049 | 6.037 | 7.491 | 7.388 | 7.401 | 9.681 | 9.711 | 9.767 | 12.301 | 12.142 | 11.904 |
| *E. coli* P[4] with Thimerosal | 125 | 153 | LKYGGRVWTFHGETPRATTDSSNTADLNN | 6.72 | 4 | 26 | 5.763 | 6.029 | 5.827 | 6.826 | 6.957 | 6.878 | 8.112 | 7.997 | 8.084 | 9.639 | 9.494 | 9.508 | 12.021 | 12.207 | 12.18 | 14.951 | 14.968 | 14.531 |
| *E. coli* P[4] with Thimerosal | 125 | 155 | LKYGGRVWTFHGETPRATTDSSNTADLNNIS | 7.15 | 4 | 28 | 6.128 | 6.445 | 6.215 | 7.689 | 7.822 | 7.853 | 9.047 | 9.138 | 9.16 | 10.367 | 10.564 | 10.712 | 13.211 | 13.355 | 13.362 | 16.075 | 16.254 | 15.966 |
| *E. coli* P[4] with Thimerosal | 135 | 151 | HGETPRATTDSSNTADL | 4.1 | 3 | 14 | 3.026 | 3.177 | 3.121 | 4.149 | 4.208 | 4.324 | 5.521 | 5.46 | 5.413 | 6.684 | 6.688 | 6.668 | 8.556 | 8.325 | 8.726 | 9.802 | 9.805 | 9.645 |
| *E. coli* P[4] with Thimerosal | 135 | 155 | HGETPRATTDSSNTADLNNIS | 4.78 | 3 | 18 | 4.783 | 4.908 | 4.754 | 6.273 | 6.357 | 6.284 | 7.858 | 7.78 | 7.793 | 9.223 | 8.915 | 9.083 | 11.04 | 11.096 | 11.113 | 12.397 | 12.507 | 12.324 |
| *E. coli* P[4] with Thimerosal | 154 | 162 | ISIIIHSEF | 8.4 | 2 | 7 | 0.157 | 0.149 | 0.127 | 0.205 | 0.227 | 0.201 | 0.477 | 0.487 | 0.481 | 1.059 | 1.073 | 1.058 | 1.59 | 1.597 | 1.62 | 2.396 | 2.439 | 2.368 |
| *E. coli* P[4] with Thimerosal | 156 | 161 | IIIHSE | 3.79 | 2 | 4 | 0.111 | 0.087 | 0.125 | 0.175 | 0.186 | 0.218 | 0.387 | 0.435 | 0.402 | 0.936 | 0.916 | 0.923 | 1.23 | 1.288 | 1.293 | 1.604 | 1.67 | 1.577 |
| *E. coli* P[4] with Thimerosal | 156 | 162 | IIIHSEF | 6.9 | 2 | 5 | 0.127 | 0.126 | 0.127 | 0.198 | 0.196 | 0.195 | 0.441 | 0.454 | 0.448 | 0.979 | 0.98 | 0.968 | 1.303 | 1.334 | 1.338 | 1.729 | 1.733 | 1.66 |
| *E. coli* P[4] with Thimerosal | 157 | 162 | IIHSEF | 5.6 | 2 | 4 | 0.133 | 0.102 | 0.131 | 0.195 | 0.2 | 0.192 | 0.426 | 0.441 | 0.435 | 0.932 | 0.925 | 0.919 | 1.166 | 1.237 | 1.226 | 1.401 | 1.477 | 1.377 |
| *E. coli* P[4] with Thimerosal | 163 | 170 | YIIPRSQE | 4.82 | 2 | 5 | 2.185 | 2.316 | 2.311 | 2.5 | 2.568 | 2.554 | 2.796 | 2.869 | 2.834 | 3.183 | 3.242 | 3.215 | 3.291 | 3.355 | 3.4 | 3.322 | 3.39 | 3.357 |
| *E. coli* P[4] with Thimerosal | 163 | 173 | YIIPRSQESKC | 4.63 | 2 | 8 | 3.51 | 3.493 | 3.595 | 3.963 | 3.906 | 3.766 | 4.009 | 4.03 | 4.075 | 4.502 | 4.713 | 4.53 | 4.639 | 4.63 | 4.777 | 4.558 | 4.746 | 4.639 |
| *E. coli* P[4] with Thimerosal | 163 | 175 | YIIPRSQESKCNE | 4.57 | 3 | 10 | 3.746 | 3.994 | 3.983 | 4.573 | 4.76 | 4.8 | 5.151 | 5.317 | 5.208 | 5.625 | 5.739 | 5.627 | 5.695 | 5.895 | 5.929 | 5.775 | 5.917 | 5.777 |
| *E. coli* P[4] with Thimerosal | 174 | 181 | NEYINNGL | 6.32 | 1 | 6 | 0.57 | 0.667 | 0.759 | 1.187 | 1.381 | 1.409 | 2.497 | 2.455 | 2.587 | 3.787 | 3.822 | 3.838 | 4.476 | 4.518 | 4.544 | 4.585 | 4.591 | 4.664 |
| *E. coli* P[4] with 13A1 | 1 | 3 | MQY | 4.26 | 1 | 1 | 1.044 | 1.025 | 1.019 | 1.05 | 1.031 | 1.07 | 1.028 | 1.022 | 1.075 | 1.054 | 1.062 | 1.052 | 1.076 | 1.058 | 1.039 | 1.044 | 1.067 | 1.041 |
| *E. coli* P[4] with 13A1 | 1 | 6 | MQYIKA | 4.99 | 2 | 4 | 3.139 | 3.321 | 3.249 | 3.312 | 3.186 | 3.25 | 3.273 | 3.253 | 3.259 | 3.281 | 3.287 | 3.252 | 3.333 | 3.317 | 3.341 | 3.3 | 3.31 | 3.284 |
| *E. coli* P[4] with 13A1 | 1 | 10 | MQYIKANSKF | 5.88 | 3 | 8 | 5.59 | 5.931 | 5.801 | 5.903 | 5.651 | 5.812 | 5.849 | 5.754 | 5.823 | 5.848 | 5.852 | 5.79 | 5.909 | 5.914 | 5.877 | 5.864 | 5.918 | 5.795 |
| *E. coli* P[4] with 13A1 | 1 | 12 | MQYIKANSKFIG | 6.41 | 3 | 10 | 7.071 | 7.438 | 7.264 | 7.371 | 7.118 | 7.249 | 7.315 | 7.212 | 7.263 | 7.331 | 7.341 | 7.279 | 7.406 | 7.372 | 7.362 | 7.364 | 7.413 | 7.271 |
| *E. coli* P[4] with 13A1 | 3 | 10 | YIKANSKF | 4.36 | 3 | 6 | 4.081 | 4.486 | 4.338 | 4.57 | 4.411 | 4.481 | 4.443 | 4.378 | 4.528 | 4.514 | 4.493 | 4.47 | 4.52 | 4.53 | 4.564 | 4.491 | 4.536 | 4.449 |
| *E. coli* P[4] with 13A1 | 11 | 15 | IGITE | 4.47 | 1 | 3 | 2.727 | 2.91 | 2.857 | 2.889 | 2.771 | 2.849 | 2.866 | 2.842 | 2.867 | 2.892 | 2.894 | 2.855 | 2.932 | 2.912 | 2.9 | 2.898 | 2.919 | 2.877 |
| *E. coli* P[4] with 13A1 | 11 | 23 | IGITELGSGSGVL | 8.57 | 2 | 11 | 7.268 | 7.739 | 7.447 | 7.757 | 7.436 | 7.749 | 7.731 | 7.731 | 7.757 | 7.887 | 7.838 | 7.745 | 8.066 | 8.153 | 8.135 | 8.425 | 8.535 | 8.327 |
| *E. coli* P[4] with 13A1 | 11 | 37 | IGITELGSGSGVLDGPYQPTTFKPPND | 8.8 | 2 | 21 | 9.386 | 10 | 9.72 | 10.3 | 9.865 | 10.088 | 10.651 | 10.549 | 10.579 | 11.421 | 11.383 | 11.297 | 12.634 | 12.61 | 12.562 | 13.872 | 13.867 | 13.678 |
| *E. coli* P[4] with 13A1 | 13 | 23 | ITELGSGSGVL | 7.1 | 1 | 9 | 5.206 | 5.758 | 5.597 | 5.673 | 5.553 | 5.737 | 5.517 | 5.332 | 5.563 | 5.505 | 5.686 | 5.698 | 5.632 | 5.902 | 5.95 | 6.104 | 6.253 | 6.128 |
| *E. coli* P[4] with 13A1 | 13 | 37 | ITELGSGSGVLDGPYQPTTFKPPND | 8.07 | 2 | 19 | 7.62 | 8.029 | 7.907 | 8.337 | 8.045 | 8.224 | 8.792 | 8.655 | 8.723 | 9.46 | 9.476 | 9.389 | 10.689 | 10.725 | 10.658 | 11.951 | 12.044 | 11.81 |
| *E. coli* P[4] with 13A1 | 16 | 37 | LGSGSGVLDGPYQPTTFKPPND | 7.6 | 2 | 16 | 4.993 | 5.296 | 5.151 | 5.616 | 5.403 | 5.516 | 6.044 | 5.909 | 5.992 | 6.793 | 6.749 | 6.661 | 7.946 | 7.921 | 7.937 | 9.175 | 9.226 | 9.107 |
| *E. coli* P[4] with 13A1 | 16 | 38 | LGSGSGVLDGPYQPTTFKPPNDY | 8.24 | 2 | 17 | 4.43 | 5.444 | 5.351 | 5.898 | 5.516 | 5.779 | 6.239 | 6.055 | 6.193 | 5.34 | 5.987 | 5.873 | 6.508 | 6.41 | 6.383 | 7.751 | 7.813 | 7.556 |
| *E. coli* P[4] with 13A1 | 16 | 39 | LGSGSGVLDGPYQPTTFKPPNDYW | 9.8 | 3 | 18 | 4.572 | 4.877 | 4.724 | 5.175 | 4.94 | 5.048 | 5.625 | 5.454 | 5.509 | 6.214 | 6.199 | 6.102 | 7.255 | 7.222 | 7.181 | 8.367 | 8.396 | 8.222 |
| *E. coli* P[4] with 13A1 | 17 | 37 | GSGSGVLDGPYQPTTFKPPND | 7.32 | 2 | 15 | 4.303 | 4.604 | 4.417 | 4.933 | 4.675 | 4.862 | 5.354 | 5.212 | 5.329 | 6.004 | 6.045 | 6.004 | 7.291 | 7.232 | 7.18 | 8.61 | 8.642 | 8.531 |
| *E. coli* P[4] with 13A1 | 17 | 39 | GSGSGVLDGPYQPTTFKPPNDYW | 9.61 | 2 | 17 | 4.443 | 4.627 | 4.475 | 4.97 | 4.8 | 4.85 | 5.309 | 5.271 | 5.376 | 6.076 | 6.077 | 5.952 | 7.123 | 7.089 | 7.134 | 8.237 | 8.223 | 8.201 |
| *E. coli* P[4] with 13A1 | 24 | 37 | DGPYQPTTFKPPND | 6.14 | 2 | 8 | 2.37 | 2.516 | 2.475 | 2.592 | 2.556 | 2.564 | 2.821 | 2.787 | 2.778 | 3.461 | 3.482 | 3.417 | 4.332 | 4.325 | 4.257 | 5.002 | 5.06 | 4.931 |
| *E. coli* P[4] with 13A1 | 24 | 39 | DGPYQPTTFKPPNDYW | 9.19 | 2 | 10 | 2.254 | 2.399 | 2.316 | 2.482 | 2.366 | 2.423 | 2.683 | 2.694 | 2.651 | 3.328 | 3.315 | 3.268 | 4.066 | 4.062 | 4.071 | 4.685 | 4.69 | 4.679 |
| *E. coli* P[4] with 13A1 | 38 | 41 | YWLL | 11.1 | 1 | 2 | 0.03 | 0.026 | 0.026 | 0.024 | 0.032 | 0.025 | 0.028 | 0.023 | 0.025 | 0.023 | 0.027 | 0.025 | 0.023 | 0.024 | 0.026 | 0.025 | 0.021 | 0.02 |
| *E. coli* P[4] with 13A1 | 40 | 51 | LLISSNTNGVVY | 7.65 | 2 | 10 | 1.844 | 2.03 | 1.913 | 2.73 | 2.642 | 2.685 | 3.519 | 3.523 | 3.47 | 3.768 | 3.749 | 3.666 | 3.897 | 3.815 | 3.777 | 3.885 | 3.841 | 3.809 |
| *E. coli* P[4] with 13A1 | 40 | 59 | LLISSNTNGVVYESTNNNDF | 8.18 | 2 | 18 | 2.182 | 2.318 | 2.239 | 2.986 | 2.931 | 2.93 | 3.838 | 3.743 | 3.791 | 4.172 | 4.16 | 4.132 | 4.544 | 4.557 | 4.508 | 4.905 | 4.896 | 4.768 |
| *E. coli* P[4] with 13A1 | 41 | 51 | LISSNTNGVVY | 6.17 | 1 | 9 | 2.096 | 2.118 | 2.045 | 2.818 | 2.706 | 2.721 | 3.648 | 3.561 | 3.578 | 3.798 | 3.809 | 3.786 | 3.952 | 3.94 | 3.891 | 3.871 | 3.879 | 3.823 |
| *E. coli* P[4] with 13A1 | 42 | 59 | ISSNTNGVVYESTNNNDF | 6.82 | 2 | 16 | 2.098 | 2.241 | 2.155 | 2.941 | 2.843 | 2.86 | 3.762 | 3.7 | 3.696 | 4.198 | 4.199 | 4.142 | 4.74 | 4.719 | 4.702 | 5.205 | 5.214 | 5.14 |
| *E. coli* P[4] with 13A1 | 52 | 59 | ESTNNNDF | 4.28 | 1 | 6 | 0.181 | 0.174 | 0.169 | 0.189 | 0.207 | 0.202 | 0.31 | 0.317 | 0.309 | 0.65 | 0.638 | 0.635 | 1.116 | 1.102 | 1.131 | 1.568 | 1.632 | 1.553 |
| *E. coli* P[4] with 13A1 | 59 | 63 | FWTAV | 8.1 | 1 | 3 | 0.097 | 0.078 | 0.099 | 0.062 | 0.092 | 0.064 | 0.075 | 0.057 | 0.081 | 0.043 | 0.063 | 0.052 | 0.03 | 0.024 | 0.018 | 0.013 | 0.008 | 0.007 |
| *E. coli* P[4] with 13A1 | 59 | 64 | FWTAVI | 9.72 | 2 | 4 | 0.415 | 0.108 | 0.427 | 0.036 | 0.573 | 0.091 | 0.091 | 0.204 | -0.011 | 0.035 | 0.207 | 0.171 | 0.031 | 0.296 | 0.067 | 0.247 | 0.268 | 0.026 |
| *E. coli* P[4] with 13A1 | 60 | 63 | WTAV | 5.58 | 1 | 2 | 0.033 | 0.029 | 0.026 | 0.022 | 0.026 | 0.029 | 0.031 | 0.026 | 0.028 | 0.022 | 0.024 | 0.026 | 0.024 | 0.024 | 0.032 | 0.025 | 0.025 | 0.025 |
| *E. coli* P[4] with 13A1 | 60 | 64 | WTAVI | 7.81 | 1 | 3 | 0.053 | 0.045 | 0.04 | 0.048 | 0.043 | 0.046 | 0.048 | 0.043 | 0.042 | 0.048 | 0.052 | 0.042 | 0.042 | 0.043 | 0.046 | 0.052 | 0.035 | 0.037 |
| *E. coli* P[4] with 13A1 | 64 | 73 | IAVEPHVSQT | 4.1 | 2 | 7 | 1.394 | 1.513 | 1.458 | 1.903 | 1.836 | 1.865 | 2.211 | 2.191 | 2.211 | 2.354 | 2.362 | 2.336 | 2.688 | 2.683 | 2.687 | 3.232 | 3.263 | 3.211 |
| *E. coli* P[4] with 13A1 | 64 | 76 | IAVEPHVSQTNRQ | 3.65 | 2 | 10 | 2.473 | 2.677 | 2.613 | 3.367 | 3.241 | 3.318 | 3.884 | 3.784 | 3.873 | 4.134 | 4.158 | 4.066 | 4.824 | 4.814 | 4.811 | 5.548 | 5.648 | 5.49 |
| *E. coli* P[4] with 13A1 | 64 | 79 | IAVEPHVSQTNRQYIL | 7.05 | 3 | 13 | 2.197 | 2.352 | 2.281 | 3.172 | 3.139 | 3.201 | 3.948 | 3.831 | 3.907 | 4.192 | 4.174 | 4.107 | 4.759 | 4.809 | 4.824 | 5.434 | 5.48 | 5.356 |
| *E. coli* P[4] with 13A1 | 65 | 73 | AVEPHVSQT | 3.14 | 2 | 6 | 1.517 | 1.591 | 1.521 | 1.97 | 1.956 | 1.929 | 2.33 | 2.288 | 2.316 | 2.43 | 2.478 | 2.435 | 2.773 | 2.756 | 2.784 | 3.347 | 3.389 | 3.346 |
| *E. coli* P[4] with 13A1 | 65 | 76 | AVEPHVSQTNRQ | 2.89 | 3 | 9 | 2.561 | 2.772 | 2.707 | 3.517 | 3.369 | 3.462 | 4.025 | 3.962 | 4 | 4.306 | 4.318 | 4.243 | 5.008 | 4.989 | 4.997 | 5.777 | 5.861 | 5.723 |
| *E. coli* P[4] with 13A1 | 65 | 79 | AVEPHVSQTNRQYIL | 6.64 | 3 | 12 | 2.572 | 2.727 | 2.657 | 3.427 | 3.407 | 3.518 | 4.581 | 4.093 | 4.215 | 4.535 | 4.425 | 4.486 | 5.002 | 5.165 | 5.089 | 5.842 | 5.755 | 5.804 |
| *E. coli* P[4] with 13A1 | 74 | 79 | NRQYIL | 6.48 | 2 | 4 | 1.004 | 0.852 | 0.862 | 1.449 | 1.456 | 1.465 | 1.881 | 1.843 | 1.924 | 1.919 | 1.843 | 1.926 | 1.92 | 1.965 | 1.932 | 1.954 | 1.914 | 1.937 |
| *E. coli* P[4] with 13A1 | 80 | 86 | FGENKQF | 5.21 | 2 | 5 | 1.196 | 1.306 | 1.254 | 1.361 | 1.32 | 1.341 | 1.376 | 1.361 | 1.375 | 1.412 | 1.408 | 1.382 | 1.528 | 1.52 | 1.517 | 1.894 | 1.897 | 1.854 |
| *E. coli* P[4] with 13A1 | 80 | 97 | FGENKQFNVENNSDKWKF | 8.1 | 4 | 16 | 2.284 | 2.551 | 2.454 | 2.582 | 2.595 | 2.626 | 2.935 | 2.887 | 2.947 | 3.838 | 3.866 | 3.844 | 4.99 | 5.081 | 5.096 | 5.682 | 5.754 | 5.671 |
| *E. coli* P[4] with 13A1 | 84 | 97 | KQFNVENNSDKWKF | 7.49 | 4 | 12 | 1.107 | 1.266 | 1.212 | 1.252 | 1.222 | 1.272 | 1.526 | 1.533 | 1.584 | 2.37 | 2.456 | 2.461 | 3.555 | 3.652 | 3.708 | 4.458 | 4.236 | 4.281 |
| *E. coli* P[4] with 13A1 | 98 | 100 | FEM | 6.24 | 1 | 1 | 0.032 | 0.033 | 0.022 | 0.021 | 0.026 | 0.024 | 0.022 | 0.021 | 0.027 | 0.026 | 0.018 | 0.022 | 0.02 | 0.023 | 0.021 | 0.022 | 0.023 | 0.02 |
| *E. coli* P[4] with 13A1 | 98 | 109 | FEMFKGSSQGDF | 8.35 | 2 | 10 | 3.571 | 3.851 | 3.765 | 4.011 | 3.735 | 3.946 | 4.137 | 4.032 | 4.159 | 4.253 | 4.159 | 4.105 | 4.301 | 4.339 | 4.279 | 4.393 | 4.403 | 4.313 |
| *E. coli* P[4] with 13A1 | 98 | 121 | FEMFKGSSQGDFSNRRTLTSSNRL | 7.18 | 3 | 22 | 5.181 | 5.585 | 5.156 | 5.722 | 5.637 | 5.836 | 6.225 | 6.192 | 6.359 | 6.858 | 7.039 | 7.058 | 7.287 | 7.632 | 7.618 | 7.226 | 7.408 | 7.183 |
| *E. coli* P[4] with 13A1 | 101 | 109 | FKGSSQGDF | 4.88 | 2 | 7 | 3.815 | 4.151 | 4.001 | 4.311 | 4.223 | 4.355 | 4.515 | 4.441 | 4.604 | 4.847 | 4.842 | 4.598 | 4.838 | 4.861 | 4.888 | 4.648 | 4.667 | 4.625 |
| *E. coli* P[4] with 13A1 | 101 | 121 | FKGSSQGDFSNRRTLTSSNRL | 5.31 | 3 | 19 | 5.803 | 6.453 | 6.227 | 6.769 | 6.811 | 7.02 | 7.518 | 7.43 | 7.714 | 8.246 | 8.304 | 8.227 | 8.74 | 8.976 | 8.973 | 8.766 | 8.97 | 8.712 |
| *E. coli* P[4] with 13A1 | 101 | 123 | FKGSSQGDFSNRRTLTSSNRLVG | 5.49 | 4 | 21 | 5.626 | 6.256 | 6.055 | 6.642 | 6.556 | 6.826 | 7.333 | 7.256 | 7.483 | 8.038 | 8.037 | 7.94 | 8.476 | 8.771 | 8.717 | 8.612 | 8.859 | 8.573 |
| *E. coli* P[4] with 13A1 | 101 | 125 | FKGSSQGDFSNRRTLTSSNRLVGML | 7.76 | 4 | 23 | 5.441 | 6.406 | 5.944 | 6.606 | 6.373 | 6.725 | 7.383 | 7.081 | 7.481 | 8.32 | 7.679 | 7.896 | 8.433 | 8.573 | 8.471 | 8.805 | 8.457 | 8.486 |
| *E. coli* P[4] with 13A1 | 102 | 121 | KGSSQGDFSNRRTLTSSNRL | 4.92 | 4 | 18 | 5.352 | 5.845 | 5.856 | 6.585 | 6.282 | 6.622 | 6.849 | 7.279 | 7.143 | 7.644 | 8.104 | 7.754 | 8.254 | 8.391 | 8.429 | 8.178 | 9.245 | 8.314 |
| *E. coli* P[4] with 13A1 | 122 | 124 | VGM | 2.87 | 1 | 1 | 0.061 | 0.112 | 0.115 | 0.044 | 0.031 | 0.059 | 0.027 | 0.041 | 0.024 | 0.035 | 0.08 | 0.078 | 0.041 | 0.056 | 0.037 | 0.146 | 0.123 | 0.199 |
| *E. coli* P[4] with 13A1 | 122 | 134 | VGMLKYGGRVWTF | 9.52 | 3 | 11 | 1.046 | 1.307 | 1.296 | 1.208 | 1.261 | 1.303 | 1.211 | 1.251 | 1.282 | 1.219 | 1.331 | 1.327 | 1.459 | 1.51 | 1.51 | 2.058 | 2.068 | 1.999 |
| *E. coli* P[4] with 13A1 | 122 | 147 | VGMLKYGGRVWTFHGETPRATTDSSN | 7.37 | 4 | 23 | 2.054 | 2.668 | 2.236 | 2.335 | 2.757 | 2.753 | 3.211 | 2.589 | 4.315 | 4.095 | 4.367 | 4.372 | 4.116 | 4.628 | 4.692 | 4.978 | 5.942 | 5.966 |
| *E. coli* P[4] with 13A1 | 122 | 151 | VGMLKYGGRVWTFHGETPRATTDSSNTADL | 7.85 | 4 | 27 | 3.512 | 4.077 | 3.87 | 4.726 | 4.726 | 4.734 | 5.543 | 5.573 | 5.693 | 6.196 | 6.252 | 6.197 | 6.991 | 7.17 | 7.495 | 8.243 | 8.362 | 8.763 |
| *E. coli* P[4] with 13A1 | 122 | 153 | VGMLKYGGRVWTFHGETPRATTDSSNTADLNN | 7.48 | 4 | 29 | 5.087 | 5.553 | 4.936 | 6.353 | 7.331 | 6.023 | 7.305 | 7.205 | 7.102 | 7.918 | 7.836 | 7.823 | 8.852 | 8.927 | 9.018 | 10.286 | 10.212 | 10.052 |
| *E. coli* P[4] with 13A1 | 122 | 155 | VGMLKYGGRVWTFHGETPRATTDSSNTADLNNIS | 7.78 | 4 | 31 | 4.973 | 5.132 | 4.926 | 6.193 | 5.869 | 5.906 | 7.22 | 6.874 | 7.745 | 7.579 | 7.588 | 7.637 | 8.195 | 8.37 | 8.868 | 10.565 | 9.888 | 9.481 |
| *E. coli* P[4] with 13A1 | 124 | 134 | MLKYGGRVWTF | 9.2 | 3 | 9 | 1.083 | 1.349 | 1.348 | 1.188 | 1.265 | 1.328 | 1.208 | 1.289 | 1.306 | 1.208 | 1.336 | 1.335 | 1.45 | 1.535 | 1.481 | 1.974 | 2.024 | 2.001 |
| *E. coli* P[4] with 13A1 | 124 | 151 | MLKYGGRVWTFHGETPRATTDSSNTADL | 7.49 | 4 | 25 | 3.542 | 4.032 | 3.936 | 4.679 | 4.937 | 4.913 | 5.418 | 5.671 | 5.673 | 6.087 | 6.223 | 6.11 | 6.998 | 7.093 | 7.145 | 8.225 | 8.324 | 9.646 |
| *E. coli* P[4] with 13A1 | 125 | 134 | LKYGGRVWTF | 8.88 | 3 | 8 | 1.147 | 1.335 | 1.299 | 1.208 | 1.277 | 1.32 | 1.187 | 1.285 | 1.331 | 1.255 | 1.353 | 1.305 | 1.427 | 1.554 | 1.529 | 1.971 | 1.966 | 1.93 |
| *E. coli* P[4] with 13A1 | 125 | 151 | LKYGGRVWTFHGETPRATTDSSNTADL | 7.17 | 4 | 24 | 3.594 | 4.111 | 3.945 | 4.765 | 4.75 | 4.817 | 5.608 | 5.662 | 5.776 | 6.259 | 6.299 | 6.233 | 7.036 | 7.122 | 7.161 | 8.21 | 8.322 | 8.198 |
| *E. coli* P[4] with 13A1 | 125 | 153 | LKYGGRVWTFHGETPRATTDSSNTADLNN | 6.72 | 4 | 26 | 5.239 | 5.818 | 5.527 | 6.64 | 6.32 | 6.615 | 7.618 | 7.481 | 7.514 | 8.159 | 8.243 | 8.051 | 8.882 | 9.135 | 9.105 | 10.406 | 10.375 | 10.183 |
| *E. coli* P[4] with 13A1 | 125 | 155 | LKYGGRVWTFHGETPRATTDSSNTADLNNIS | 7.15 | 4 | 28 | 5.725 | 6.191 | 5.912 | 7.439 | 7.229 | 7.296 | 8.459 | 8.266 | 8.313 | 9.156 | 8.968 | 8.974 | 10.058 | 10.008 | 10.017 | 11.444 | 11.318 | 11.052 |
| *E. coli* P[4] with 13A1 | 135 | 151 | HGETPRATTDSSNTADL | 4.1 | 3 | 14 | 2.697 | 2.939 | 2.898 | 3.985 | 3.869 | 3.924 | 4.96 | 4.92 | 4.984 | 5.657 | 5.693 | 5.587 | 6.431 | 6.418 | 6.376 | 7.222 | 7.334 | 7.175 |
| *E. coli* P[4] with 13A1 | 135 | 155 | HGETPRATTDSSNTADLNNIS | 4.78 | 3 | 18 | 4.914 | 5.145 | 5.172 | 6.312 | 6.309 | 6.347 | 7.941 | 7.572 | 7.596 | 7.813 | 8.402 | 8.278 | 8.615 | 8.676 | 8.418 | 9.124 | 9.343 | 9.392 |
| *E. coli* P[4] with 13A1 | 154 | 162 | ISIIIHSEF | 8.4 | 2 | 7 | 0.098 | 0.107 | 0.107 | 0.172 | 0.173 | 0.18 | 0.366 | 0.352 | 0.352 | 0.668 | 0.665 | 0.663 | 0.963 | 0.975 | 0.972 | 1.53 | 1.54 | 1.494 |
| *E. coli* P[4] with 13A1 | 156 | 161 | IIIHSE | 3.79 | 2 | 4 | 0.098 | 0.104 | 0.096 | 0.118 | 0.148 | 0.153 | 0.296 | 0.275 | 0.301 | 0.578 | 0.554 | 0.544 | 0.843 | 0.839 | 0.807 | 1.297 | 1.342 | 1.211 |
| *E. coli* P[4] with 13A1 | 156 | 162 | IIIHSEF | 6.9 | 2 | 5 | 0.092 | 0.108 | 0.104 | 0.15 | 0.151 | 0.149 | 0.322 | 0.299 | 0.308 | 0.591 | 0.575 | 0.567 | 0.811 | 0.83 | 0.824 | 1.247 | 1.253 | 1.192 |
| *E. coli* P[4] with 13A1 | 157 | 162 | IIHSEF | 5.6 | 2 | 4 | 0.126 | 0.11 | 0.09 | 0.119 | 0.102 | 0.144 | 0.268 | 0.219 | 0.243 | 0.439 | 0.431 | 0.422 | 0.649 | 0.635 | 0.641 | 0.997 | 1.013 | 0.941 |
| *E. coli* P[4] with 13A1 | 163 | 170 | YIIPRSQE | 4.82 | 2 | 5 | 1.164 | 1.219 | 1.173 | 1.451 | 1.39 | 1.459 | 2.024 | 1.97 | 1.971 | 2.631 | 2.563 | 2.576 | 2.907 | 3 | 2.883 | 3.393 | 3.398 | 3.376 |
| *E. coli* P[4] with 13A1 | 163 | 173 | YIIPRSQESKC | 4.63 | 2 | 8 | 1.821 | 1.815 | 1.677 | 2.275 | 2.206 | 2.345 | 3.089 | 2.857 | 3.099 | 3.732 | 3.612 | 3.604 | 4.327 | 4.337 | 4.383 | 5.017 | 4.739 | 4.754 |
| *E. coli* P[4] with 13A1 | 163 | 175 | YIIPRSQESKCNE | 4.57 | 3 | 10 | 1.544 | 1.618 | 1.586 | 2.092 | 1.969 | 2.041 | 2.866 | 2.722 | 2.79 | 3.609 | 3.539 | 3.456 | 4.53 | 4.483 | 4.435 | 5.205 | 5.257 | 5.088 |
| *E. coli* P[4] with 13A1 | 174 | 181 | NEYINNGL | 6.32 | 1 | 6 | 0.583 | 0.372 | 0.476 | 0.567 | 0.357 | 0.615 | 0.59 | 0.714 | 0.6 | 0.969 | 0.916 | 0.893 | 1.071 | 1.055 | 1.187 | 1.182 | 1.089 | 1.127 |

1-3: MQY (#1)

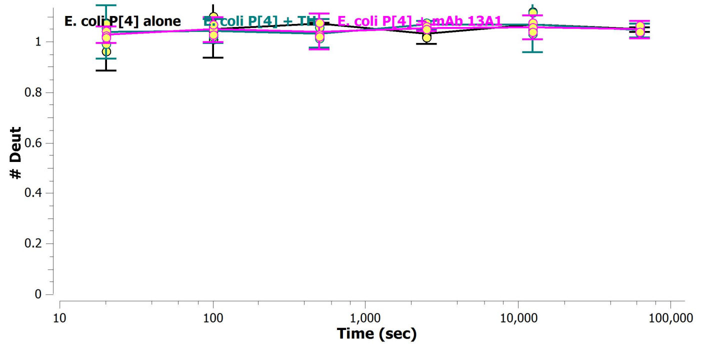


1-6: MQYIKA (#2)

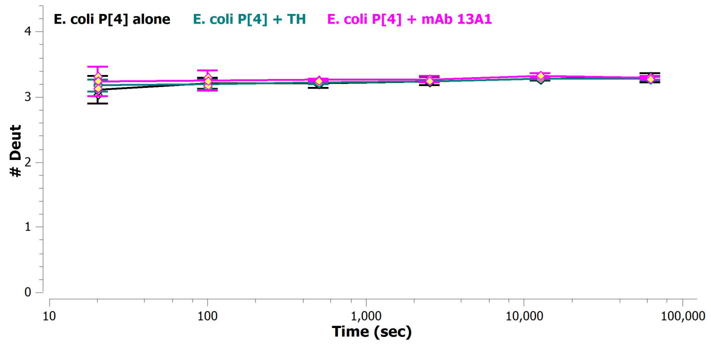


1-10: MQYIKANSKF (#3)

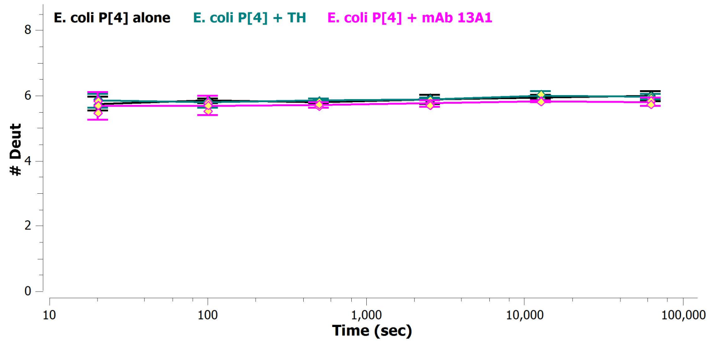


1-12: MQYIKANSKFIG (#4)

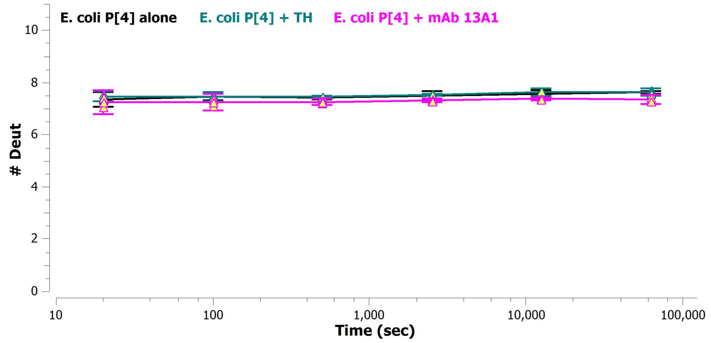


3-10: YIKANSKF (#5)

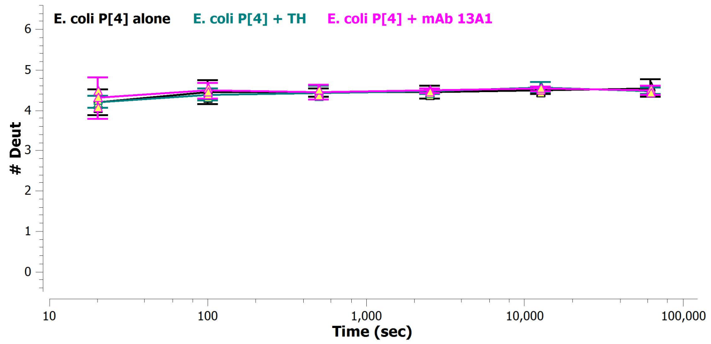


11-15: IGITE (#6)

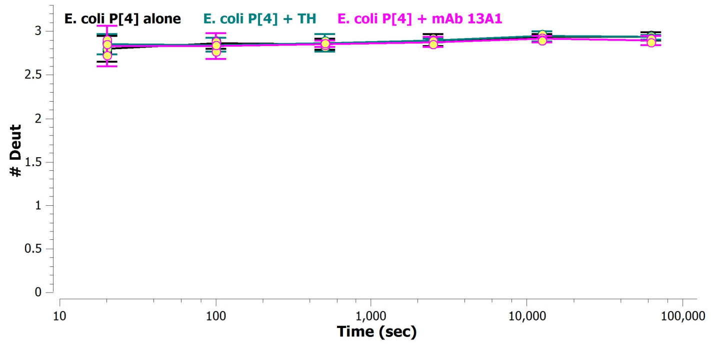


11-23: IGITELGSGSGVL (#7)

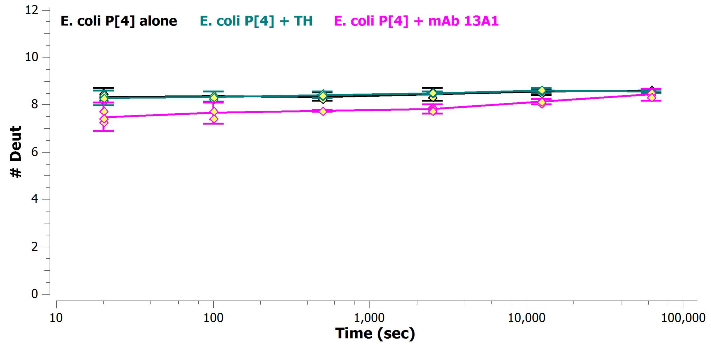


11-37: IGITELGSGSGVLDGPYQPTTFKPPND (#8)

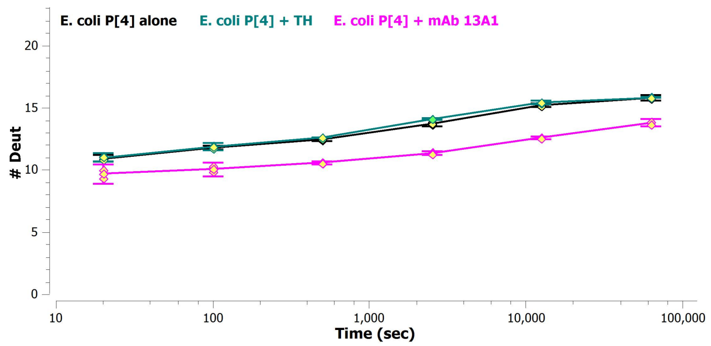


13-23: ITELGSGSGVL (#9)

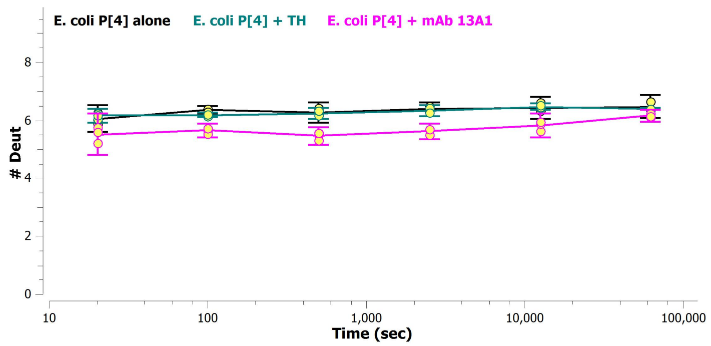


13-37: ITELGSGSGVLDGPYQPTTFKPPND (#10)

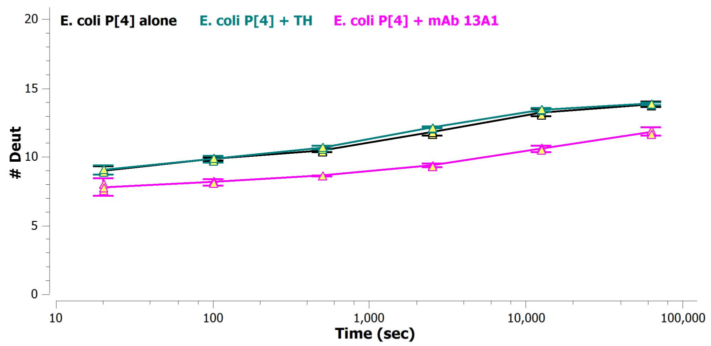

**16-37: LGSGSGVLDGPYQPTTFKPPND (#11)**

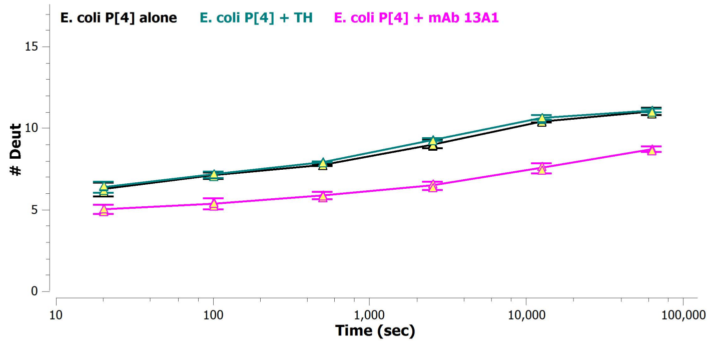


**16-38: LGSGSGVLDGPYQPTTFKPPNDY (#12)**

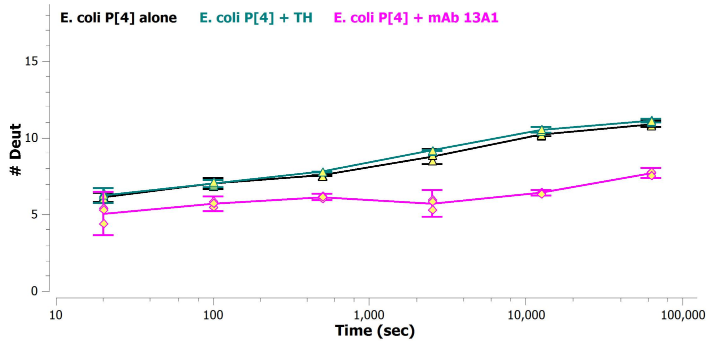


**16-39: LGSGSGVLDGPYQPTTFKPPNDYW (#13)**

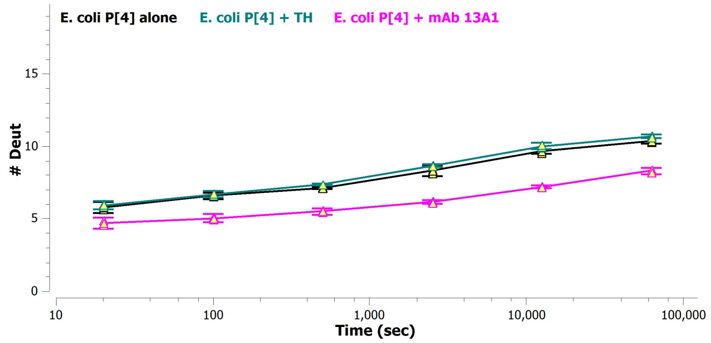


**17-37: GSGSGVLDGPYQPTTFKPPND (#14)**

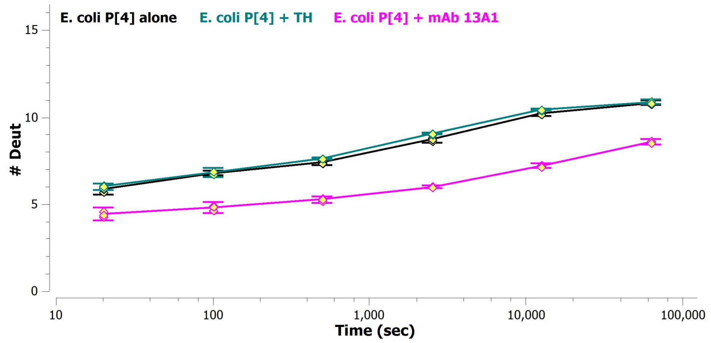


**17-39: GSGSGVLDGPYQPTTFKPPNDYW (#15)**

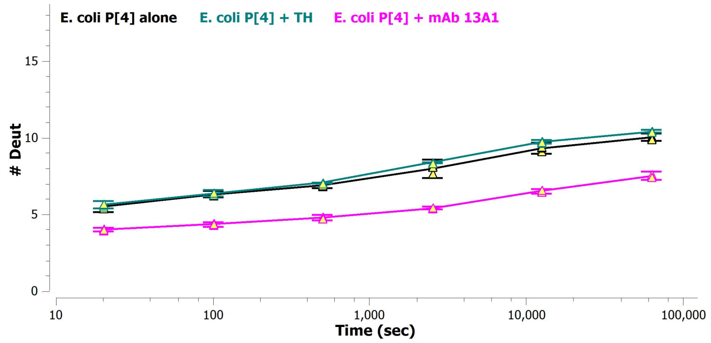


**24-37: DGPYQPTTFKPPND (#16)**

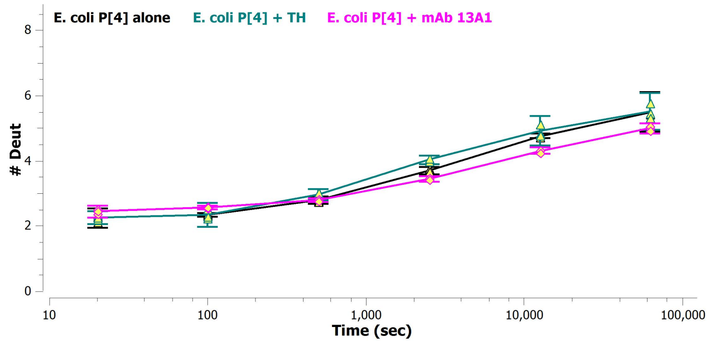


**24-39: DGPYQPTTFKPPNDYW (#17)**

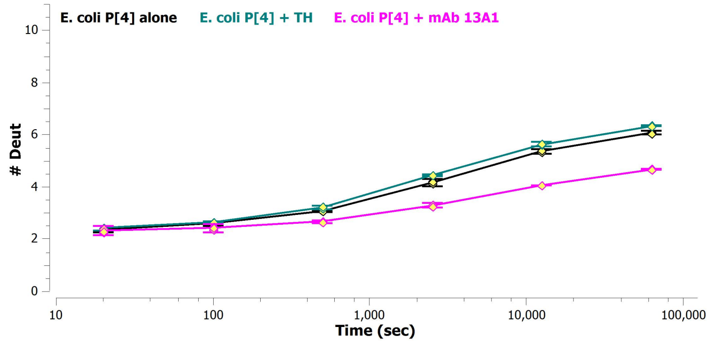


**38-41: YWLL (#18)**

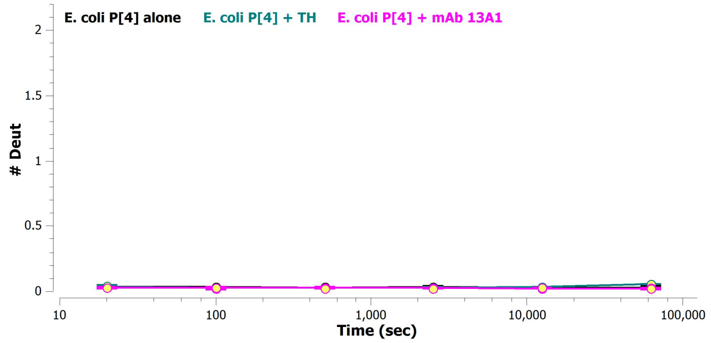


**40-51: LLISSNTNGVVY (#19)**

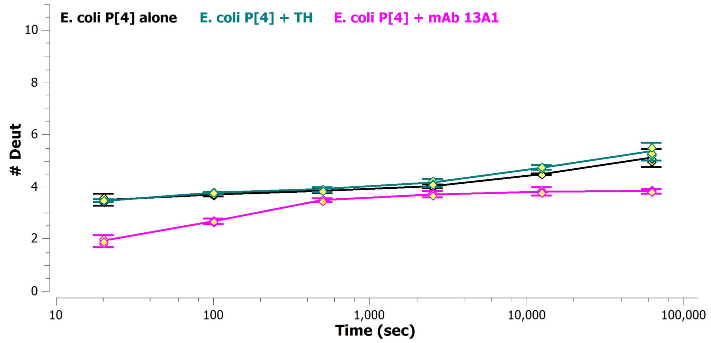


**40-59: LLISSNTNGVVYESTNNNDF (#20)**

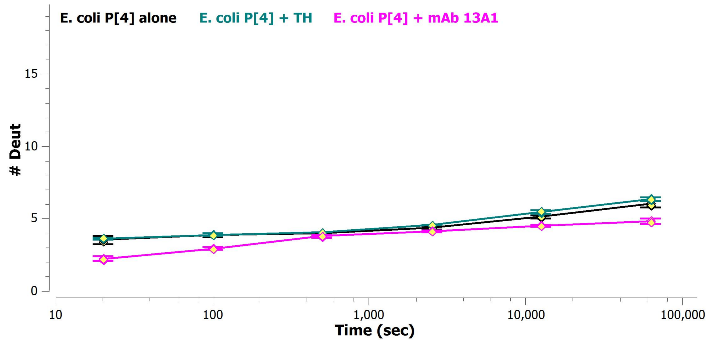

41-51: LISSNTNGVVY (#21)

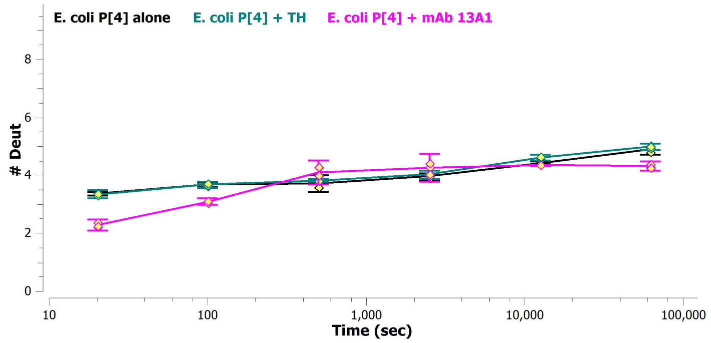


42-59: ISSNTNGVVYESTNNNDF (#22)

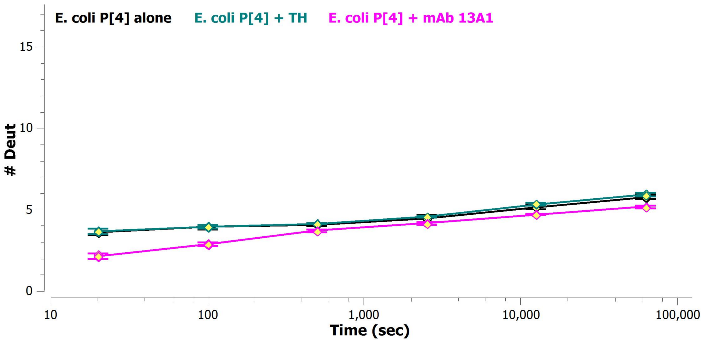


52-59: ESTNNNDF (#23)

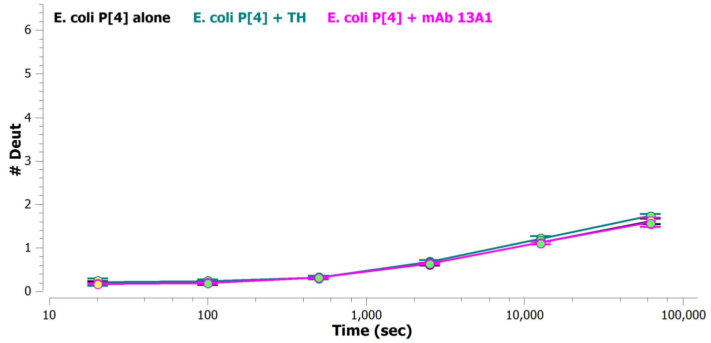


59-63: FWTAV (#24)

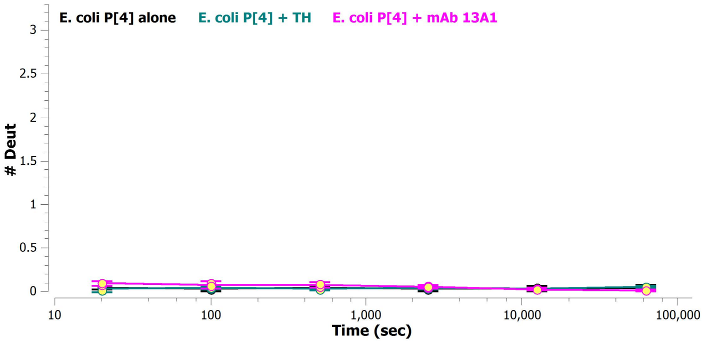


59-64: FWTAVI (#25)

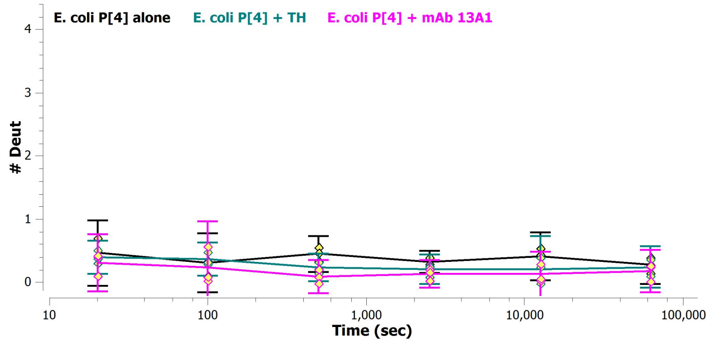


60-63: WTAV (#26)

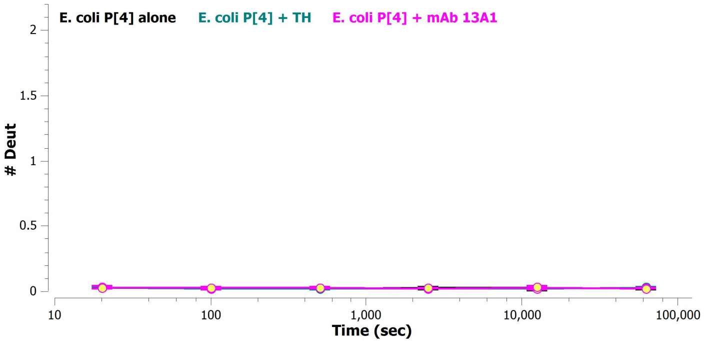


60-64: WTAVI (#27)

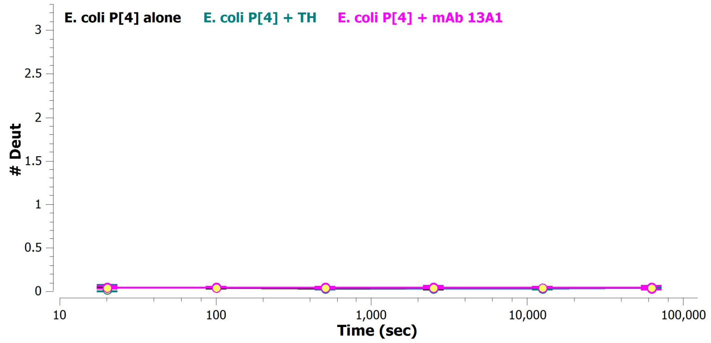


64-73: IAVEPHVSQT (#28)

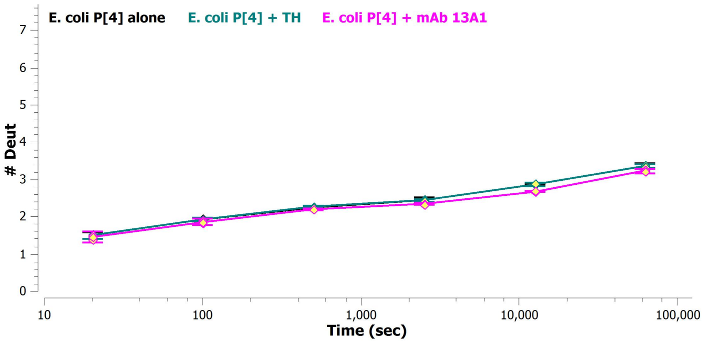


64-76: IAVEPHVSQTNRQ (#29)

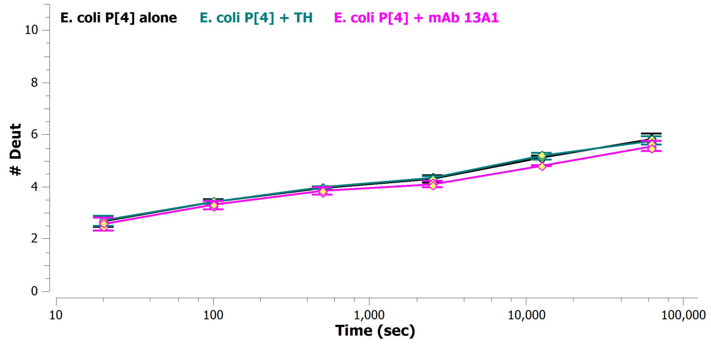


64-79: IAVEPHVSQTNRQYIL (#30)

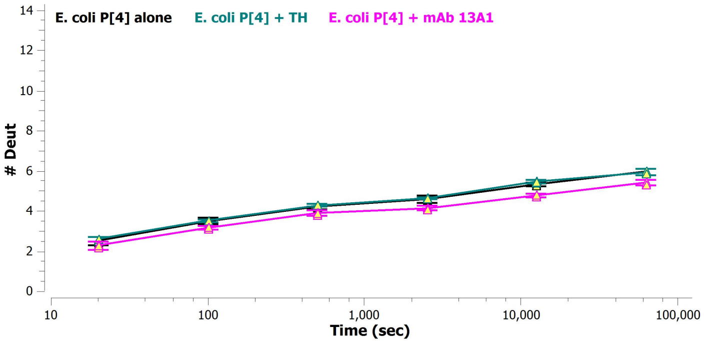

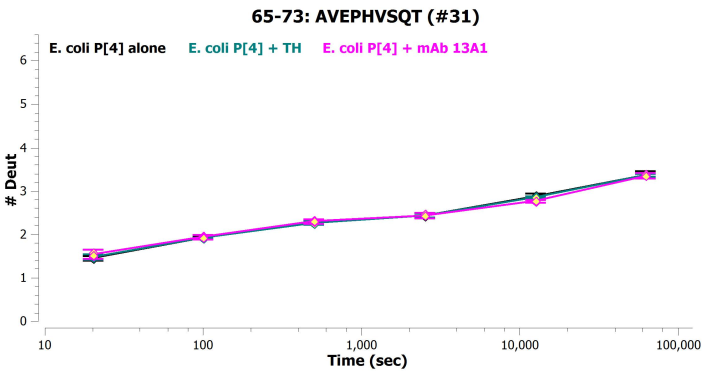
65-73: AVEPHVSQT (#31)
E. coli P[4] alone
E. coli P[4] + TH
E. coli P[4] + mAb 13A1
# Deut
Time (sec)

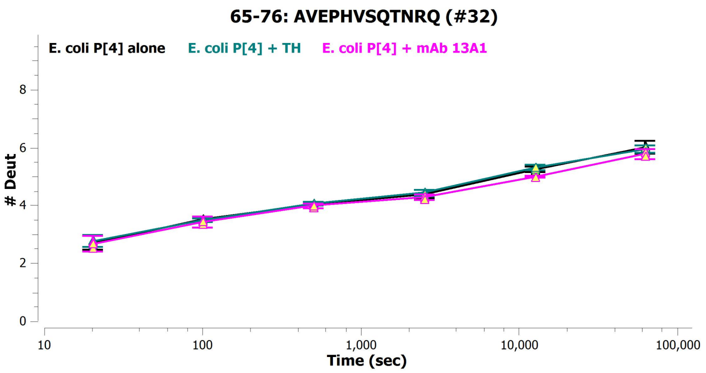
65-76: AVEPHVSQTNRQ (#32)
E. coli P[4] alone
E. coli P[4] + TH
E. coli P[4] + mAb 13A1
# Deut
Time (sec)

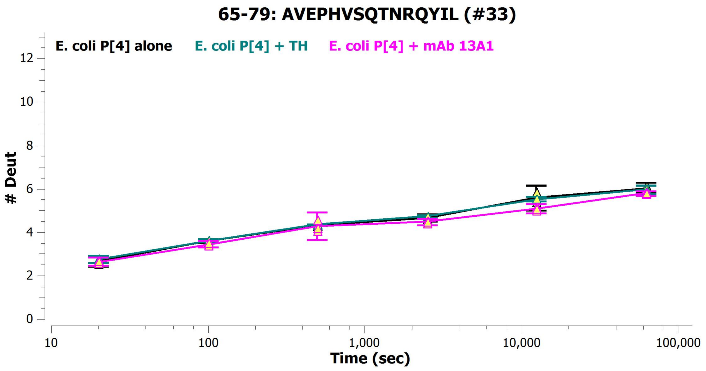
65-79: AVEPHVSQTNRQYIL (#33)
E. coli P[4] alone
E. coli P[4] + TH
E. coli P[4] + mAb 13A1
# Deut
Time (sec)

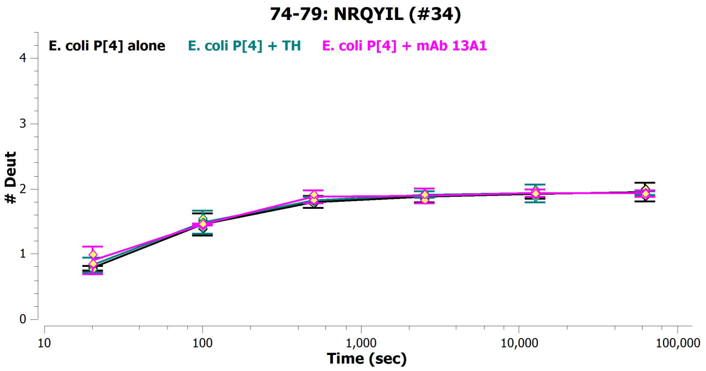
74-79: NRQYIL (#34)
E. coli P[4] alone
E. coli P[4] + TH
E. coli P[4] + mAb 13A1
# Deut
Time (sec)

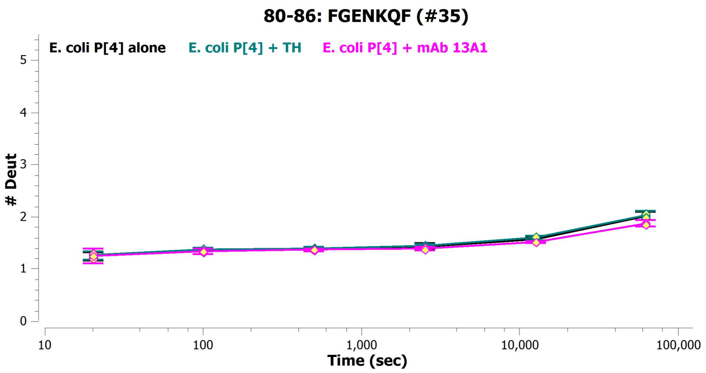
80-86: FGENKQF (#35)
E. coli P[4] alone
E. coli P[4] + TH
E. coli P[4] + mAb 13A1
# Deut
Time (sec)

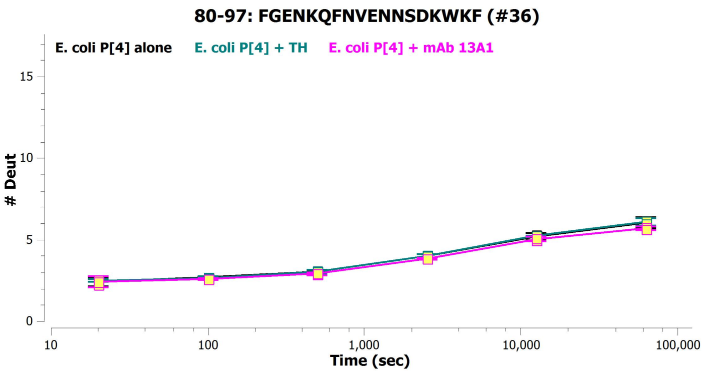
80-97: FGENKQFNVENNSDKWKF (#36)
E. coli P[4] alone
E. coli P[4] + TH
E. coli P[4] + mAb 13A1
# Deut
Time (sec)

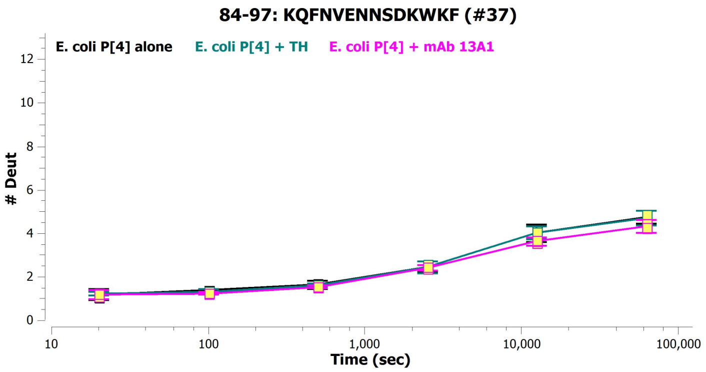
84-97: KQFNVENNSDKWKF (#37)
E. coli P[4] alone
E. coli P[4] + TH
E. coli P[4] + mAb 13A1
# Deut
Time (sec)

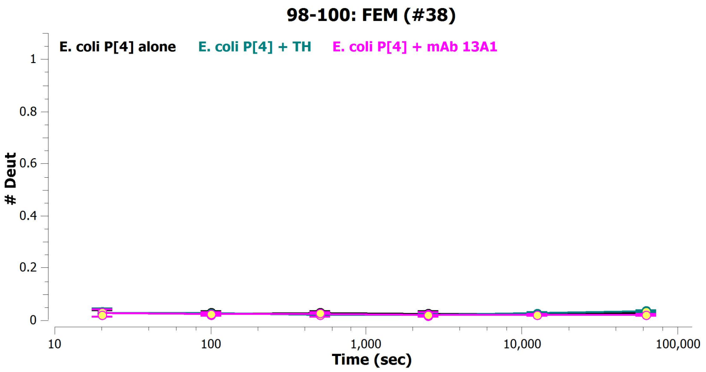
98-100: FEM (#38)
E. coli P[4] alone
E. coli P[4] + TH
E. coli P[4] + mAb 13A1
# Deut
Time (sec)

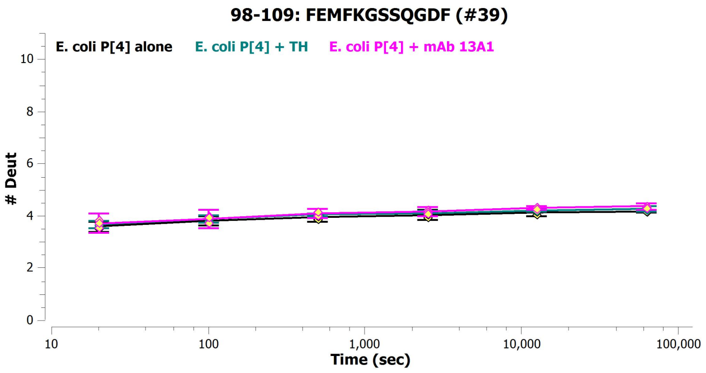
98-109: FEMFKGSSQGDF (#39)
E. coli P[4] alone
E. coli P[4] + TH
E. coli P[4] + mAb 13A1
# Deut
Time (sec)

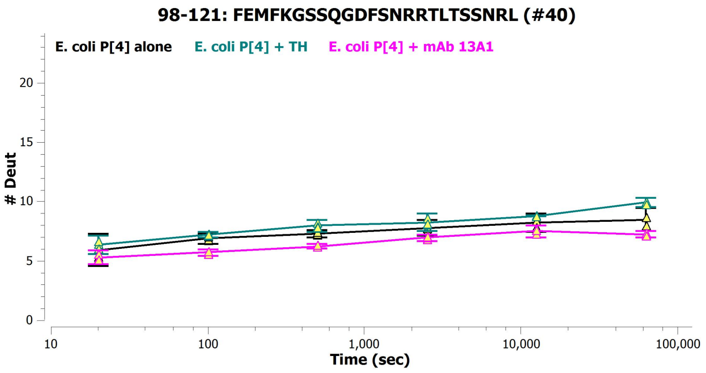
98-121: FEMFKGSSQGDFSNRRTLTSSNRL (#40)
E. coli P[4] alone
E. coli P[4] + TH
E. coli P[4] + mAb 13A1
# Deut
Time (sec)

101-109: FKGSSQGDF (#41)

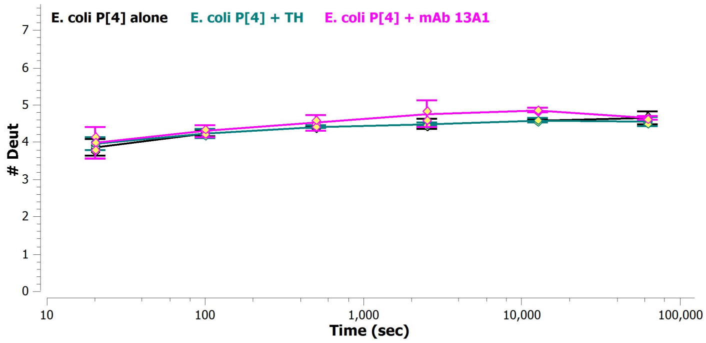


101-121: FKGSSQGDFSNRRTLTSSNRL (#42)

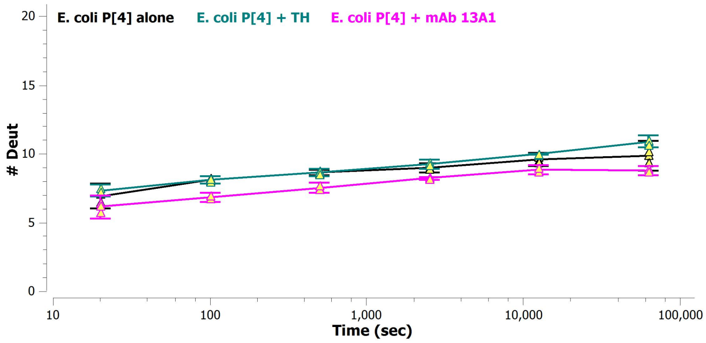


101-123: FKGSSQGDFSNRRTLTSSNRLVG (#43)

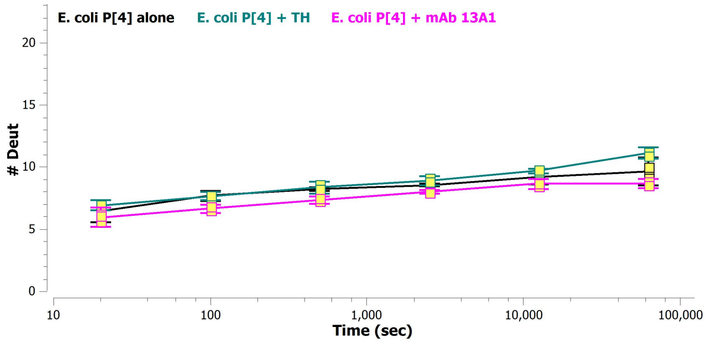


101-125: FKGSSQGDFSNRRTLTSSNRLVGML (#44)

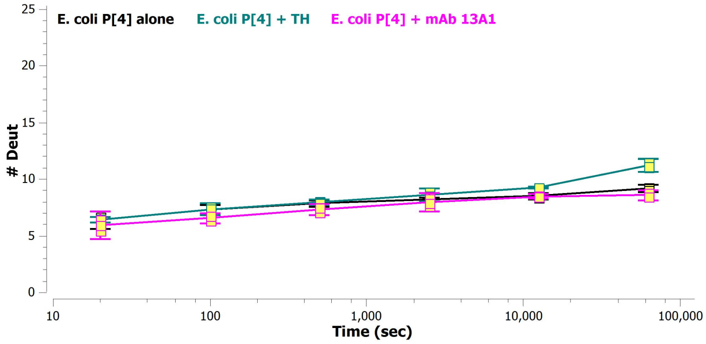


102-121: KGSSQGDFSNRRTLTSSNRL (#45)

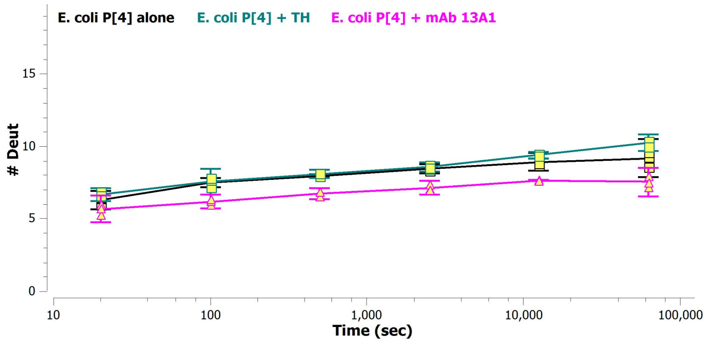


122-124: VGM (#46)

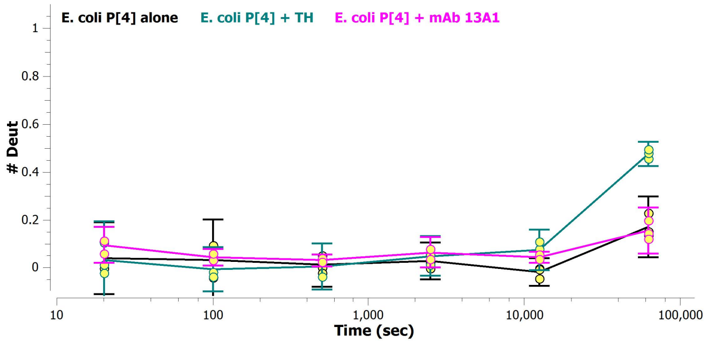


122-134: VGMLKYGGRVWTF (#47)

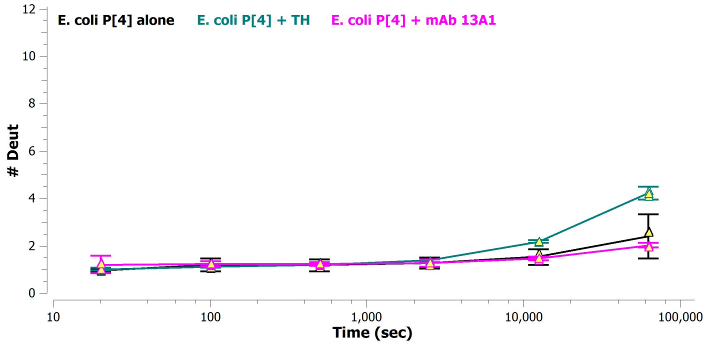


122-147: VGMLKYGGRVWTFHGETPRATTDSSN (#48)

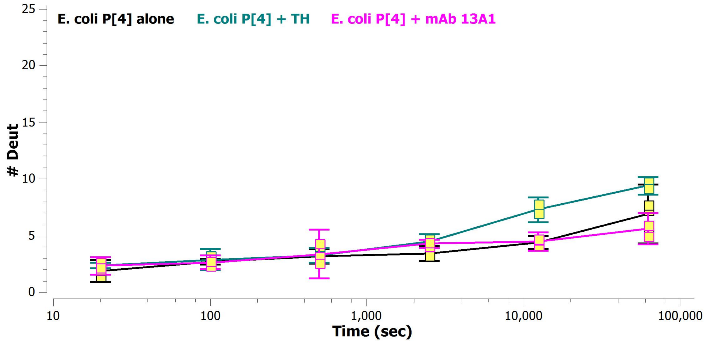


122-151: VGMLKYGGRVWTFHGETPRATTDSSNTADL (#49)

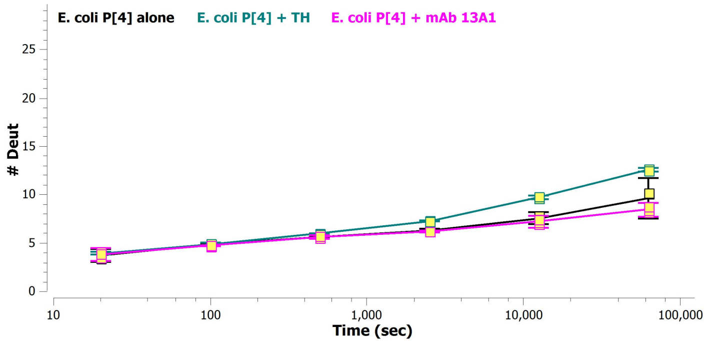


122-153: VGMLKYGGRVWTFHGETPRATTDSSNTADLNN (#50)

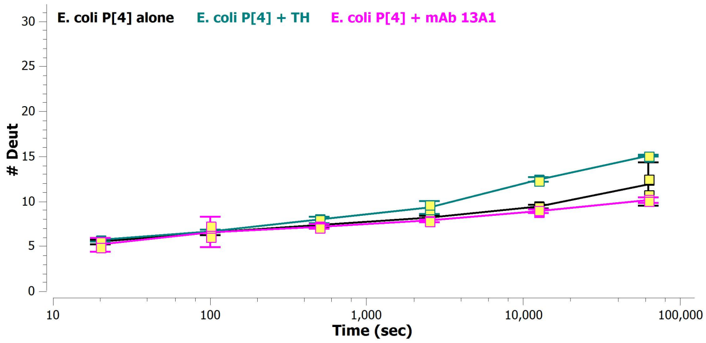

**122-155: VGMLKYGGRVWTFHGETPRATTDSSNTADLNNIS (#51)**

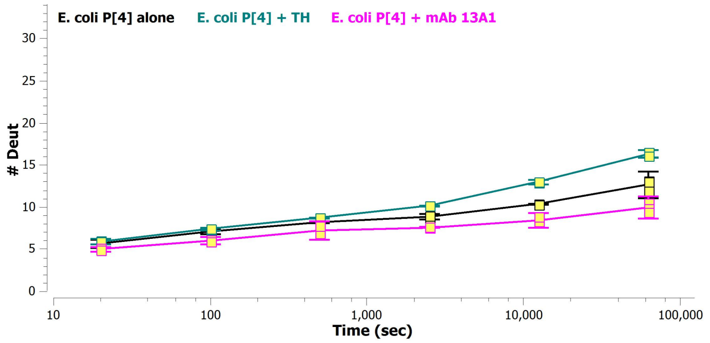


**124-134: MLKYGGRVWTF (#52)**

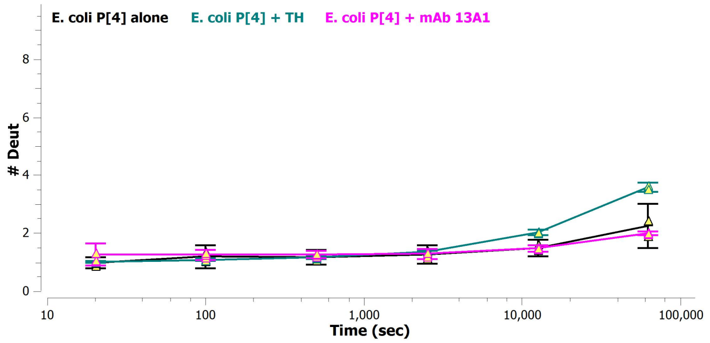


**124-151: MLKYGGRVWTFHGETPRATTDSSNTADL (#53)**

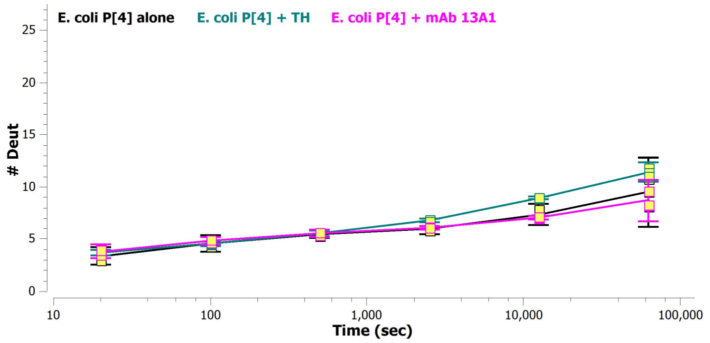


**125-134: LKYGGRVWTF (#54)**

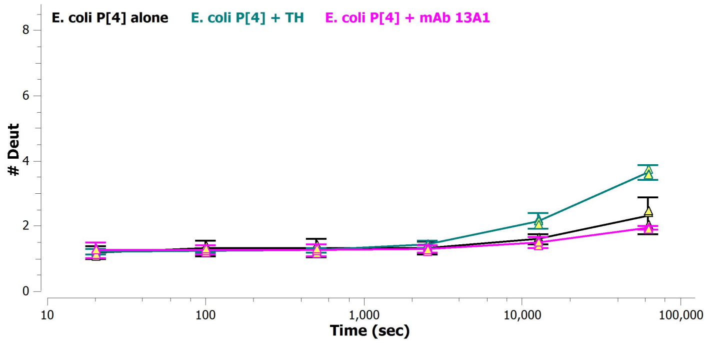


**125-151: LKYGGRVWTFHGETPRATTDSSNTADL (#55)**

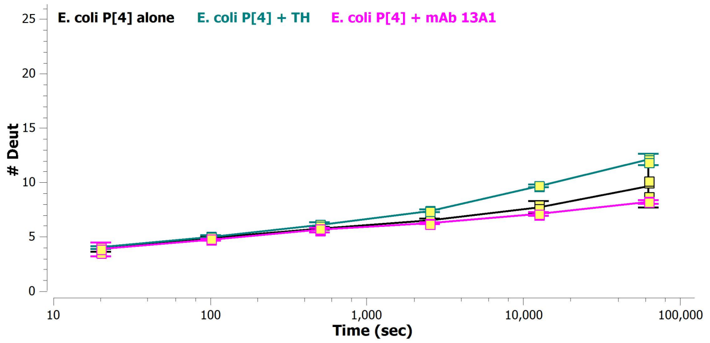


**125-153: LKYGGRVWTFHGETPRATTDSSNTADLNN (#56)**

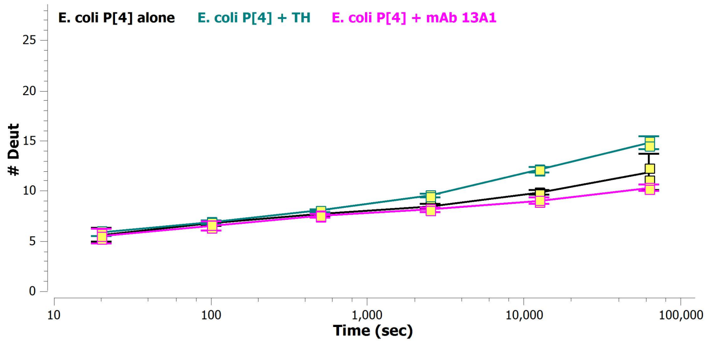


**125-155: LKYGGRVWTFHGETPRATTDSSNTADLNNIS (#57)**

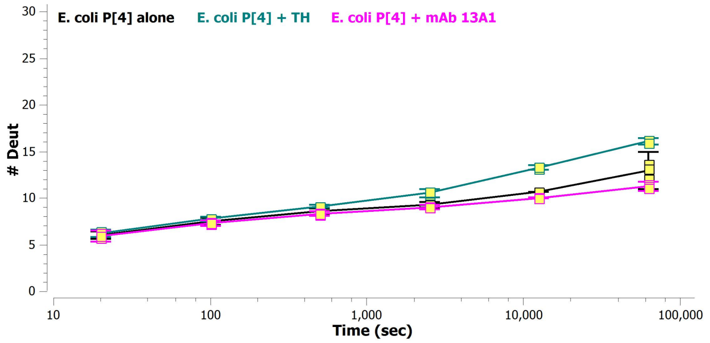


**135-151: HGETPRATTDSSNTADL (#58)**

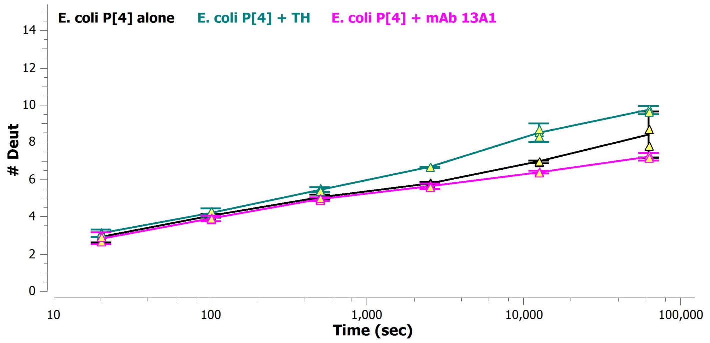


**135-155: HGETPRATTDSSNTADLNNIS (#59)**

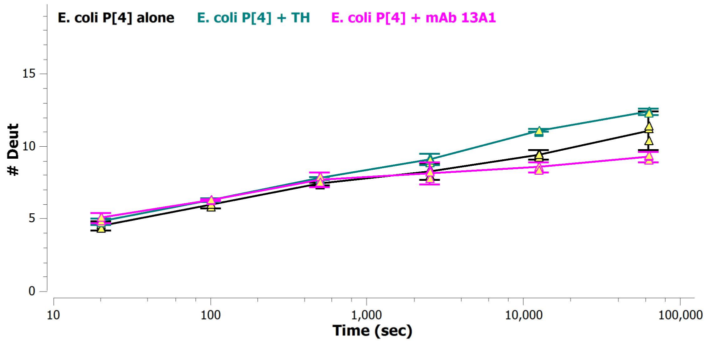


**154-162: ISIIIHSEF (#60)**

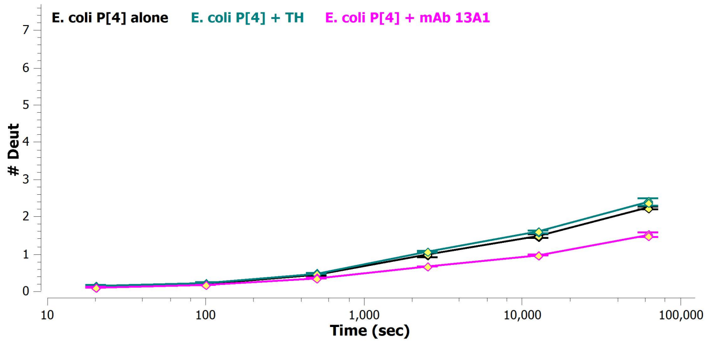

### 156-161: IIIHSE (#61)

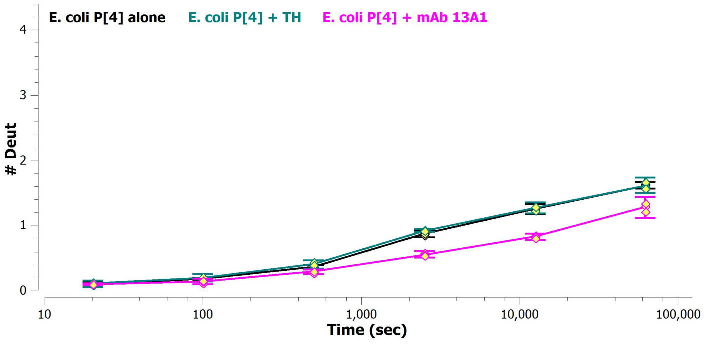


### 156-162: IIIHSEF (#62)

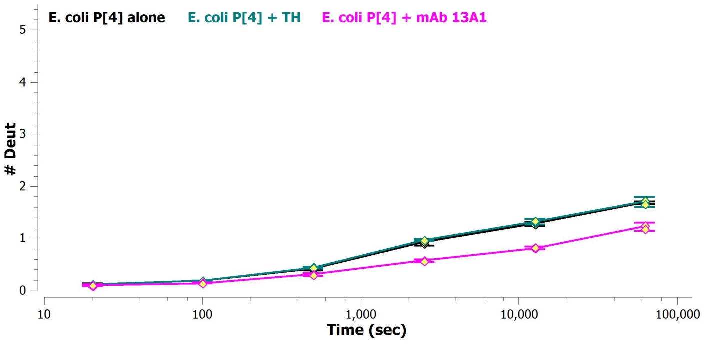


### 157-162: IIHSEF (#63)

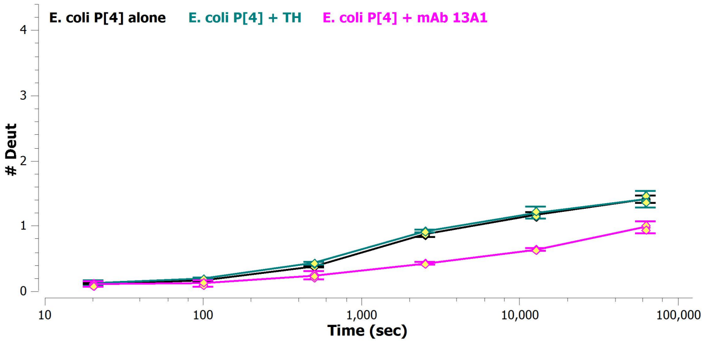


### 163-170: YIIPRSQE (#64)

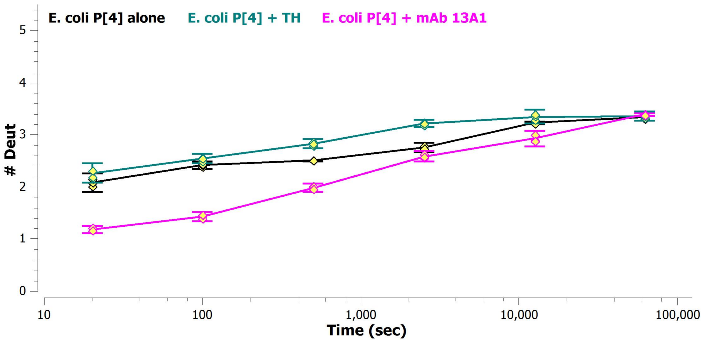


### 163-173: YIIPRSQESKC (#65)

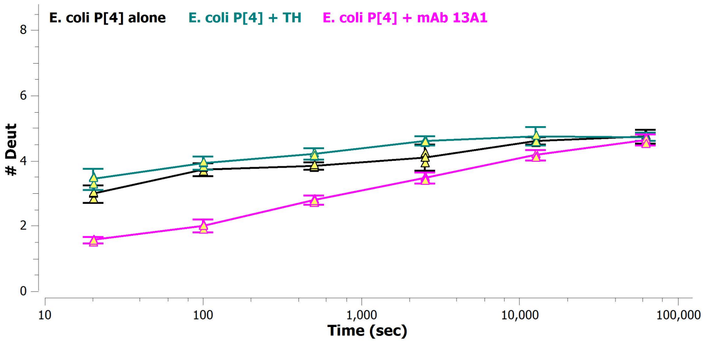


### 163-175: YIIPRSQESKCNE (#66)

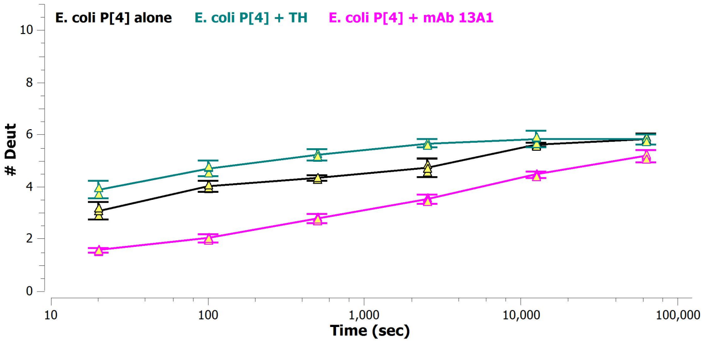


### 174-181: NEYINNGL (#67)

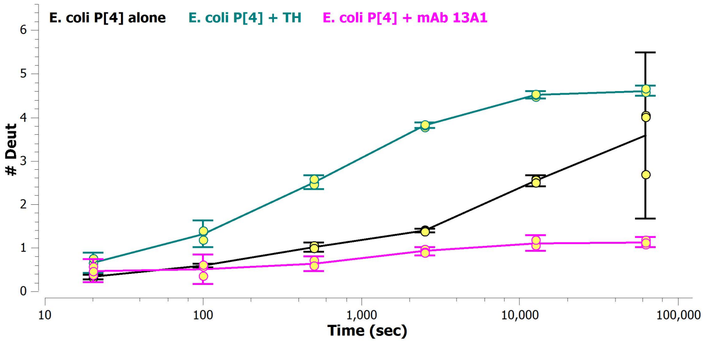

| | | | | | | | 20s | 20s | 20s | 100s | 100s | 100s | 500s | 500s | 500s | 2500s | 2500s | 2500s | 12500s | 12500s | 12500s | 62500s | 62500s | 62500s |
|---|---|---|---|---|---|---|---|---|---|---|---|---|---|---|---|---|---|---|---|---|---|---|---|---|
| State | Start | End | Sequence | Search RT | Charge | Max D | #D | #D | #D | #D | #D | #D | #D | #D | #D | #D | #D | #D | #D | #D | #D | #D | #D | #D |
| *Pp* P[4] alone | 1 | 6 | MQYIKA | 4.94 | 2 | 4 | 2.875 | 2.999 | 2.958 | 3.035 | 3.189 | 3.173 | 3.107 | 3.118 | 3.224 | 3.052 | 3.163 | 3.177 | 3.202 | 3.145 | 3.217 | 3.33 | 3.234 | 3.252 |
| *Pp* P[4] alone | 1 | 10 | MQYIKANSKF | 5.85 | 3 | 8 | 5.993 | 6.138 | 5.966 | 5.898 | 5.967 | 6.052 | 5.832 | 5.909 | 5.968 | 5.836 | 5.952 | 5.976 | 6.036 | 6.041 | 6.046 | 6.073 | 6.071 | 6.091 |
| *Pp* P[4] alone | 1 | 12 | MQYIKANSKFIG | 6.37 | 3 | 10 | 7.51 | 7.396 | 6.658 | 6.902 | 7.356 | 7.492 | 7.453 | 7.228 | 7.187 | 7.098 | 7.417 | 7.282 | 7.446 | 7.411 | 7.516 | 7.497 | 7.611 | 7.649 |
| *Pp* P[4] alone | 3 | 10 | YIKANSKF | 4.31 | 3 | 6 | 4.359 | 4.499 | 4.446 | 4.461 | 4.479 | 4.471 | 4.444 | 4.393 | 4.426 | 4.389 | 4.423 | 4.4 | 4.454 | 4.448 | 4.472 | 4.492 | 4.42 | 4.431 |
| *Pp* P[4] alone | 11 | 14 | IGIT | 3.88 | 1 | 2 | 1.676 | 1.668 | 1.692 | 1.881 | 1.894 | 1.903 | 1.908 | 1.861 | 1.936 | 2.002 | 1.958 | 1.945 | 1.908 | 1.932 | 2.019 | 2.111 | 2.054 | 2.041 |
| *Pp* P[4] alone | 11 | 16 | IGITEL | 7.56 | 1 | 4 | 3.543 | 3.688 | 3.611 | 3.697 | 3.663 | 3.692 | 3.696 | 3.614 | 3.637 | 3.631 | 3.656 | 3.631 | 3.686 | 3.683 | 3.661 | 3.703 | 3.649 | 3.67 |
| *Pp* P[4] alone | 11 | 23 | IGITELGSGSGVL | 8.57 | 2 | 11 | 8.727 | 8.735 | 8.456 | 8.484 | 8.552 | 8.608 | 8.593 | 8.472 | 8.445 | 8.469 | 8.599 | 8.527 | 8.563 | 8.622 | 8.584 | 8.667 | 8.529 | 8.594 |
| *Pp* P[4] alone | 11 | 37 | IGITELGSGSGVLDGPYQPTTFKPPND | 8.81 | 2 | 21 | 11.51 | 11.647 | 11.407 | 12.209 | 12.162 | 12.338 | 12.964 | 12.744 | 12.76 | 13.947 | 14.13 | 13.976 | 15.293 | 15.299 | 15.298 | 16.036 | 15.879 | 15.903 |
| *Pp* P[4] alone | 13 | 16 | ITEL | 4.88 | 1 | 2 | 1.955 | 1.761 | 1.805 | 1.785 | 1.869 | 1.816 | 1.731 | 1.778 | 1.816 | 1.774 | 1.775 | 1.738 | 1.755 | 1.898 | 1.782 | 1.827 | 1.717 | 1.781 |
| *Pp* P[4] alone | 13 | 23 | ITELGSGSGVL | 7.09 | 2 | 9 | 6.198 | 6.406 | 5.689 | 6.067 | 6.145 | 6.047 | 6.49 | 6.019 | 5.901 | 5.969 | 6.175 | 6.186 | 6.241 | 6.241 | 6.163 | 6.447 | 6.187 | 6.089 |
| *Pp* P[4] alone | 13 | 37 | ITELGSGSGVLDGPYQPTTFKPPND | 8.06 | 2 | 19 | 9.446 | 9.535 | 9.399 | 10.038 | 10.189 | 10.283 | 11.105 | 10.85 | 10.927 | 12.072 | 12.225 | 12.209 | 13.423 | 13.443 | 13.449 | 14.127 | 14.022 | 14.023 |
| *Pp* P[4] alone | 15 | 37 | ELGSGSGVLDGPYQPTTFKPPND | 7.79 | 2 | 17 | 7.394 | 7.545 | 7.276 | 8.191 | 8.089 | 8.202 | 9.4 | 9.192 | 9.257 | 10.534 | 10.644 | 10.562 | 11.692 | 11.677 | 11.65 | 12.418 | 12.238 | 12.29 |
| *Pp* P[4] alone | 16 | 37 | LGSGSGVLDGPYQPTTFKPPND | 7.58 | 3 | 16 | 6.594 | 6.85 | 6.699 | 7.402 | 7.451 | 7.492 | 8.149 | 8.03 | 8.072 | 9.178 | 9.273 | 9.278 | 10.539 | 10.564 | 10.52 | 11.223 | 11.121 | 11.126 |
| *Pp* P[4] alone | 16 | 38 | LGSGSGVLDGPYQPTTFKPPNDY | 8.21 | 2 | 17 | 6.467 | 6.742 | 6.502 | 7.432 | 7.413 | 7.462 | 8.166 | 7.906 | 7.966 | 9.063 | 9.181 | 9.126 | 10.46 | 10.489 | 10.417 | 11.138 | 11.027 | 11.089 |
| *Pp* P[4] alone | 16 | 39 | LGSGSGVLDGPYQPTTFKPPNDYW | 9.78 | 3 | 18 | 6.08 | 6.353 | 6.215 | 6.897 | 6.98 | 7.027 | 7.512 | 7.472 | 7.502 | 8.403 | 8.573 | 8.564 | 9.774 | 9.858 | 9.82 | 10.482 | 10.438 | 10.411 |
| *Pp* P[4] alone | 17 | 37 | GSGSGVLDGPYQPTTFKPPND | 7.31 | 2 | 15 | 6.115 | 6.374 | 6.173 | 6.934 | 6.992 | 7.031 | 7.802 | 7.674 | 7.67 | 8.875 | 8.996 | 8.891 | 10.301 | 10.296 | 10.261 | 11.024 | 10.854 | 10.891 |
| *Pp* P[4] alone | 17 | 39 | GSGSGVLDGPYQPTTFKPPNDYW | 9.59 | 2 | 17 | 5.77 | 5.988 | 5.848 | 6.512 | 6.682 | 6.731 | 7.205 | 7.182 | 7.257 | 8.261 | 8.425 | 8.368 | 9.565 | 9.615 | 9.589 | 10.343 | 10.218 | 10.202 |
| *Pp* P[4] alone | 24 | 37 | DGPYQPTTFKPPND | 6.11 | 2 | 8 | 2.454 | 2.553 | 2.539 | 2.774 | 2.781 | 2.821 | 3.348 | 3.326 | 3.352 | 4.424 | 4.469 | 4.507 | 5.686 | 5.709 | 5.699 | 6.338 | 6.335 | 6.314 |
| *Pp* P[4] alone | 24 | 39 | DGPYQPTTFKPPNDYW | 9.15 | 2 | 10 | 2.44 | 2.486 | 2.415 | 2.563 | 2.588 | 2.69 | 3.346 | 3.185 | 3.216 | 4.27 | 4.291 | 4.278 | 5.332 | 5.396 | 5.368 | 6.068 | 6 | 5.935 |
| *Pp* P[4] alone | 38 | 41 | YWLL | 11.07 | 1 | 2 | 0.017 | 0.015 | 0.021 | 0.025 | 0.015 | 0.01 | 0.007 | 0.005 | 0.002 | 0.011 | 0.003 | 0.005 | 0.004 | 0.002 | 0.002 | 0.003 | 0.01 | 0.004 |
| *Pp* P[4] alone | 40 | 51 | LLISSNTNGVVY | 7.62 | 1 | 10 | 3.394 | 3.572 | 3.467 | 3.694 | 3.783 | 3.874 | 3.84 | 3.852 | 3.839 | 4.045 | 4.033 | 4.049 | 4.572 | 4.575 | 4.594 | 5.088 | 5.069 | 5.053 |
| *Pp* P[4] alone | 40 | 59 | LLISSNTNGVVYESTNNNDF | 8.16 | 2 | 18 | 3.422 | 3.745 | 3.694 | 3.974 | 4.014 | 4.081 | 4.115 | 4.144 | 4.175 | 4.427 | 4.548 | 4.519 | 5.274 | 5.333 | 5.304 | 6.12 | 6.147 | 6.111 |
| *Pp* P[4] alone | 41 | 51 | LISSNTNGVVY | 6.14 | 1 | 9 | 3.39 | 3.581 | 3.435 | 3.765 | 3.845 | 3.838 | 3.938 | 3.789 | 3.909 | 3.987 | 3.962 | 4.098 | 4.41 | 4.542 | 4.561 | 4.956 | 4.911 | 5 |
| *Pp* P[4] alone | 42 | 51 | ISSNTNGVVY | 5.31 | 2 | 8 | 3.38 | 3.54 | 3.455 | 3.665 | 3.704 | 3.793 | 3.832 | 3.762 | 3.808 | 3.94 | 3.976 | 3.998 | 4.373 | 4.359 | 4.368 | 4.483 | 4.426 | 4.459 |
| *Pp* P[4] alone | 42 | 59 | ISSNTNGVVYESTNNNDF | 6.8 | 2 | 16 | 3.558 | 3.79 | 3.735 | 3.975 | 4.058 | 4.093 | 4.154 | 4.161 | 4.159 | 4.497 | 4.566 | 4.551 | 5.256 | 5.294 | 5.346 | 5.836 | 5.831 | 5.813 |
| *Pp* P[4] alone | 49 | 59 | VVYESTNNNDF | 6.04 | 1 | 9 | 0.299 | 0.281 | 0.247 | 0.34 | 0.272 | 0.278 | 0.418 | 0.4 | 0.297 | 0.691 | 0.683 | 0.695 | 1.169 | 1.269 | 1.185 | 1.717 | 1.551 | 1.686 |
| *Pp* P[4] alone | 52 | 59 | ESTNNNDF | 4.25 | 1 | 6 | 0.175 | 0.198 | 0.186 | 0.232 | 0.229 | 0.202 | 0.296 | 0.287 | 0.299 | 0.607 | 0.667 | 0.594 | 1.14 | 1.121 | 1.119 | 1.626 | 1.595 | 1.579 |
| *Pp* P[4] alone | 59 | 63 | FWTAV | 8.09 | 1 | 3 | 0.035 | 0.039 | 0.034 | 0.074 | 0.028 | 0.019 | 0.051 | 0.042 | 0.022 | 0.049 | 0.029 | 0.028 | 0.044 | 0.028 | 0.036 | 0.043 | 0.045 | 0.038 |
| *Pp* P[4] alone | 59 | 64 | FWTAVI | 9.72 | 1 | 4 | -0.019 | -0.006 | 0.09 | 0.018 | -0.049 | -0.059 | -0.056 | -0.063 | -0.047 | -0.064 | -0.062 | -0.061 | -0.051 | -0.075 | -0.093 | -0.033 | -0.065 | -0.06 |
| *Pp* P[4] alone | 60 | 63 | WTAV | 5.53 | 1 | 2 | 0.033 | 0.031 | 0.025 | 0.03 | 0.024 | 0.02 | 0.025 | 0.024 | 0.024 | 0.026 | 0.023 | 0.026 | 0.022 | 0.024 | 0.02 | 0.026 | 0.023 | 0.026 |
| *Pp* P[4] alone | 60 | 64 | WTAVI | 7.79 | 1 | 3 | 0.032 | 0.031 | 0.028 | 0.035 | 0.022 | 0.032 | 0.029 | 0.023 | 0.023 | 0.024 | 0.018 | 0.025 | 0.021 | 0.028 | 0.023 | 0.023 | 0.021 | 0.027 |
| *Pp* P[4] alone | 64 | 73 | IAVEPHVSQT | 4.06 | 2 | 7 | 1.478 | 1.541 | 1.502 | 1.927 | 1.965 | 1.982 | 2.282 | 2.272 | 2.289 | 2.439 | 2.452 | 2.454 | 2.918 | 2.902 | 2.91 | 3.361 | 3.367 | 3.366 |
| *Pp* P[4] alone | 64 | 76 | IAVEPHVSQTNRQ | 3.61 | 2 | 10 | 2.73 | 2.828 | 2.766 | 3.501 | 3.571 | 3.557 | 4.081 | 4.05 | 4.065 | 4.356 | 4.381 | 4.378 | 5.248 | 5.257 | 5.221 | 5.904 | 5.827 | 5.859 |
| *Pp* P[4] alone | 64 | 79 | IAVEPHVSQTNRQYIL | 7.03 | 2 | 13 | 2.747 | 2.805 | 2.71 | 3.825 | 3.769 | 3.691 | 4.542 | 4.379 | 4.357 | 4.78 | 4.851 | 4.772 | 5.518 | 5.533 | 5.448 | 6.095 | 6.072 | 6.096 |
| *Pp* P[4] alone | 65 | 73 | AVEPHVSQT | 3.14 | 2 | 6 | 1.458 | 1.539 | 1.523 | 1.93 | 1.978 | 2.003 | 2.299 | 2.352 | 2.283 | 2.446 | 2.491 | 2.48 | 2.974 | 2.972 | 2.973 | 3.407 | 3.46 | 3.416 |
| *Pp* P[4] alone | 65 | 76 | AVEPHVSQTNRQ | 2.9 | 3 | 9 | 2.763 | 2.866 | 2.811 | 3.565 | 3.636 | 3.656 | 4.15 | 4.137 | 4.156 | 4.435 | 4.451 | 4.476 | 5.378 | 5.358 | 5.366 | 6.009 | 6.001 | 6.017 |
| *Pp* P[4] alone | 74 | 79 | NRQYIL | 6.43 | 2 | 4 | 0.738 | 0.67 | 0.682 | 1.342 | 1.325 | 1.295 | 1.77 | 1.701 | 1.771 | 1.8 | 1.757 | 1.776 | 1.854 | 1.847 | 1.832 | 1.813 | 1.851 | 1.862 |
| *Pp* P[4] alone | 80 | 86 | FGENKQF | 5.15 | 2 | 5 | 1.273 | 1.343 | 1.295 | 1.44 | 1.447 | 1.437 | 1.452 | 1.419 | 1.429 | 1.45 | 1.46 | 1.458 | 1.626 | 1.615 | 1.601 | 2.05 | 2.034 | 2.028 |
| *Pp* P[4] alone | 80 | 97 | FGENKQFNVENNSDKWKF | 8.05 | 4 | 16 | 2.476 | 2.764 | 2.681 | 3 | 2.911 | 2.894 | 3.353 | 3.206 | 3.14 | 4.216 | 4.192 | 4.158 | 5.437 | 5.345 | 5.3 | 6.351 | 6.179 | 6.095 |
| *Pp* P[4] alone | 84 | 97 | KQFNVENNSDKWKF | 7.44 | 4 | 12 | 1.09 | 1.44 | 1.276 | 1.527 | 1.463 | 1.42 | 1.891 | 1.682 | 1.651 | 2.778 | 2.557 | 2.592 | 3.846 | 3.554 | 3.583 | 4.561 | 4.263 | 4.176 |
| *Pp* P[4] alone | 98 | 100 | FEM | 6.19 | 1 | 1 | 0.034 | 0.031 | 0.03 | 0.03 | 0.025 | 0.027 | 0.019 | 0.028 | 0.025 | 0.023 | 0.027 | 0.021 | 0.017 | 0.022 | 0.023 | 0.026 | 0.025 | 0.025 |
| *Pp* P[4] alone | 98 | 121 | FEMFKGSSQGDFSNRRTLTSSNRL | 7.15 | 3 | 22 | 7.168 | 6.938 | 6.304 | 7.535 | 7.655 | 7.786 | 7.627 | 7.838 | 7.757 | 7.924 | 8.156 | 8.252 | 8.551 | 8.783 | 8.417 | 9.138 | 8.857 | 8.59 |
| *Pp* P[4] alone | 98 | 123 | FEMFKGSSQGDFSNRRTLTSSNRLVG | 7.21 | 4 | 24 | 7.734 | 6.597 | 6.092 | 7.653 | 7.121 | 7.384 | 7.647 | 7.418 | 7.322 | 7.78 | 7.721 | 7.86 | 8.095 | 8.025 | 8.189 | 9.018 | 8.367 | 8.306 |
| *Pp* P[4] alone | 101 | 109 | FKGSSQGDF | 4.82 | 2 | 7 | 3.867 | 4.059 | 3.92 | 4.25 | 4.318 | 4.271 | 4.495 | 4.393 | 4.42 | 4.429 | 4.481 | 4.48 | 4.642 | 4.579 | 4.561 | 4.62 | 4.571 | 4.541 |
| *Pp* P[4] alone | 101 | 121 | FKGSSQGDFSNRRTLTSSNRL | 5.25 | 3 | 19 | 7.157 | 7.926 | 7.681 | 8.987 | 8.826 | 8.646 | 9.524 | 9.123 | 8.922 | 9.499 | 9.429 | 9.447 | 10.071 | 9.842 | 9.697 | 10.479 | 10.077 | 9.841 |
| *Pp* P[4] alone | 101 | 123 | FKGSSQGDFSNRRTLTSSNRLVG | 5.42 | 4 | 21 | 7.872 | 7.594 | 7.203 | 8.443 | 8.418 | 8.204 | 9.034 | 8.691 | 8.582 | 9.13 | 9.033 | 8.981 | 9.772 | 9.417 | 9.404 | 10.331 | 9.836 | 9.6 |
| *Pp* P[4] alone | 101 | 125 | FKGSSQGDFSNRRTLTSSNRLVGML | 7.73 | 4 | 23 | 6.949 | 7.361 | 7.29 | 7.702 | 7.905 | 7.981 | 8.671 | 8.338 | 8.333 | 8.19 | 8.387 | 8.411 | 8.579 | 8.609 | 8.656 | 9.224 | 9.417 | 9.034 |
| *Pp* P[4] alone | 122 | 134 | VGMLKYGGRVWTF | 9.49 | 3 | 11 | 0.875 | 1.194 | 1.092 | 1.463 | 1.326 | 1.229 | 1.485 | 1.293 | 1.21 | 1.455 | 1.335 | 1.235 | 1.814 | 1.674 | 1.566 | 2.725 | 2.589 | 2.102 |
| *Pp* P[4] alone | 122 | 143 | VGMLKYGGRVWTFHGETPRATT | 7.38 | 4 | 19 | 1.192 | 2.368 | 1.888 | 2.983 | 3.068 | 2.502 | 3.148 | 2.724 | 2.833 | 3.41 | 2.844 | 2.888 | 4.19 | 4.194 | 2.967 | 5.919 | 5.846 | 4.913 |
| *Pp* P[4] alone | 122 | 147 | VGMLKYGGRVWTFHGETPRATTDSSN | 7.33 | 4 | 23 | 2.516 | 2.719 | 2.79 | 3.203 | 2.795 | 2.931 | 3.746 | 3.256 | 3.07 | 3.764 | 3.599 | 4.018 | 4.395 | 4.048 | 4.632 | 7.207 | 5.754 | 6.183 |
| *Pp* P[4] alone | 122 | 153 | VGMLKYGGRVWTFHGETPRATTDSSNTADLNN | 7.44 | 4 | 29 | 5.364 | 5.834 | 5.582 | 6.963 | 7.105 | 6.947 | 7.89 | 7.897 | 7.734 | 8.34 | 8.425 | 8.511 | 9.839 | 9.751 | 9.751 | 12.365 | 12.27 | 10.996 |
| *Pp* P[4] alone | 122 | 155 | VGMLKYGGRVWTFHGETPRATTDSSNTADLNNIS | 7.74 | 4 | 31 | 5.794 | 6.143 | 6.067 | 7.364 | 7.476 | 7.415 | 8.607 | 8.655 | 8.499 | 9.078 | 9.181 | 9.245 | 10.486 | 10.547 | 10.555 | 13.14 | 13.094 | 12.173 |
| *Pp* P[4] alone | 124 | 134 | MLKYGGRVWTF | 9.16 | 3 | 9 | 0.865 | 1.255 | 1.108 | 1.497 | 1.36 | 1.281 | 1.509 | 1.343 | 1.195 | 1.503 | 1.398 | 1.308 | 1.787 | 1.649 | 1.579 | 2.633 | 2.531 | 2.071 |
| *Pp* P[4] alone | 124 | 151 | MLKYGGRVWTFHGETPRATTDSSNTADL | 7.45 | 4 | 25 | 3.227 | 4.152 | 3.862 | 5.122 | 4.957 | 4.826 | 6.079 | 5.768 | 5.544 | 6.629 | 6.227 | 6.372 | 8.029 | 7.5 | 7.36 | 10.871 | 9.711 | 8.715 |
| *Pp* P[4] alone | 124 | 155 | MLKYGGRVWTFHGETPRATTDSSNTADLNNIS | 7.41 | 4 | 29 | 6.033 | 6.227 | 5.964 | 7.313 | 7.506 | 7.585 | 8.545 | 8.416 | 8.449 | 10.119 | 9.394 | 9.204 | 10.676 | 10.828 | 10.612 | 13.281 | 12.974 | 12.205 |
| *Pp* P[4] alone | 125 | 134 | LKYGGRVWTF | 8.82 | 3 | 8 | 1.144 | 1.444 | 1.31 | 1.59 | 1.468 | 1.41 | 1.506 | 1.446 | 1.36 | 1.576 | 1.473 | 1.417 | 1.816 | 1.714 | 1.686 | 2.553 | 2.483 | 2.141 |
| *Pp* P[4] alone | 125 | 151 | LKYGGRVWTFHGETPRATTDSSNTADL | 7.14 | 4 | 24 | 3.82 | 4.419 | 4.196 | 5.317 | 5.319 | 5.404 | 6.353 | 6.151 | 5.941 | 6.869 | 6.712 | 6.781 | 8.16 | 7.928 | 7.913 | 10.49 | 9.957 | 9.118 |
| *Pp* P[4] alone | 125 | 153 | LKYGGRVWTFHGETPRATTDSSNTADLNN | 6.68 | 4 | 26 | 5.863 | 6.455 | 6.378 | 7.283 | 7.255 | 7.178 | 8.408 | 8.064 | 8.107 | 8.732 | 8.765 | 8.827 | 10.171 | 10.175 | 10.164 | 12.627 | 12.326 | 11.466 |
| *Pp* P[4] alone | 125 | 155 | LKYGGRVWTFHGETPRATTDSSNTADLNNIS | 7.12 | 4 | 28 | 6.273 | 6.675 | 6.381 | 7.858 | 7.997 | 7.972 | 8.924 | 9.019 | 8.764 | 9.498 | 9.607 | 9.658 | 10.875 | 11.056 | 11.039 | 13.386 | 13.437 | 12.5 |
| *Pp* P[4] alone | 126 | 155 | KYGGRVWTFHGETPRATTDSSNTADLNNIS | 6.74 | 4 | 27 | 6.36 | 6.562 | 6.4 | 7.689 | 7.845 | 7.969 | 9.041 | 9.02 | 8.981 | 9.635 | 9.84 | 9.81 | 11.108 | 11.224 | 11.267 | 13.838 | 13.58 | 12.885 |
| *Pp* P[4] alone | 135 | 151 | HGETPRATTDSSNTADL | 4.06 | 3 | 14 | 2.935 | 3.085 | 3.051 | 4.08 | 4.242 | 4.231 | 5.246 | 5.201 | 5.174 | 5.86 | 5.875 | 5.935 | 7.043 | 7.109 | 7.009 | 8.696 | 8.41 | 7.805 |
| *Pp* P[4] alone | 135 | 155 | HGETPRATTDSSNTADLNNIS | 4.73 | 3 | 18 | 4.315 | 4.737 | 4.722 | 6.126 | 6.199 | 6.412 | 7.488 | 7.524 | 7.599 | 8.393 | 8.282 | 8.436 | 9.65 | 9.684 | 9.438 | 11.374 | 11.256 | 10.597 |
| *Pp* P[4] alone | 154 | 162 | ISIIIHSEF | 8.38 | 2 | 7 | 0.139 | 0.128 | 0.14 | 0.227 | 0.238 | 0.226 | 0.475 | 0.457 | 0.466 | 1.012 | 1.024 | 1.037 | 1.574 | 1.591 | 1.584 | 2.279 | 2.299 | 2.278 |
| *Pp* P[4] alone | 156 | 161 | IIIHSE | 3.75 | 2 | 4 | 0.13 | 0.105 | 0.139 | 0.162 | 0.184 | 0.186 | 0.408 | 0.384 | 0.393 | 0.877 | 0.863 | 0.868 | 1.293 | 1.28 | 1.215 | 1.6 | 1.591 | 1.582 |
| *Pp* P[4] alone | 156 | 162 | IIIHSEF | 6.87 | 2 | 5 | 0.147 | 0.147 | 0.138 | 0.222 | 0.226 | 0.209 | 0.465 | 0.451 | 0.435 | 0.944 | 0.959 | 0.968 | 1.313 | 1.323 | 1.297 | 1.665 | 1.631 | 1.648 |
| *Pp* P[4] alone | 157 | 162 | IIHSEF | 5.54 | 2 | 4 | 0.138 | 0.122 | 0.086 | 0.209 | 0.174 | 0.183 | 0.432 | 0.401 | 0.392 | 0.858 | 0.871 | 0.879 | 1.177 | 1.178 | 1.168 | 1.357 | 1.347 | 1.356 |
| *Pp* P[4] alone | 158 | 162 | IHSEF | 6.86 | 1 | 3 | 0.226 | 0.127 | 0.199 | 0.402 | 0.306 | 0.207 | 0.581 | 0.488 | 0.419 | 0.89 | 0.91 | 0.916 | 1.123 | 1.192 | 1.164 | 1.447 | 1.485 | 1.522 |
| *Pp* P[4] alone | 163 | 170 | YIIPRSQE | 4.77 | 2 | 5 | 2.16 | 2.231 | 2.187 | 2.493 | 2.527 | 2.537 | 2.637 | 2.619 | 2.603 | 2.857 | 2.87 | 2.872 | 3.374 | 3.389 | 3.367 | 3.462 | 3.396 | 3.413 |
| *Pp* P[4] alone | 163 | 173 | YIIPRSQESKC | 4.58 | 2 | 8 | 3.383 | 3.451 | 3.289 | 4.092 | 4.133 | 4.172 | 4.092 | 4.181 | 4.171 | 4.372 | 4.445 | 4.359 | 4.856 | 4.936 | 4.985 | 4.959 | 4.823 | 4.949 |
| *Pp* P[4] alone | 163 | 175 | YIIPRSQESKCNE | 4.53 | 3 | 10 | 3.346 | 3.5 | 3.411 | 4.351 | 4.446 | 4.4 | 4.77 | 4.689 | 4.651 | 5.014 | 5.115 | 5.113 | 6.016 | 5.975 | 5.966 | 6.115 | 6.042 | 6.06 |
| *Pp* P[4] alone | 174 | 181 | NEYINNGL | 6.28 | 2 | 6 | 0.42 | 0.467 | 0.462 | 0.821 | 0.605 | 0.617 | 0.952 | 0.971 | 0.868 | 1.25 | 1.351 | 1.277 | 2.214 | 2.474 | 2.436 | 3.971 | 3.899 | 2.501 |
| *Pp* P[4] alone | 176 | 181 | YINNGL | 5.26 | 2 | 4 | 0.282 | 0.288 | 0.24 | 0.529 | 0.582 | 0.537 | 0.888 | 1.018 | 0.864 | 1.224 | 1.272 | 1.31 | 2.018 | 2.133 | 2.04 | 2.836 | 2.82 | 2.193 |
| *Pp* P[4] with Thimerosal | 1 | 6 | MQYIKA | 4.94 | 2 | 4 | 2.986 | 2.849 | 2.947 | 3.09 | 3.126 | 3.206 | 3.239 | 3.167 | 3.119 | 3.206 | 3.192 | 3.07 | 3.163 | 3.245 | 3.201 | 3.209 | 3.19 | 3.268 |
| *Pp* P[4] with Thimerosal | 1 | 10 | MQYIKANSKF | 5.85 | 3 | 8 | 5.682 | 6.025 | 6.101 | 6.02 | 5.971 | 6.065 | 5.978 | 5.939 | 5.931 | 6.037 | 6.067 | 6.085 | 6.089 | 6.088 | 6.129 | 6.021 | 5.984 | 6.025 |
| *Pp* P[4] with Thimerosal | 1 | 12 | MQYIKANSKFIG | 6.37 | 3 | 10 | 7.343 | 7.478 | 7.616 | 7.381 | 7.279 | 7.383 | 7.457 | 7.498 | 7.374 | 7.452 | 7.545 | 7.574 | 7.955 | 7.812 | 7.859 | 7.71 | 7.457 | 7.828 |
| *Pp* P[4] with Thimerosal | 3 | 10 | YIKANSKF | 4.31 | 3 | 6 | 4.385 | 4.412 | 4.507 | 4.487 | 4.398 | 4.519 | 4.442 | 4.409 | 4.384 | 4.423 | 4.419 | 4.451 | 4.466 | 4.491 | 4.511 | 4.425 | 4.367 | 4.409 |
| *Pp* P[4] with Thimerosal | 11 | 14 | IGIT | 3.88 | 1 | 2 | 1.606 | 1.727 | 1.668 | 1.886 | 1.897 | 1.913 | 1.739 | 1.799 | 1.909 | 1.933 | 1.929 | 1.908 | 1.948 | 1.866 | 1.816 | 1.987 | 1.797 | 1.937 |
| *Pp* P[4] with Thimerosal | 11 | 16 | IGITEL | 7.56 | 1 | 4 | 3.601 | 3.629 | 3.701 | 3.693 | 3.634 | 3.702 | 3.638 | 3.62 | 3.613 | 3.649 | 3.669 | 3.712 | 3.693 | 3.711 | 3.712 | 3.667 | 3.617 | 3.626 |
| *Pp* P[4] with Thimerosal | 11 | 23 | IGITELGSGSGVL | 8.57 | 2 | 11 | 8.452 | 8.579 | 8.577 | 8.504 | 8.525 | 8.624 | 8.481 | 8.5 | 8.429 | 8.515 | 8.573 | 8.608 | 8.641 | 8.65 | 8.646 | 8.604 | 8.525 | 8.531 |
| *Pp* P[4] with Thimerosal | 11 | 37 | IGITELGSGSGVLDGPYQPTTFKPPND | 8.81 | 2 | 21 | 11.31 | 11.476 | 11.673 | 12.351 | 12.252 | 12.3 | 12.876 | 12.854 | 12.843 | 14.238 | 14.356 | 14.436 | 15.455 | 15.61 | 15.678 | 15.958 | 15.9 | 15.946 |
| *Pp* P[4] with Thimerosal | 13 | 16 | ITEL | 4.88 | 1 | 2 | 1.853 | 1.842 | 1.952 | 1.841 | 1.777 | 1.841 | 1.831 | 1.927 | 1.844 | 1.716 | 1.753 | 1.705 | 1.78 | 1.973 | 1.864 | 1.769 | 1.869 | 1.771 |
| *Pp* P[4] with Thimerosal | 13 | 23 | ITELGSGSGVL | 7.09 | 2 | 9 | 5.996 | 5.146 | 6.325 | 6.26 | 6.14 | 5.947 | 6.014 | 5.822 | 5.802 | 6.227 | 6.085 | 6.111 | 6.008 | 6.284 | 6.238 | 6.146 | 6.055 | 5.91 |
| *Pp* P[4] with Thimerosal | 13 | 37 | ITELGSGSGVLDGPYQPTTFKPPND | 8.06 | 2 | 19 | 9.285 | 9.359 | 9.401 | 10.302 | 10.183 | 10.272 | 11.06 | 10.869 | 11.012 | 12.448 | 12.532 | 12.608 | 13.631 | 13.793 | 13.773 | 14.056 | 13.971 | 14.071 |
| *Pp* P[4] with Thimerosal | 15 | 37 | ELGSGSGVLDGPYQPTTFKPPND | 7.79 | 2 | 17 | 7.283 | 7.366 | 7.578 | 8.388 | 8.217 | 8.372 | 9.419 | 9.356 | 9.421 | 10.912 | 10.95 | 11.078 | 11.903 | 12.012 | 12.039 | 12.276 | 12.215 | 12.261 |
| *Pp* P[4] with Thimerosal | 16 | 37 | LGSGSGVLDGPYQPTTFKPPND | 7.58 | 3 | 16 | 6.643 | 6.685 | 6.87 | 7.666 | 7.513 | 7.59 | 8.202 | 8.153 | 8.201 | 9.568 | 9.548 | 9.692 | 10.763 | 10.874 | 10.881 | 11.213 | 11.12 | 11.152 |
| *Pp* P[4] with Thimerosal | 16 | 38 | LGSGSGVLDGPYQPTTFKPPNDY | 8.21 | 2 | 17 | 6.536 | 6.557 | 6.63 | 7.388 | 7.38 | 7.468 | 8.073 | 7.968 | 8.079 | 9.463 | 9.474 | 9.576 | 10.704 | 10.821 | 10.831 | 11.31 | 11.384 | 11.263 |
| *Pp* P[4] with Thimerosal | 16 | 39 | LGSGSGVLDGPYQPTTFKPPNDYW | 9.78 | 3 | 18 | 6.217 | 6.251 | 6.427 | 7.104 | 7.001 | 7.075 | 7.627 | 7.587 | 7.648 | 8.874 | 8.949 | 8.986 | 10.125 | 10.264 | 10.255 | 10.798 | 10.757 | 10.77 |
| *Pp* P[4] with Thimerosal | 17 | 37 | GSGSGVLDGPYQPTTFKPPND | 7.31 | 2 | 15 | 6.219 | 6.221 | 6.344 | 7.145 | 6.999 | 7.097 | 7.778 | 7.764 | 7.771 | 9.253 | 9.286 | 9.336 | 10.459 | 10.517 | 10.593 | 10.955 | 10.904 | 10.889 |
| *Pp* P[4] with Thimerosal | 17 | 39 | GSGSGVLDGPYQPTTFKPPNDYW | 9.59 | 2 | 17 | 5.836 | 5.876 | 6.042 | 6.738 | 6.642 | 6.716 | 7.392 | 7.322 | 7.364 | 8.656 | 8.683 | 8.767 | 9.815 | 9.922 | 9.977 | 10.546 | 10.561 | 10.563 |
| *Pp* P[4] with Thimerosal | 24 | 37 | DGPYQPTTFKPPND | 6.11 | 2 | 8 | 2.5 | 2.504 | 2.568 | 2.847 | 2.84 | 2.855 | 3.454 | 3.453 | 3.441 | 4.783 | 4.758 | 4.791 | 5.876 | 5.917 | 5.916 | 6.396 | 6.321 | 6.338 |
| *Pp* P[4] with Thimerosal | 24 | 39 | DGPYQPTTFKPPNDYW | 9.15 | 2 | 10 | 2.344 | 2.472 | 2.503 | 2.728 | 2.635 | 2.692 | 3.305 | 3.325 | 3.282 | 4.543 | 4.577 | 4.508 | 5.606 | 5.66 | 5.674 | 6.341 | 6.302 | 6.383 |
| *Pp* P[4] with Thimerosal | 38 | 41 | YWLL | 11.07 | 1 | 2 | 0.026 | 0.035 | 0.023 | 0.009 | 0.01 | 0.009 | -0.001 | 0.005 | 0.011 | 0.012 | 0.008 | 0.003 | 0.01 | 0.013 | 0.011 | 0.035 | 0.04 | 0.041 |
| *Pp* P[4] with Thimerosal | 40 | 51 | LLISSNTNGVVY | 7.62 | 1 | 10 | 3.503 | 3.495 | 3.512 | 3.845 | 3.817 | 3.867 | 3.896 | 3.881 | 3.868 | 4.189 | 4.203 | 4.205 | 4.726 | 4.843 | 4.809 | 5.273 | 5.258 | 5.295 |
| *Pp* P[4] with Thimerosal | 40 | 59 | LLISSNTNGVVYESTNNNDF | 8.16 | 2 | 18 | 3.664 | 3.714 | 3.731 | 4.064 | 4.069 | 4.092 | 4.193 | 4.184 | 4.201 | 4.681 | 4.695 | 4.698 | 5.59 | 5.661 | 5.662 | 6.503 | 6.429 | 6.504 |
| *Pp* P[4] with Thimerosal | 41 | 51 | LISSNTNGVVY | 6.14 | 1 | 9 | 3.553 | 3.45 | 3.504 | 3.964 | 3.797 | 3.845 | 3.899 | 3.879 | 3.879 | 4.214 | 4.172 | 4.141 | 4.688 | 4.771 | 4.748 | 5.165 | 5.022 | 5.12 |
| *Pp* P[4] with Thimerosal | 42 | 51 | ISSNTNGVVY | 5.31 | 2 | 8 | 3.417 | 3.393 | 3.404 | 3.724 | 3.748 | 3.784 | 3.821 | 3.792 | 3.798 | 4.026 | 4.006 | 4.063 | 4.405 | 4.464 | 4.438 | 4.493 | 4.455 | 4.445 |
| *Pp* P[4] with Thimerosal | 42 | 59 | ISSNTNGVVYESTNNNDF | 6.8 | 2 | 16 | 3.723 | 3.751 | 3.809 | 4.084 | 4.069 | 4.123 | 4.18 | 4.206 | 4.206 | 4.662 | 4.698 | 4.71 | 5.441 | 5.509 | 5.517 | 6.034 | 5.997 | 6.018 |
| *Pp* P[4] with Thimerosal | 49 | 59 | VVYESTNNNDF | 6.04 | 1 | 9 | 0.306 | 0.178 | 0.486 | 0.371 | 0.241 | 0.352 | 0.455 | 0.413 | 0.446 | 0.745 | 0.646 | 0.821 | 1.222 | 1.354 | 1.264 | 1.848 | 1.828 | 1.88 |
| *Pp* P[4] with Thimerosal | 52 | 59 | ESTNNNDF | 4.25 | 1 | 6 | 0.217 | 0.167 | 0.238 | 0.225 | 0.223 | 0.211 | 0.343 | 0.341 | 0.324 | 0.679 | 0.683 | 0.683 | 1.201 | 1.246 | 1.218 | 1.776 | 1.749 | 1.74 |
| *Pp* P[4] with Thimerosal | 59 | 63 | FWTAV | 8.09 | 1 | 3 | 0.026 | 0.044 | 0.038 | 0.052 | 0.025 | 0.027 | 0.021 | 0.032 | 0.021 | 0.039 | 0.022 | 0.032 | 0.031 | 0.051 | 0.043 | 0.036 | 0.042 | 0.047 |
| *Pp* P[4] with Thimerosal | 59 | 64 | FWTAVI | 9.72 | 1 | 4 | -0.006 | 0.001 | -0.011 | -0.083 | -0.092 | -0.061 | -0.052 | -0.077 | -0.082 | -0.081 | -0.075 | -0.062 | -0.061 | -0.072 | -0.035 | -0.067 | -0.064 | -0.036 |
| *Pp* P[4] with Thimerosal | 60 | 63 | WTAV | 5.53 | 1 | 2 | 0.028 | 0.031 | 0.034 | 0.029 | 0.023 | 0.022 | 0.024 | 0.026 | 0.021 | 0.03 | 0.027 | 0.027 | 0.027 | 0.026 | 0.025 | 0.032 | 0.031 | 0.029 |
| *Pp* P[4] with Thimerosal | 60 | 64 | WTAVI | 7.79 | 1 | 3 | 0.035 | 0.034 | 0.035 | 0.027 | 0.031 | 0.021 | 0.022 | 0.022 | 0.018 | 0.023 | 0.019 | 0.026 | 0.026 | 0.027 | 0.03 | 0.03 | 0.036 | 0.043 |

| | | | | | | | | | | | | | | | | | | | | | | | | |
|---|---|---|---|---|---|---|---|---|---|---|---|---|---|---|---|---|---|---|---|---|---|---|---|---|
| *Pp* P[4] with Thimerosal | 64 | 73 | IAVEPHVSQT | 4.06 | 2 | 7 | 1.491 | 1.503 | 1.503 | 1.971 | 1.955 | 1.958 | 2.304 | 2.269 | 2.298 | 2.475 | 2.454 | 2.465 | 2.897 | 2.93 | 2.914 | 3.371 | 3.336 | 3.357 |
| *Pp* P[4] with Thimerosal | 64 | 76 | IAVEPHVSQTNRQ | 3.61 | 2 | 10 | 2.725 | 2.729 | 2.869 | 3.595 | 3.482 | 3.53 | 4.131 | 4.081 | 4.058 | 4.416 | 4.436 | 4.453 | 5.234 | 5.36 | 5.348 | 5.882 | 5.804 | 5.789 |
| *Pp* P[4] with Thimerosal | 64 | 79 | IAVEPHVSQTNRQYIL | 7.03 | 2 | 13 | 2.81 | 2.882 | 3.04 | 3.861 | 3.738 | 3.742 | 4.511 | 4.477 | 4.478 | 4.882 | 4.886 | 4.928 | 5.577 | 5.737 | 5.697 | 6.037 | 5.974 | 6.044 |
| *Pp* P[4] with Thimerosal | 65 | 73 | AVEPHVSQT | 3.14 | 2 | 6 | 1.522 | 1.466 | 1.531 | 2.054 | 1.946 | 2.013 | 2.326 | 2.294 | 2.298 | 2.504 | 2.463 | 2.508 | 2.927 | 2.922 | 2.96 | 3.439 | 3.368 | 3.391 |
| *Pp* P[4] with Thimerosal | 65 | 76 | AVEPHVSQTNRQ | 2.9 | 3 | 9 | 2.772 | 2.755 | 2.848 | 3.624 | 3.582 | 3.623 | 4.197 | 4.174 | 4.151 | 4.514 | 4.522 | 4.497 | 5.343 | 5.467 | 5.439 | 6.013 | 5.948 | 5.902 |
| *Pp* P[4] with Thimerosal | 74 | 79 | NRQYIL | 6.43 | 2 | 4 | 0.7 | 0.738 | 0.715 | 1.269 | 1.282 | 1.306 | 1.789 | 1.791 | 1.77 | 1.816 | 1.834 | 1.786 | 1.884 | 1.911 | 1.871 | 1.874 | 1.809 | 1.865 |
| *Pp* P[4] with Thimerosal | 80 | 86 | FGENKQF | 5.15 | 2 | 5 | 1.302 | 1.288 | 1.345 | 1.464 | 1.421 | 1.447 | 1.461 | 1.421 | 1.431 | 1.471 | 1.488 | 1.491 | 1.623 | 1.662 | 1.667 | 2.079 | 2.052 | 2.059 |
| *Pp* P[4] with Thimerosal | 80 | 97 | FGENKQFNVENNSDKWKF | 8.05 | 4 | 16 | 2.653 | 2.583 | 2.804 | 2.952 | 2.886 | 2.835 | 3.206 | 3.174 | 3.151 | 4.179 | 4.193 | 4.225 | 5.271 | 5.403 | 5.399 | 6.168 | 6.125 | 6.188 |
| *Pp* P[4] with Thimerosal | 84 | 97 | KQFNVENNSDKWKF | 7.44 | 4 | 12 | 1.338 | 1.278 | 1.45 | 1.497 | 1.43 | 1.322 | 1.689 | 1.667 | 1.614 | 2.567 | 2.628 | 2.554 | 3.51 | 3.602 | 3.687 | 4.284 | 4.184 | 4.249 |
| *Pp* P[4] with Thimerosal | 98 | 100 | FEM | 6.19 | 1 | 1 | 0.029 | 0.036 | 0.03 | 0.027 | 0.02 | 0.027 | 0.022 | 0.022 | 0.016 | 0.02 | 0.023 | 0.025 | 0.024 | 0.028 | 0.034 | 0.036 | 0.035 | 0.037 |
| *Pp* P[4] with Thimerosal | 98 | 121 | FEMFKGSSQGDFSNRRTLTSSNRL | 7.15 | 3 | 22 | 6.414 | 6.41 | 7.052 | 7.397 | 7.427 | 8.053 | 7.925 | 7.672 | 7.797 | 8.466 | 8.643 | 8.485 | 9.053 | 9.426 | 9.286 | 9.834 | 9.833 | 10.147 |
| *Pp* P[4] with Thimerosal | 98 | 123 | FEMFKGSSQGDFSNRRTLTSSNRLVG | 7.21 | 4 | 24 | 6.408 | 6.878 | 7.559 | 7.459 | 7.23 | 7.288 | 7.819 | 7.496 | 7.745 | 8.271 | 8.125 | 8.13 | 8.409 | 8.858 | 9.258 | 9.609 | 9.577 | 10.332 |
| *Pp* P[4] with Thimerosal | 101 | 109 | FKGSSQGDF | 4.82 | 2 | 7 | 3.901 | 3.81 | 3.925 | 4.3 | 4.192 | 4.254 | 4.426 | 4.363 | 4.415 | 4.476 | 4.479 | 4.472 | 4.51 | 4.573 | 4.562 | 4.514 | 4.43 | 4.478 |
| *Pp* P[4] with Thimerosal | 101 | 121 | FKGSSQGDFSNRRTLTSSNRL | 5.25 | 3 | 19 | 7.681 | 7.655 | 8.171 | 8.799 | 8.653 | 8.679 | 9.224 | 8.998 | 9.025 | 9.689 | 9.681 | 10.024 | 10.061 | 10.7 | 10.66 | 11.265 | 11.017 | 11.362 |
| *Pp* P[4] with Thimerosal | 101 | 123 | FKGSSQGDFSNRRTLTSSNRLVG | 5.42 | 4 | 21 | 7.241 | 6.916 | 7.714 | 8.32 | 8.279 | 8.088 | 8.81 | 8.555 | 8.564 | 9.363 | 9.279 | 9.258 | 9.609 | 10.083 | 10.221 | 11.533 | 11.26 | 11.375 |
| *Pp* P[4] with Thimerosal | 101 | 125 | FKGSSQGDFSNRRTLTSSNRLVGML | 7.73 | 4 | 23 | 7.331 | 7.123 | 7.491 | 8.439 | 8.28 | 7.844 | 8.528 | 8.149 | 8.45 | 8.864 | 8.942 | 9.064 | 9.466 | 9.683 | 10.145 | 11.283 | 11.353 | 11.78 |
| *Pp* P[4] with Thimerosal | 122 | 134 | VGMLKYGGRVWTF | 9.49 | 3 | 11 | 1.036 | 0.833 | 1.083 | 1.185 | 1.205 | 1.119 | 1.209 | 1.186 | 1.248 | 1.412 | 1.399 | 1.313 | 2.129 | 2.224 | 2.292 | 4.265 | 4.386 | 4.497 |
| *Pp* P[4] with Thimerosal | 122 | 143 | VGMLKYGGRVWTFHGETPRATT | 7.38 | 4 | 19 | 1.849 | 1.374 | 2.635 | 2.505 | 2.442 | 2.493 | 2.966 | 3.182 | 2.652 | 3.249 | 3.164 | 3.815 | 4.681 | 5.603 | 5.639 | 7.9 | 7.671 | 7.715 |
| *Pp* P[4] with Thimerosal | 122 | 147 | VGMLKYGGRVWTFHGETPRATTDSSN | 7.33 | 4 | 23 | 2.766 | 2.197 | 2.561 | 3.453 | 2.753 | 2.931 | 3.39 | 3.29 | 3.675 | 4.834 | 4.546 | 4.42 | 7.326 | 8.037 | 7.372 | 10.175 | 9.856 | 10.15 |
| *Pp* P[4] with Thimerosal | 122 | 153 | VGMLKYGGRVWTFHGETPRATTDSSNTADLNN | 7.44 | 4 | 29 | 5.897 | 5.63 | 6.012 | 7.3 | 7.211 | 6.873 | 8.056 | 8.063 | 8.142 | 9.4 | 9.539 | 10.011 | 11.996 | 12.855 | 12.646 | 15.173 | 14.987 | 15.292 |
| *Pp* P[4] with Thimerosal | 122 | 155 | VGMLKYGGRVWTFHGETPRATTDSSNTADLNNIS | 7.74 | 4 | 31 | 6.069 | 6.163 | 6.241 | 7.747 | 7.731 | 7.743 | 8.89 | 8.953 | 8.9 | 10.291 | 10.379 | 10.516 | 13.463 | 13.511 | 13.254 | 16.131 | 16.411 | 16.717 |
| *Pp* P[4] with Thimerosal | 124 | 134 | MLKYGGRVWTF | 9.16 | 3 | 9 | 1.075 | 0.835 | 1.026 | 1.21 | 1.197 | 1.104 | 1.256 | 1.187 | 1.199 | 1.415 | 1.339 | 1.332 | 2.013 | 2.062 | 2.047 | 3.63 | 3.64 | 3.801 |
| *Pp* P[4] with Thimerosal | 124 | 151 | MLKYGGRVWTFHGETPRATTDSSNTADL | 7.45 | 4 | 25 | 3.838 | 3.543 | 3.908 | 4.812 | 5.044 | 4.802 | 5.736 | 5.637 | 5.762 | 6.892 | 6.963 | 7.175 | 8.868 | 9.277 | 9.306 | 11.532 | 11.197 | 11.483 |
| *Pp* P[4] with Thimerosal | 124 | 155 | MLKYGGRVWTFHGETPRATTDSSNTADLNNIS | 7.41 | 4 | 29 | 6.12 | 6.146 | 6.254 | 7.665 | 7.618 | 7.562 | 8.93 | 8.897 | 8.698 | 10.188 | 10.494 | 10.344 | 13.348 | 13.503 | 13.552 | 16.207 | 15.879 | 16.275 |
| *Pp* P[4] with Thimerosal | 125 | 134 | LKYGGRVWTF | 8.82 | 3 | 8 | 1.243 | 1.032 | 1.348 | 1.385 | 1.357 | 1.215 | 1.418 | 1.356 | 1.362 | 1.581 | 1.524 | 1.458 | 2.219 | 2.242 | 2.27 | 3.745 | 3.559 | 3.783 |
| *Pp* P[4] with Thimerosal | 125 | 151 | LKYGGRVWTFHGETPRATTDSSNTADL | 7.14 | 4 | 24 | 4.349 | 4.276 | 4.601 | 5.357 | 5.434 | 5.174 | 6.278 | 6.259 | 6.279 | 7.615 | 7.705 | 7.689 | 9.552 | 9.878 | 10.078 | 12.467 | 12.052 | 12.484 |
| *Pp* P[4] with Thimerosal | 125 | 153 | LKYGGRVWTFHGETPRATTDSSNTADLNN | 6.68 | 4 | 26 | 6.26 | 6.744 | 5.995 | 7.521 | 7.318 | 7.23 | 8.402 | 8.275 | 8.375 | 9.932 | 9.878 | 10.18 | 12.34 | 12.516 | 12.692 | 15.16 | 14.694 | 15.192 |
| *Pp* P[4] with Thimerosal | 125 | 155 | LKYGGRVWTFHGETPRATTDSSNTADLNNIS | 7.12 | 4 | 28 | 6.376 | 6.737 | 6.627 | 8.284 | 8.148 | 8.209 | 9.447 | 9.227 | 9.249 | 10.934 | 10.972 | 11.095 | 13.514 | 13.754 | 13.828 | 16.276 | 16.211 | 16.342 |
| *Pp* P[4] with Thimerosal | 126 | 155 | KYGGRVWTFHGETPRATTDSSNTADLNNIS | 6.74 | 4 | 27 | 6.486 | 6.51 | 6.62 | 8.139 | 8.1 | 8.152 | 9.427 | 9.371 | 9.369 | 10.949 | 11.029 | 11.079 | 13.667 | 13.858 | 14.04 | 16.338 | 16.107 | 16.492 |
| *Pp* P[4] with Thimerosal | 135 | 151 | HGETPRATTDSSNTADL | 4.06 | 3 | 14 | 3.101 | 3.055 | 3.288 | 4.407 | 4.369 | 4.417 | 5.526 | 5.522 | 5.582 | 6.761 | 6.76 | 6.672 | 8.72 | 8.933 | 8.905 | 9.816 | 9.69 | 9.826 |
| *Pp* P[4] with Thimerosal | 135 | 155 | HGETPRATTDSSNTADLNNIS | 4.73 | 3 | 18 | 4.664 | 4.781 | 4.887 | 6.604 | 6.456 | 6.537 | 8.028 | 7.984 | 7.916 | 9.359 | 9.284 | 9.27 | 11.368 | 11.38 | 11.542 | 12.574 | 12.481 | 12.645 |
| *Pp* P[4] with Thimerosal | 154 | 162 | ISIIIHSEF | 8.38 | 2 | 7 | 0.138 | 0.139 | 0.157 | 0.237 | 0.225 | 0.211 | 0.514 | 0.516 | 0.506 | 1.132 | 1.126 | 1.142 | 1.701 | 1.74 | 1.759 | 2.469 | 2.46 | 2.488 |
| *Pp* P[4] with Thimerosal | 156 | 161 | IIIHSE | 3.75 | 2 | 4 | 0.111 | 0.119 | 0.142 | 0.208 | 0.188 | 0.206 | 0.446 | 0.43 | 0.45 | 0.941 | 0.916 | 0.943 | 1.28 | 1.355 | 1.346 | 1.664 | 1.618 | 1.618 |
| *Pp* P[4] with Thimerosal | 156 | 162 | IIIHSEF | 6.87 | 2 | 5 | 0.129 | 0.146 | 0.147 | 0.231 | 0.21 | 0.216 | 0.493 | 0.476 | 0.474 | 1.014 | 1.025 | 1.031 | 1.339 | 1.391 | 1.392 | 1.711 | 1.691 | 1.71 |
| *Pp* P[4] with Thimerosal | 157 | 162 | IIHSEF | 5.54 | 2 | 4 | 0.107 | 0.122 | 0.163 | 0.204 | 0.173 | 0.189 | 0.458 | 0.439 | 0.453 | 0.935 | 0.935 | 0.976 | 1.2 | 1.267 | 1.26 | 1.438 | 1.407 | 1.388 |
| *Pp* P[4] with Thimerosal | 158 | 162 | IHSEF | 6.86 | 1 | 3 | 0.233 | 0.19 | 0.182 | 0.255 | 0.258 | 0.264 | 0.494 | 0.51 | 0.499 | 1.03 | 1.008 | 1.019 | 1.24 | 1.25 | 1.239 | 1.525 | 1.424 | 1.464 |
| *Pp* P[4] with Thimerosal | 163 | 170 | YIIPRSQE | 4.77 | 2 | 5 | 2.298 | 2.262 | 2.386 | 2.643 | 2.614 | 2.626 | 2.936 | 2.891 | 2.931 | 3.284 | 3.286 | 3.31 | 3.385 | 3.426 | 3.432 | 3.391 | 3.39 | 3.383 |
| *Pp* P[4] with Thimerosal | 163 | 173 | YIIPRSQESKC | 4.58 | 2 | 8 | 3.561 | 3.645 | 4.026 | 4.332 | 4.25 | 4.21 | 4.486 | 4.629 | 4.485 | 4.845 | 4.809 | 4.857 | 4.806 | 4.975 | 4.978 | 4.795 | 4.821 | 4.855 |
| *Pp* P[4] with Thimerosal | 163 | 175 | YIIPRSQESKCNE | 4.53 | 3 | 10 | 4.007 | 4.079 | 4.358 | 5.096 | 5.01 | 5.043 | 5.526 | 5.509 | 5.551 | 5.917 | 5.939 | 6 | 5.996 | 6.189 | 6.188 | 6 | 5.911 | 5.931 |
| *Pp* P[4] with Thimerosal | 174 | 181 | NEYINNGL | 6.28 | 2 | 6 | 0.541 | 0.601 | 0.84 | 1.382 | 1.351 | 1.16 | 2.252 | 2.271 | 2.289 | 3.77 | 3.813 | 3.661 | 4.346 | 4.34 | 4.246 | 4.388 | 4.425 | 4.558 |
| *Pp* P[4] with Thimerosal | 176 | 181 | YINNGL | 5.26 | 2 | 4 | 0.439 | 0.435 | 0.464 | 0.987 | 1.092 | 1.104 | 1.74 | 1.77 | 1.824 | 2.653 | 2.69 | 2.675 | 2.968 | 3.012 | 2.961 | 2.955 | 2.966 | 2.982 |
| *Pp* P[4] with Thimerosal | 1 | 6 | MQYIKA | 4.94 | 2 | 4 | 3.215 | 3.126 | 3.149 | 3.186 | 3.205 | 3.266 | 3.137 | 3.114 | 3.12 | 3.247 | 3.262 | 3.27 | 3.25 | 3.243 | 3.239 | 3.263 | 3.295 | 3.244 |
| *Pp* P[4] with Thimerosal | 1 | 10 | MQYIKANSKF | 5.85 | 3 | 8 | 5.807 | 5.677 | 5.902 | 5.905 | 5.754 | 5.85 | 5.84 | 5.733 | 5.717 | 5.979 | 5.939 | 5.906 | 6.021 | 5.97 | 5.963 | 5.922 | 5.916 | 5.818 |
| *Pp* P[4] with Thimerosal | 1 | 12 | MQYIKANSKFIG | 6.37 | 3 | 10 | 7.146 | 7.043 | 7.228 | 7.128 | 7.114 | 7.18 | 7.119 | 7.091 | 7.033 | 7.179 | 7.308 | 7.302 | 7.394 | 7.312 | 7.389 | 7.326 | 7.343 | 7.297 |
| *Pp* P[4] with Thimerosal | 3 | 10 | YIKANSKF | 4.31 | 3 | 6 | 4.361 | 4.296 | 4.411 | 4.385 | 4.296 | 4.366 | 4.318 | 4.266 | 4.245 | 4.417 | 4.399 | 4.387 | 4.453 | 4.43 | 4.409 | 4.368 | 4.388 | 4.299 |
| *Pp* P[4] with Thimerosal | 11 | 14 | IGIT | 3.88 | 1 | 2 | 1.79 | 1.847 | 1.836 | 1.988 | 1.966 | 1.991 | 1.976 | 1.946 | 1.953 | 2.017 | 2.007 | 2.029 | 2.079 | 2.041 | 2.057 | 2.051 | 2.098 | 2.079 |
| *Pp* P[4] with Thimerosal | 11 | 16 | IGITEL | 7.56 | 1 | 4 | 3.581 | 3.524 | 3.633 | 3.584 | 3.536 | 3.552 | 3.541 | 3.518 | 3.486 | 3.563 | 3.576 | 3.57 | 3.614 | 3.579 | 3.614 | 3.556 | 3.572 | 3.554 |
| *Pp* P[4] with Thimerosal | 11 | 23 | IGITELGSGSGVL | 8.57 | 2 | 11 | 7.672 | 7.758 | 7.765 | 7.759 | 7.35 | 7.529 | 7.456 | 7.723 | 7.611 | 8.015 | 7.829 | 7.942 | 8.191 | 8.024 | 8.243 | 8.521 | 8.492 | 8.506 |
| *Pp* P[4] with Thimerosal | 11 | 37 | IGITELGSGSGVLDGPYQPTTFKPPND | 8.81 | 2 | 21 | 9.954 | 9.898 | 10.134 | 10.469 | 10.263 | 10.303 | 10.722 | 10.631 | 10.581 | 11.633 | 11.55 | 11.528 | 12.895 | 12.805 | 12.82 | 14.026 | 13.942 | 13.833 |
| *Pp* P[4] with Thimerosal | 13 | 16 | ITEL | 4.88 | 1 | 2 | 1.83 | 1.689 | 1.826 | 1.745 | 1.769 | 1.933 | 1.898 | 1.844 | 1.82 | 1.786 | 1.901 | 1.815 | 1.825 | 1.818 | 1.87 | 1.777 | 1.78 | 1.82 |
| *Pp* P[4] with Thimerosal | 13 | 23 | ITELGSGSGVL | 7.09 | 2 | 9 | 5.561 | 5.64 | 5.825 | 5.944 | 5.69 | 5.764 | 5.639 | 5.718 | 5.95 | 5.728 | 5.832 | 5.85 | 6.082 | 6.25 | 5.962 | 6.316 | 6.379 | 6.422 |
| *Pp* P[4] with Thimerosal | 13 | 37 | ITELGSGSGVLDGPYQPTTFKPPND | 8.06 | 2 | 19 | 8.069 | 7.972 | 8.237 | 8.507 | 8.262 | 8.366 | 8.839 | 8.781 | 8.722 | 9.68 | 9.697 | 9.673 | 10.985 | 10.881 | 10.954 | 12.208 | 12.102 | 11.973 |
| *Pp* P[4] with Thimerosal | 15 | 37 | ELGSGSGVLDGPYQPTTFKPPND | 7.79 | 2 | 17 | 6.218 | 6.169 | 6.391 | 6.702 | 6.516 | 6.565 | 7.126 | 7.094 | 6.991 | 8.247 | 8.137 | 8.062 | 9.658 | 9.535 | 9.522 | 10.673 | 10.561 | 10.441 |
| *Pp* P[4] with Thimerosal | 16 | 37 | LGSGSGVLDGPYQPTTFKPPND | 7.58 | 3 | 16 | 5.318 | 5.281 | 5.474 | 5.686 | 5.519 | 5.663 | 6.012 | 5.909 | 5.902 | 6.854 | 6.824 | 6.756 | 8.074 | 7.939 | 8.003 | 9.158 | 9.086 | 8.909 |
| *Pp* P[4] with Thimerosal | 16 | 38 | LGSGSGVLDGPYQPTTFKPPNDY | 8.21 | 2 | 17 | 4.862 | 5.707 | 5.633 | 5.978 | 5.891 | 5.938 | 6.1 | 5.921 | 6.148 | 7.277 | 7.627 | 7.681 | 8.249 | 8.058 | 8.154 | 9.639 | 10.252 | 10.012 |
| *Pp* P[4] with Thimerosal | 16 | 39 | LGSGSGVLDGPYQPTTFKPPNDYW | 9.78 | 3 | 18 | 4.969 | 4.962 | 5.086 | 5.396 | 5.177 | 5.232 | 5.658 | 5.54 | 5.502 | 6.412 | 6.339 | 6.329 | 7.52 | 7.399 | 7.36 | 8.548 | 8.43 | 8.3 |
| *Pp* P[4] with Thimerosal | 17 | 37 | GSGSGVLDGPYQPTTFKPPND | 7.31 | 2 | 15 | 4.568 | 4.472 | 4.56 | 5.051 | 4.917 | 4.938 | 5.37 | 5.221 | 5.245 | 6.217 | 6.188 | 6.128 | 7.488 | 7.434 | 7.386 | 8.664 | 8.676 | 8.448 |
| *Pp* P[4] with Thimerosal | 17 | 39 | GSGSGVLDGPYQPTTFKPPNDYW | 9.59 | 2 | 17 | 4.611 | 4.575 | 4.708 | 5.082 | 4.877 | 4.88 | 5.311 | 5.233 | 5.247 | 6.038 | 6.076 | 6.037 | 7.202 | 7.129 | 7.182 | 8.266 | 8.304 | 8.155 |
| *Pp* P[4] with Thimerosal | 24 | 37 | DGPYQPTTFKPPND | 6.11 | 2 | 8 | 2.417 | 2.418 | 2.473 | 2.507 | 2.509 | 2.581 | 2.82 | 2.774 | 2.749 | 3.507 | 3.51 | 3.505 | 4.467 | 4.42 | 4.391 | 5.039 | 5.008 | 4.982 |
| *Pp* P[4] with Thimerosal | 24 | 39 | DGPYQPTTFKPPNDYW | 9.15 | 2 | 10 | 2.374 | 2.367 | 2.369 | 2.448 | 2.385 | 2.433 | 2.619 | 2.62 | 2.598 | 3.315 | 3.295 | 3.279 | 4.142 | 4.084 | 4.153 | 4.729 | 4.703 | 4.714 |
| *Pp* P[4] with Thimerosal | 38 | 41 | YWLL | 11.07 | 1 | 2 | 0.006 | 0.01 | 0.009 | 0.003 | -0.001 | 0.004 | 0.004 | 0.004 | -0.002 | 0.004 | 0 | 0 | 0.001 | 0.002 | 0 | 0.001 | -0.002 | 0.002 |
| *Pp* P[4] with Thimerosal | 40 | 51 | LLISSNTNGVVY | 7.62 | 1 | 10 | 2.012 | 1.939 | 1.968 | 2.737 | 2.668 | 2.663 | 3.541 | 3.471 | 3.474 | 3.8 | 3.775 | 3.76 | 3.886 | 3.847 | 3.842 | 3.879 | 3.868 | 3.782 |
| *Pp* P[4] with Thimerosal | 40 | 59 | LLISSNTNGVVYESTNNNDF | 8.16 | 2 | 18 | 2.279 | 2.277 | 2.275 | 3.012 | 2.953 | 2.934 | 3.774 | 3.707 | 3.743 | 4.237 | 4.209 | 4.173 | 4.667 | 4.623 | 4.591 | 4.992 | 4.896 | 4.807 |
| *Pp* P[4] with 13A1 | 41 | 51 | LISSNTNGVVY | 6.14 | 1 | 9 | 2.108 | 2.114 | 2.103 | 2.907 | 2.912 | 2.935 | 3.635 | 3.592 | 3.622 | 3.959 | 3.927 | 3.89 | 4.031 | 3.948 | 3.904 | 3.993 | 3.934 | 3.9 |
| *Pp* P[4] with 13A1 | 42 | 51 | ISSNTNGVVY | 5.31 | 2 | 8 | 1.908 | 1.906 | 1.999 | 2.714 | 2.678 | 2.698 | 3.522 | 3.467 | 3.515 | 3.803 | 3.784 | 3.752 | 3.894 | 3.863 | 3.867 | 3.855 | 3.867 | 3.792 |
| *Pp* P[4] with 13A1 | 42 | 59 | ISSNTNGVVYESTNNNDF | 6.8 | 2 | 16 | 2.172 | 2.194 | 2.214 | 2.928 | 2.873 | 2.881 | 3.757 | 3.672 | 3.705 | 4.26 | 4.235 | 4.233 | 4.83 | 4.78 | 4.778 | 5.279 | 5.251 | 5.162 |
| *Pp* P[4] with 13A1 | 49 | 59 | VVYESTNNNDF | 6.04 | 1 | 9 | 0.388 | 0.344 | 0.514 | 0.415 | 0.353 | 0.387 | 0.564 | 0.527 | 0.512 | 0.91 | 0.775 | 0.752 | 1.358 | 1.361 | 1.407 | 1.719 | 1.617 | 1.772 |
| *Pp* P[4] with 13A1 | 52 | 59 | ESTNNNDF | 4.25 | 1 | 6 | 0.192 | 0.17 | 0.181 | 0.186 | 0.182 | 0.197 | 0.286 | 0.299 | 0.303 | 0.645 | 0.641 | 0.625 | 1.153 | 1.127 | 1.136 | 1.601 | 1.588 | 1.579 |
| *Pp* P[4] with 13A1 | 59 | 63 | FWTAV | 8.09 | 1 | 3 | 0.072 | 0.112 | 0.169 | 0.088 | 0.096 | 0.068 | 0.052 | 0.077 | 0.082 | 0.029 | 0.047 | 0.057 | 0.002 | 0.011 | 0.034 | 0.004 | 0.004 | 0.012 |
| *Pp* P[4] with 13A1 | 59 | 64 | FWTAVI | 9.72 | 1 | 4 | 0.055 | -0.007 | 0.057 | -0.002 | -0.023 | -0.012 | 0.013 | -0.004 | 0.039 | 0.022 | 0.017 | 0.009 | 0.024 | 0.034 | 0.057 | 0.025 | 0.075 | 0.076 |
| *Pp* P[4] with 13A1 | 60 | 63 | WTAV | 5.53 | 1 | 2 | 0.033 | 0.037 | 0.04 | 0.031 | 0.026 | 0.031 | 0.029 | 0.034 | 0.028 | 0.028 | 0.03 | 0.027 | 0.029 | 0.028 | 0.032 | 0.032 | 0.03 | 0.033 |
| *Pp* P[4] with 13A1 | 60 | 64 | WTAVI | 7.79 | 1 | 3 | 0.04 | 0.036 | 0.053 | 0.037 | 0.039 | 0.024 | 0.03 | 0.028 | 0.034 | 0.029 | 0.043 | 0.031 | 0.038 | 0.024 | 0.035 | 0.032 | 0.029 | 0.029 |
| *Pp* P[4] with 13A1 | 64 | 73 | IAVEPHVSQT | 4.06 | 2 | 7 | 1.48 | 1.442 | 1.5 | 1.885 | 1.855 | 1.879 | 2.209 | 2.163 | 2.179 | 2.378 | 2.379 | 2.362 | 2.742 | 2.711 | 2.714 | 3.249 | 3.254 | 3.225 |
| *Pp* P[4] with 13A1 | 64 | 76 | IAVEPHVSQTNRQ | 3.61 | 2 | 10 | 2.69 | 2.613 | 2.722 | 3.394 | 3.315 | 3.389 | 3.879 | 3.792 | 3.801 | 4.245 | 4.251 | 4.24 | 4.962 | 4.92 | 4.898 | 5.61 | 5.651 | 5.491 |
| *Pp* P[4] with 13A1 | 64 | 79 | IAVEPHVSQTNRQYIL | 7.03 | 2 | 13 | 3.415 | 2.709 | 2.867 | 3.61 | 3.497 | 3.513 | 4.083 | 4.024 | 3.993 | 4.259 | 4.066 | 4.19 | 4.626 | 4.607 | 4.596 | 5.209 | 5.117 | 5.009 |
| *Pp* P[4] with 13A1 | 65 | 73 | AVEPHVSQT | 3.14 | 2 | 6 | 1.551 | 1.545 | 1.614 | 1.973 | 2.015 | 2.007 | 2.376 | 2.289 | 2.34 | 2.504 | 2.513 | 2.518 | 2.846 | 2.842 | 2.821 | 3.375 | 3.422 | 3.374 |
| *Pp* P[4] with 13A1 | 65 | 76 | AVEPHVSQTNRQ | 2.9 | 3 | 9 | 2.759 | 2.702 | 2.818 | 3.5 | 3.467 | 3.489 | 4.015 | 3.94 | 3.94 | 4.389 | 4.372 | 4.362 | 5.119 | 5.091 | 5.075 | 5.851 | 5.872 | 5.703 |
| *Pp* P[4] with 13A1 | 74 | 79 | NRQYIL | 6.43 | 2 | 4 | 0.842 | 0.776 | 0.773 | 1.363 | 1.337 | 1.414 | 1.752 | 1.796 | 1.864 | 1.895 | 1.882 | 1.834 | 1.982 | 1.902 | 1.909 | 1.889 | 1.821 | 1.86 |
| *Pp* P[4] with 13A1 | 80 | 86 | FGENKQF | 5.15 | 2 | 5 | 1.31 | 1.269 | 1.33 | 1.381 | 1.361 | 1.369 | 1.382 | 1.35 | 1.356 | 1.441 | 1.443 | 1.436 | 1.579 | 1.559 | 1.563 | 1.942 | 1.943 | 1.887 |
| *Pp* P[4] with 13A1 | 80 | 97 | FGENKQFNVENNSDKWKF | 8.05 | 4 | 16 | 2.598 | 2.559 | 2.706 | 2.762 | 2.691 | 2.714 | 2.945 | 2.945 | 2.99 | 3.973 | 3.979 | 3.975 | 5.177 | 5.225 | 5.252 | 5.798 | 5.799 | 5.723 |
| *Pp* P[4] with 13A1 | 84 | 97 | KQFNVENNSDKWKF | 7.44 | 4 | 12 | 1.217 | 1.102 | 1.373 | 1.219 | 1.303 | 1.27 | 1.438 | 1.5 | 1.493 | 2.377 | 2.387 | 2.443 | 3.471 | 3.6 | 3.607 | 3.885 | 3.998 | 3.967 |
| *Pp* P[4] with 13A1 | 98 | 100 | FEM | 6.19 | 1 | 1 | 0.026 | 0.03 | 0.034 | 0.023 | 0.027 | 0.026 | 0.018 | 0.023 | 0.022 | 0.023 | 0.026 | 0.018 | 0.022 | 0.021 | 0.02 | 0.023 | 0.02 | 0.021 |
| *Pp* P[4] with 13A1 | 98 | 121 | FEMFKGSSQGDFSNRRTLTSSNRL | 7.15 | 3 | 22 | 5.447 | 5.295 | 6.071 | 6.077 | 6.144 | 6.405 | 6.872 | 6.425 | 6.676 | 6.98 | 7.067 | 6.898 | 7.745 | 7.951 | 8.23 | 7.55 | 7.141 | 6.853 |
| *Pp* P[4] with 13A1 | 98 | 123 | FEMFKGSSQGDFSNRRTLTSSNRLVG | 7.21 | 4 | 24 | 5.017 | 5.404 | 5.689 | 6.034 | 6.215 | 5.724 | 6.111 | 6.198 | 6.316 | 7.297 | 7.209 | 7.113 | 8.068 | 7.66 | 8.058 | 7.925 | 8.418 | 8.174 |
| *Pp* P[4] with 13A1 | 101 | 109 | FKGSSQGDF | 4.82 | 2 | 7 | 3.993 | 3.902 | 4.144 | 4.23 | 4.317 | 4.302 | 4.442 | 4.437 | 4.495 | 4.587 | 4.587 | 4.575 | 4.619 | 4.678 | 4.629 | 4.582 | 4.594 | 4.457 |
| *Pp* P[4] with 13A1 | 101 | 121 | FKGSSQGDFSNRRTLTSSNRL | 5.25 | 3 | 19 | 6.631 | 6.534 | 6.794 | 7.246 | 7.148 | 7.235 | 7.577 | 7.613 | 7.647 | 8.693 | 8.648 | 8.707 | 9.392 | 9.363 | 9.299 | 9.022 | 9.131 | 8.841 |
| *Pp* P[4] with 13A1 | 101 | 123 | FKGSSQGDFSNRRTLTSSNRLVG | 5.42 | 4 | 21 | 6.439 | 6.354 | 6.643 | 7.056 | 6.869 | 6.998 | 7.34 | 7.413 | 7.358 | 8.389 | 8.383 | 8.363 | 9.061 | 9.133 | 9.072 | 8.932 | 8.954 | 8.764 |
| *Pp* P[4] with 13A1 | 101 | 125 | FKGSSQGDFSNRRTLTSSNRLVGML | 7.73 | 4 | 23 | 6.442 | 6.76 | 6.803 | 7.482 | 7.433 | 6.83 | 7.365 | 6.853 | 7.501 | 8.141 | 8.081 | 8.279 | 8.779 | 8.796 | 8.644 | 9.021 | 8.834 | 8.354 |
| *Pp* P[4] with 13A1 | 122 | 134 | VGMLKYGGRVWTF | 9.49 | 3 | 11 | 1.389 | 1.212 | 1.44 | 1.202 | 1.347 | 1.368 | 1.18 | 1.245 | 1.269 | 1.249 | 1.345 | 1.336 | 1.497 | 1.536 | 1.549 | 2.084 | 2.059 | 2.02 |
| *Pp* P[4] with 13A1 | 122 | 143 | VGMLKYGGRVWTFHGETPRATT | 7.38 | 4 | 19 | | | | | | | | | | | | | | | | | | |
| *Pp* P[4] with 13A1 | 122 | 147 | VGMLKYGGRVWTFHGETPRATTDSSN | 7.33 | 4 | 23 | 2.333 | 1.691 | 2.308 | 2.172 | 2.428 | 2.243 | 2.603 | 3.271 | 3.669 | 3.405 | 2.992 | 3.049 | 3.988 | 4.095 | 4.105 | 5.034 | 5.373 | 5.354 |
| *Pp* P[4] with 13A1 | 122 | 153 | VGMLKYGGRVWTFHGETPRATTDSSNTADLNN | 7.44 | 4 | 29 | 5.439 | 5.476 | 5.6 | 6.29 | 6.351 | 6.232 | 7.278 | 7.223 | 6.994 | 8.03 | 7.962 | 7.866 | 9.156 | 9.079 | 8.971 | 10.392 | 10.169 | 10.116 |
| *Pp* P[4] with 13A1 | 122 | 155 | VGMLKYGGRVWTFHGETPRATTDSSNTADLNNIS | 7.74 | 4 | 31 | 4.933 | 5.092 | 5.158 | 6.468 | 5.971 | 6.008 | 6.883 | 6.861 | 6.638 | 7.871 | 7.69 | 7.647 | 9.135 | 8.491 | 8.464 | 10.166 | 9.703 | 9.726 |
| *Pp* P[4] with 13A1 | 124 | 134 | MLKYGGRVWTF | 9.16 | 3 | 9 | 1.462 | 1.306 | 1.492 | 1.302 | 1.429 | 1.399 | 1.238 | 1.328 | 1.348 | 1.305 | 1.432 | 1.406 | 1.532 | 1.633 | 1.608 | 2.064 | 2.102 | 2.02 |
| *Pp* P[4] with 13A1 | 124 | 151 | MLKYGGRVWTFHGETPRATTDSSNTADL | 7.45 | 4 | 25 | 4.214 | 4.074 | 4.408 | 4.805 | 4.958 | 4.952 | 5.472 | 5.636 | 5.755 | 6.253 | 6.46 | 6.497 | 7.222 | 7.33 | 7.273 | 8.368 | 8.376 | 8.581 |
| *Pp* P[4] with 13A1 | 124 | 155 | MLKYGGRVWTFHGETPRATTDSSNTADLNNIS | 7.41 | 4 | 29 | 5.861 | 5.919 | 5.867 | 6.882 | 6.388 | 7.384 | 7.63 | 7.578 | 7.416 | 8.704 | 8.482 | 8.429 | 9.695 | 10.342 | 9.481 | 10.932 | 10.887 | 10.343 |
| *Pp* P[4] with 13A1 | 125 | 134 | LKYGGRVWTF | 8.82 | 3 | 8 | 1.427 | 1.386 | 1.54 | 1.323 | 1.431 | 1.439 | 1.262 | 1.378 | 1.356 | 1.369 | 1.47 | 1.467 | 1.598 | 1.668 | 1.638 | 2.017 | 2.061 | 2.036 |
| *Pp* P[4] with 13A1 | 125 | 151 | LKYGGRVWTFHGETPRATTDSSNTADL | 7.14 | 4 | 24 | 4.149 | 4.068 | 4.267 | 4.976 | 4.964 | 4.949 | 5.661 | 5.666 | 5.732 | 6.424 | 6.445 | 6.409 | 7.309 | 7.286 | 7.235 | 8.361 | 8.303 | 8.126 |
| *Pp* P[4] with 13A1 | 125 | 153 | LKYGGRVWTFHGETPRATTDSSNTADLNN | 6.68 | 4 | 26 | 5.928 | 6.011 | 6.141 | 6.829 | 6.748 | 6.779 | 7.526 | 7.552 | 7.585 | 8.484 | 8.325 | 8.253 | 9.363 | 9.292 | 9.32 | 10.566 | 10.405 | 10.121 |
| *Pp* P[4] with 13A1 | 125 | 155 | LKYGGRVWTFHGETPRATTDSSNTADLNNIS | 7.12 | 4 | 28 | 6.203 | 6.193 | 6.414 | 7.969 | 7.382 | 7.539 | 8.526 | 8.319 | 8.404 | 9.386 | 9.212 | 9.258 | 10.316 | 10.144 | 10.162 | 11.694 | 11.445 | 11.294 |
| *Pp* P[4] with 13A1 | 126 | 155 | KYGGRVWTFHGETPRATTDSSNTADLNNIS | 6.74 | 4 | 27 | 5.737 | 5.755 | 6.01 | 7.447 | 7.175 | 7.054 | 8.261 | 8.073 | 8.583 | 9.341 | 9.153 | 8.991 | 9.447 | 10.113 | 10.017 | 11.772 | 11.275 | 11.236 |
| *Pp* P[4] with 13A1 | 135 | 151 | HGETPRATTDSSNTADL | 4.06 | 3 | 14 | 2.938 | 2.882 | 3.005 | 4.041 | 3.925 | 4.001 | 4.983 | 4.871 | 4.931 | 5.723 | 5.737 | 5.755 | 6.516 | 6.508 | 6.503 | 7.33 | 7.315 | 7.222 |
| *Pp* P[4] with 13A1 | 135 | 155 | HGETPRATTDSSNTADLNNIS | 4.73 | 3 | 18 | 5.476 | 5.229 | 5.92 | 6.52 | 6.628 | 6.852 | 8.085 | 7.653 | 7.988 | 8.53 | 8.556 | 8.879 | 9.35 | 9.492 | 9.39 | 10.125 | 10.682 | 11.185 |
| *Pp* P[4] with 13A1 | 154 | 162 | ISIIIHSEF | 8.38 | 2 | 7 | 0.119 | 0.121 | 0.134 | 0.188 | 0.184 | 0.187 | 0.389 | 0.379 | 0.38 | 0.701 | 0.689 | 0.692 | 1.023 | 1.007 | 1.015 | 1.633 | 1.613 | 1.578 |
| *Pp* P[4] with 13A1 | 156 | 161 | IIIHSE | 3.75 | 2 | 4 | 0.117 | 0.124 | 0.115 | 0.156 | 0.158 | 0.157 | 0.314 | 0.306 | 0.293 | 0.622 | 0.577 | 0.597 | 0.873 | 0.874 | 0.876 | 1.294 | 1.289 | 1.219 |

| | | | | | | | | | | | | | | | | | | | | | | | | |
|---|---|---|---|---|---|---|---|---|---|---|---|---|---|---|---|---|---|---|---|---|---|---|---|---|
| *Pp* | P[4] with 13A1 | 156 | 162 | IIIHSEF | 6.87 | 2 | 5 | 0.115 | 0.12 | 0.118 | 0.176 | 0.178 | 0.174 | 0.35 | 0.331 | 0.344 | 0.619 | 0.608 | 0.611 | 0.886 | 0.852 | 0.859 | 1.272 | 1.25 | 1.187 |
| *Pp* | P[4] with 13A1 | 157 | 162 | IIHSEF | 5.54 | 2 | 4 | 0.082 | 0.072 | 0.14 | 0.138 | 0.143 | 0.133 | 0.27 | 0.235 | 0.228 | 0.444 | 0.428 | 0.449 | 0.655 | 0.663 | 0.632 | 0.982 | 1.001 | 0.885 |
| *Pp* | P[4] with 13A1 | 158 | 162 | IHSEF | 6.86 | 1 | 3 | 0.173 | 0.485 | 0.358 | 0.262 | 0.414 | 0.394 | 0.476 | 0.513 | 0.719 | 0.876 | 0.787 | 0.839 | 1.012 | 1.039 | 1.083 | 1.276 | 1.276 | 1.257 |
| *Pp* | P[4] with 13A1 | 163 | 170 | YIIPRSQE | 4.77 | 2 | 5 | 1.189 | 1.225 | 1.289 | 1.446 | 1.456 | 1.426 | 1.944 | 1.902 | 1.907 | 2.618 | 2.633 | 2.622 | 3.034 | 3.065 | 3.026 | 3.41 | 3.438 | 3.389 |
| *Pp* | P[4] with 13A1 | 163 | 173 | YIIPRSQESKC | 4.58 | 2 | 8 | 1.987 | 1.942 | 2.037 | 2.509 | 2.436 | 2.5 | 3.012 | 3.153 | 3.124 | 3.849 | 3.756 | 3.781 | 4.484 | 4.444 | 4.439 | 5.023 | 4.769 | 4.801 |
| *Pp* | P[4] with 13A1 | 163 | 175 | YIIPRSQESKCNE | 4.53 | 3 | 10 | 1.672 | 1.681 | 1.743 | 2.134 | 2.036 | 2.096 | 2.79 | 2.681 | 2.684 | 3.65 | 3.599 | 3.61 | 4.623 | 4.536 | 4.501 | 5.303 | 5.257 | 5.104 |
| *Pp* | P[4] with 13A1 | 174 | 181 | NEYINNGL | 6.28 | 2 | 6 | 0.392 | 0.375 | 0.466 | 0.546 | 0.438 | 0.512 | 0.777 | 0.636 | 0.757 | 1.094 | 1.005 | 0.978 | 1.218 | 1.237 | 1.207 | 1.329 | 1.205 | 1.38 |
| *Pp* | P[4] with 13A1 | 176 | 181 | YINNGL | 5.26 | 2 | 4 | 0.166 | 0.165 | 0.187 | 0.254 | 0.225 | 0.235 | 0.406 | 0.435 | 0.444 | 0.765 | 0.729 | 0.765 | 0.93 | 0.926 | 0.921 | 1.013 | 1.009 | 1.012 |

**1-6: MQYIKA (#1)**

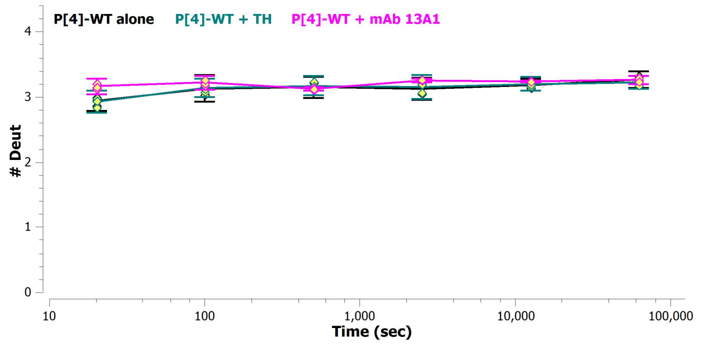


**1-10: MQYIKANSKF (#2)**

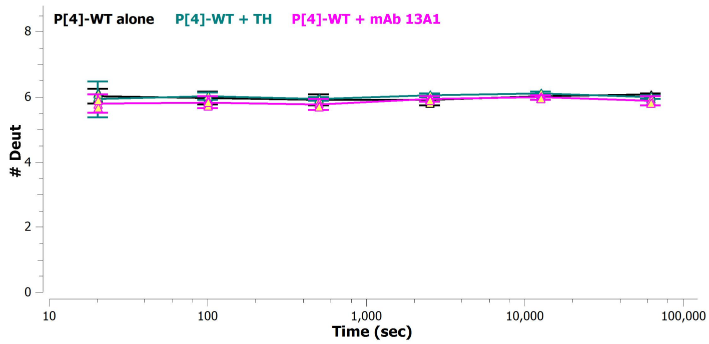


**1-12: MQYIKANSKFIG (#3)**

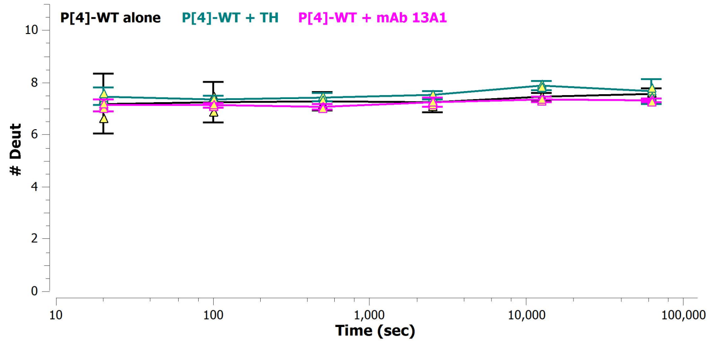


**3-10: YIKANSKF (#4)**

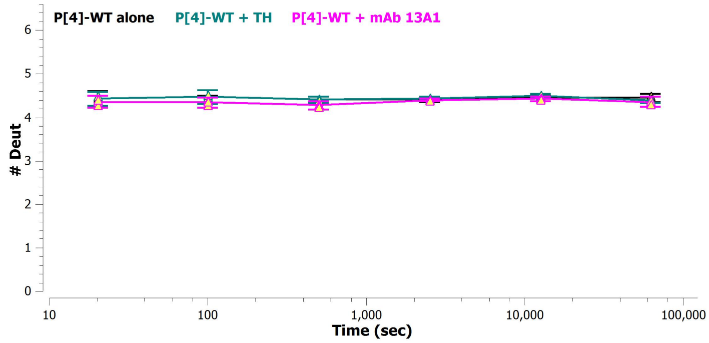


**11-14: IGIT (#5)**

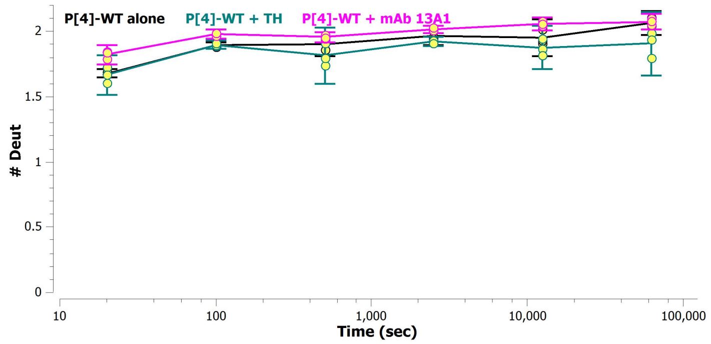


**11-16: IGITEL (#6)**

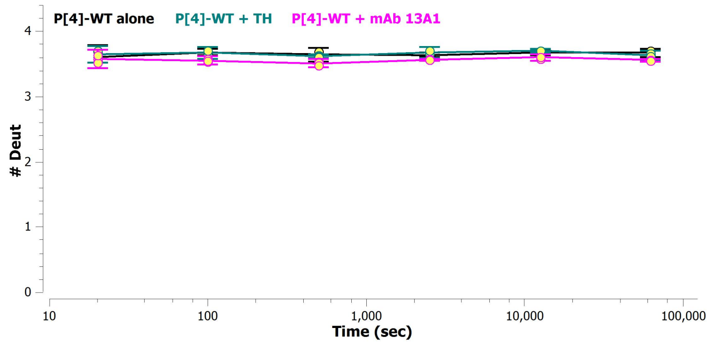


**11-23: IGITELGSGSGVL (#7)**

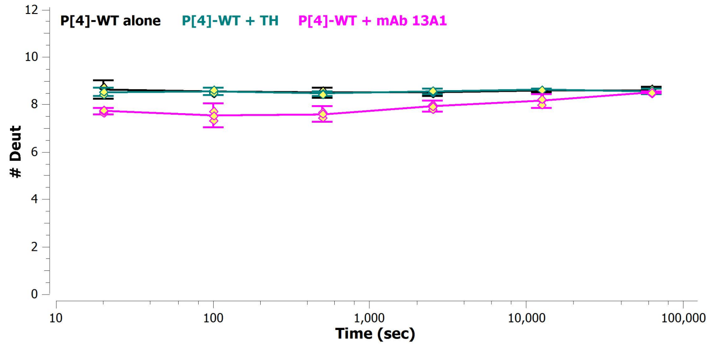


**11-37: IGITELGSGSGVLDGPYQPTTFKPPND (#8)**

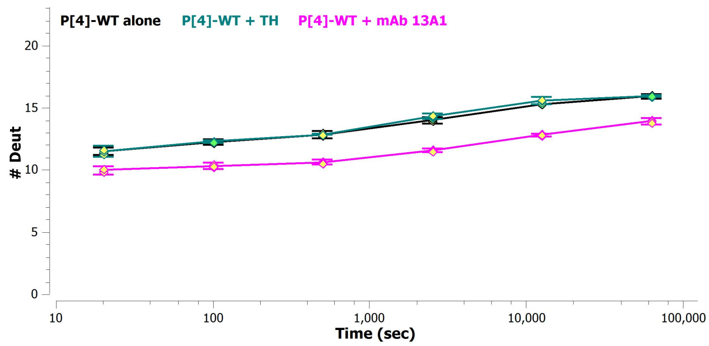


**13-16: ITEL (#9)**

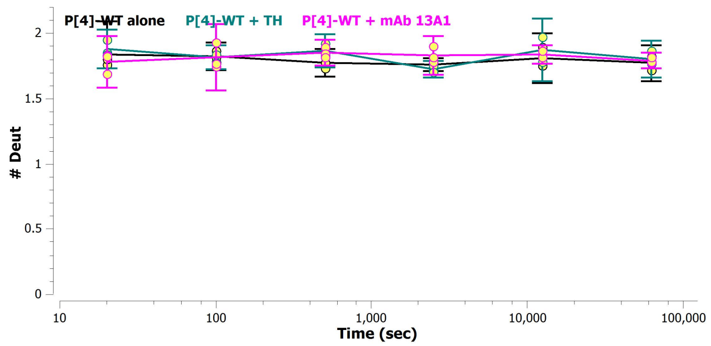


**13-23: ITELGSGSGVL (#10)**

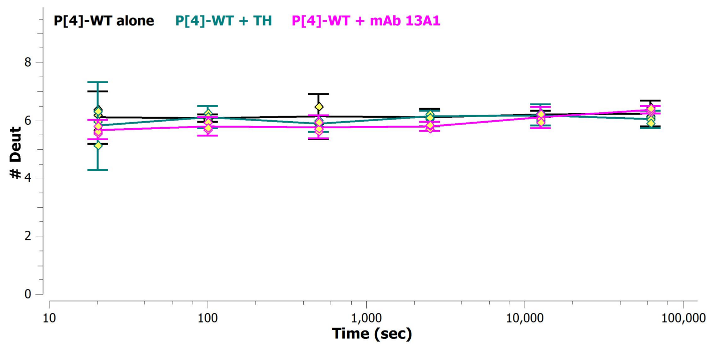

### 13-37: ITELGSGSGVLDGPYQPTTFKPPND (#11)

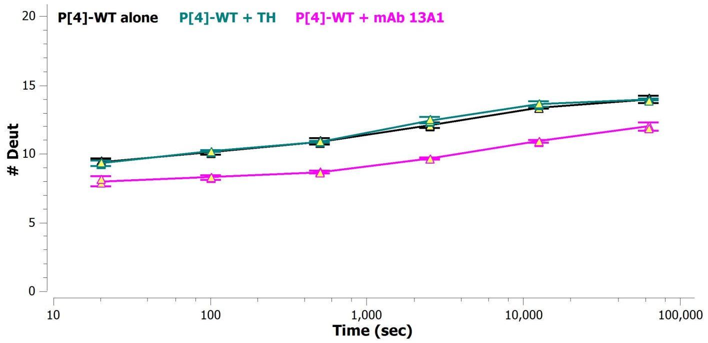


### 15-37: ELGSGSGVLDGPYQPTTFKPPND (#12)

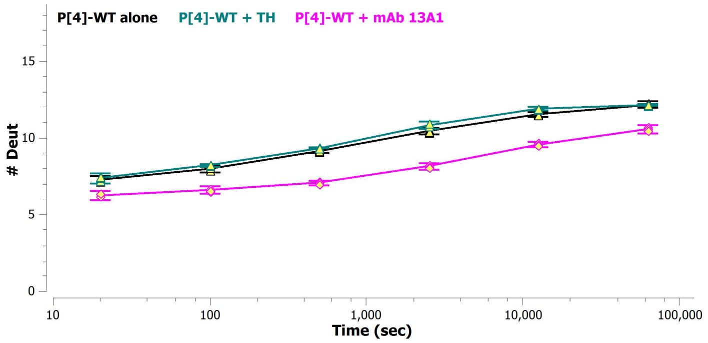


### 16-37: LGSGSGVLDGPYQPTTFKPPND (#13)

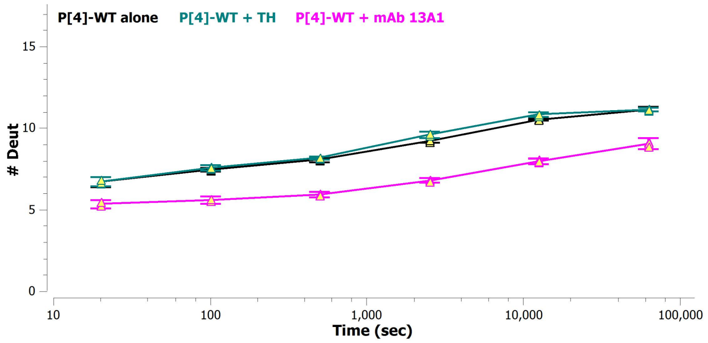


### 16-38: LGSGSGVLDGPYQPTTFKPPNDY (#14)

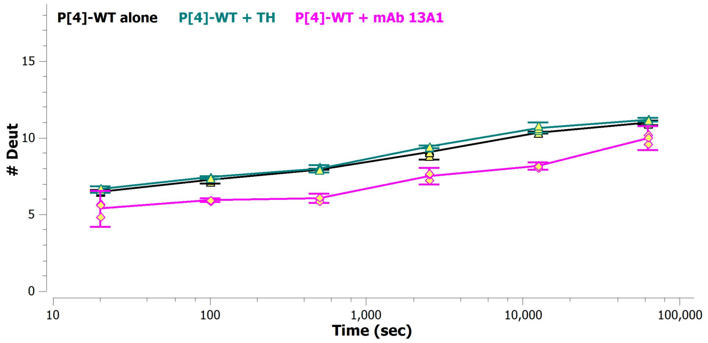


### 16-39: LGSGSGVLDGPYQPTTFKPPNDYW (#15)

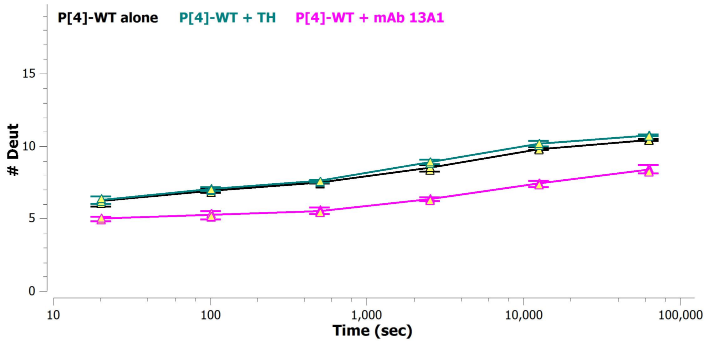


### 17-37: GSGSGVLDGPYQPTTFKPPND (#16)

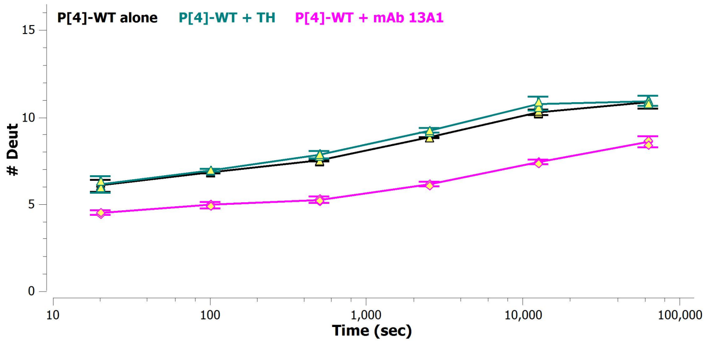


### 17-39: GSGSGVLDGPYQPTTFKPPNDYW (#17)

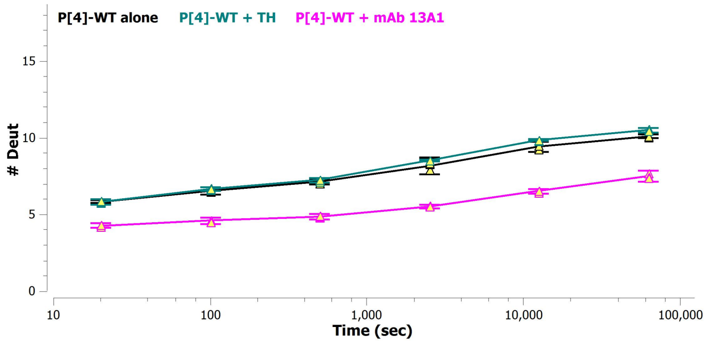


### 24-37: DGPYQPTTFKPPND (#18)

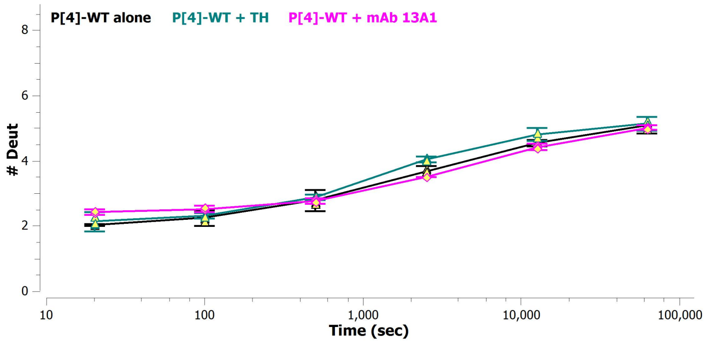


### 24-39: DGPYQPTTFKPPNDYW (#19)

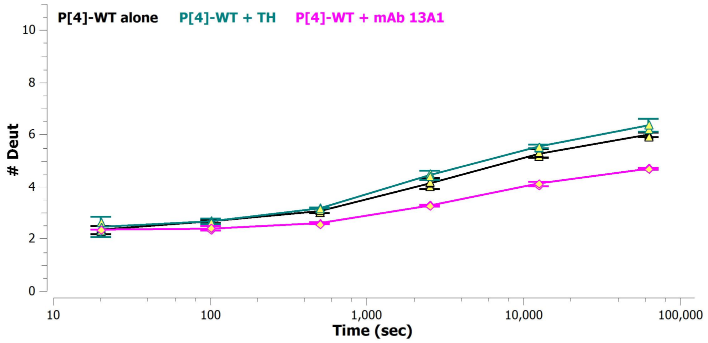


### 38-41: YWLL (#20)

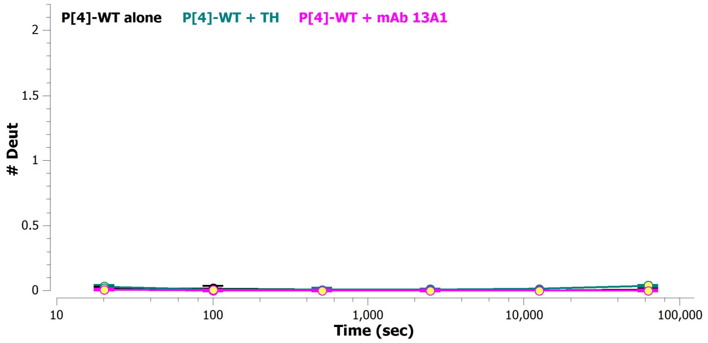

**40-51: LLISSNTNGVVY (#21)**

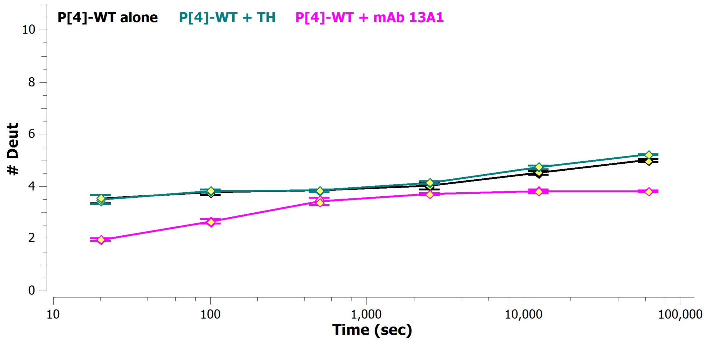


**40-59: LLISSNTNGVVYESTNNNDF (#22)**

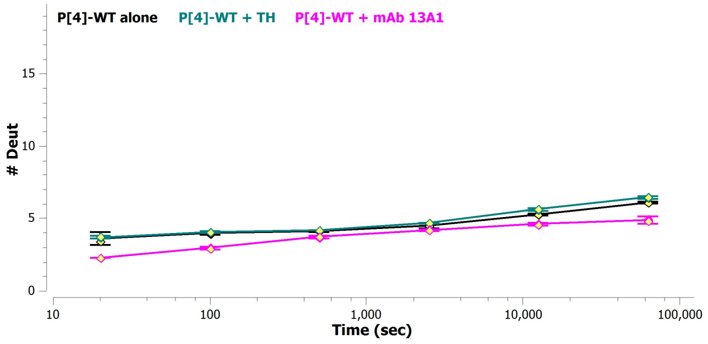


**41-51: LISSNTNGVVY (#23)**

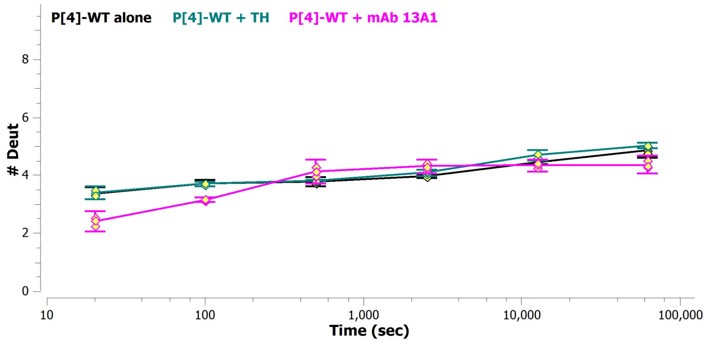


**42-51: ISSNTNGVVY (#24)**

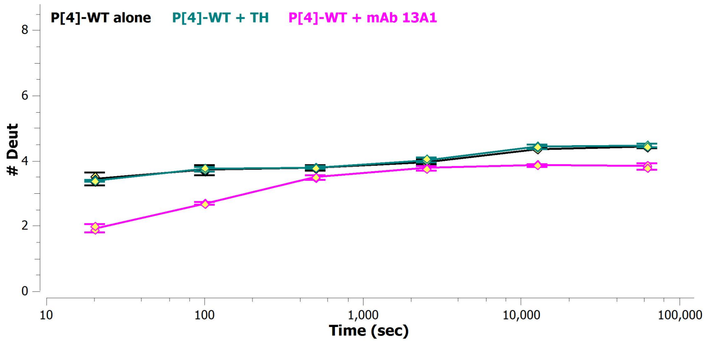


**42-59: ISSNTNGVVYESTNNNDF (#25)**

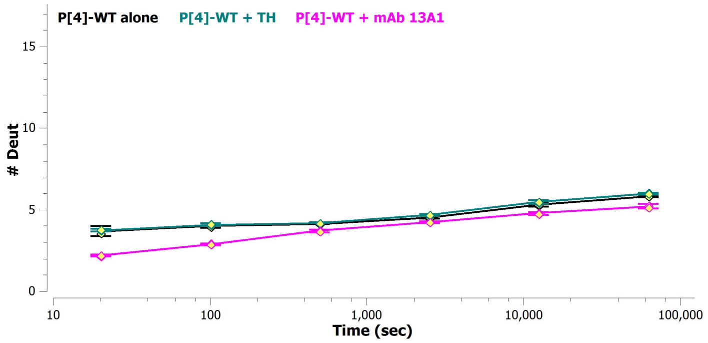


**49-59: VVYESTNNNDF (#26)**

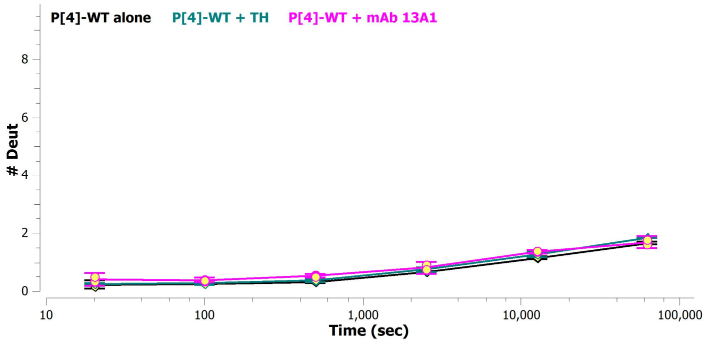


**52-59: ESTNNNDF (#27)**

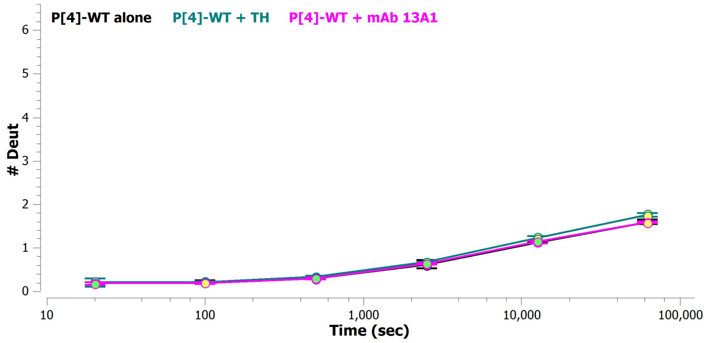


**59-63: FWTAV (#28)**

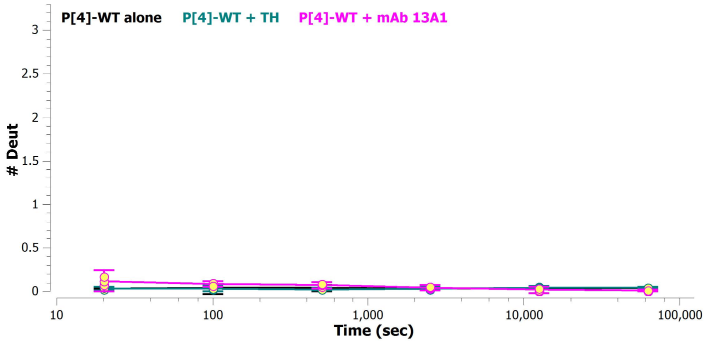


**59-64: FWTAVI (#29)**

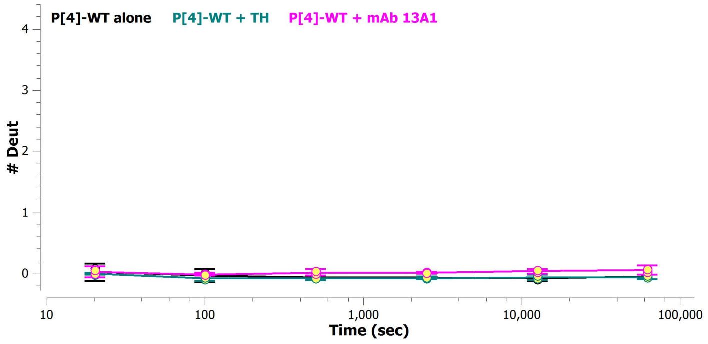


**60-63: WTAV (#30)**

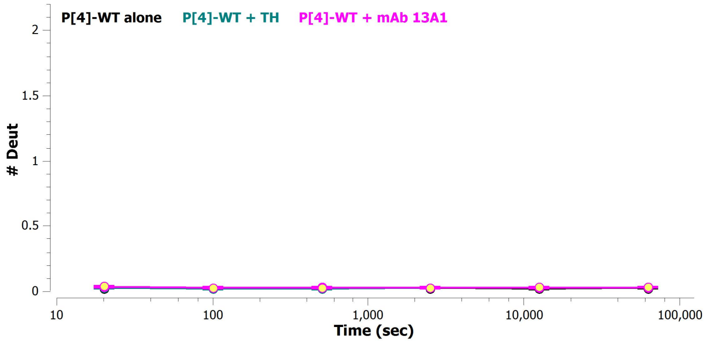

**60-64: WTAVI (#31)**

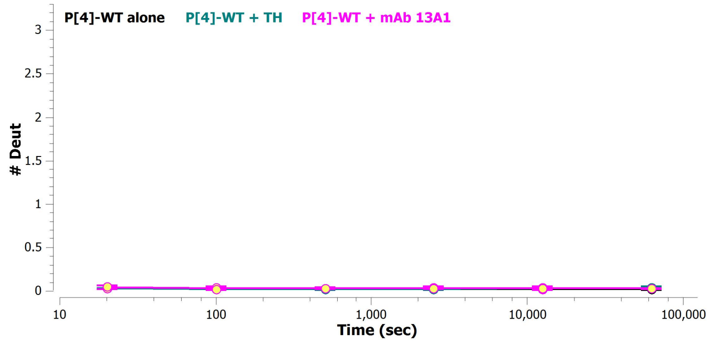


**64-73: IAVEPHVSQT (#32)**

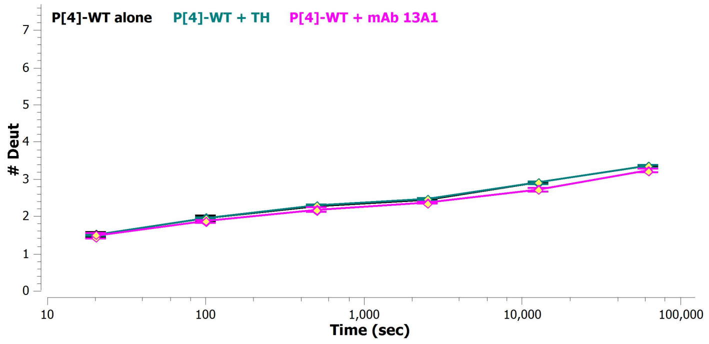


**64-76: IAVEPHVSQTNRQ (#33)**

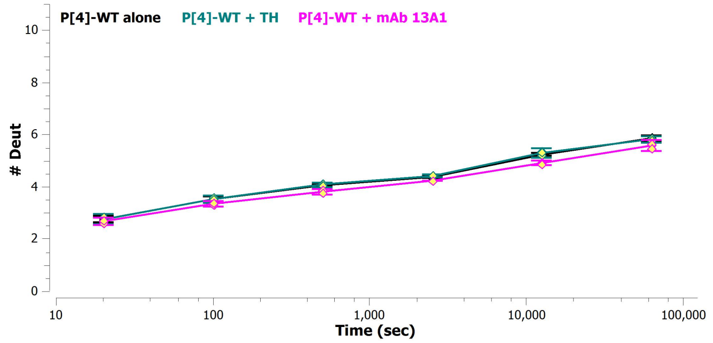


**64-79: IAVEPHVSQTNRQYIL (#34)**

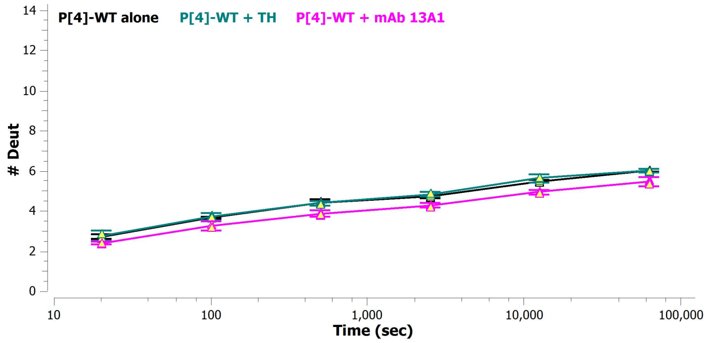


**65-73: AVEPHVSQT (#35)**

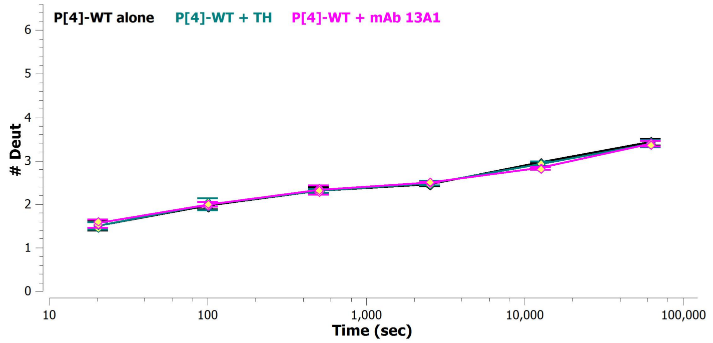


**65-76: AVEPHVSQTNRQ (#36)**

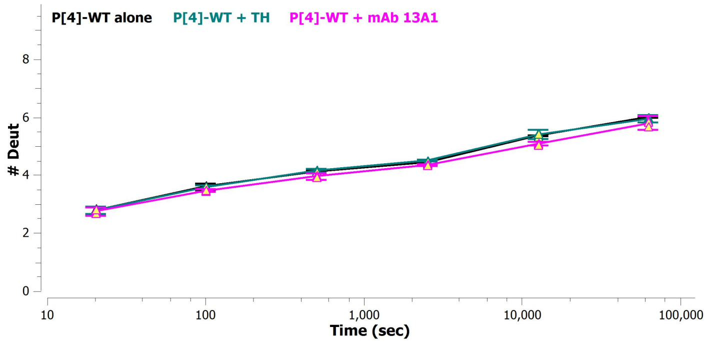


**74-79: NRQYIL (#37)**

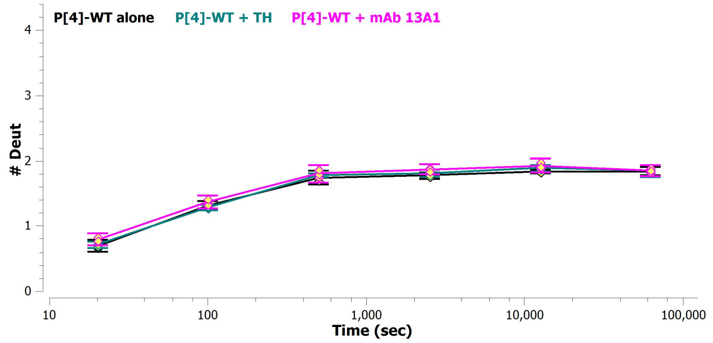


**80-86: FGENKQF (#38)**

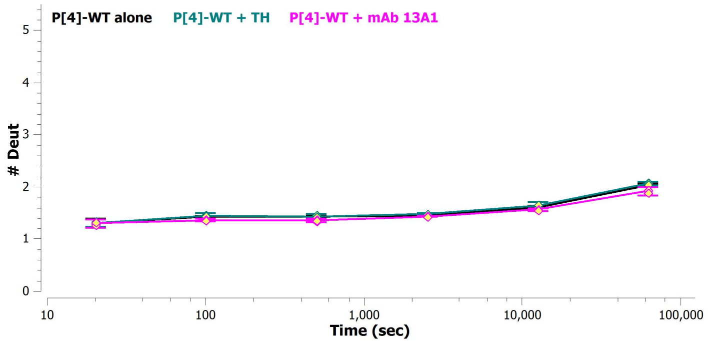


**80-97: FGENKQFNVENNSDKWKF (#39)**

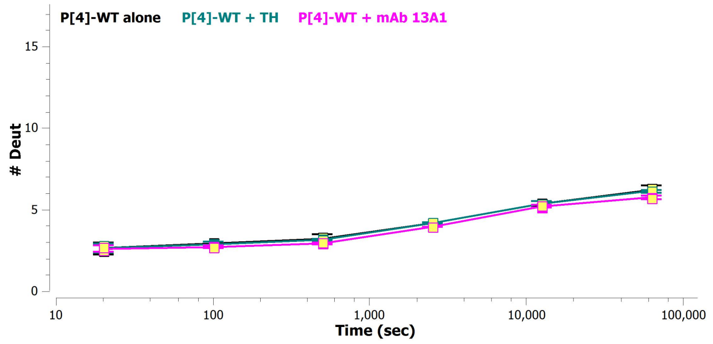


**84-97: KQFNVENNSDKWKF (#40)**

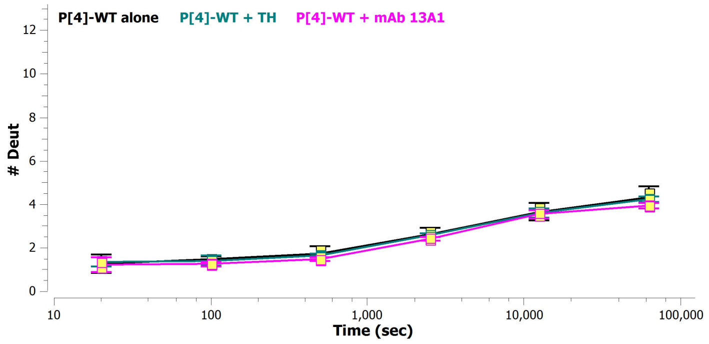

98-100: FEM (#41)

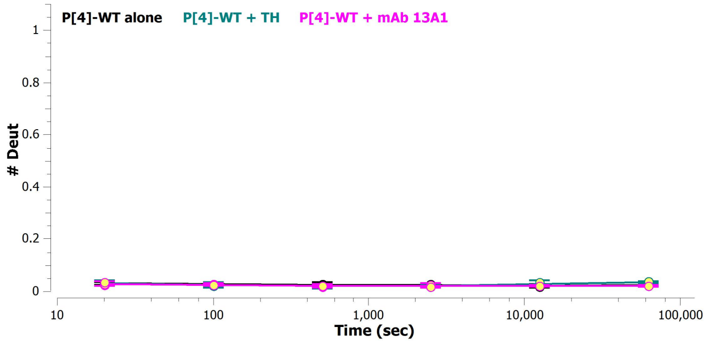


98-121: FEMFKGSSQGDFSNRRTLTSSNRL (#42)

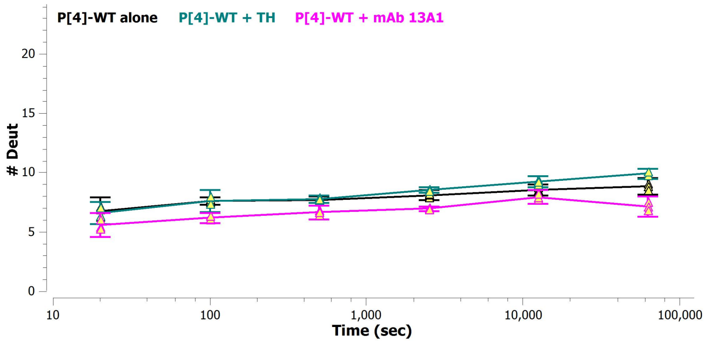


98-123: FEMFKGSSQGDFSNRRTLTSSNRLVG (#43)

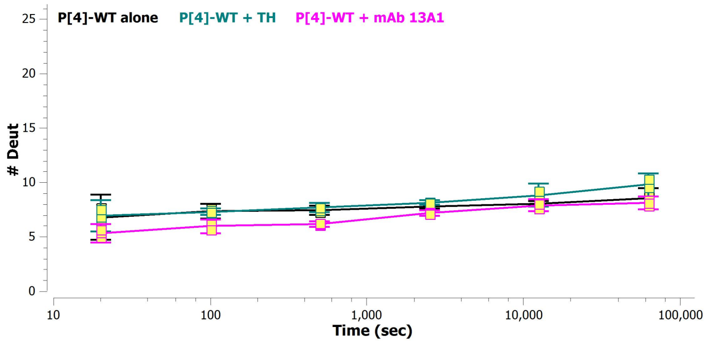


101-109: FKGSSQGDF (#44)

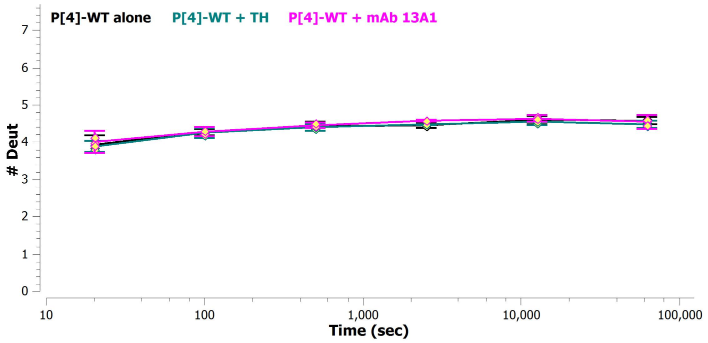


101-121: FKGSSQGDFSNRRTLTSSNRL (#45)

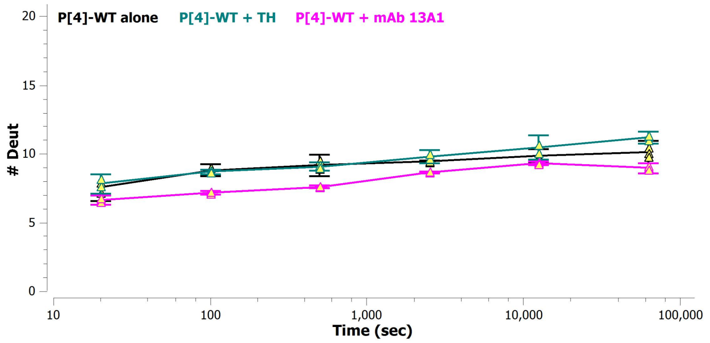


101-123: FKGSSQGDFSNRRTLTSSNRLVG (#46)

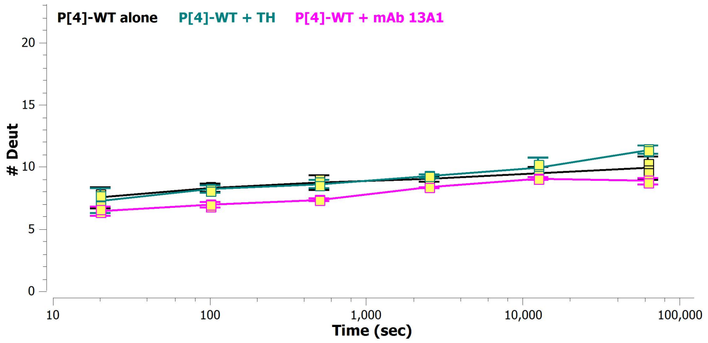


101-125: FKGSSQGDFSNRRTLTSSNRLVGML (#47)

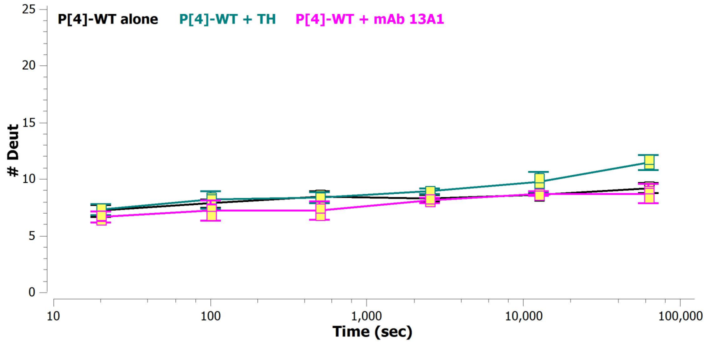


122-134: VGMLKYGGRVWTF (#48)

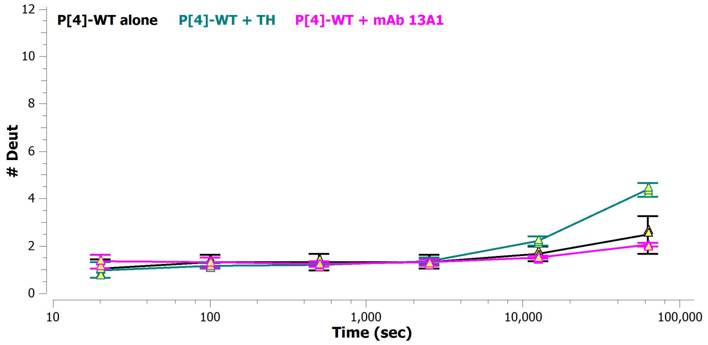


122-143: VGMLKYGGRVWTFHGETPRATT (#49)

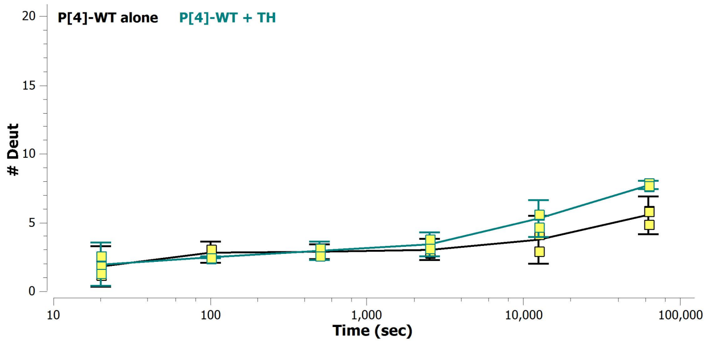


122-147: VGMLKYGGRVWTFHGETPRATTDSSN (#50)

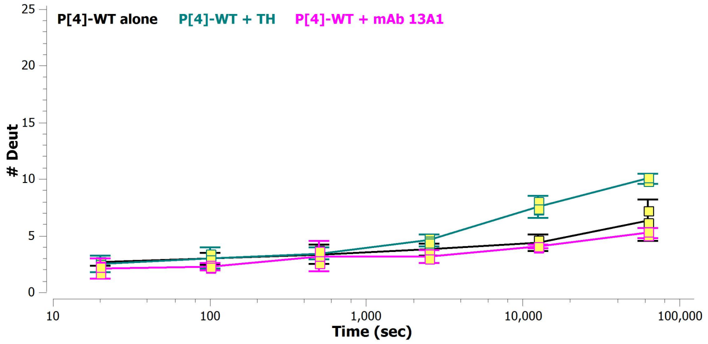

122-153: VGMLKYGGRVWTFHGETPRATTDSSNTADLNN (#51)

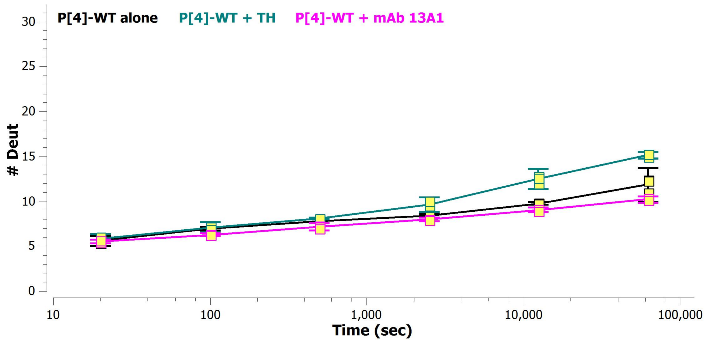


122-155: VGMLKYGGRVWTFHGETPRATTDSSNTADLNNIS (#52)

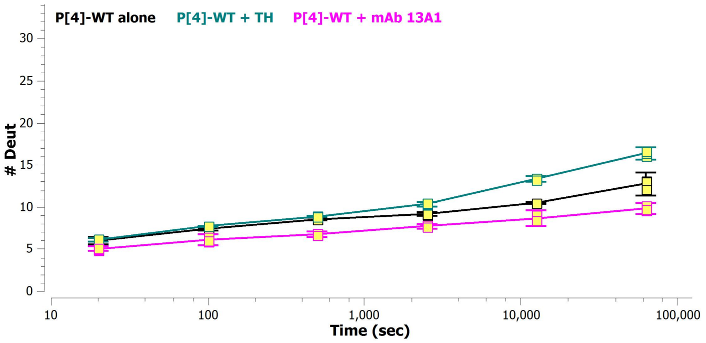


124-134: MLKYGGRVWTF (#53)

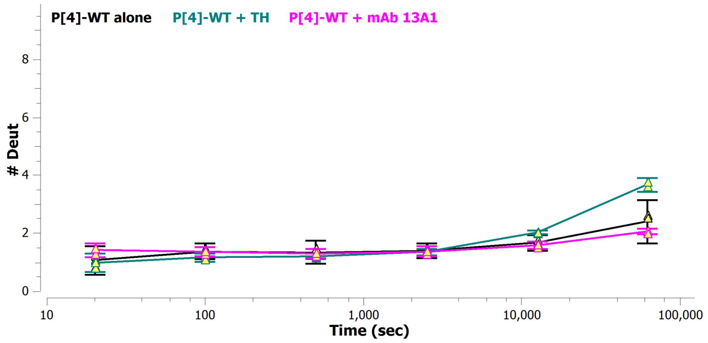


124-151: MLKYGGRVWTFHGETPRATTDSSNTADL (#54)

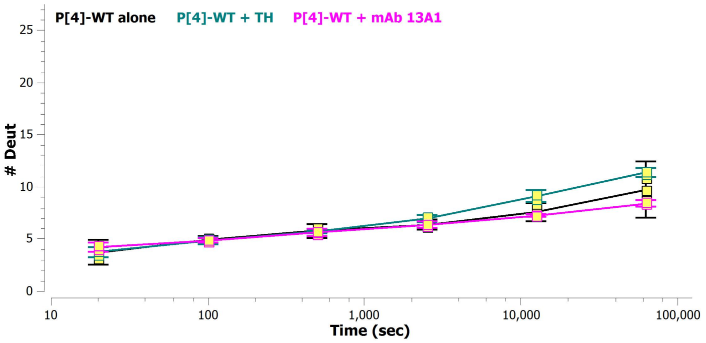


124-155: MLKYGGRVWTFHGETPRATTDSSNTADLNNIS (#55)

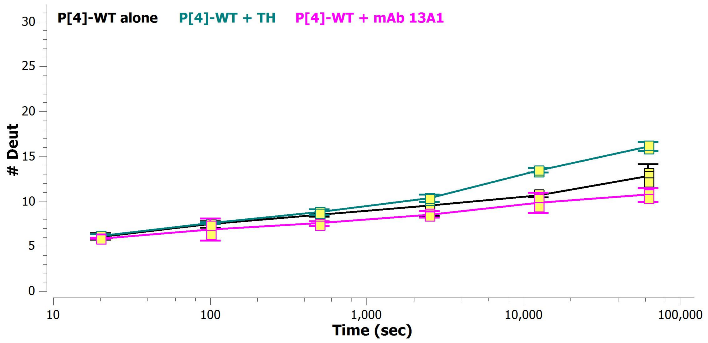


125-134: LKYGGRVWTF (#56)

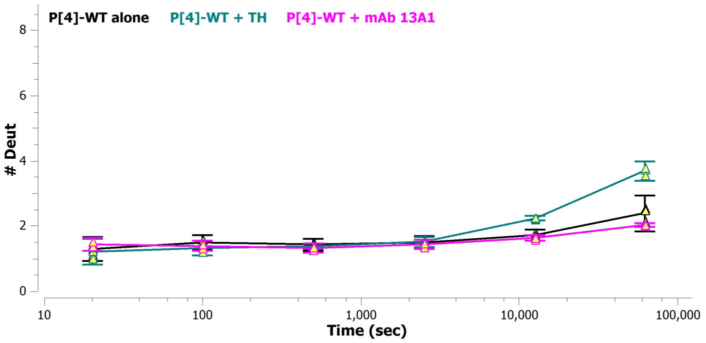


125-151: LKYGGRVWTFHGETPRATTDSSNTADL (#57)

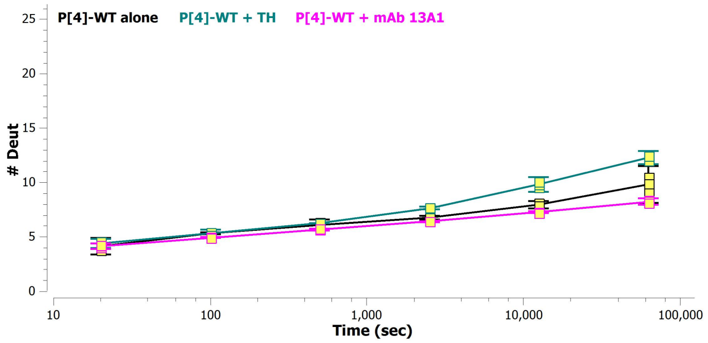


125-153: LKYGGRVWTFHGETPRATTDSSNTADLNN (#58)

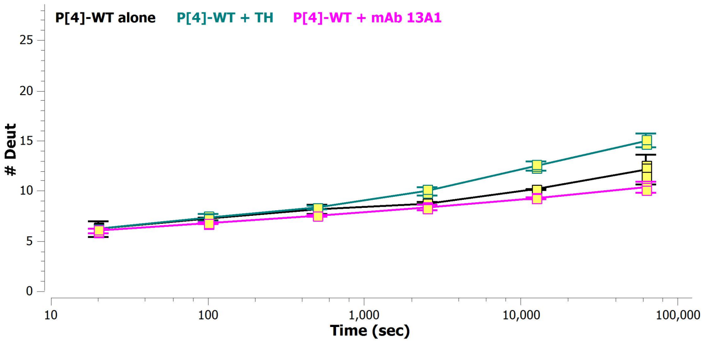


125-155: LKYGGRVWTFHGETPRATTDSSNTADLNNIS (#59)

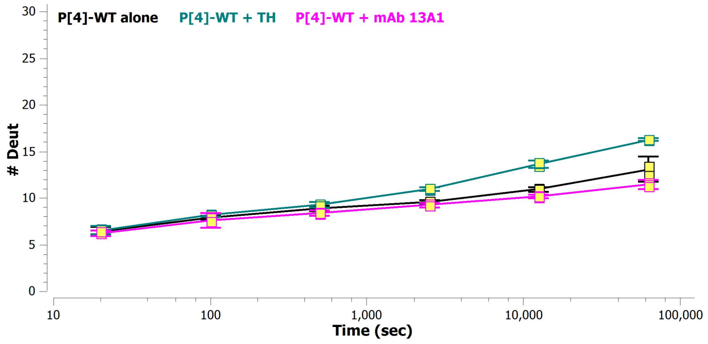


126-155: KYGGRVWTFHGETPRATTDSSNTADLNNIS (#60)

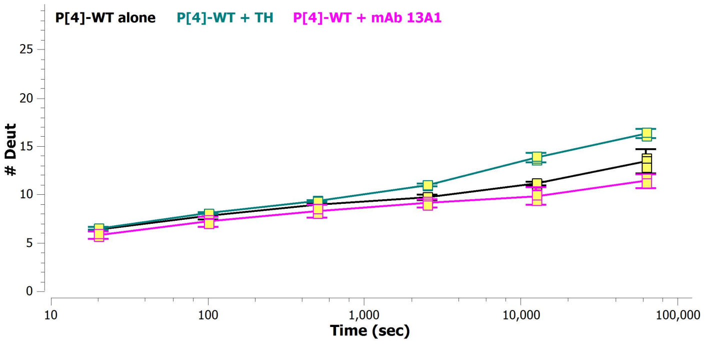

135-151: HGETPRATTDSSNTADL (#61)

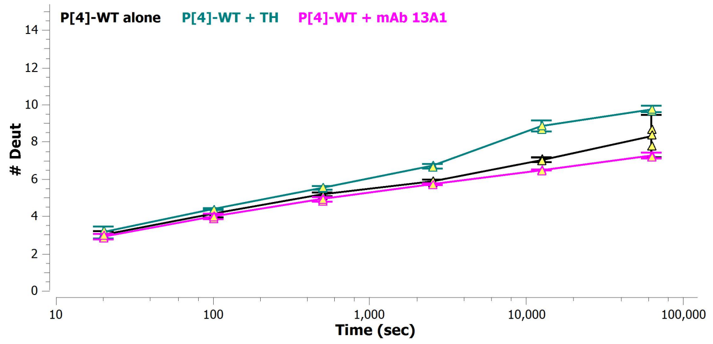


135-155: HGETPRATTDSSNTADLNNIS (#62)

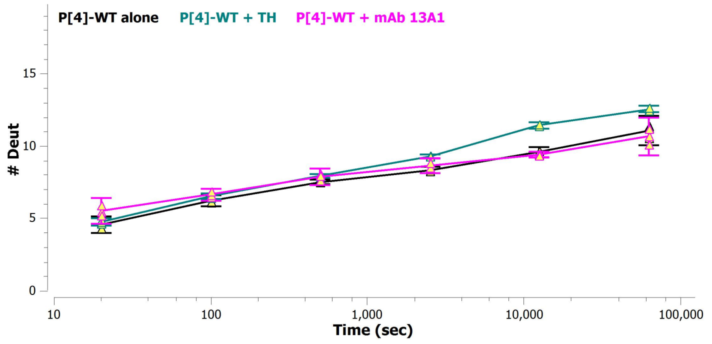


154-162: ISIIIHSEF (#63)

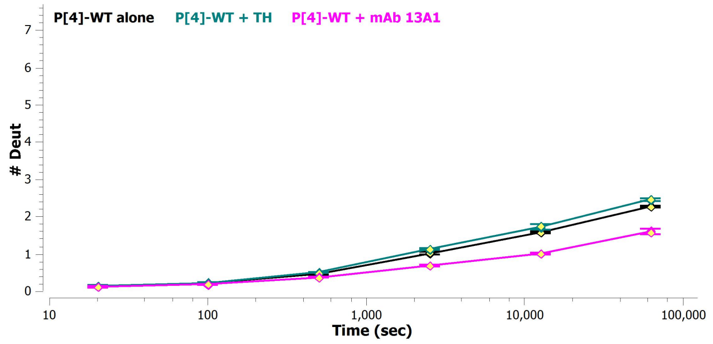


156-161: IIIHSE (#64)

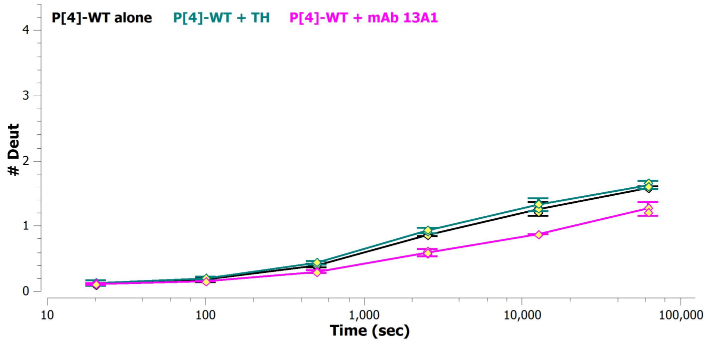


156-162: IIIHSEF (#65)

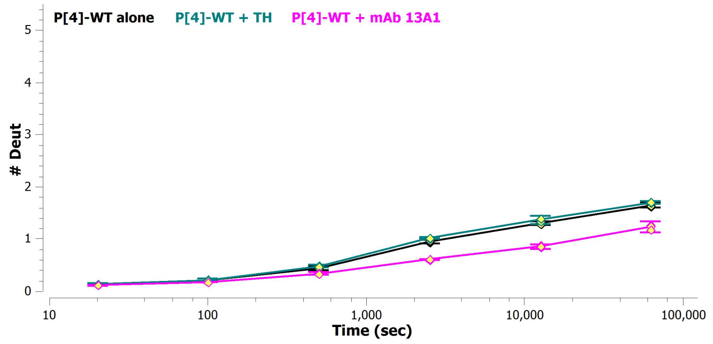


157-162: IIHSEF (#66)

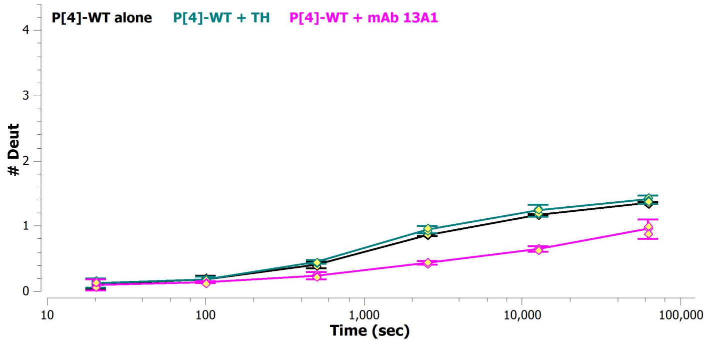


158-162: IHSEF (#67)

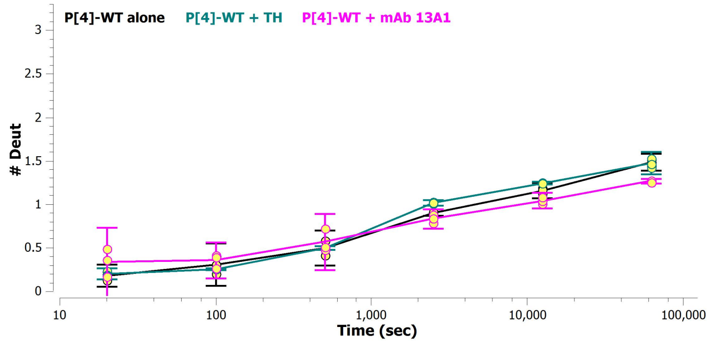


163-170: YIIPRSQE (#68)

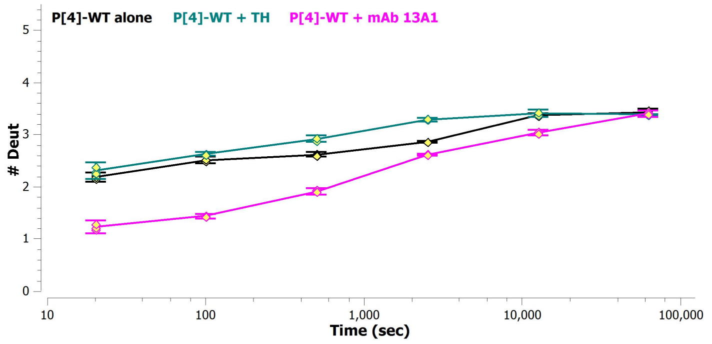


163-173: YIIPRSQESKC (#69)

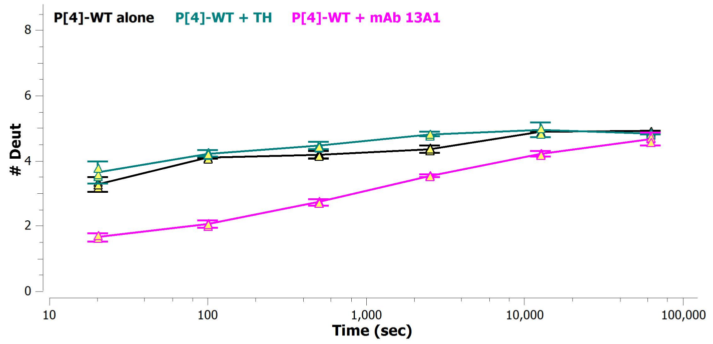


163-175: YIIPRSQESKCNE (#70)

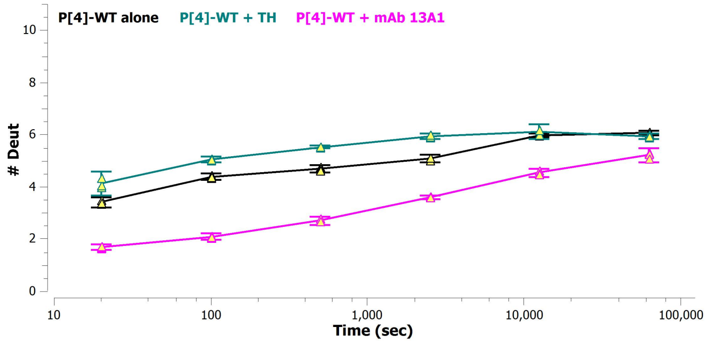

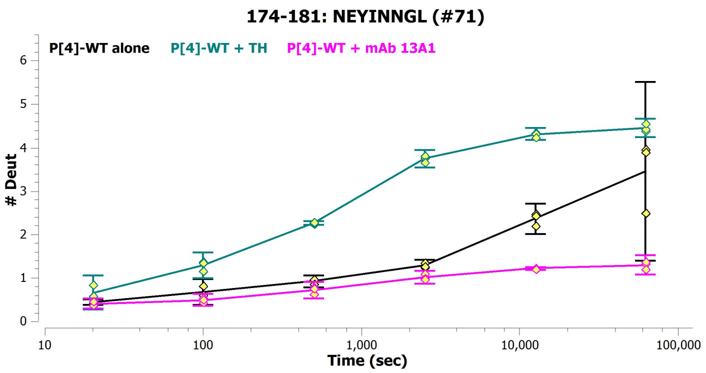
174-181: NEYINNGL (#71)
P[4]-WT alone
P[4]-WT + TH
P[4]-WT + mAb 13A1
# Deut
0
1
2
3
4
5
6
10
100
1,000
10,000
100,000
Time (sec)

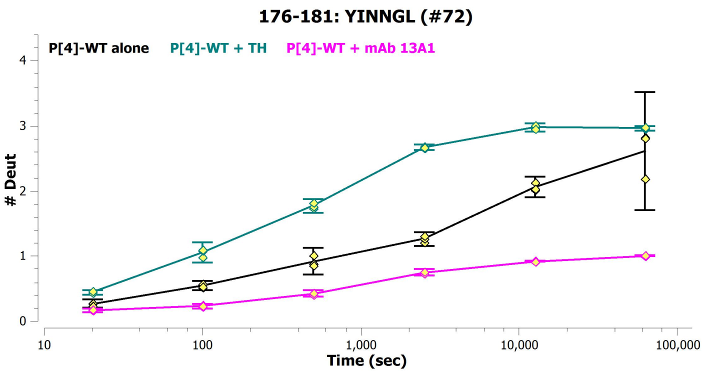
176-181: YINNGL (#72)
P[4]-WT alone
P[4]-WT + TH
P[4]-WT + mAb 13A1
# Deut
0
1
2
3
4
10
100
1,000
10,000
100,000
Time (sec)

| State | Start | End | Sequence | Search RT | Charge | Max D | 20s #D | 20s #D | 20s #D | 100s #D | 100s #D | 100s #D | 500s #D | 500s #D | 500s #D | 2500s #D | 2500s #D | 2500s #D | 12500s #D | 12500s #D | 12500s #D | 62500s #D | 62500s #D | 62500s #D |
|---|---|---|---|---|---|---|---|---|---|---|---|---|---|---|---|---|---|---|---|---|---|---|---|---|
| *Pp* P[4]-C173S alone | 1 | 6 | MQYIKA | 4.97 | 2 | 4 | 3.069 | 3.205 | 2.913 | 3.209 | 3.178 | 3.233 | 3.228 | 3.198 | 3.214 | 3.213 | 3.254 | 3.251 | 3.284 | 3.269 | 3.268 | 2.878 | 2.958 | 2.902 |
| *Pp* P[4]-C173S alone | 1 | 10 | MQYIKANSKF | 5.86 | 3 | 8 | 5.957 | 6.077 | 5.78 | 5.997 | 5.893 | 6.029 | 5.852 | 5.933 | 6.002 | 5.912 | 5.918 | 5.94 | 6.048 | 6.055 | 6.056 | 5.227 | 5.329 | 5.285 |
| *Pp* P[4]-C173S alone | 1 | 12 | MQYIKANSKFIG | 6.37 | 3 | 10 | 7.609 | 7.645 | 7.448 | 7.498 | 7.451 | 7.585 | 7.356 | 7.463 | 7.63 | 7.487 | 7.435 | 7.465 | 7.602 | 7.636 | 7.63 | 6.549 | 6.666 | 6.632 |
| *Pp* P[4]-C173S alone | 3 | 10 | YIKANSKF | 4.36 | 3 | 6 | 4.285 | 4.463 | 4.244 | 4.433 | 4.321 | 4.481 | 4.325 | 4.414 | 4.454 | 4.328 | 4.382 | 4.352 | 4.484 | 4.466 | 4.459 | 3.884 | 3.915 | 3.893 |
| *Pp* P[4]-C173S alone | 3 | 12 | YIKANSKFIG | 5.15 | 3 | 8 | 5.918 | 5.858 | 5.987 | 5.62 | 5.617 | 5.626 | 5.56 | 5.688 | 5.795 | 5.584 | 5.572 | 5.682 | 5.771 | 5.875 | 5.769 | 5.015 | 5.253 | 5.094 |
| *Pp* P[4]-C173S alone | 11 | 14 | IGIT | 3.91 | 1 | 2 | 1.687 | 1.532 | 1.446 | 2.043 | 1.663 | 1.721 | 1.747 | 1.879 | 1.756 | 1.879 | 1.891 | 1.882 | 1.734 | 1.91 | 1.89 | 1.648 | 1.689 | 1.661 |
| *Pp* P[4]-C173S alone | 11 | 15 | IGITE | 4.47 | 1 | 3 | 2.447 | 2.648 | 2.455 | 2.608 | 2.653 | 2.729 | 2.649 | 2.696 | 2.671 | 2.678 | 2.707 | 2.594 | 2.706 | 2.738 | 2.755 | 2.594 | 2.605 | 2.596 |
| *Pp* P[4]-C173S alone | 11 | 16 | IGITEL | 7.55 | 1 | 4 | 3.563 | 3.688 | 3.765 | 3.642 | 3.591 | 3.673 | 3.566 | 3.593 | 3.626 | 3.603 | 3.59 | 3.606 | 3.645 | 3.66 | 3.644 | 3.194 | 3.269 | 3.225 |
| *Pp* P[4]-C173S alone | 11 | 23 | IGITELGSGSGVL | 8.57 | 2 | 11 | 8.725 | 8.584 | 8.761 | 8.503 | 8.389 | 8.512 | 8.281 | 8.387 | 8.492 | 8.528 | 8.44 | 8.425 | 8.631 | 8.597 | 8.552 | 7.372 | 7.533 | 7.435 |
| *Pp* P[4]-C173S alone | 11 | 37 | IGITELGSGSGVLDGPYQPTTFKPPND | 8.82 | 2 | 21 | 11.722 | 11.811 | 12.413 | 12.25 | 12.235 | 12.448 | 12.732 | 12.815 | 12.934 | 14.098 | 14.051 | 13.988 | 15.491 | 15.422 | 15.449 | 14.519 | 14.734 | 14.589 |
| *Pp* P[4]-C173S alone | 11 | 39 | IGITELGSGSGVLDGPYQPTTFKPPNDYW | 10.49 | 3 | 23 | 11.048 | 11.363 | 11.693 | 11.555 | 11.623 | 11.85 | 11.759 | 12.021 | 12.219 | 12.813 | 13.124 | 13.166 | 14.182 | 14.403 | 14.523 | 13.409 | 13.829 | 13.719 |
| *Pp* P[4]-C173S alone | 13 | 16 | ITEL | 4.9 | 1 | 2 | 1.6 | 1.685 | 1.924 | 1.645 | 1.591 | 1.833 | 1.586 | 1.704 | 1.498 | 1.558 | 1.567 | 1.601 | 1.617 | 1.656 | 1.705 | 1.452 | 1.453 | 1.435 |
| *Pp* P[4]-C173S alone | 13 | 23 | ITELGSGSGVL | 7.09 | 1 | 9 | 6.219 | 6.327 | 5.629 | 6.351 | 6.266 | 6.155 | 6.277 | 6.269 | 6.108 | 6.319 | 6.329 | 6.352 | 6.471 | 6.477 | 6.488 | 5.465 | 5.467 | 5.51 |
| *Pp* P[4]-C173S alone | 13 | 37 | ITELGSGSGVLDGPYQPTTFKPPND | 8.08 | 2 | 19 | 9.57 | 9.688 | 10.103 | 10.209 | 10.161 | 10.333 | 10.778 | 10.83 | 11.015 | 12.227 | 12.15 | 12.133 | 13.59 | 13.5 | 13.541 | 12.847 | 13.009 | 12.914 |
| *Pp* P[4]-C173S alone | 15 | 37 | ELGSGSGVLDGPYQPTTFKPPND | 7.8 | 2 | 17 | 7.367 | 7.633 | 7.419 | 8.341 | 8.206 | 8.309 | 9.278 | 9.365 | 9.534 | 10.905 | 10.741 | 10.78 | 11.952 | 11.907 | 11.868 | 11.369 | 11.46 | 11.4 |
| *Pp* P[4]-C173S alone | 16 | 37 | LGSGSGVLDGPYQPTTFKPPND | 7.59 | 2 | 16 | 6.76 | 7.021 | 6.814 | 7.584 | 7.489 | 7.666 | 7.915 | 8.137 | 8.227 | 9.227 | 9.307 | 9.325 | 10.8 | 10.772 | 10.799 | 10.47 | 10.51 | 10.483 |
| *Pp* P[4]-C173S alone | 16 | 39 | LGSGSGVLDGPYQPTTFKPPNDYW | 9.79 | 2 | 18 | 6.32 | 6.708 | 6.298 | 7.169 | 7.185 | 7.337 | 7.5 | 7.723 | 7.808 | 8.652 | 8.721 | 8.75 | 9.95 | 10.077 | 10.136 | 9.745 | 9.908 | 9.866 |
| *Pp* P[4]-C173S alone | 16 | 41 | LGSGSGVLDGPYQPTTFKPPNDYWLL | 11.27 | 3 | 20 | 5.839 | 6.324 | 6.143 | 6.801 | 6.767 | 6.962 | 7.087 | 7.26 | 7.326 | 7.832 | 8.136 | 8.117 | 9.171 | 9.384 | 9.454 | 8.991 | 9.141 | 9.123 |
| *Pp* P[4]-C173S alone | 17 | 23 | GSGSGVL | 4 | 1 | 5 | 3.51 | 3.525 | 3.571 | 3.508 | 3.423 | 3.531 | 3.548 | 3.425 | 3.496 | 3.414 | 3.442 | 3.525 | 3.831 | 3.44 | 3.544 | 3.486 | 3.397 | 3.402 |
| *Pp* P[4]-C173S alone | 17 | 37 | GSGSGVLDGPYQPTTFKPPND | 7.31 | 2 | 15 | 6.72 | 6.511 | 6.527 | 7.112 | 7.095 | 7.164 | 7.554 | 7.711 | 7.807 | 8.958 | 8.963 | 8.952 | 10.454 | 10.404 | 10.388 | 10.132 | 10.213 | 10.162 |
| *Pp* P[4]-C173S alone | 17 | 39 | GSGSGVLDGPYQPTTFKPPNDYW | 9.6 | 2 | 17 | 6.043 | 6.319 | 6.487 | 6.82 | 6.89 | 6.983 | 7.252 | 7.403 | 7.527 | 8.33 | 8.62 | 8.542 | 9.758 | 9.932 | 9.885 | 9.575 | 9.734 | 9.753 |
| *Pp* P[4]-C173S alone | 24 | 37 | DGPYQPTTFKPPND | 6.11 | 2 | 8 | 2.406 | 2.563 | 2.451 | 2.725 | 2.745 | 2.789 | 3.209 | 3.263 | 3.289 | 4.358 | 4.452 | 4.438 | 5.638 | 5.717 | 5.706 | 5.941 | 5.988 | 5.957 |
| *Pp* P[4]-C173S alone | 24 | 39 | DGPYQPTTFKPPNDYW | 9.16 | 2 | 10 | 2.376 | 2.588 | 2.85 | 2.778 | 2.725 | 2.778 | 3.275 | 3.273 | 3.289 | 4.413 | 4.294 | 4.461 | 5.481 | 5.448 | 5.572 | 5.839 | 5.803 | 5.872 |
| *Pp* P[4]-C173S alone | 24 | 41 | DGPYQPTTFKPPNDYWLL | 10.95 | 2 | 12 | 2.528 | 2.547 | 2.711 | 2.568 | 2.62 | 2.635 | 3.006 | 3.026 | 2.982 | 3.876 | 4.083 | 4.079 | 4.94 | 5.071 | 5.234 | 5.283 | 5.285 | 5.241 |
| *Pp* P[4]-C173S alone | 38 | 41 | YWLL | 11.07 | 1 | 2 | 0.04 | 0.029 | 0.067 | 0.03 | 0.027 | 0.028 | 0.027 | 0.03 | 0.028 | 0.025 | 0.025 | 0.027 | 0.027 | 0.028 | 0.025 | 0.03 | 0.029 | 0.024 |
| *Pp* P[4]-C173S alone | 40 | 51 | LLISSNTNGVVY | 7.63 | 2 | 10 | 3.308 | 3.515 | 3.467 | 3.706 | 3.703 | 3.75 | 3.719 | 3.828 | 3.809 | 3.981 | 4.037 | 4.061 | 4.479 | 4.553 | 4.569 | 4.736 | 4.871 | 4.661 |
| *Pp* P[4]-C173S alone | 40 | 59 | LLISSNTNGVVYESTNNNDF | 8.16 | 2 | 18 | 3.459 | 3.796 | 3.737 | 4.038 | 4.078 | 4.119 | 4.106 | 4.183 | 4.221 | 4.472 | 4.545 | 4.558 | 5.333 | 5.412 | 5.422 | 5.725 | 5.823 | 5.845 |
| *Pp* P[4]-C173S alone | 41 | 51 | LISSNTNGVVY | 6.14 | 2 | 9 | 3.412 | 3.437 | 3.5 | 3.658 | 3.644 | 3.713 | 3.773 | 3.762 | 3.772 | 3.962 | 3.87 | 3.903 | 4.466 | 4.455 | 4.438 | 4.555 | 4.522 | 4.479 |
| *Pp* P[4]-C173S alone | 42 | 51 | ISSNTNGVVY | 5.31 | 2 | 8 | 3.294 | 3.447 | 3.179 | 3.673 | 3.68 | 3.754 | 3.72 | 3.771 | 3.8 | 3.877 | 3.918 | 3.907 | 4.271 | 4.334 | 4.335 | 4.05 | 4.039 | 4.025 |
| *Pp* P[4]-C173S alone | 42 | 59 | ISSNTNGVVYESTNNNDF | 6.79 | 2 | 16 | 3.471 | 3.778 | 3.623 | 3.979 | 4.009 | 4.058 | 4.048 | 4.158 | 4.179 | 4.45 | 4.526 | 4.527 | 5.21 | 5.263 | 5.288 | 5.307 | 5.385 | 5.381 |
| *Pp* P[4]-C173S alone | 59 | 63 | FWTAV | 8.09 | 1 | 3 | 0.095 | 0.05 | 0.097 | 0.032 | 0.023 | 0.004 | 0.028 | 0.015 | 0.022 | 0.034 | 0.021 | 0.022 | 0.04 | 0.027 | 0.015 | 0.021 | 0.032 | 0.028 |
| *Pp* P[4]-C173S alone | 59 | 64 | FWTAVI | 9.72 | 1 | 4 | 0.052 | -0.007 | 0.197 | -0.034 | -0.043 | -0.066 | -0.025 | -0.053 | -0.055 | -0.038 | -0.083 | -0.041 | -0.019 | -0.081 | -0.069 | -0.046 | -0.063 | -0.049 |
| *Pp* P[4]-C173S alone | 60 | 63 | WTAV | 5.56 | 1 | 2 | 0.038 | 0.031 | 0.067 | 0.031 | 0.03 | 0.027 | 0.025 | 0.021 | 0.023 | 0.023 | 0.026 | 0.024 | 0.027 | 0.029 | 0.03 | 0.025 | 0.023 | 0.026 |
| *Pp* P[4]-C173S alone | 60 | 64 | WTAVI | 7.8 | 1 | 3 | 0.049 | 0.042 | 0.062 | 0.036 | 0.035 | 0.032 | 0.022 | 0.025 | 0.032 | 0.029 | 0.025 | 0.033 | 0.039 | 0.029 | 0.028 | 0.023 | 0.034 | 0.029 |
| *Pp* P[4]-C173S alone | 64 | 73 | IAVEPHVSQT | 4.1 | 2 | 7 | 1.471 | 1.527 | 1.378 | 1.93 | 1.941 | 1.986 | 2.22 | 2.267 | 2.292 | 2.405 | 2.458 | 2.451 | 2.884 | 2.905 | 2.921 | 3.156 | 3.179 | 3.163 |
| *Pp* P[4]-C173S alone | 64 | 76 | IAVEPHVSQTNRQ | 3.68 | 3 | 10 | 2.716 | 2.824 | 2.667 | 3.535 | 3.493 | 3.612 | 3.948 | 4.046 | 4.131 | 4.335 | 4.372 | 4.385 | 5.29 | 5.341 | 5.361 | 5.49 | 5.436 | 5.465 |
| *Pp* P[4]-C173S alone | 64 | 79 | IAVEPHVSQTNRQYIL | 7.03 | 3 | 13 | 2.711 | 2.82 | 2.947 | 3.723 | 3.635 | 3.734 | 4.225 | 4.349 | 4.458 | 4.597 | 4.697 | 4.664 | 5.535 | 5.51 | 5.547 | 5.619 | 5.605 | 5.69 |
| *Pp* P[4]-C173S alone | 65 | 73 | AVEPHVSQT | 3.17 | 2 | 6 | 1.448 | 1.534 | 1.411 | 1.89 | 1.98 | 1.98 | 2.264 | 2.287 | 2.298 | 2.421 | 2.453 | 2.4 | 2.888 | 2.913 | 2.903 | 3.183 | 3.189 | 3.167 |
| *Pp* P[4]-C173S alone | 65 | 76 | AVEPHVSQTNRQ | 2.92 | 3 | 9 | 2.747 | 2.848 | 2.666 | 3.564 | 3.55 | 3.654 | 4.029 | 4.117 | 4.196 | 4.406 | 4.459 | 4.45 | 5.377 | 5.471 | 5.431 | 5.639 | 5.585 | 5.584 |
| *Pp* P[4]-C173S alone | 65 | 79 | AVEPHVSQTNRQYIL | 6.6 | 3 | 12 | 2.952 | 2.92 | 2.815 | 3.958 | 3.727 | 3.773 | 4.301 | 4.466 | 4.526 | 4.677 | 4.678 | 4.723 | 5.626 | 5.615 | 5.637 | 5.689 | 5.693 | 5.746 |
| *Pp* P[4]-C173S alone | 66 | 76 | VEPHVSQTNRQ | 3.68 | 2 | 8 | 2.537 | 2.567 | 2.486 | 3.267 | 3.26 | 3.412 | 3.655 | 3.679 | 3.819 | 4.102 | 4.047 | 4.094 | 4.887 | 5.002 | 5.037 | 5.055 | 4.968 | 5.145 |
| *Pp* P[4]-C173S alone | 74 | 79 | NRQYIL | 6.44 | 2 | 4 | 0.781 | 0.707 | 1.032 | 1.311 | 1.335 | 1.393 | 1.703 | 1.779 | 1.786 | 1.817 | 1.809 | 1.866 | 1.915 | 1.883 | 1.908 | 1.859 | 1.823 | 1.835 |
| *Pp* P[4]-C173S alone | 80 | 86 | FGENKQF | 5.18 | 2 | 5 | 1.265 | 1.336 | 1.329 | 1.414 | 1.393 | 1.437 | 1.375 | 1.405 | 1.44 | 1.421 | 1.424 | 1.427 | 1.595 | 1.601 | 1.602 | 1.88 | 1.875 | 1.869 |
| *Pp* P[4]-C173S alone | 80 | 97 | FGENKQFNVENNSDKWKF | 8.07 | 4 | 16 | 2.714 | 2.864 | 2.519 | 3.092 | 2.916 | 2.946 | 3.208 | 3.193 | 3.26 | 4.15 | 4.126 | 4.085 | 5.473 | 5.408 | 5.351 | 5.887 | 5.847 | 5.823 |
| *Pp* P[4]-C173S alone | 81 | 97 | GENKQFNVENNSDKWKF | 7.59 | 4 | 15 | 2.593 | 2.73 | 2.602 | 2.962 | 2.802 | 2.828 | 3.076 | 3.093 | 3.185 | 4.13 | 4.145 | 4.057 | 5.462 | 5.396 | 5.342 | 5.79 | 5.776 | 5.777 |
| *Pp* P[4]-C173S alone | 84 | 97 | KQFNVENNSDKWKF | 7.45 | 4 | 12 | 1.294 | 1.368 | 0.862 | 1.595 | 1.424 | 1.408 | 1.708 | 1.665 | 1.695 | 2.585 | 2.542 | 2.364 | 3.768 | 3.622 | 3.528 | 4.274 | 4.069 | 3.949 |
| *Pp* P[4]-C173S alone | 87 | 97 | NVENNSDKWKF | 7.21 | 3 | 9 | 0.982 | 1.057 | 0.877 | 1.177 | 1.102 | 1.121 | 1.398 | 1.43 | 1.498 | 2.293 | 2.321 | 2.306 | 3.372 | 3.386 | 3.361 | 3.403 | 3.384 | 3.41 |
| *Pp* P[4]-C173S alone | 98 | 100 | FEM | 6.21 | 1 | 1 | 0.03 | 0.023 | 0.047 | 0.027 | 0.024 | 0.023 | 0.02 | 0.02 | 0.023 | 0.024 | 0.02 | 0.02 | 0.023 | 0.026 | 0.028 | 0.028 | 0.03 | 0.027 |
| *Pp* P[4]-C173S alone | 98 | 101 | FEMF | 9.22 | 1 | 2 | 0.017 | 0.013 | 0.117 | 0.017 | 0.018 | 0.004 | 0.002 | 0.004 | 0.019 | 0.043 | -0.023 | -0.006 | -0.008 | 0.013 | 0.015 | 0.027 | 0.025 | 0.022 |
| *Pp* P[4]-C173S alone | 98 | 121 | FEMFKGSSQGDFSNRRTLTSSNRL | 7.14 | 3 | 22 | 7.53 | 7.787 | 7.315 | 8.144 | 7.955 | 7.875 | 7.753 | 8.042 | 8.142 | 8.192 | 8.246 | 8.144 | 8.674 | 8.671 | 8.722 | 8.243 | 8.234 | 8.345 |
| *Pp* P[4]-C173S alone | 98 | 123 | FEMFKGSSQGDFSNRRTLTSSNRLVG | 7.21 | 4 | 24 | 8.541 | 7.138 | 7.328 | 7.817 | 8.032 | 7.994 | 8.03 | 8.347 | 8.552 | 9.01 | 7.874 | 8.203 | 9.075 | 8.964 | 8.954 | 8.884 | 8.94 | 8.671 |
| *Pp* P[4]-C173S alone | 101 | 109 | FKGSSQGDF | 4.87 | 2 | 7 | 3.897 | 4.059 | 3.955 | 4.201 | 4.14 | 4.231 | 4.233 | 4.333 | 4.429 | 4.314 | 4.322 | 4.383 | 4.51 | 4.502 | 4.535 | 3.948 | 4.013 | 3.966 |
| *Pp* P[4]-C173S alone | 101 | 121 | FKGSSQGDFSNRRTLTSSNRL | 5.24 | 3 | 19 | 7.742 | 8.263 | 8.355 | 9.293 | 8.845 | 9.029 | 9.255 | 9.264 | 9.485 | 9.483 | 9.441 | 9.265 | 10.276 | 10.173 | 10.135 | 9.311 | 9.141 | 9.228 |
| *Pp* P[4]-C173S alone | 101 | 123 | FKGSSQGDFSNRRTLTSSNRLVG | 5.42 | 4 | 21 | 7.359 | 7.844 | 7.961 | 9.02 | 8.575 | 8.661 | 8.987 | 8.841 | 9.24 | 9.1 | 8.95 | 8.906 | 9.923 | 9.733 | 9.814 | 9.225 | 9.198 | 9.17 |
| *Pp* P[4]-C173S alone | 101 | 125 | FKGSSQGDFSNRRTLTSSNRLVGML | 7.72 | 4 | 23 | 6.966 | 7.6 | 7.436 | 8.234 | 8.302 | 8.436 | 8.407 | 8.466 | 8.909 | 8.211 | 8.644 | 8.664 | 9.206 | 9.121 | 9.131 | 8.906 | 8.819 | 9.039 |
| *Pp* P[4]-C173S alone | 102 | 121 | KGSSQGDFSNRRTLTSSNRL | 4.89 | 4 | 18 | 7.119 | 7.57 | 6.425 | 8.835 | 8.162 | 8.247 | 8.446 | 8.419 | 8.703 | 8.675 | 8.688 | 8.468 | 9.437 | 9.206 | 9.403 | 8.725 | 8.505 | 8.589 |
| *Pp* P[4]-C173S alone | 122 | 124 | VGM | 2.91 | 1 | 1 | 0.119 | 0.042 | 0.125 | -0.032 | 0.019 | -0.016 | -0.042 | 0.008 | -0.016 | 0.031 | -0.008 | -0.024 | 0.098 | 0.06 | 0.058 | 0.247 | 0.237 | 0.22 |
| *Pp* P[4]-C173S alone | 122 | 125 | VGML | 5.69 | 1 | 2 | 0.004 | 0.028 | 0.076 | 0.016 | 0.009 | 0.003 | 0.004 | 0.01 | 0.018 | 0.013 | 0.022 | 0.028 | 0.09 | 0.094 | 0.099 | 0.386 | 0.378 | 0.378 |
| *Pp* P[4]-C173S alone | 122 | 134 | VGMLKYGGRVWTF | 9.5 | 3 | 11 | 1.359 | 1.322 | 0.406 | 1.527 | 1.322 | 1.195 | 1.426 | 1.312 | 1.278 | 1.447 | 1.436 | 1.281 | 1.841 | 1.805 | 1.705 | 2.829 | 2.783 | 2.605 |
| *Pp* P[4]-C173S alone | 122 | 147 | VGMLKYGGRVWTFHGETPRATTDSSN | 7.33 | 4 | 23 | 2.35 | 2.63 | 1.79 | 3.964 | 3.302 | 3.784 | 3.858 | 3.311 | 3.576 | 4.429 | 4.361 | 4.342 | 6.695 | 6.998 | 7.004 | 7.977 | 7.909 | 8.09 |
| *Pp* P[4]-C173S alone | 122 | 153 | VGMLKYGGRVWTFHGETPRATTDSSNTADLNN | 7.45 | 4 | 29 | 6.383 | 6.701 | 5.761 | 7.231 | 7.167 | 7.164 | 8.013 | 7.872 | 8.067 | 8.737 | 8.597 | 8.417 | 10.127 | 10.151 | 10.343 | 12.526 | 11.911 | 12.109 |
| *Pp* P[4]-C173S alone | 122 | 155 | VGMLKYGGRVWTFHGETPRATTDSSNTADLNNIS | 7.75 | 4 | 31 | 6.01 | 6.617 | 6.739 | 7.728 | 7.789 | 7.879 | 8.574 | 8.732 | 8.94 | 9.178 | 9.441 | 9.404 | 10.81 | 10.987 | 11.037 | 12.772 | 13.093 | 13.073 |
| *Pp* P[4]-C173S alone | 124 | 134 | MLKYGGRVWTF | 9.17 | 3 | 9 | 1.281 | 1.296 | 0.419 | 1.535 | 1.304 | 1.177 | 1.443 | 1.271 | 1.286 | 1.439 | 1.399 | 1.276 | 1.845 | 1.801 | 1.661 | 2.597 | 2.492 | 2.386 |
| *Pp* P[4]-C173S alone | 124 | 151 | MLKYGGRVWTFHGETPRATTDSSNTADL | 7.45 | 4 | 25 | 3.952 | 4.434 | 2.734 | 5.437 | 5.095 | 4.947 | 6.111 | 5.71 | 5.815 | 6.663 | 6.526 | 6.193 | 8.523 | 7.941 | 7.696 | 10.291 | 9.536 | 9.351 |
| *Pp* P[4]-C173S alone | 124 | 155 | MLKYGGRVWTFHGETPRATTDSSNTADLNNIS | 7.41 | 4 | 29 | 6.723 | 6.709 | 6.97 | 7.551 | 7.845 | 7.834 | 8.645 | 8.701 | 9.033 | 9.612 | 9.574 | 9.471 | 11.348 | 11.372 | 11.436 | 14.192 | 13.284 | 13.229 |
| *Pp* P[4]-C173S alone | 125 | 134 | LKYGGRVWTF | 8.83 | 3 | 8 | 1.38 | 1.426 | 0.545 | 1.645 | 1.429 | 1.297 | 1.465 | 1.318 | 1.345 | 1.468 | 1.478 | 1.344 | 1.832 | 1.841 | 1.78 | 2.527 | 2.496 | 2.343 |
| *Pp* P[4]-C173S alone | 125 | 151 | LKYGGRVWTFHGETPRATTDSSNTADL | 7.14 | 4 | 24 | 4.228 | 4.674 | 3.245 | 5.618 | 5.514 | 5.385 | 6.314 | 6.144 | 6.238 | 6.893 | 6.911 | 6.658 | 8.434 | 8.26 | 8.152 | 9.949 | 9.787 | 9.698 |
| *Pp* P[4]-C173S alone | 125 | 153 | LKYGGRVWTFHGETPRATTDSSNTADLNN | 6.68 | 4 | 26 | 6.369 | 6.727 | 6.568 | 7.744 | 7.518 | 7.466 | 8.256 | 8.203 | 8.459 | 8.969 | 9.047 | 8.928 | 10.624 | 10.501 | 10.514 | 11.948 | 11.988 | 12.145 |
| *Pp* P[4]-C173S alone | 125 | 155 | LKYGGRVWTFHGETPRATTDSSNTADLNNIS | 7.12 | 4 | 28 | 6.55 | 6.943 | 6.873 | 8.278 | 8.278 | 8.437 | 8.957 | 9.121 | 9.337 | 9.774 | 9.903 | 9.857 | 11.314 | 11.448 | 11.509 | 12.843 | 13.207 | 13.162 |
| *Pp* P[4]-C173S alone | 126 | 155 | KYGGRVWTFHGETPRATTDSSNTADLNNIS | 6.74 | 4 | 27 | 6.404 | 6.946 | 6.97 | 8.151 | 8.322 | 8.372 | 9.029 | 9.176 | 9.446 | 9.929 | 10.128 | 10.011 | 11.599 | 11.723 | 11.786 | 13.138 | 13.425 | 13.451 |
| *Pp* P[4]-C173S alone | 135 | 151 | HGETPRATTDSSNTADL | 4.1 | 3 | 14 | 3.043 | 3.331 | 2.979 | 4.307 | 4.286 | 4.331 | 5.148 | 5.35 | 5.366 | 5.875 | 6.004 | 5.98 | 7.116 | 7.261 | 7.271 | 8.4 | 8.172 | 8.15 |
| *Pp* P[4]-C173S alone | 135 | 155 | HGETPRATTDSSNTADLNNIS | 4.76 | 3 | 18 | 4.681 | 5.006 | 5.335 | 6.443 | 6.499 | 6.668 | 7.445 | 7.32 | 7.762 | 8.155 | 8.504 | 8.399 | 9.523 | 9.903 | 9.761 | 10.646 | 10.877 | 10.754 |
| *Pp* P[4]-C173S alone | 154 | 162 | ISIIIHSEF | 8.38 | 2 | 7 | 0.151 | 0.154 | 0.208 | 0.261 | 0.256 | 0.252 | 0.494 | 0.522 | 0.525 | 1.044 | 1.061 | 1.071 | 1.576 | 1.62 | 1.619 | 2.248 | 2.227 | 2.277 |
| *Pp* P[4]-C173S alone | 156 | 161 | IIIHSE | 3.8 | 2 | 4 | 0.181 | 0.188 | 0.171 | 0.248 | 0.212 | 0.266 | 0.468 | 0.494 | 0.511 | 0.877 | 0.936 | 0.95 | 1.278 | 1.279 | 1.294 | 1.556 | 1.51 | 1.577 |
| *Pp* P[4]-C173S alone | 156 | 162 | IIIHSEF | 6.87 | 2 | 5 | 0.152 | 0.156 | 0.171 | 0.249 | 0.238 | 0.243 | 0.467 | 0.494 | 0.517 | 0.962 | 0.973 | 0.972 | 1.309 | 1.321 | 1.311 | 1.588 | 1.553 | 1.638 |
| *Pp* P[4]-C173S alone | 157 | 162 | IIHSEF | 5.57 | 2 | 4 | 0.1 | 0.129 | 0.235 | 0.219 | 0.212 | 0.202 | 0.414 | 0.434 | 0.454 | 0.853 | 0.869 | 0.893 | 1.144 | 1.171 | 1.167 | 1.275 | 1.23 | 1.289 |
| *Pp* P[4]-C173S alone | 162 | 175 | FYIIPRSQESKSNE | 5.95 | 3 | 11 | 4.634 | 4.798 | 5.053 | 5.235 | 5.011 | 5.199 | 5.4 | 5.566 | 5.697 | 5.732 | 5.774 | 5.828 | 5.99 | 6.049 | 6.063 | 5.383 | 5.388 | 5.429 |
| *Pp* P[4]-C173S alone | 163 | 170 | YIIPRSQE | 4.81 | 2 | 5 | 2.658 | 2.7 | 2.661 | 2.849 | 2.753 | 2.812 | 3.076 | 3.085 | 3.107 | 3.329 | 3.358 | 3.358 | 3.465 | 3.464 | 3.471 | 3.152 | 3.13 | 3.115 |
| *Pp* P[4]-C173S alone | 163 | 175 | YIIPRSQESKSNE | 4.17 | 3 | 10 | 4.842 | 5.052 | 4.858 | 5.436 | 5.312 | 5.514 | 5.721 | 5.854 | 5.968 | 6.062 | 6.086 | 6.088 | 6.389 | 6.388 | 6.401 | 5.678 | 5.627 | 5.631 |
| *Pp* P[4]-C173S alone | 163 | 181 | YIIPRSQESKSNEYINNGL | 6.78 | 3 | 16 | 5.144 | 5.632 | 5.745 | 6.327 | 6.002 | 6.155 | 7.478 | 6.953 | 7.356 | 8.11 | 8.276 | 8.257 | 9.83 | 9.902 | 9.89 | 9.68 | 9.772 | 9.795 |
| *Pp* P[4]-C173S alone | 164 | 175 | IIPRSQESKSNE | 2.74 | 3 | 9 | 6.943 | 6.989 | 7.394 | 7.011 | 6.7 | 6.978 | 6.529 | 6.754 | 6.897 | 6.679 | 6.811 | 6.845 | 6.823 | 6.974 | 6.927 | 6.243 | 5.921 | 5.888 |
| *Pp* P[4]-C173S alone | 176 | 181 | YINNGL | 5.3 | 2 | 4 | 0.342 | 0.336 | 0.433 | 0.623 | 0.655 | 0.724 | 1.051 | 1.08 | 1.124 | 1.748 | 1.799 | 1.844 | 2.704 | 2.689 | 2.63 | 2.909 | 2.877 | 2.874 |
| *Pp* P[4]-C173S with Thimerosal | 1 | 6 | MQYIKA | 4.97 | 2 | 4 | 3.312 | 3.104 | 3.283 | 3.145 | 3.277 | 3.105 | 3.219 | 3.171 | 3.211 | 3.235 | 3.242 | 3.264 | 3.316 | 2.951 | 3.147 | 2.912 | 2.967 | 2.907 |
| *Pp* P[4]-C173S with Thimerosal | 1 | 10 | MQYIKANSKF | 5.86 | 3 | 8 | 6.186 | 6.037 | 6.222 | 6.015 | 6.024 | 5.895 | 5.989 | 5.975 | 5.902 | 6.034 | 6.023 | 5.997 | 6.037 | 5.37 | 5.709 | 5.36 | 5.373 | 5.388 |
| *Pp* P[4]-C173S with Thimerosal | 1 | 12 | MQYIKANSKFIG | 6.37 | 3 | 10 | 7.714 | 7.65 | 7.858 | 7.571 | 7.581 | 7.466 | 7.535 | 7.558 | 7.437 | 7.618 | 7.621 | 7.547 | 7.663 | 6.712 | 7.254 | 6.726 | 6.785 | 6.786 |
| *Pp* P[4]-C173S with Thimerosal | 3 | 10 | YIKANSKF | 4.36 | 3 | 6 | 4.568 | 4.287 | 4.576 | 4.395 | 4.467 | 4.369 | 4.415 | 4.398 | 4.338 | 4.425 | 4.446 | 4.389 | 4.458 | 3.986 | 4.165 | 3.931 | 3.938 | 3.991 |
| *Pp* P[4]-C173S with Thimerosal | 3 | 12 | YIKANSKFIG | 5.15 | 3 | 8 | 5.822 | 5.611 | 6.088 | 5.708 | 5.759 | 5.73 | 5.798 | 5.646 | 5.45 | 5.711 | 5.637 | 5.572 | 5.609 | 4.987 | 5.293 | 4.831 | 4.929 | 5.052 |
| *Pp* P[4]-C173S with Thimerosal | 11 | 14 | IGIT | 3.91 | 1 | 2 | 1.905 | 1.87 | 1.802 | 1.545 | 1.73 | 1.572 | 1.562 | 1.768 | 1.638 | 1.945 | 1.892 | 1.894 | 1.894 | 1.791 | 1.76 | 1.65 | 1.675 | 1.664 |
| *Pp* P[4]-C173S with Thimerosal | 11 | 15 | IGITE | 4.47 | 1 | 3 | 2.74 | 2.635 | 2.753 | 2.637 | 2.71 | 2.607 | 2.686 | 2.604 | 2.689 | 2.671 | 2.723 | 2.7 | 2.774 | 2.629 | 2.518 | 2.61 | 2.61 | 2.61 |
| *Pp* P[4]-C173S with Thimerosal | 11 | 16 | IGITEL | 7.55 | 1 | 4 | 3.704 | 3.673 | 3.759 | 3.671 | 3.635 | 3.562 | 3.627 | 3.601 | 3.582 | 3.638 | 3.641 | 3.624 | 3.679 | 3.296 | 3.491 | 3.255 | 3.273 | 3.275 |
| *Pp* P[4]-C173S with Thimerosal | 11 | 23 | IGITELGSGSGVL | 8.57 | 2 | 11 | 8.672 | 8.551 | 8.801 | 8.539 | 8.469 | 8.333 | 8.511 | 8.326 | 8.418 | 8.537 | 8.559 | 8.523 | 8.595 | 7.587 | 8.181 | 7.437 | 7.643 | 7.592 |
| *Pp* P[4]-C173S with Thimerosal | 11 | 37 | IGITELGSGSGVLDGPYQPTTFKPPND | 8.82 | 2 | 21 | 11.799 | 11.694 | 12.017 | 12.4 | 12.372 | 12.157 | 12.966 | 12.921 | 12.852 | 14.186 | 14.201 | 14.153 | 15.464 | 14.259 | 15.032 | 14.692 | 14.846 | 14.854 |
| *Pp* P[4]-C173S with Thimerosal | 11 | 39 | IGITELGSGSGVLDGPYQPTTFKPPNDYW | 10.49 | 3 | 23 | 11.357 | 11.145 | 11.509 | 11.749 | 11.761 | 11.512 | 12.275 | 12.264 | 12.126 | 13.299 | 13.385 | 13.297 | 14.559 | 13.41 | 14.075 | 13.912 | 14.026 | 14.066 |
| *Pp* P[4]-C173S with Thimerosal | 13 | 16 | ITEL | 4.9 | 1 | 2 | 1.685 | 1.795 | 1.62 | 1.562 | 1.678 | 1.598 | 1.579 | 1.429 | 1.5 | 1.721 | 1.647 | 1.651 | 1.599 | 1.656 | 1.589 | 1.432 | 1.398 | 1.483 |
| *Pp* P[4]-C173S with Thimerosal | 13 | 23 | ITELGSGSGVL | 7.09 | 1 | 9 | 6.357 | 6.332 | 6.417 | 6.464 | 6.206 | 6.11 | 6.345 | 6.115 | 6.185 | 6.38 | 6.312 | 6.326 | 6.364 | 5.584 | 6.085 | 5.647 | 5.573 | 5.565 |
| *Pp* P[4]-C173S with Thimerosal | 13 | 37 | ITELGSGSGVLDGPYQPTTFKPPND | 8.08 | 2 | 19 | 9.698 | 9.615 | 9.83 | 10.246 | 10.341 | 10.129 | 10.974 | 10.869 | 10.829 | 12.21 | 12.316 | 12.225 | 13.584 | 12.536 | 13.063 | 13.052 | 13.071 | 13.1 |
| *Pp* P[4]-C173S with Thimerosal | 15 | 37 | ELGSGSGVLDGPYQPTTFKPPND | 7.8 | 2 | 17 | 7.632 | 7.46 | 7.74 | 8.218 | 8.217 | 8.166 | 9.55 | 9.436 | 9.446 | 10.93 | 10.913 | 10.838 | 11.905 | 11.001 | 11.492 | 11.419 | 11.537 | 11.504 |
| *Pp* P[4]-C173S with Thimerosal | 16 | 37 | LGSGSGVLDGPYQPTTFKPPND | 7.59 | 2 | 16 | 7.095 | 7.013 | 7.231 | 7.627 | 7.604 | 7.524 | 8.231 | 8.128 | 8.089 | 9.475 | 9.495 | 9.464 | 10.778 | 10.021 | 10.427 | 10.539 | 10.582 | 10.651 |
| *Pp* P[4]-C173S with Thimerosal | 16 | 39 | LGSGSGVLDGPYQPTTFKPPNDYW | 9.79 | 2 | 18 | 6.782 | 6.545 | 6.912 | 7.253 | 7.271 | 7.179 | 7.808 | 7.711 | 7.75 | 8.929 | 8.981 | 8.959 | 10.179 | 9.464 | 9.754 | 9.935 | 10.019 | 10.365 |
| *Pp* P[4]-C173S with Thimerosal | 16 | 41 | LGSGSGVLDGPYQPTTFKPPNDYWLL | 11.27 | 3 | 20 | 6.427 | 6.268 | 6.554 | 6.879 | 6.845 | 6.711 | 7.33 | 7.235 | 7.222 | 8.239 | 8.36 | 8.265 | 9.463 | 8.76 | 9.043 | 9.192 | 9.242 | 9.38 |
| *Pp* P[4]-C173S with Thimerosal | 17 | 23 | GSGSGVL | 4 | 1 | 5 | 3.471 | 3.203 | 3.632 | 3.499 | 3.501 | 3.488 | 3.492 | 3.479 | 3.503 | 3.564 | 3.678 | 3.603 | 3.566 | 3.224 | 3.306 | 3.162 | 3.188 | 3.197 |
| *Pp* P[4]-C173S with Thimerosal | 17 | 37 | GSGSGVLDGPYQPTTFKPPND | 7.31 | 2 | 15 | 6.483 | 6.459 | 6.624 | 7.159 | 7.144 | 7.037 | 7.76 | 7.749 | 7.714 | 9.102 | 9.102 | 9.085 | 10.383 | 9.759 | 10.022 | 10.155 | 10.27 | 10.312 |
| *Pp* P[4]-C173S with Thimerosal | 17 | 39 | GSGSGVLDGPYQPTTFKPPNDYW | 9.6 | 2 | 17 | 6.36 | 6.222 | 6.455 | 6.883 | 6.871 | 6.763 | 7.51 | 7.441 | 7.41 | 8.673 | 8.652 | 8.659 | 9.976 | 9.221 | 9.634 | 9.761 | 9.791 | 9.84 |
| *Pp* P[4]-C173S with Thimerosal | 24 | 37 | DGPYQPTTFKPPND | 6.11 | 2 | 8 | 2.596 | 2.513 | 2.612 | 2.741 | 2.746 | 2.738 | 3.293 | 3.259 | 3.253 | 4.475 | 4.498 | 4.578 | 5.703 | 5.385 | 5.452 | 5.965 | 5.991 | 6.053 |
| *Pp* P[4]-C173S with Thimerosal | 24 | 39 | DGPYQPTTFKPPNDYW | 9.16 | 2 | 10 | 2.643 | 2.628 | 2.625 | 2.743 | 2.763 | 2.723 | 3.331 | 3.164 | 3.309 | 4.487 | 4.46 | 4.44 | 5.519 | 5.323 | 5.202 | 5.885 | 5.767 | 5.937 |

| | | | | | | | | | | | | | | | | | | | | | | | | |
|---|---|---|---|---|---|---|---|---|---|---|---|---|---|---|---|---|---|---|---|---|---|---|---|---|
| *Pp* P[4]-C173S with Thimerosal | 24 | 41 | DGPYQPTTFKPPNDYWLL | 10.95 | 2 | 12 | 2.644 | 2.281 | 2.574 | 2.656 | 2.64 | 2.537 | 3.043 | 3.181 | 3.158 | 4.072 | 4.027 | 4.064 | 5.004 | 4.821 | 4.852 | 5.409 | 5.27 | 5.328 |
| *Pp* P[4]-C173S with Thimerosal | 38 | 41 | YWLL | 11.07 | 1 | 2 | 0.035 | 0.047 | 0.026 | 0.027 | 0.024 | 0.029 | 0.025 | 0.025 | 0.023 | 0.029 | 0.027 | 0.023 | 0.02 | 0.025 | 0.028 | 0.028 | 0.031 | 0.027 |
| *Pp* P[4]-C173S with Thimerosal | 40 | 51 | LLISSNTNGVVY | 7.63 | 2 | 10 | 3.571 | 3.503 | 3.658 | 3.726 | 3.793 | 3.701 | 3.884 | 3.851 | 3.833 | 4.002 | 4.066 | 4.076 | 4.532 | 4.22 | 4.442 | 4.951 | 4.909 | 4.717 |
| *Pp* P[4]-C173S with Thimerosal | 40 | 59 | LLISSNTNGVVYESTNNNDF | 8.16 | 2 | 18 | 3.869 | 3.839 | 3.953 | 4.116 | 4.113 | 4.076 | 4.218 | 4.284 | 4.213 | 4.661 | 4.633 | 4.642 | 5.435 | 5.068 | 5.209 | 5.882 | 5.901 | 5.974 |
| *Pp* P[4]-C173S with Thimerosal | 41 | 51 | LISSNTNGVVY | 6.14 | 2 | 9 | 3.482 | 3.383 | 3.55 | 3.651 | 3.709 | 3.675 | 3.749 | 3.704 | 3.712 | 3.942 | 3.919 | 3.962 | 4.448 | 4.088 | 4.248 | 4.618 | 4.549 | 4.601 |
| *Pp* P[4]-C173S with Thimerosal | 42 | 51 | ISSNTNGVVY | 5.31 | 2 | 8 | 3.503 | 3.405 | 3.59 | 3.716 | 3.749 | 3.65 | 3.804 | 3.778 | 3.753 | 3.961 | 3.956 | 3.959 | 4.29 | 3.959 | 4.155 | 4.044 | 4.068 | 4.088 |
| *Pp* P[4]-C173S with Thimerosal | 42 | 59 | ISSNTNGVVYESTNNNDF | 6.79 | 2 | 16 | 3.803 | 3.772 | 3.899 | 4.062 | 4.065 | 4.003 | 4.186 | 4.162 | 4.141 | 4.597 | 4.59 | 4.589 | 5.284 | 4.924 | 5.078 | 5.449 | 5.435 | 5.496 |
| *Pp* P[4]-C173S with Thimerosal | 59 | 63 | FWTAV | 8.09 | 1 | 3 | 0.047 | 0.029 | 0.029 | 0.038 | 0.022 | 0.025 | 0.021 | 0.022 | 0.016 | 0.022 | 0.013 | 0.032 | 0.019 | 0.013 | 0.024 | 0.017 | 0.033 | 0.024 |
| *Pp* P[4]-C173S with Thimerosal | 59 | 64 | FWTAVI | 9.72 | 1 | 4 | -0.024 | 0.039 | -0.015 | -0.045 | -0.049 | -0.059 | -0.07 | -0.059 | -0.044 | -0.06 | -0.084 | -0.067 | -0.063 | -0.05 | -0.039 | -0.047 | -0.056 | -0.074 |
| *Pp* P[4]-C173S with Thimerosal | 60 | 63 | WTAV | 5.56 | 1 | 2 | 0.027 | 0.04 | 0.033 | 0.034 | 0.023 | 0.03 | 0.029 | 0.024 | 0.022 | 0.023 | 0.025 | 0.025 | 0.029 | 0.022 | 0.021 | 0.022 | 0.022 | 0.027 |
| *Pp* P[4]-C173S with Thimerosal | 60 | 64 | WTAVI | 7.8 | 1 | 3 | 0.037 | 0.052 | 0.035 | 0.037 | 0.028 | 0.03 | 0.025 | 0.026 | 0.03 | 0.039 | 0.029 | 0.031 | 0.028 | 0.025 | 0.029 | 0.03 | 0.029 | 0.033 |
| *Pp* P[4]-C173S with Thimerosal | 64 | 73 | IAVEPHVSQT | 4.1 | 2 | 7 | 1.54 | 1.5 | 1.583 | 1.933 | 1.96 | 1.931 | 2.284 | 2.266 | 2.271 | 2.478 | 2.491 | 2.464 | 2.914 | 2.76 | 2.805 | 3.203 | 3.184 | 3.231 |
| *Pp* P[4]-C173S with Thimerosal | 64 | 76 | IAVEPHVSQTNRQ | 3.68 | 3 | 10 | 2.903 | 2.813 | 2.999 | 3.543 | 3.543 | 3.533 | 4.1 | 4.061 | 4.021 | 4.497 | 4.516 | 4.498 | 5.344 | 5.037 | 5.118 | 5.497 | 5.486 | 5.593 |
| *Pp* P[4]-C173S with Thimerosal | 64 | 79 | IAVEPHVSQTNRQYIL | 7.03 | 3 | 13 | 2.863 | 2.957 | 2.971 | 3.805 | 3.707 | 3.78 | 4.512 | 4.449 | 4.422 | 4.824 | 4.809 | 4.811 | 5.575 | 5.302 | 5.408 | 5.673 | 5.716 | 5.792 |
| *Pp* P[4]-C173S with Thimerosal | 65 | 73 | AVEPHVSQT | 3.17 | 2 | 6 | 1.539 | 1.452 | 1.616 | 1.948 | 1.962 | 1.964 | 2.292 | 2.257 | 2.299 | 2.497 | 2.48 | 2.462 | 2.953 | 2.811 | 2.831 | 3.207 | 3.207 | 3.209 |
| *Pp* P[4]-C173S with Thimerosal | 65 | 76 | AVEPHVSQTNRQ | 2.92 | 3 | 9 | 2.937 | 2.782 | 2.993 | 3.566 | 3.6 | 3.547 | 4.143 | 4.106 | 4.088 | 4.56 | 4.591 | 4.56 | 5.426 | 5.121 | 5.188 | 5.638 | 5.598 | 5.702 |
| *Pp* P[4]-C173S with Thimerosal | 65 | 79 | AVEPHVSQTNRQYIL | 6.6 | 3 | 12 | 2.963 | 3.075 | 3.07 | 3.984 | 3.765 | 3.868 | 4.559 | 4.583 | 4.515 | 4.923 | 4.875 | 4.899 | 5.683 | 5.35 | 5.488 | 5.764 | 5.754 | 5.861 |
| *Pp* P[4]-C173S with Thimerosal | 66 | 76 | VEPHVSQTNRQ | 3.68 | 2 | 8 | 2.608 | 2.513 | 2.795 | 3.365 | 3.345 | 3.272 | 3.925 | 3.839 | 3.835 | 4.206 | 4.179 | 4.114 | 4.997 | 4.795 | 4.72 | 5.115 | 5.149 | 5.289 |
| *Pp* P[4]-C173S with Thimerosal | 74 | 79 | NRQYIL | 6.44 | 2 | 4 | 0.868 | 0.785 | 0.793 | 1.296 | 1.337 | 1.351 | 1.758 | 1.789 | 1.785 | 1.882 | 1.883 | 1.85 | 1.914 | 1.848 | 1.86 | 1.798 | 1.944 | 1.878 |
| *Pp* P[4]-C173S with Thimerosal | 80 | 86 | FGENKQF | 5.18 | 2 | 5 | 1.348 | 1.298 | 1.404 | 1.403 | 1.414 | 1.378 | 1.426 | 1.398 | 1.388 | 1.471 | 1.464 | 1.455 | 1.602 | 1.475 | 1.51 | 1.886 | 1.891 | 1.917 |
| *Pp* P[4]-C173S with Thimerosal | 80 | 97 | FGENKQFNVENNSDKWKF | 8.07 | 4 | 16 | 2.959 | 2.817 | 3.006 | 2.959 | 2.958 | 2.972 | 3.296 | 3.25 | 3.238 | 4.148 | 4.218 | 4.149 | 5.34 | 5.052 | 5.193 | 5.756 | 5.785 | 5.902 |
| *Pp* P[4]-C173S with Thimerosal | 81 | 97 | GENKQFNVENNSDKWKF | 7.59 | 4 | 15 | 2.816 | 2.747 | 2.889 | 2.869 | 2.845 | 2.882 | 3.2 | 3.151 | 3.114 | 4.138 | 4.221 | 4.162 | 5.327 | 5.103 | 5.165 | 5.715 | 5.775 | 5.81 |
| *Pp* P[4]-C173S with Thimerosal | 84 | 97 | KQFNVENNSDKWKF | 7.45 | 4 | 12 | 1.554 | 1.271 | 1.574 | 1.443 | 1.423 | 1.425 | 1.705 | 1.642 | 1.656 | 2.419 | 2.594 | 2.485 | 3.421 | 3.387 | 3.31 | 3.859 | 3.894 | 3.943 |
| *Pp* P[4]-C173S with Thimerosal | 87 | 97 | NVENNSDKWKF | 7.21 | 3 | 9 | 1.054 | 1.021 | 1.067 | 1.154 | 1.102 | 1.137 | 1.498 | 1.449 | 1.426 | 2.353 | 2.369 | 2.37 | 3.351 | 3.208 | 3.221 | 3.37 | 3.412 | 3.458 |
| *Pp* P[4]-C173S with Thimerosal | 98 | 100 | FEM | 6.21 | 1 | 1 | 0.026 | 0.031 | 0.026 | 0.03 | 0.024 | 0.03 | 0.025 | 0.024 | 0.025 | 0.026 | 0.023 | 0.026 | 0.024 | 0.024 | 0.023 | 0.034 | 0.029 | 0.029 |
| *Pp* P[4]-C173S with Thimerosal | 98 | 101 | FEMF | 9.22 | 1 | 2 | -0.001 | 0.033 | 0.038 | 0.008 | 0.012 | 0.007 | 0.002 | -0.006 | 0.006 | -0.003 | 0.02 | -0.002 | 0.002 | -0.008 | -0.005 | 0.029 | 0.008 | 0.012 |
| *Pp* P[4]-C173S with Thimerosal | 98 | 121 | FEMFKGSSQGDFSNRRTLTSSNRL | 7.14 | 3 | 22 | 7.579 | 7.155 | 7.849 | 8.319 | 7.919 | 8.154 | 8.39 | 8.175 | 8.437 | 8.381 | 8.324 | 8.386 | 8.906 | 7.883 | 8.632 | 8.243 | 8.347 | 8.644 |
| *Pp* P[4]-C173S with Thimerosal | 98 | 123 | FEMFKGSSQGDFSNRRTLTSSNRLVG | 7.21 | 4 | 24 | 8.117 | 7.024 | 7.516 | 8.443 | 8.433 | 8.191 | 8.716 | 8.682 | 8.504 | 8.582 | 8.22 | 8.65 | 9.245 | 8.489 | 8.607 | 8.737 | 9.223 | 8.656 |
| *Pp* P[4]-C173S with Thimerosal | 101 | 109 | FKGSSQGDF | 4.87 | 2 | 7 | 4.072 | 3.827 | 4.215 | 4.16 | 4.192 | 4.105 | 4.378 | 4.356 | 4.27 | 4.429 | 4.467 | 4.441 | 4.456 | 4.011 | 4.23 | 3.975 | 3.99 | 4.046 |
| *Pp* P[4]-C173S with Thimerosal | 101 | 121 | FKGSSQGDFSNRRTLTSSNRL | 5.24 | 3 | 19 | 8.661 | 8.156 | 8.812 | 8.939 | 9.007 | 8.869 | 9.483 | 9.451 | 9.47 | 9.548 | 9.816 | 9.691 | 10.016 | 8.924 | 9.359 | 9.117 | 9.298 | 9.463 |
| *Pp* P[4]-C173S with Thimerosal | 101 | 123 | FKGSSQGDFSNRRTLTSSNRLVG | 5.42 | 4 | 21 | 8.326 | 7.391 | 8.54 | 8.453 | 8.691 | 8.522 | 8.977 | 9.141 | 9.139 | 9.157 | 9.569 | 9.231 | 9.592 | 8.748 | 9.273 | 9.053 | 9.384 | 9.487 |
| *Pp* P[4]-C173S with Thimerosal | 101 | 125 | FKGSSQGDFSNRRTLTSSNRLVGML | 7.72 | 4 | 23 | 7.84 | 7.863 | 7.896 | 8.477 | 8.536 | 8.533 | 8.843 | 8.996 | 8.952 | 8.907 | 8.929 | 8.859 | 9.213 | 8.568 | 8.951 | 9.264 | 9.168 | 9.257 |
| *Pp* P[4]-C173S with Thimerosal | 102 | 121 | KGSSQGDFSNRRTLTSSNRL | 4.89 | 4 | 18 | 7.918 | 7.03 | 8.071 | 8.082 | 8.214 | 8.147 | 8.613 | 8.641 | 8.614 | 8.731 | 8.861 | 8.806 | 9.025 | 8.167 | 8.408 | 8.501 | 8.696 | 8.813 |
| *Pp* P[4]-C173S with Thimerosal | 122 | 124 | VGM | 2.91 | 1 | 1 | 0.013 | 0.106 | 0.021 | 0.009 | 0.011 | -0.004 | -0.033 | -0.039 | 0.02 | 0.033 | 0.052 | 0.007 | 0.103 | 0.035 | 0.114 | 0.215 | 0.236 | 0.286 |
| *Pp* P[4]-C173S with Thimerosal | 122 | 125 | VGML | 5.69 | 1 | 2 | 0.014 | 0.023 | 0.012 | 0.017 | 0.015 | 0.01 | 0.01 | 0.008 | 0.012 | 0.027 | 0.031 | 0.024 | 0.105 | 0.102 | 0.085 | 0.37 | 0.379 | 0.401 |
| *Pp* P[4]-C173S with Thimerosal | 122 | 134 | VGMLKYGGRVWTF | 9.5 | 3 | 11 | 1.453 | 0.859 | 1.215 | 1.021 | 1.18 | 1.077 | 1.208 | 1.162 | 1.169 | 1.224 | 1.267 | 1.27 | 1.608 | 1.458 | 1.428 | 2.474 | 2.478 | 2.558 |
| *Pp* P[4]-C173S with Thimerosal | 122 | 147 | VGMLKYGGRVWTFHGETPRATTDSSN | 7.33 | 4 | 23 | 3.491 | 2.69 | 3.899 | 3.322 | 3.194 | 4.096 | 3.333 | 3.531 | 4.145 | 4.278 | 3.659 | 3.949 | 4.945 | 4.608 | 4.664 | 8.176 | 7.934 | 7.92 |
| *Pp* P[4]-C173S with Thimerosal | 122 | 153 | VGMLKYGGRVWTFHGETPRATTDSSNTADLNN | 7.45 | 4 | 29 | 6.555 | 5.862 | 6.52 | 6.953 | 7.264 | 7.237 | 8.143 | 8.191 | 7.998 | 8.631 | 8.723 | 8.715 | 10.125 | 9.571 | 10.73 | 11.893 | 12.445 | 12.245 |
| *Pp* P[4]-C173S with Thimerosal | 122 | 155 | VGMLKYGGRVWTFHGETPRATTDSSNTADLNNIS | 7.75 | 4 | 31 | 6.656 | 6.556 | 6.799 | 7.792 | 7.838 | 7.777 | 8.96 | 8.89 | 8.833 | 9.613 | 9.682 | 9.628 | 11.158 | 10.502 | 10.762 | 13.094 | 13.188 | 13.67 |
| *Pp* P[4]-C173S with Thimerosal | 124 | 134 | MLKYGGRVWTF | 9.17 | 3 | 9 | 1.506 | 0.827 | 1.274 | 1.093 | 1.166 | 1.134 | 1.202 | 1.141 | 1.168 | 1.19 | 1.314 | 1.227 | 1.541 | 1.413 | 1.424 | 2.266 | 2.281 | 2.265 |
| *Pp* P[4]-C173S with Thimerosal | 124 | 151 | MLKYGGRVWTFHGETPRATTDSSNTADL | 7.45 | 4 | 25 | 4.624 | 3.618 | 4.465 | 4.602 | 5.017 | 4.756 | 5.686 | 5.719 | 5.734 | 6.174 | 6.423 | 6.279 | 7.543 | 7.123 | 7.112 | 9.11 | 9.357 | 9.298 |
| *Pp* P[4]-C173S with Thimerosal | 124 | 155 | MLKYGGRVWTFHGETPRATTDSSNTADLNNIS | 7.41 | 4 | 29 | 6.445 | 6.709 | 6.829 | 7.983 | 7.856 | 7.981 | 9.018 | 8.775 | 8.926 | 9.656 | 9.788 | 9.728 | 11.154 | 10.611 | 10.93 | 13.056 | 13.123 | 13.252 |
| *Pp* P[4]-C173S with Thimerosal | 125 | 134 | LKYGGRVWTF | 8.83 | 3 | 8 | 1.542 | 1.16 | 1.429 | 1.234 | 1.354 | 1.316 | 1.371 | 1.327 | 1.286 | 1.35 | 1.422 | 1.427 | 1.685 | 1.574 | 1.522 | 2.323 | 2.419 | 2.406 |
| *Pp* P[4]-C173S with Thimerosal | 125 | 151 | LKYGGRVWTFHGETPRATTDSSNTADL | 7.14 | 4 | 24 | 4.86 | 4.073 | 4.819 | 5.178 | 5.389 | 5.295 | 6.213 | 6.152 | 6.226 | 6.664 | 6.94 | 6.818 | 8.046 | 7.506 | 7.593 | 9.424 | 9.756 | 9.867 |
| *Pp* P[4]-C173S with Thimerosal | 125 | 153 | LKYGGRVWTFHGETPRATTDSSNTADLNN | 6.68 | 4 | 26 | 6.765 | 6.528 | 6.905 | 7.76 | 7.694 | 7.371 | 8.503 | 8.434 | 8.401 | 9.026 | 9.166 | 9.1 | 10.388 | 9.839 | 10.095 | 11.912 | 12.197 | 12.364 |
| *Pp* P[4]-C173S with Thimerosal | 125 | 155 | LKYGGRVWTFHGETPRATTDSSNTADLNNIS | 7.12 | 4 | 28 | 6.841 | 6.812 | 7.213 | 8.383 | 8.404 | 8.369 | 9.324 | 9.34 | 9.28 | 9.999 | 10.106 | 10.005 | 11.553 | 10.875 | 11.135 | 13.226 | 13.481 | 13.52 |
| *Pp* P[4]-C173S with Thimerosal | 126 | 155 | KYGGRVWTFHGETPRATTDSSNTADLNNIS | 6.74 | 4 | 27 | 6.981 | 6.757 | 7.184 | 8.346 | 8.569 | 8.43 | 9.561 | 9.429 | 9.421 | 10.195 | 10.3 | 10.21 | 11.761 | 10.974 | 11.385 | 13.391 | 13.66 | 13.682 |
| *Pp* P[4]-C173S with Thimerosal | 135 | 151 | HGETPRATTDSSNTADL | 4.1 | 3 | 14 | 3.437 | 3.217 | 3.492 | 4.306 | 4.33 | 4.302 | 5.303 | 5.317 | 5.28 | 6.104 | 6.166 | 6.111 | 7.18 | 6.811 | 6.84 | 8.191 | 8.125 | 8.383 |
| *Pp* P[4]-C173S with Thimerosal | 135 | 155 | HGETPRATTDSSNTADLNNIS | 4.76 | 3 | 18 | 5.154 | 5.293 | 5.388 | 6.521 | 6.502 | 6.478 | 7.777 | 7.826 | 7.681 | 8.647 | 8.673 | 8.599 | 9.875 | 9.235 | 9.366 | 11.007 | 10.841 | 11.058 |
| *Pp* P[4]-C173S with Thimerosal | 154 | 162 | ISIIIHSEF | 8.38 | 2 | 7 | 0.166 | 0.191 | 0.184 | 0.262 | 0.254 | 0.265 | 0.53 | 0.517 | 0.51 | 1.117 | 1.132 | 1.126 | 1.634 | 1.622 | 1.573 | 2.287 | 2.273 | 2.338 |
| *Pp* P[4]-C173S with Thimerosal | 156 | 161 | IIIHSE | 3.8 | 2 | 4 | 0.195 | 0.196 | 0.195 | 0.279 | 0.272 | 0.285 | 0.51 | 0.468 | 0.513 | 0.985 | 0.982 | 1.03 | 1.297 | 1.256 | 1.209 | 1.576 | 1.499 | 1.596 |
| *Pp* P[4]-C173S with Thimerosal | 156 | 162 | IIIHSEF | 6.87 | 2 | 5 | 0.168 | 0.177 | 0.162 | 0.244 | 0.239 | 0.244 | 0.519 | 0.495 | 0.491 | 1.04 | 1.036 | 1.029 | 1.326 | 1.299 | 1.243 | 1.575 | 1.615 | 1.648 |
| *Pp* P[4]-C173S with Thimerosal | 157 | 162 | IIHSEF | 5.57 | 2 | 4 | 0.147 | 0.167 | 0.148 | 0.202 | 0.192 | 0.228 | 0.458 | 0.447 | 0.419 | 0.96 | 0.975 | 0.946 | 1.182 | 1.188 | 1.113 | 1.314 | 1.269 | 1.322 |
| *Pp* P[4]-C173S with Thimerosal | 162 | 175 | FYIIPRSQESKSNE | 5.95 | 3 | 11 | 4.983 | 5.011 | 5.103 | 5.236 | 5.154 | 5.216 | 5.676 | 5.617 | 5.584 | 6.002 | 5.998 | 5.982 | 6.072 | 5.546 | 5.804 | 5.465 | 5.523 | 5.578 |
| *Pp* P[4]-C173S with Thimerosal | 163 | 170 | YIIPRSQE | 4.81 | 2 | 5 | 2.775 | 2.676 | 2.791 | 2.774 | 2.824 | 2.742 | 3.097 | 3.044 | 3.056 | 3.423 | 3.421 | 3.409 | 3.455 | 3.145 | 3.302 | 3.141 | 3.161 | 3.19 |
| *Pp* P[4]-C173S with Thimerosal | 163 | 175 | YIIPRSQESKSNE | 4.17 | 3 | 10 | 5.252 | 5.067 | 5.357 | 5.437 | 5.455 | 5.4 | 5.928 | 5.857 | 5.838 | 6.292 | 6.364 | 6.266 | 6.387 | 5.778 | 6.003 | 5.68 | 5.702 | 5.801 |
| *Pp* P[4]-C173S with Thimerosal | 163 | 181 | YIIPRSQESKSNEYINNGL | 6.78 | 3 | 16 | 5.495 | 5.522 | 5.59 | 6.202 | 6.012 | 6.126 | 7.104 | 7.089 | 7.032 | 8.525 | 8.504 | 8.412 | 9.895 | 9.356 | 9.635 | 9.817 | 9.959 | 9.971 |
| *Pp* P[4]-C173S with Thimerosal | 164 | 175 | IIPRSQESKSNE | 2.74 | 3 | 9 | 7.207 | 7.357 | 7.179 | 7.207 | 6.93 | 7.038 | 7.046 | 7.021 | 6.75 | 7.054 | 7.101 | 6.889 | 6.937 | 6.325 | 6.568 | 6.072 | 6.051 | 6.32 |
| *Pp* P[4]-C173S with Thimerosal | 176 | 181 | YINNGL | 5.3 | 2 | 4 | 0.274 | 0.352 | 0.347 | 0.652 | 0.622 | 0.67 | 1.11 | 1.103 | 1.146 | 1.835 | 1.778 | 1.837 | 2.677 | 2.69 | 2.589 | 2.897 | 2.876 | 2.924 |
| *Pp* P[4]-C173S with 13A1 | 1 | 6 | MQYIKA | 4.97 | 2 | 4 | 3.288 | 3.352 | 3.25 | 3.262 | 3.331 | 3.256 | 3.197 | 3.282 | 3.215 | 3.304 | 3.259 | 3.297 | 2.998 | 3.039 | 2.968 | 3.193 | 3.037 | 2.914 |
| *Pp* P[4]-C173S with 13A1 | 1 | 10 | MQYIKANSKF | 5.86 | 3 | 8 | 5.761 | 5.897 | 5.672 | 5.81 | 5.882 | 5.809 | 5.785 | 5.74 | 5.858 | 5.902 | 5.888 | 5.888 | 5.372 | 5.451 | 5.199 | 5.863 | 5.467 | 5.209 |
| *Pp* P[4]-C173S with 13A1 | 1 | 12 | MQYIKANSKFIG | 6.37 | 3 | 10 | 7.143 | 7.412 | 7.08 | 7.322 | 7.324 | 7.297 | 7.254 | 7.198 | 7.326 | 7.43 | 7.397 | 7.391 | 6.723 | 6.866 | 6.568 | 7.406 | 6.885 | 6.597 |
| *Pp* P[4]-C173S with 13A1 | 3 | 10 | YIKANSKF | 4.36 | 3 | 6 | 4.273 | 4.368 | 4.198 | 4.25 | 4.329 | 4.31 | 4.27 | 4.277 | 4.337 | 4.376 | 4.369 | 4.373 | 3.989 | 4.093 | 3.877 | 4.3 | 4.075 | 3.93 |
| *Pp* P[4]-C173S with 13A1 | 3 | 12 | YIKANSKFIG | 5.15 | 3 | 8 | 5.556 | 6.036 | 5.219 | 5.535 | 6.091 | 5.705 | 5.401 | 5.306 | 5.528 | 5.388 | 5.755 | 5.676 | 4.996 | 5.134 | 4.704 | 5.475 | 5.178 | 5.023 |
| *Pp* P[4]-C173S with 13A1 | 11 | 14 | IGIT | 3.91 | 1 | 2 | 1.561 | 1.672 | 1.762 | 1.881 | 1.96 | 1.665 | 1.867 | 1.83 | 1.887 | 1.79 | 1.751 | 1.756 | 1.736 | 1.683 | 1.615 | 1.883 | 1.769 | 1.606 |
| *Pp* P[4]-C173S with 13A1 | 11 | 15 | IGITE | 4.47 | 1 | 3 | 2.827 | 2.684 | 2.564 | 2.67 | 2.635 | 2.636 | 2.707 | 2.693 | 2.718 | 2.733 | 2.753 | 2.69 | 2.649 | 2.697 | 2.569 | 2.741 | 2.702 | 2.57 |
| *Pp* P[4]-C173S with 13A1 | 11 | 16 | IGITEL | 7.55 | 1 | 4 | 3.493 | 3.579 | 3.49 | 3.504 | 3.571 | 3.504 | 3.478 | 3.481 | 3.515 | 3.539 | 3.515 | 3.508 | 3.234 | 3.279 | 3.169 | 3.518 | 3.314 | 3.155 |
| *Pp* P[4]-C173S with 13A1 | 11 | 23 | IGITELGSGSGVL | 8.57 | 2 | 11 | 7.494 | 7.457 | 7.287 | 7.484 | 7.523 | 7.578 | 7.572 | 7.45 | 7.583 | 7.816 | 7.804 | 7.832 | 7.569 | 7.648 | 7.343 | 7.916 | 7.917 | 7.664 |
| *Pp* P[4]-C173S with 13A1 | 11 | 37 | IGITELGSGSGVLDGPYQPTTFKPPND | 8.82 | 2 | 21 | 9.953 | 10.19 | 9.712 | 10.426 | 10.453 | 10.302 | 10.828 | 10.73 | 10.796 | 11.821 | 11.77 | 11.729 | 12.312 | 12.395 | 11.946 | 14.321 | 13.648 | 13.123 |
| *Pp* P[4]-C173S with 13A1 | 11 | 39 | IGITELGSGSGVLDGPYQPTTFKPPNDYW | 10.49 | 3 | 23 | 9.551 | 9.626 | 9.139 | 9.906 | 9.797 | 9.794 | 10.322 | 10.073 | 10.132 | 11.163 | 10.944 | 10.952 | 11.468 | 11.471 | 10.934 | 13.322 | 12.516 | 12.104 |
| *Pp* P[4]-C173S with 13A1 | 13 | 16 | ITEL | 4.9 | 1 | 2 | 1.703 | 1.588 | 1.681 | 1.645 | 1.817 | 1.672 | 1.609 | 1.6 | 1.698 | 1.638 | 1.567 | 1.727 | 1.468 | 1.52 | 1.526 | 1.651 | 1.541 | 1.378 |
| *Pp* P[4]-C173S with 13A1 | 13 | 23 | ITELGSGSGVL | 7.09 | 1 | 9 | 5.277 | 5.575 | 5.238 | 5.153 | 5.271 | 5.473 | 5.357 | 5.391 | 5.407 | 5.469 | 5.585 | 5.581 | 5.279 | 5.577 | 5.245 | 5.986 | 5.614 | 5.654 |
| *Pp* P[4]-C173S with 13A1 | 13 | 37 | ITELGSGSGVLDGPYQPTTFKPPND | 8.08 | 2 | 19 | 7.908 | 8.139 | 7.885 | 8.349 | 8.446 | 8.306 | 8.772 | 8.761 | 8.888 | 9.848 | 9.835 | 9.811 | 10.486 | 10.576 | 10.173 | 12.377 | 11.839 | 11.443 |
| *Pp* P[4]-C173S with 13A1 | 15 | 37 | ELGSGSGVLDGPYQPTTFKPPND | 7.8 | 2 | 17 | 6.233 | 6.379 | 5.991 | 6.57 | 6.701 | 6.625 | 7.131 | 7.072 | 7.216 | 8.368 | 8.379 | 8.344 | 9.204 | 9.325 | 8.996 | 10.734 | 10.341 | 10.11 |
| *Pp* P[4]-C173S with 13A1 | 16 | 37 | LGSGSGVLDGPYQPTTFKPPND | 7.59 | 2 | 16 | 5.191 | 5.429 | 5.091 | 5.643 | 5.692 | 5.554 | 6.034 | 5.971 | 6.091 | 7.043 | 7.014 | 7.018 | 7.893 | 8.004 | 7.707 | 9.509 | 9.201 | 8.987 |
| *Pp* P[4]-C173S with 13A1 | 16 | 39 | LGSGSGVLDGPYQPTTFKPPNDYW | 9.79 | 2 | 18 | 5.06 | 5.203 | 4.912 | 5.426 | 5.432 | 5.311 | 5.725 | 5.615 | 5.707 | 6.629 | 6.532 | 6.531 | 7.37 | 7.385 | 7.106 | 8.847 | 8.487 | 8.229 |
| *Pp* P[4]-C173S with 13A1 | 16 | 41 | LGSGSGVLDGPYQPTTFKPPNDYWLL | 11.27 | 3 | 20 | 4.835 | 4.885 | 4.614 | 5.119 | 5.115 | 4.984 | 5.396 | 5.233 | 5.337 | 6.168 | 6.111 | 6.068 | 6.811 | 6.8 | 6.501 | 8.128 | 7.776 | 7.606 |
| *Pp* P[4]-C173S with 13A1 | 17 | 23 | GSGSGVL | 4 | 1 | 5 | | | | | | | 2.386 | 2.329 | 2.282 | | 2.283 | | 2.42 | | 2.58 | | | |
| *Pp* P[4]-C173S with 13A1 | 17 | 37 | GSGSGVLDGPYQPTTFKPPND | 7.31 | 2 | 15 | 4.584 | 4.654 | 4.355 | 4.974 | 5.035 | 4.807 | 5.472 | 5.352 | 5.466 | 6.438 | 6.414 | 6.41 | 7.322 | 7.398 | 7.091 | 9.012 | 8.493 | 8.383 |
| *Pp* P[4]-C173S with 13A1 | 17 | 39 | GSGSGVLDGPYQPTTFKPPNDYW | 9.6 | 2 | 17 | 4.774 | 4.847 | 4.553 | 5.155 | 5.113 | 5.119 | 5.526 | 5.426 | 5.546 | 6.384 | 6.303 | 6.337 | 7.244 | 7.191 | 6.938 | 8.657 | 8.345 | 8.136 |
| *Pp* P[4]-C173S with 13A1 | 24 | 37 | DGPYQPTTFKPPND | 6.11 | 2 | 8 | 2.414 | 2.428 | 2.31 | 2.497 | 2.525 | 2.508 | 2.796 | 2.782 | 2.824 | 3.549 | 3.491 | 3.491 | 4.223 | 4.282 | 4.125 | 5.051 | 4.877 | 4.745 |
| *Pp* P[4]-C173S with 13A1 | 24 | 39 | DGPYQPTTFKPPNDYW | 9.16 | 2 | 10 | 2.425 | 2.458 | 2.383 | 2.589 | 2.529 | 2.541 | 2.773 | 2.767 | 2.832 | 3.482 | 3.472 | 3.46 | 4.123 | 4.087 | 3.991 | 4.841 | 4.693 | 4.594 |
| *Pp* P[4]-C173S with 13A1 | 24 | 41 | DGPYQPTTFKPPNDYWLL | 10.95 | 2 | 12 | 2.328 | 2.349 | 2.318 | 2.366 | 2.389 | 2.4 | 2.605 | 2.57 | 2.575 | 3.226 | 3.211 | 3.183 | 3.689 | 3.68 | 3.55 | 4.355 | 4.2 | 4.014 |
| *Pp* P[4]-C173S with 13A1 | 38 | 41 | YWLL | 11.07 | 1 | 2 | 0.023 | 0.032 | 0.034 | 0.028 | 0.025 | 0.025 | 0.019 | 0.017 | 0.017 | 0.017 | 0.018 | 0.017 | 0.015 | 0.012 | 0.016 | 0.021 | 0.017 | 0.013 |
| *Pp* P[4]-C173S with 13A1 | 40 | 51 | LLISSNTNGVVY | 7.63 | 2 | 10 | 1.882 | 1.907 | 1.846 | 2.625 | 2.639 | 2.6 | 3.455 | 3.406 | 3.435 | 3.68 | 3.668 | 3.672 | 3.72 | 3.631 | 3.517 | 3.841 | 3.684 | 3.63 |
| *Pp* P[4]-C173S with 13A1 | 40 | 59 | LLISSNTNGVVYESTNNNDF | 8.16 | 2 | 18 | 2.301 | 2.376 | 2.258 | 3.36 | 3.008 | 3.152 | 4.056 | 4.311 | 4.387 | 4.271 | 4.236 | 4.226 | 4.445 | 4.436 | 4.276 | 4.948 | 4.789 | 4.71 |
| *Pp* P[4]-C173S with 13A1 | 41 | 51 | LISSNTNGVVY | 6.14 | 2 | 9 | 2.19 | 2.297 | 2.267 | 2.997 | 2.884 | 2.925 | 3.654 | 3.825 | 3.817 | 4.018 | 4.024 | 4.032 | 3.885 | 3.835 | 3.902 | 3.976 | 3.919 | 3.829 |
| *Pp* P[4]-C173S with 13A1 | 42 | 51 | ISSNTNGVVY | 5.31 | 2 | 8 | 1.946 | 1.976 | 1.889 | 2.642 | 2.665 | 2.66 | 3.525 | 3.49 | 3.536 | 3.76 | 3.747 | 3.729 | 3.676 | 3.691 | 3.615 | 3.815 | 3.716 | 3.644 |
| *Pp* P[4]-C173S with 13A1 | 42 | 59 | ISSNTNGVVYESTNNNDF | 6.79 | 2 | 16 | 2.2 | 2.245 | 2.13 | 2.951 | 2.916 | 2.894 | 3.767 | 3.718 | 3.789 | 4.303 | 4.27 | 4.23 | 4.589 | 4.57 | 4.45 | 5.204 | 5.072 | 4.975 |
| *Pp* P[4]-C173S with 13A1 | 59 | 63 | FWTAV | 8.09 | 1 | 3 | 0.114 | 0.097 | 0.097 | 0.049 | 0.061 | 0.087 | 0.043 | 0.041 | 0.039 | 0.03 | 0.029 | 0.028 | 0.014 | 0.003 | 0.013 | -0.004 | 0.009 | 0.001 |
| *Pp* P[4]-C173S with 13A1 | 59 | 64 | FWTAVI | 9.72 | 1 | 4 | -0.016 | 0.066 | 0.071 | 0.055 | 0.012 | 0.004 | -0.006 | 0.011 | 0.009 | -0.015 | 0.025 | 0.003 | 0.023 | -0.006 | 0.007 | -0.006 | 0.018 | 0.022 |
| *Pp* P[4]-C173S with 13A1 | 60 | 63 | WTAV | 5.56 | 1 | 2 | 0.031 | 0.031 | 0.039 | 0.039 | 0.037 | 0.033 | 0.028 | 0.023 | 0.031 | 0.029 | 0.03 | 0.031 | 0.029 | 0.029 | 0.026 | 0.031 | 0.032 | 0.031 |
| *Pp* P[4]-C173S with 13A1 | 60 | 64 | WTAVI | 7.8 | 1 | 3 | 0.046 | 0.042 | 0.066 | 0.049 | 0.043 | 0.037 | 0.027 | 0.029 | 0.031 | 0.032 | 0.031 | 0.034 | 0.028 | 0.026 | 0.035 | 0.025 | 0.026 | 0.024 |
| *Pp* P[4]-C173S with 13A1 | 64 | 73 | IAVEPHVSQT | 4.1 | 2 | 7 | 1.437 | 1.47 | 1.4 | 1.844 | 1.867 | 1.855 | 2.213 | 2.174 | 2.21 | 2.391 | 2.368 | 2.362 | 2.56 | 2.583 | 2.524 | 3.204 | 3.156 | 3.083 |
| *Pp* P[4]-C173S with 13A1 | 64 | 76 | IAVEPHVSQTNRQ | 3.68 | 3 | 10 | 2.587 | 2.69 | 2.524 | 3.314 | 3.429 | 3.346 | 3.844 | 3.818 | 3.922 | 4.248 | 4.222 | 4.232 | 4.626 | 4.718 | 4.524 | 5.499 | 5.414 | 5.345 |
| *Pp* P[4]-C173S with 13A1 | 64 | 79 | IAVEPHVSQTNRQYIL | 7.03 | 3 | 13 | 2.284 | 2.461 | 2.312 | 3.332 | 3.363 | 3.222 | 3.873 | 3.798 | 3.929 | 4.267 | 4.247 | 4.246 | 4.61 | 4.665 | 4.456 | 5.32 | 5.308 | 5.251 |
| *Pp* P[4]-C173S with 13A1 | 65 | 73 | AVEPHVSQT | 3.17 | 2 | 6 | 1.502 | 1.524 | 1.504 | 1.941 | 1.958 | 1.92 | 2.306 | 2.305 | 2.315 | 2.445 | 2.466 | 2.475 | 2.668 | 2.712 | 2.64 | 3.375 | 3.271 | 3.2 |
| *Pp* P[4]-C173S with 13A1 | 65 | 76 | AVEPHVSQTNRQ | 2.92 | 3 | 9 | 2.686 | 2.762 | 2.575 | 3.398 | 3.497 | 3.427 | 3.981 | 3.949 | 4.044 | 4.392 | 4.335 | 4.353 | 4.776 | 4.847 | 4.666 | 5.685 | 5.587 | 5.494 |
| *Pp* P[4]-C173S with 13A1 | 65 | 79 | AVEPHVSQTNRQYIL | 6.6 | 3 | 12 | 2.365 | 2.594 | 2.457 | 3.401 | 3.535 | 3.444 | 3.977 | 3.907 | 4.109 | 4.504 | 4.493 | 4.485 | 4.792 | 4.92 | 4.641 | 5.487 | 5.512 | 5.501 |
| *Pp* P[4]-C173S with 13A1 | 66 | 76 | VEPHVSQTNRQ | 3.68 | 2 | 8 | 2.463 | 2.53 | 2.397 | 3.168 | 3.302 | 3.149 | 3.64 | 3.628 | 3.74 | 4.041 | 4.086 | 3.983 | 4.426 | 4.471 | 4.271 | 5.165 | 5.12 | 5.039 |
| *Pp* P[4]-C173S with 13A1 | 74 | 79 | NRQYIL | 6.44 | 2 | 4 | 0.754 | 0.976 | 0.801 | 1.617 | 1.476 | 1.389 | 1.795 | 1.739 | 1.81 | 1.889 | 1.88 | 1.858 | 1.839 | 1.848 | 1.727 | 1.846 | 1.823 | 1.933 |
| *Pp* P[4]-C173S with 13A1 | 80 | 86 | FGENKQF | 5.18 | 2 | 5 | 1.246 | 1.301 | 1.218 | 1.35 | 1.38 | 1.363 | 1.353 | 1.345 | 1.384 | 1.428 | 1.425 | 1.432 | 1.417 | 1.451 | 1.372 | 1.861 | 1.828 | 1.786 |
| *Pp* P[4]-C173S with 13A1 | 80 | 97 | FGENKQFNVENNSDKWKF | 8.07 | 4 | 16 | 2.496 | 2.687 | 2.532 | 2.706 | 2.832 | 2.759 | 2.938 | 2.926 | 3.013 | 3.944 | 4.001 | 4.011 | 4.852 | 4.943 | 4.722 | 5.639 | 5.706 | 5.619 |
| *Pp* P[4]-C173S with 13A1 | 81 | 97 | GENKQFNVENNSDKWKF | 7.59 | 4 | 15 | 2.286 | 2.502 | 2.291 | 2.53 | 2.624 | 2.518 | 2.712 | 2.716 | 2.843 | 3.833 | 3.834 | 3.871 | 4.693 | 4.834 | 4.589 | 5.371 | 5.414 | 5.405 |
| *Pp* P[4]-C173S with 13A1 | 84 | 97 | KQFNVENNSDKWKF | 7.45 | 4 | 12 | 1.062 | 1.215 | 1.206 | 1.163 | 1.3 | 1.195 | 1.381 | 1.398 | 1.499 | 2.264 | 2.357 | 2.413 | 3.141 | 3.316 | 3.201 | 3.667 | 3.894 | 3.909 |
| *Pp* P[4]-C173S with 13A1 | 87 | 97 | NVENNSDKWKF | 7.21 | 3 | 9 | 0.903 | 0.977 | 0.883 | 1.001 | 1.082 | 1.055 | 1.324 | 1.301 | 1.327 | 2.249 | 2.274 | 2.262 | 3.126 | 3.161 | 3.047 | 3.304 | 3.358 | 3.362 |
| *Pp* P[4]-C173S with 13A1 | 98 | 100 | FEM | 6.21 | 1 | 1 | 0.03 | 0.024 | 0.034 | 0.03 | 0.031 | 0.027 | 0.019 | 0.012 | 0.019 | 0.016 | 0.019 | 0.019 | 0.021 | 0.019 | 0.017 | 0.023 | 0.022 | 0.021 |

| | | | | | | | | | | | | | | | | | | | | | | | | |
|---|---|---|---|---|---|---|---|---|---|---|---|---|---|---|---|---|---|---|---|---|---|---|---|---|
| *Pp* P[4]-C173S with 13A1 | 98 | 101 | FEMF | 9.22 | 1 | 2 | -0.013 | 0.033 | 0.059 | 0.029 | 0.005 | -0.027 | -0.029 | -0.017 | -0.006 | -0.004 | -0.012 | -0.02 | -0.014 | -0.005 | -0.018 | 0.014 | 0.002 | 0 |
| *Pp* P[4]-C173S with 13A1 | 98 | 121 | FEMFKGSSQGDFSNRRTLTSSNRL | 7.14 | 3 | 22 | 5.611 | 5.496 | 5.663 | 6.372 | 6.344 | 5.885 | 6.038 | 6.265 | 6.19 | 6.994 | 7.197 | 7.22 | 6.942 | 7.087 | 6.517 | 7.279 | 7.11 | 7.138 |
| *Pp* P[4]-C173S with 13A1 | 98 | 123 | FEMFKGSSQGDFSNRRTLTSSNRLVG | 7.21 | 4 | 24 | 5.312 | 6.04 | 5.041 | 6.128 | 7.148 | 6.138 | 6.392 | 7.502 | 6.782 | 7.45 | 8.109 | 7.978 | 7.044 | 7.36 | 6.91 | 7.415 | 7.741 | 7.523 |
| *Pp* P[4]-C173S with 13A1 | 101 | 109 | FKGSSQGDF | 4.87 | 2 | 7 | 3.917 | 3.971 | 3.859 | 4.207 | 4.223 | 4.176 | 4.389 | 4.566 | 4.532 | 4.856 | 4.54 | 4.762 | 4.568 | 4.592 | 4.458 | 4.835 | 4.625 | 4.1 |
| *Pp* P[4]-C173S with 13A1 | 101 | 121 | FKGSSQGDFSNRRTLTSSNRL | 5.24 | 3 | 19 | 6.24 | 6.862 | 6.568 | 7.291 | 7.598 | 7.151 | 7.422 | 7.43 | 7.691 | 8.581 | 8.69 | 8.7 | 8.194 | 8.535 | 7.972 | 8.53 | 8.448 | 8.316 |
| *Pp* P[4]-C173S with 13A1 | 101 | 123 | FKGSSQGDFSNRRTLTSSNRLVG | 5.42 | 4 | 21 | 6.207 | 6.792 | 6.499 | 7.119 | 7.471 | 7.043 | 7.317 | 7.287 | 7.538 | 8.336 | 8.427 | 8.483 | 8.067 | 8.378 | 7.819 | 8.487 | 8.426 | 8.286 |
| *Pp* P[4]-C173S with 13A1 | 101 | 125 | FKGSSQGDFSNRRTLTSSNRLVGML | 7.72 | 4 | 23 | 6.195 | 6.752 | 6.115 | 7.028 | 7.167 | 6.672 | 7.072 | 6.852 | 7.232 | 8.213 | 8.03 | 8.109 | 7.923 | 8.023 | 7.236 | 8.38 | 8.215 | 8.236 |
| *Pp* P[4]-C173S with 13A1 | 102 | 121 | KGSSQGDFSNRRTLTSSNRL | 4.89 | 4 | 18 | 5.53 | 6.154 | 5.95 | 6.565 | 6.751 | 6.6 | 6.669 | 6.668 | 6.984 | 7.749 | 7.882 | 7.96 | 7.404 | 7.787 | 7.344 | 7.674 | 7.712 | 7.706 |
| *Pp* P[4]-C173S with 13A1 | 122 | 124 | VGM | 2.91 | 1 | 1 | 0.03 | 0.037 | 0.123 | 0.104 | 0.02 | 0.045 | 0.013 | 0.028 | 0.04 | 0.009 | 0.031 | 0.021 | 0.069 | 0.054 | 0.075 | 0.18 | 0.157 | 0.13 |
| *Pp* P[4]-C173S with 13A1 | 122 | 125 | VGML | 5.69 | 1 | 2 | 0.053 | 0.039 | 0.069 | 0.023 | 0.02 | 0.026 | 0.068 | 0.018 | 0.028 | 0.099 | 0.093 | 0.162 | 0.13 | 0.114 | 0.096 | 0.299 | 0.252 | 0.356 |
| *Pp* P[4]-C173S with 13A1 | 122 | 134 | VGMLKYGGRVWTF | 9.5 | 3 | 11 | 1.248 | 1.451 | 1.381 | 1.143 | 1.425 | 1.364 | 1.218 | 1.298 | 1.312 | 1.305 | 1.399 | 1.381 | 1.398 | 1.506 | 1.393 | 2.028 | 2.078 | 2.013 |
| *Pp* P[4]-C173S with 13A1 | 122 | 147 | VGMLKYGGRVWTFHGETPRATTDSSN | 7.33 | 4 | 23 | 3.057 | 3.308 | 2.665 | 2.899 | 3.264 | 3.376 | 2.973 | 3.589 | 3.621 | 3.934 | 4.011 | 4.069 | 3.953 | 4.291 | 3.982 | 5.664 | 5.889 | 5.725 |
| *Pp* P[4]-C173S with 13A1 | 122 | 153 | VGMLKYGGRVWTFHGETPRATTDSSNTADLNN | 7.45 | 4 | 29 | 5.734 | 5.976 | 5.753 | 6.978 | 6.978 | 6.441 | 7.571 | 7.443 | 7.535 | 8.302 | 8.265 | 8.285 | 8.81 | 8.767 | 8.296 | 10.404 | 10.252 | 9.988 |
| *Pp* P[4]-C173S with 13A1 | 122 | 155 | VGMLKYGGRVWTFHGETPRATTDSSNTADLNNIS | 7.75 | 4 | 31 | 5.802 | 5.931 | 5.792 | 7.276 | 6.841 | 6.573 | 7.228 | 6.875 | 7.182 | 8.149 | 8.11 | 8.132 | 8.508 | 8.196 | 8.028 | 10.585 | 10.277 | 9.522 |
| *Pp* P[4]-C173S with 13A1 | 124 | 134 | MLKYGGRVWTF | 9.17 | 3 | 9 | 1.28 | 1.414 | 1.38 | 1.178 | 1.471 | 1.444 | 1.287 | 1.328 | 1.412 | 1.349 | 1.471 | 1.459 | 1.487 | 1.563 | 1.456 | 2.019 | 2.071 | 1.999 |
| *Pp* P[4]-C173S with 13A1 | 124 | 151 | MLKYGGRVWTFHGETPRATTDSSNTADL | 7.45 | 4 | 25 | 3.992 | 4.301 | 3.996 | 4.764 | 5.185 | 5.203 | 5.757 | 5.836 | 5.933 | 6.35 | 6.594 | 6.606 | 6.94 | 7.61 | 6.8 | 8.531 | 8.409 | 8.438 |
| *Pp* P[4]-C173S with 13A1 | 124 | 155 | MLKYGGRVWTFHGETPRATTDSSNTADLNNIS | 7.41 | 4 | 29 | 5.878 | 6.219 | 6.102 | 7.108 | 7.28 | 7.356 | 8.111 | 7.746 | 8.122 | 8.824 | 8.69 | 8.762 | 9.033 | 9.477 | 8.925 | 11.211 | 11.204 | 10.461 |
| *Pp* P[4]-C173S with 13A1 | 125 | 134 | LKYGGRVWTF | 8.83 | 3 | 8 | 1.302 | 1.513 | 1.391 | 1.172 | 1.49 | 1.444 | 1.285 | 1.313 | 1.384 | 1.355 | 1.417 | 1.459 | 1.485 | 1.48 | 1.422 | 2.013 | 2.042 | 1.98 |
| *Pp* P[4]-C173S with 13A1 | 125 | 151 | LKYGGRVWTFHGETPRATTDSSNTADL | 7.14 | 4 | 24 | 4.124 | 4.354 | 4.115 | 4.86 | 5.313 | 5.176 | 5.823 | 5.821 | 5.908 | 6.502 | 6.636 | 6.571 | 6.849 | 7.022 | 6.656 | 8.233 | 8.238 | 8.13 |
| *Pp* P[4]-C173S with 13A1 | 125 | 153 | LKYGGRVWTFHGETPRATTDSSNTADLNN | 6.68 | 4 | 26 | 6.024 | 6.302 | 5.891 | 7.058 | 7.16 | 7.036 | 7.816 | 7.713 | 7.749 | 8.594 | 8.548 | 8.552 | 8.957 | 8.996 | 8.571 | 10.606 | 10.323 | 10.093 |
| *Pp* P[4]-C173S with 13A1 | 125 | 155 | LKYGGRVWTFHGETPRATTDSSNTADLNNIS | 7.12 | 4 | 28 | 6.465 | 6.606 | 6.374 | 8.061 | 7.908 | 7.803 | 8.815 | 8.589 | 8.662 | 9.525 | 9.492 | 9.448 | 9.805 | 9.787 | 9.353 | 11.677 | 11.352 | 11.095 |
| *Pp* P[4]-C173S with 13A1 | 126 | 155 | KYGGRVWTFHGETPRATTDSSNTADLNNIS | 6.74 | 4 | 27 | 5.856 | 5.979 | 5.669 | 7.247 | 7.463 | 7.276 | 8.555 | 8.318 | 8.425 | 9.425 | 9.279 | 9.279 | 9.673 | 9.373 | 9.278 | 11.68 | 11.36 | 10.819 |
| *Pp* P[4]-C173S with 13A1 | 135 | 151 | HGETPRATTDSSNTADL | 4.1 | 3 | 14 | 2.942 | 3.041 | 2.881 | 3.994 | 4.165 | 4.062 | 5.02 | 4.984 | 5.064 | 5.786 | 5.771 | 5.748 | 6.15 | 6.222 | 6.043 | 7.262 | 7.168 | 7.091 |
| *Pp* P[4]-C173S with 13A1 | 135 | 155 | HGETPRATTDSSNTADLNNIS | 4.76 | 3 | 18 | 5.012 | 5.256 | 5.028 | 6.568 | 6.431 | 6.309 | 7.576 | 7.528 | 7.541 | 8.395 | 8.247 | 8.428 | 8.512 | 8.617 | 8.384 | 9.825 | 9.593 | 9.367 |
| *Pp* P[4]-C173S with 13A1 | 154 | 162 | ISIIIHSEF | 8.38 | 2 | 7 | 0.138 | 0.151 | 0.16 | 0.22 | 0.223 | 0.215 | 0.414 | 0.393 | 0.411 | 0.734 | 0.736 | 0.738 | 1.047 | 1.046 | 1.016 | 1.689 | 1.712 | 1.73 |
| *Pp* P[4]-C173S with 13A1 | 156 | 161 | IIIHSE | 3.8 | 2 | 4 | 0.153 | 0.169 | 0.152 | 0.206 | 0.199 | 0.212 | 0.335 | 0.312 | 0.348 | 0.637 | 0.621 | 0.62 | 0.879 | 0.895 | 0.834 | 1.255 | 1.343 | 1.365 |
| *Pp* P[4]-C173S with 13A1 | 156 | 162 | IIIHSEF | 6.87 | 2 | 5 | 0.135 | 0.152 | 0.137 | 0.195 | 0.203 | 0.191 | 0.345 | 0.338 | 0.356 | 0.631 | 0.636 | 0.636 | 0.847 | 0.854 | 0.802 | 1.236 | 1.266 | 1.291 |
| *Pp* P[4]-C173S with 13A1 | 157 | 162 | IIHSEF | 5.57 | 2 | 4 | 0.11 | 0.117 | 0.102 | 0.148 | 0.148 | 0.14 | 0.226 | 0.227 | 0.254 | 0.464 | 0.441 | 0.475 | 0.632 | 0.657 | 0.601 | 0.944 | 0.984 | 1.018 |
| *Pp* P[4]-C173S with 13A1 | 162 | 175 | FYIIPRSQESKSNE | 5.95 | 3 | 11 | 2.557 | 2.738 | 2.571 | 3.292 | 3.358 | 3.21 | 3.604 | 3.57 | 3.696 | 4.51 | 4.499 | 4.495 | 4.942 | 5.061 | 4.79 | 5.413 | 5.382 | 5.317 |
| *Pp* P[4]-C173S with 13A1 | 163 | 170 | YIIPRSQE | 4.81 | 2 | 5 | 2.178 | 2.254 | 2.113 | 2.532 | 2.603 | 2.518 | 2.596 | 2.603 | 2.599 | 2.906 | 2.898 | 2.918 | 3.087 | 3.156 | 3.039 | 3.318 | 3.205 | 3.153 |
| *Pp* P[4]-C173S with 13A1 | 163 | 175 | YIIPRSQESKSNE | 4.17 | 3 | 10 | 2.88 | 3.039 | 2.845 | 3.59 | 3.701 | 3.581 | 4.058 | 4.02 | 4.165 | 5.021 | 4.994 | 5.004 | 5.446 | 5.556 | 5.291 | 5.917 | 5.867 | 5.792 |
| *Pp* P[4]-C173S with 13A1 | 163 | 181 | YIIPRSQESKSNEYINNGL | 6.78 | 3 | 16 | 2.91 | 3.369 | 2.982 | 3.896 | 4.051 | 3.896 | 4.495 | 4.123 | 4.769 | 6.211 | 6.462 | 6.203 | 6.554 | 7.195 | 6.487 | 7.604 | 7.44 | 8.029 |
| *Pp* P[4]-C173S with 13A1 | 164 | 175 | IIPRSQESKSNE | 2.74 | 2 | 9 | 6.95 | 7.555 | 6.651 | 7.64 | 7.462 | 7.43 | 7.404 | 7.329 | 7.546 | 7.762 | 7.774 | 7.653 | 7.533 | 7.522 | 7.5 | 5.597 | 5.703 | 5.455 |
| *Pp* P[4]-C173S with 13A1 | 176 | 181 | YINNGL | 5.3 | 2 | 4 | 0.124 | 0.22 | 0.172 | 0.259 | 0.261 | 0.194 | 0.387 | 0.388 | 0.363 | 0.762 | 0.744 | 0.733 | 0.931 | 0.971 | 0.961 | 1.239 | 1.269 | 1.281 |

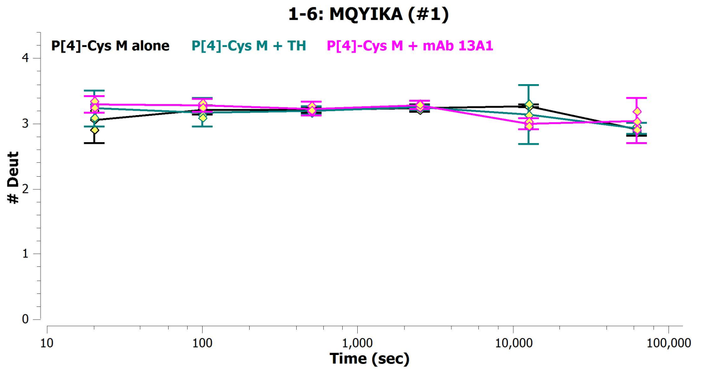
1-6: MQYIKA (#1)
P[4]-Cys M alone
P[4]-Cys M + TH
P[4]-Cys M + mAb 13A1
# Deut
0
1
2
3
4
10
100
1,000
10,000
100,000
Time (sec)

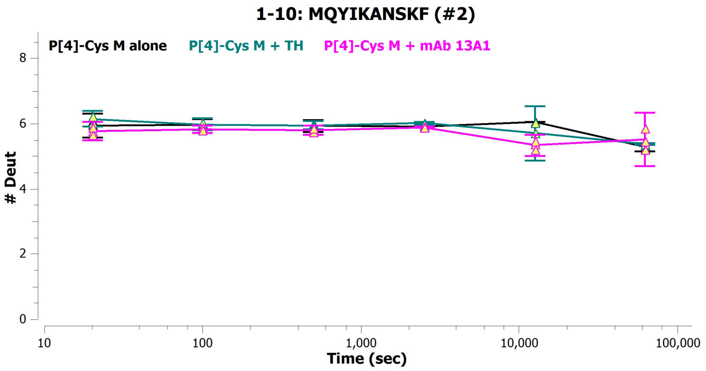
1-10: MQYIKANSKF (#2)
P[4]-Cys M alone
P[4]-Cys M + TH
P[4]-Cys M + mAb 13A1
# Deut
0
2
4
6
8
10
100
1,000
10,000
100,000
Time (sec)

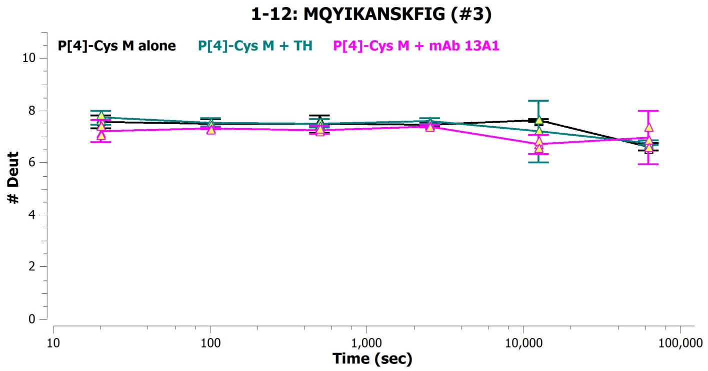
1-12: MQYIKANSKFIG (#3)
P[4]-Cys M alone
P[4]-Cys M + TH
P[4]-Cys M + mAb 13A1
# Deut
0
2
4
6
8
10
10
100
1,000
10,000
100,000
Time (sec)

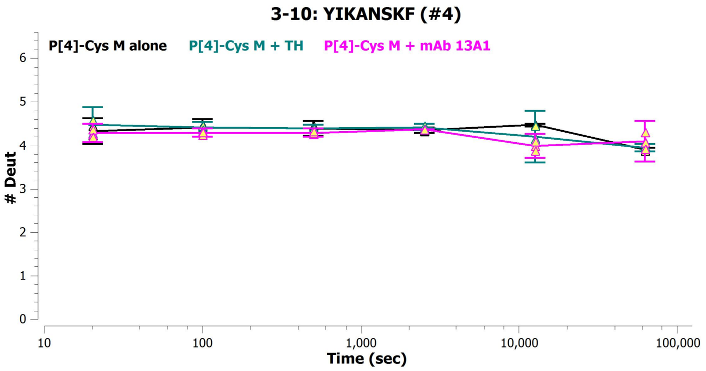
3-10: YIKANSKF (#4)
P[4]-Cys M alone
P[4]-Cys M + TH
P[4]-Cys M + mAb 13A1
# Deut
0
1
2
3
4
5
6
10
100
1,000
10,000
100,000
Time (sec)

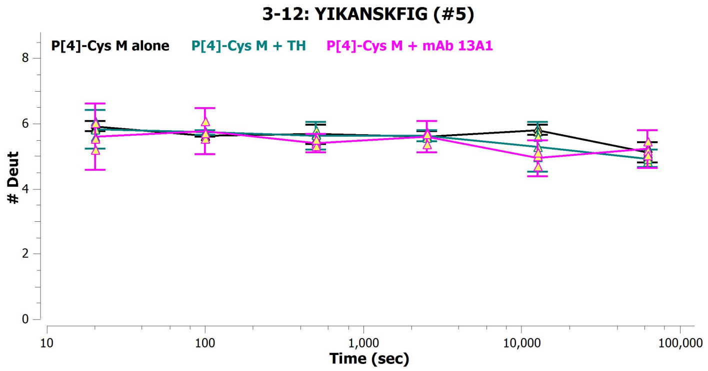
3-12: YIKANSKFIG (#5)
P[4]-Cys M alone
P[4]-Cys M + TH
P[4]-Cys M + mAb 13A1
# Deut
0
2
4
6
8
10
100
1,000
10,000
100,000
Time (sec)

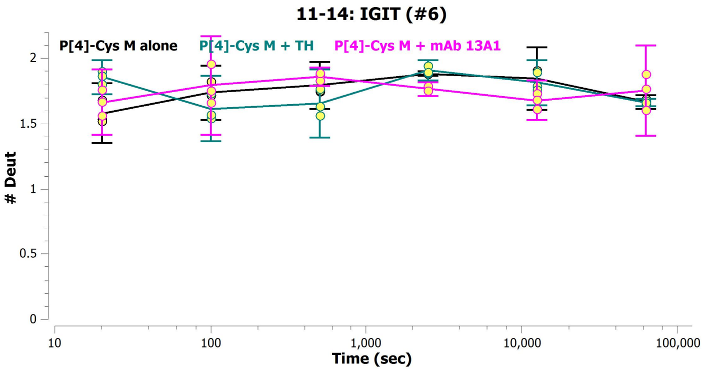
11-14: IGIT (#6)
P[4]-Cys M alone
P[4]-Cys M + TH
P[4]-Cys M + mAb 13A1
# Deut
0
0.5
1
1.5
2
10
100
1,000
10,000
100,000
Time (sec)

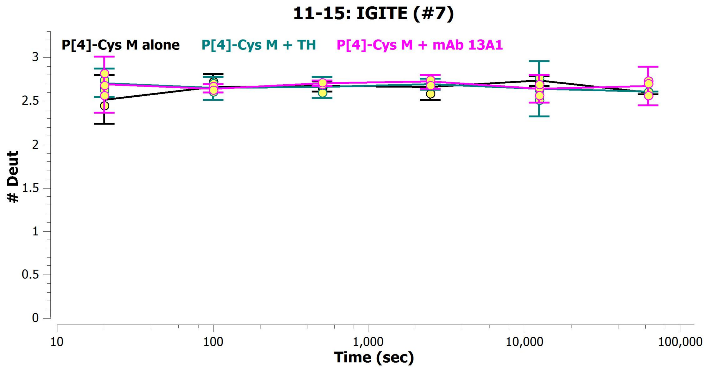
11-15: IGITE (#7)
P[4]-Cys M alone
P[4]-Cys M + TH
P[4]-Cys M + mAb 13A1
# Deut
0
0.5
1
1.5
2
2.5
3
10
100
1,000
10,000
100,000
Time (sec)

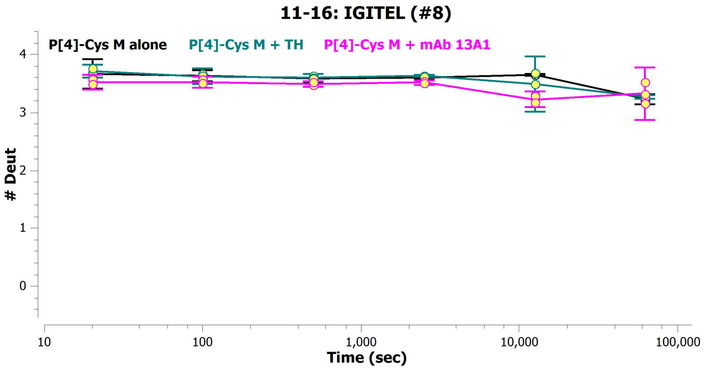
11-16: IGITEL (#8)
P[4]-Cys M alone
P[4]-Cys M + TH
P[4]-Cys M + mAb 13A1
# Deut
0
1
2
3
4
10
100
1,000
10,000
100,000
Time (sec)

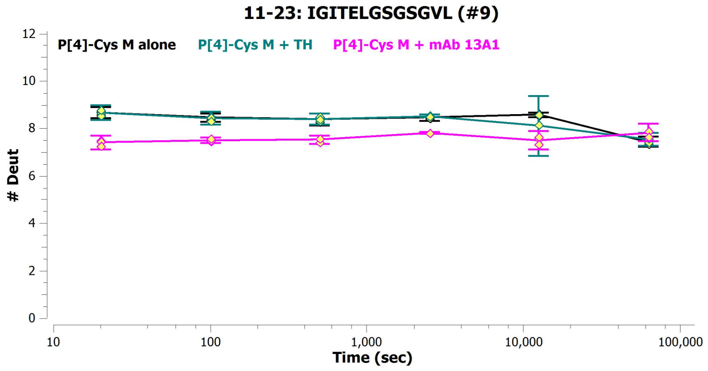
11-23: IGITELGSGSGVL (#9)
P[4]-Cys M alone
P[4]-Cys M + TH
P[4]-Cys M + mAb 13A1
# Deut
0
2
4
6
8
10
12
10
100
1,000
10,000
100,000
Time (sec)

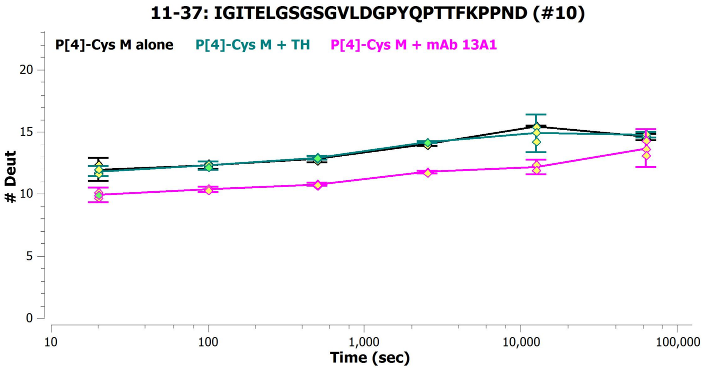
11-37: IGITELGSGSGVLDGPYQPTTFKPPND (#10)
P[4]-Cys M alone
P[4]-Cys M + TH
P[4]-Cys M + mAb 13A1
# Deut
0
5
10
15
20
10
100
1,000
10,000
100,000
Time (sec)

**11-39: IGITELGSGSGVLDGPYQPTTFKPPNDYW (#11)**

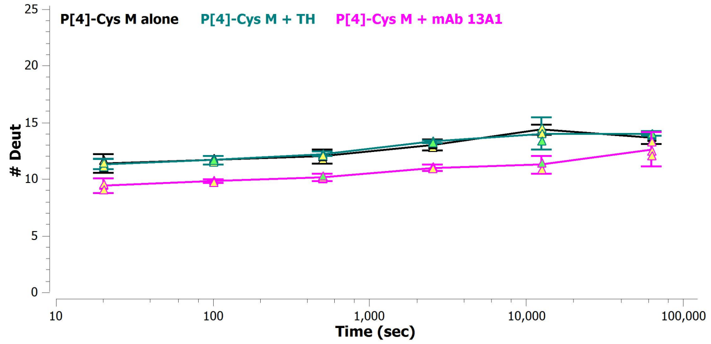


**13-16: ITEL (#12)**

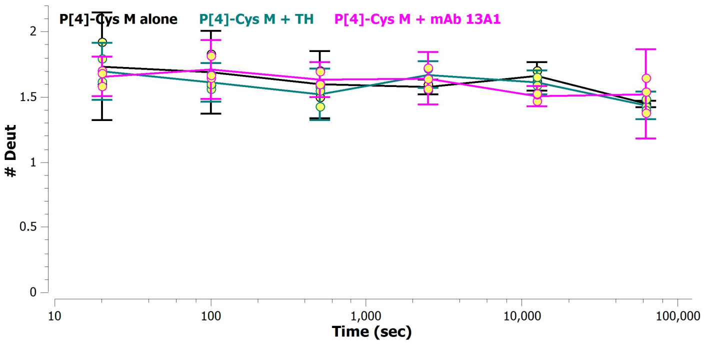


**13-23: ITELGSGSGVL (#13)**

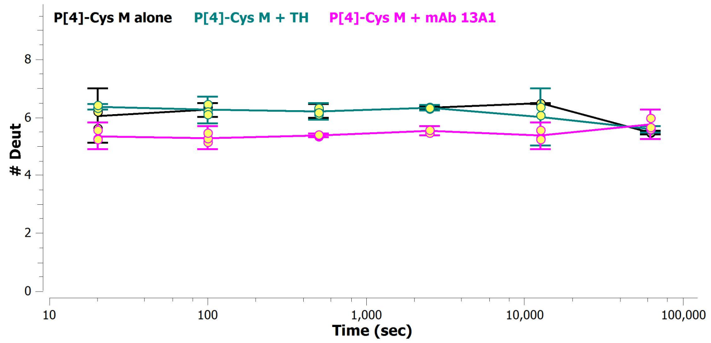


**13-37: ITELGSGSGVLDGPYQPTTFKPPND (#14)**

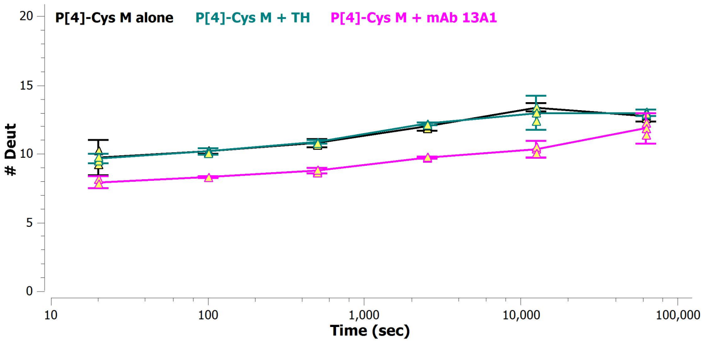


**15-37: ELGSGSGVLDGPYQPTTFKPPND (#15)**

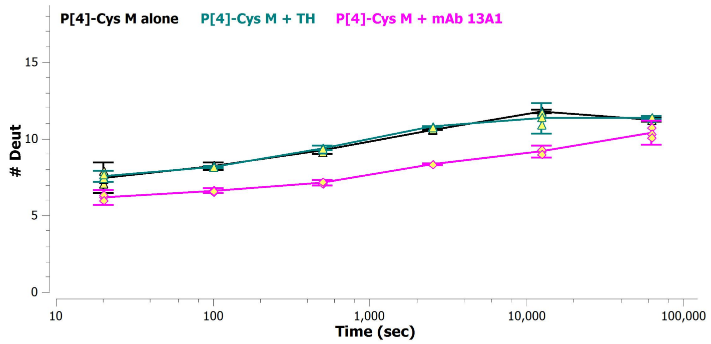


**16-37: LGSGSGVLDGPYQPTTFKPPND (#16)**

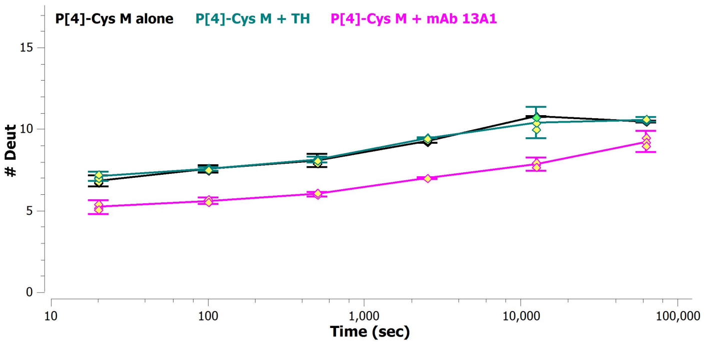


**16-39: LGSGSGVLDGPYQPTTFKPPNDYW (#17)**

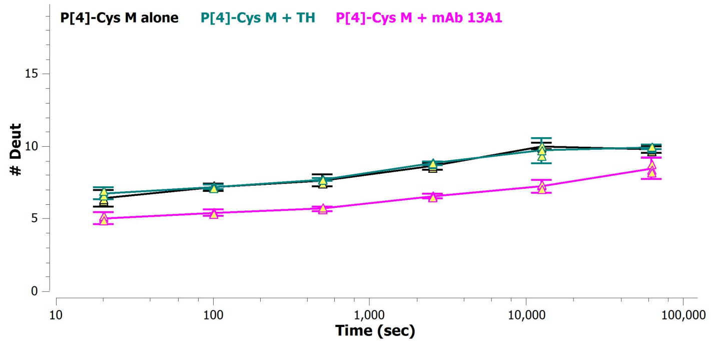


**16-41: LGSGSGVLDGPYQPTTFKPPNDYWLL (#18)**

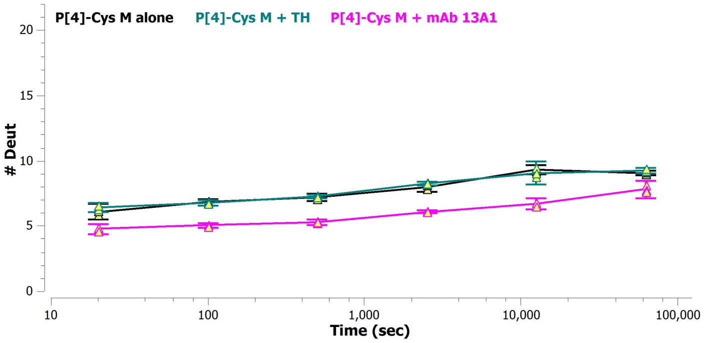


**17-23: GSGSGVL (#19)**

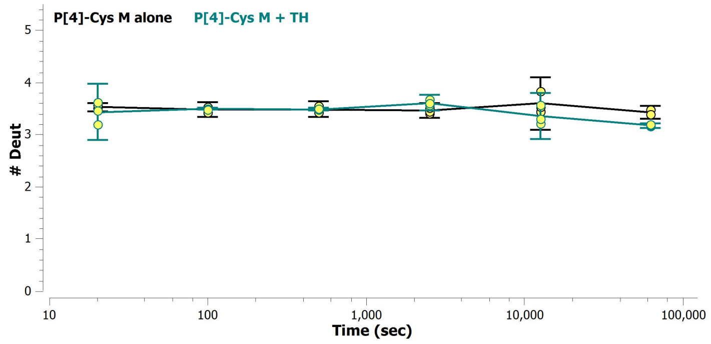


**17-37: GSGSGVLDGPYQPTTFKPPND (#20)**

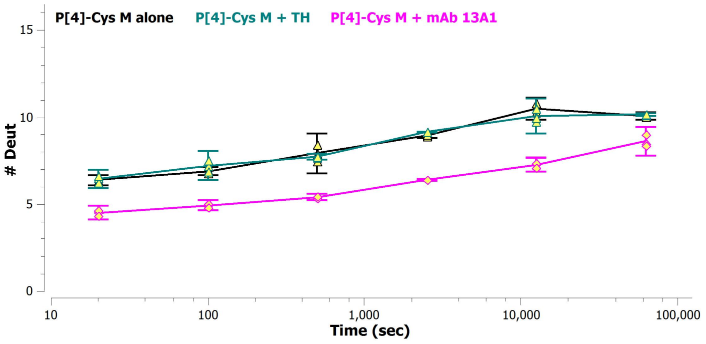

### 17-39: GSGSGVLDGPYQPTTFKPPNDYW (#21)

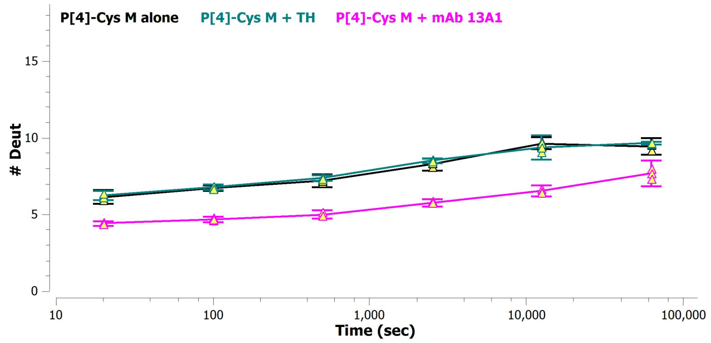


### 24-37: DGPYQPTTFKPPND (#22)

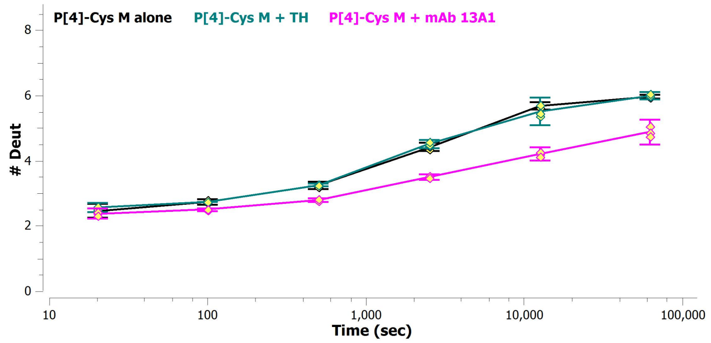


### 24-39: DGPYQPTTFKPPNDYW (#23)

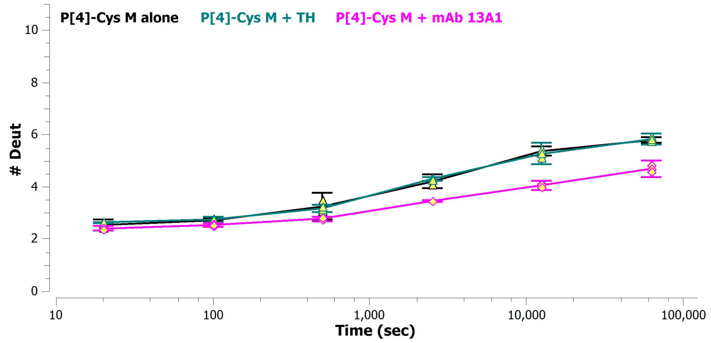


### 24-41: DGPYQPTTFKPPNDYWLL (#24)

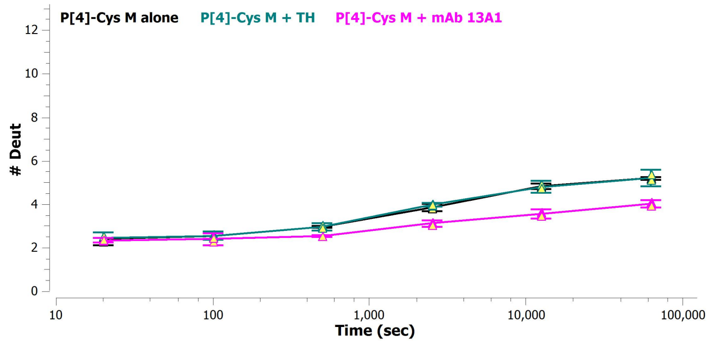


### 38-41: YWLL (#25)

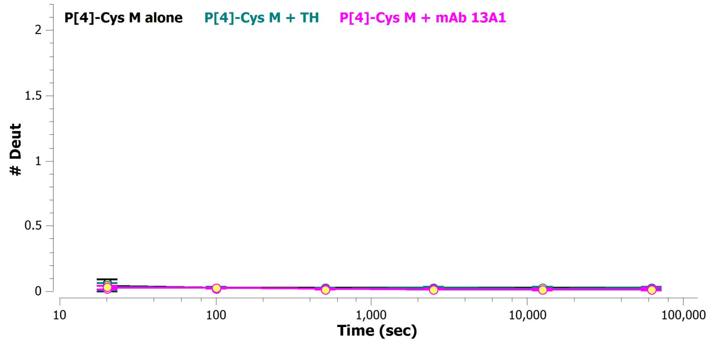


### 40-51: LLISSNTNGVVY (#26)

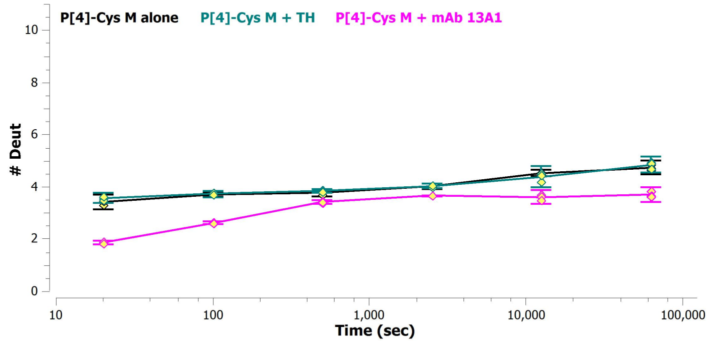


### 40-59: LLISSNTNGVVYESTNNNDF (#27)

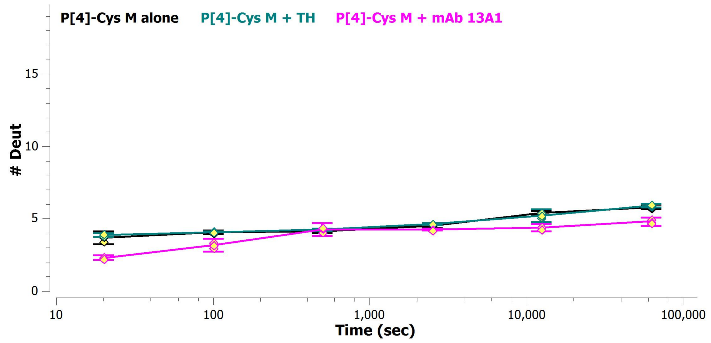


### 41-51: LISSNTNGVVY (#28)

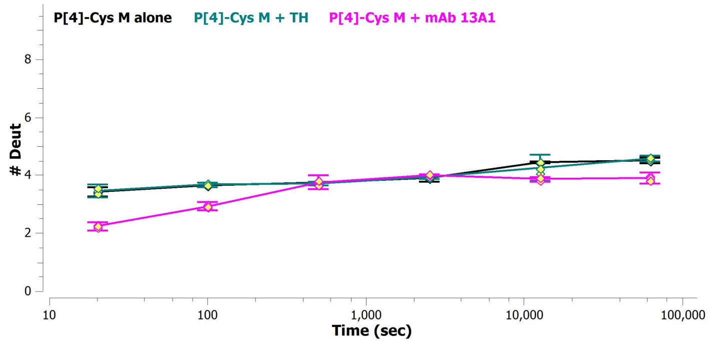


### 42-51: ISSNTNGVVY (#29)

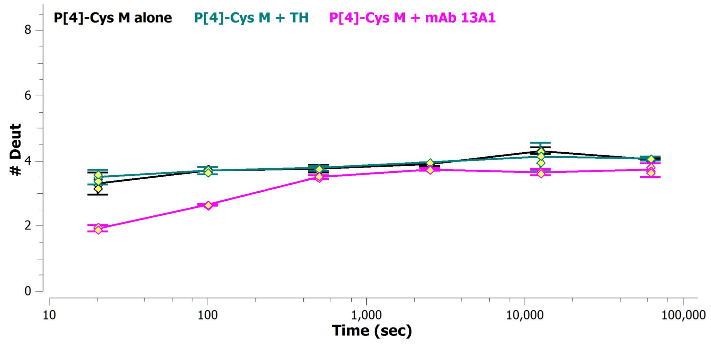


### 42-59: ISSNTNGVVYESTNNNDF (#30)

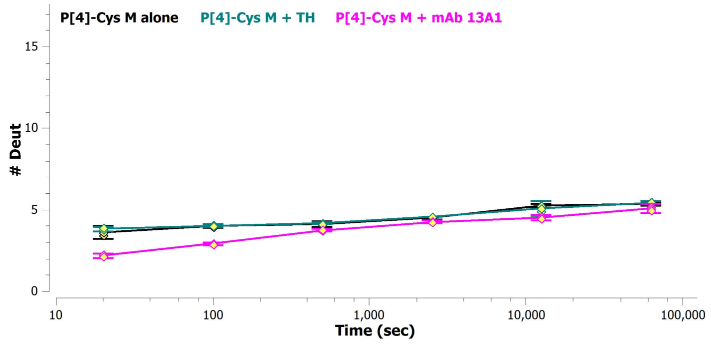

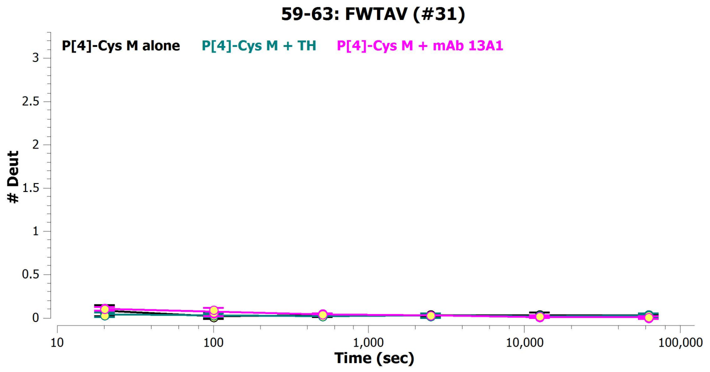
59-63: FWTAV (#31)
P[4]-Cys M alone
P[4]-Cys M + TH
P[4]-Cys M + mAb 13A1
# Deut
Time (sec)

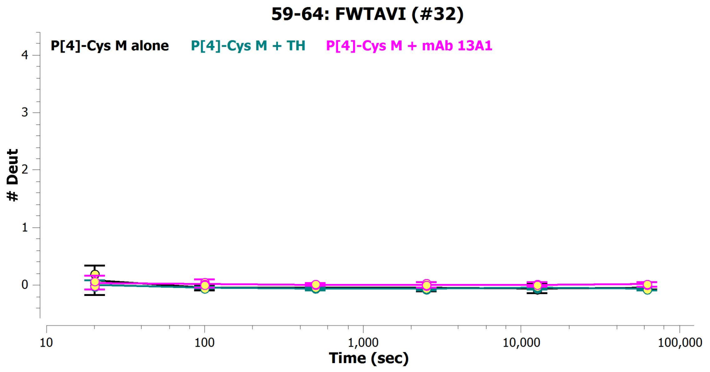
59-64: FWTAVI (#32)
P[4]-Cys M alone
P[4]-Cys M + TH
P[4]-Cys M + mAb 13A1
# Deut
Time (sec)

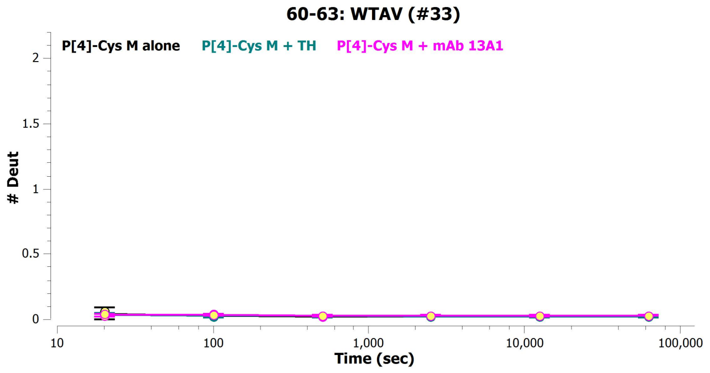
60-63: WTAV (#33)
P[4]-Cys M alone
P[4]-Cys M + TH
P[4]-Cys M + mAb 13A1
# Deut
Time (sec)

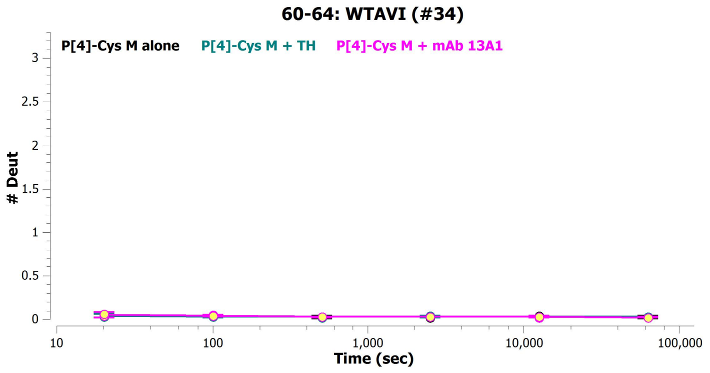
60-64: WTAVI (#34)
P[4]-Cys M alone
P[4]-Cys M + TH
P[4]-Cys M + mAb 13A1
# Deut
Time (sec)

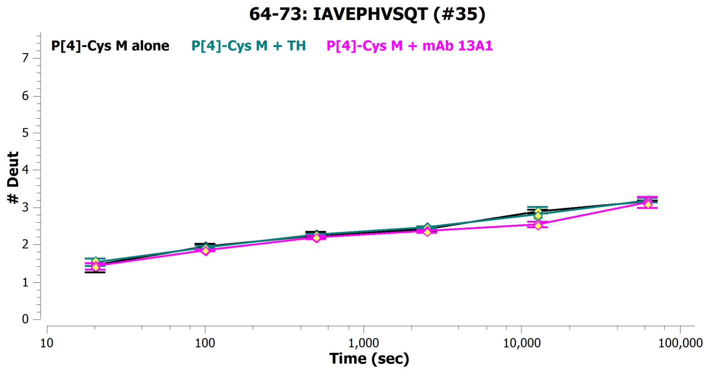
64-73: IAVEPHVSQT (#35)
P[4]-Cys M alone
P[4]-Cys M + TH
P[4]-Cys M + mAb 13A1
# Deut
Time (sec)

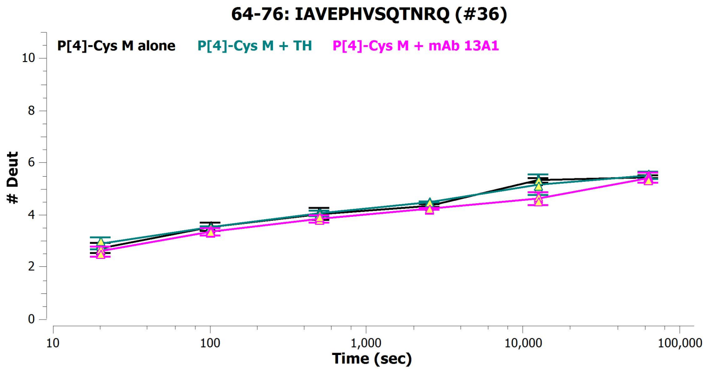
64-76: IAVEPHVSQTNRQ (#36)
P[4]-Cys M alone
P[4]-Cys M + TH
P[4]-Cys M + mAb 13A1
# Deut
Time (sec)

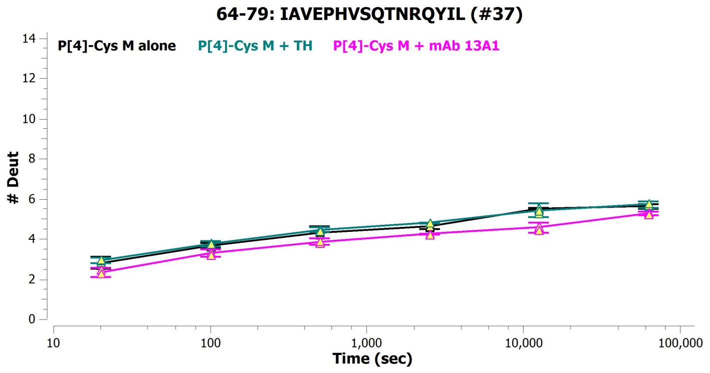
64-79: IAVEPHVSQTNRQYIL (#37)
P[4]-Cys M alone
P[4]-Cys M + TH
P[4]-Cys M + mAb 13A1
# Deut
Time (sec)

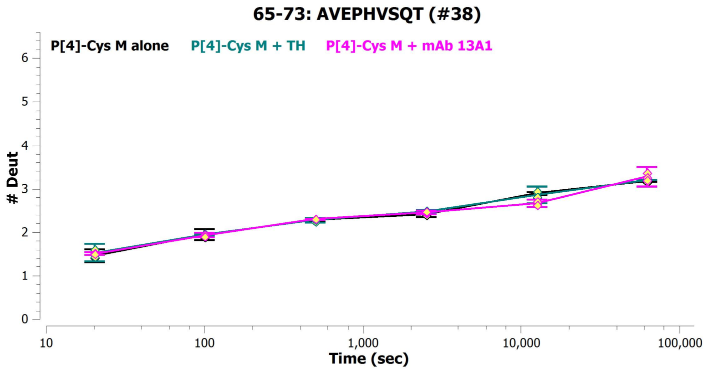
65-73: AVEPHVSQT (#38)
P[4]-Cys M alone
P[4]-Cys M + TH
P[4]-Cys M + mAb 13A1
# Deut
Time (sec)

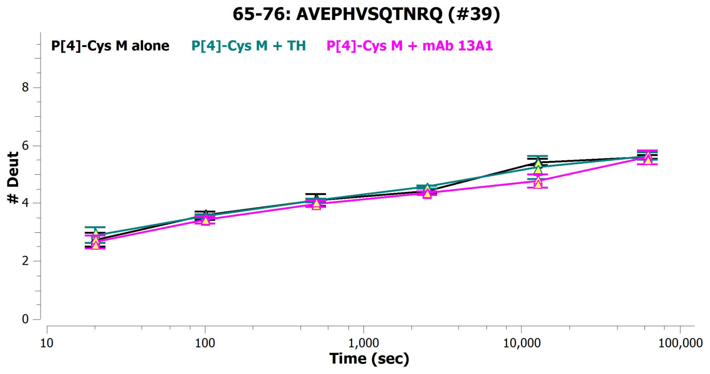
65-76: AVEPHVSQTNRQ (#39)
P[4]-Cys M alone
P[4]-Cys M + TH
P[4]-Cys M + mAb 13A1
# Deut
Time (sec)

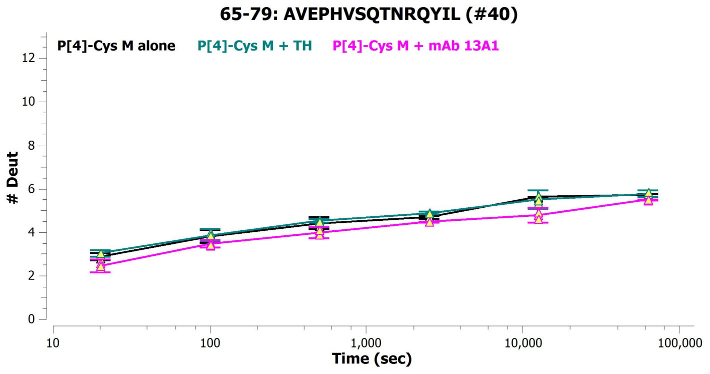
65-79: AVEPHVSQTNRQYIL (#40)
P[4]-Cys M alone
P[4]-Cys M + TH
P[4]-Cys M + mAb 13A1
# Deut
Time (sec)

66-76: VEPHVSQTNRQ (#41)

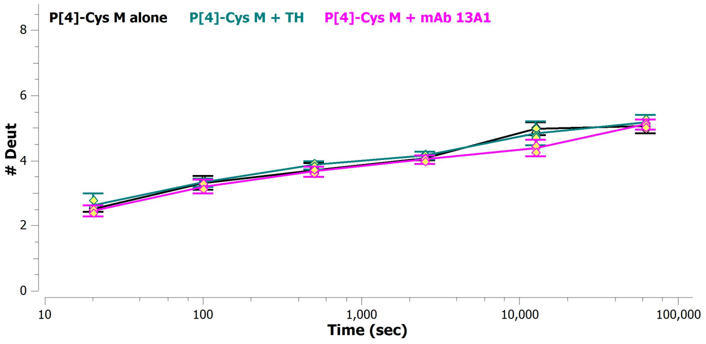


74-79: NRQYIL (#42)

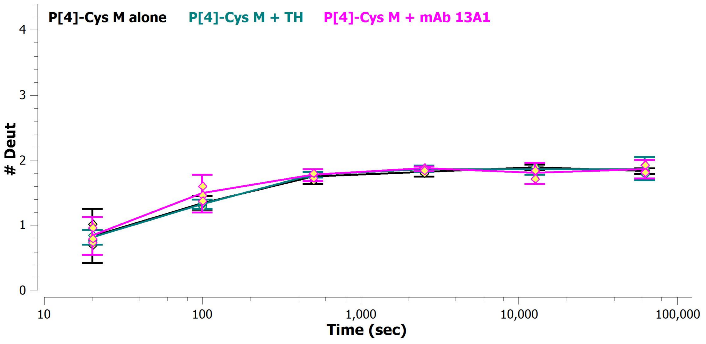


80-86: FGENKQF (#43)

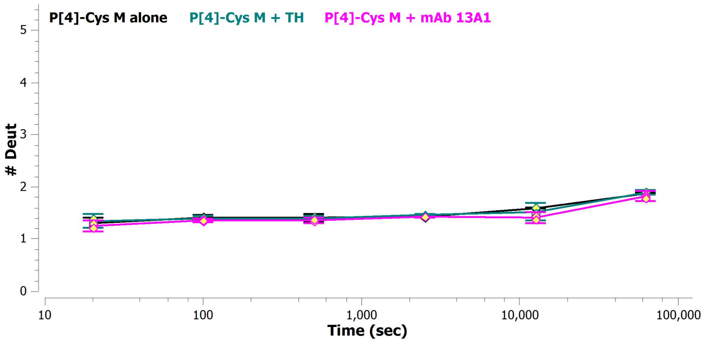


80-97: FGENKQFNVENNSDKWKF (#44)

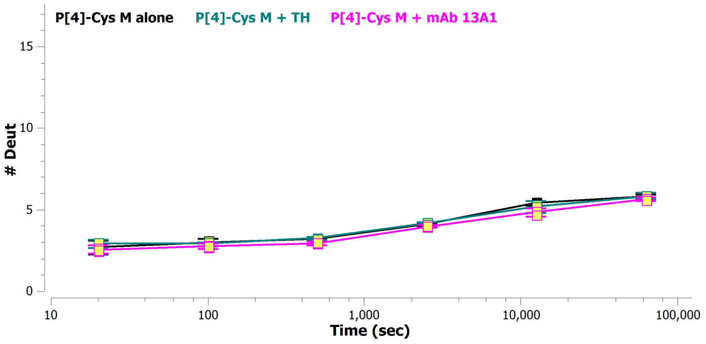


81-97: GENKQFNVENNSDKWKF (#45)

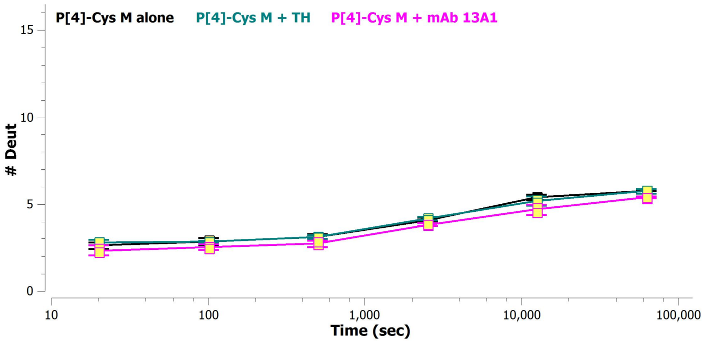


84-97: KQFNVENNSDKWKF (#46)

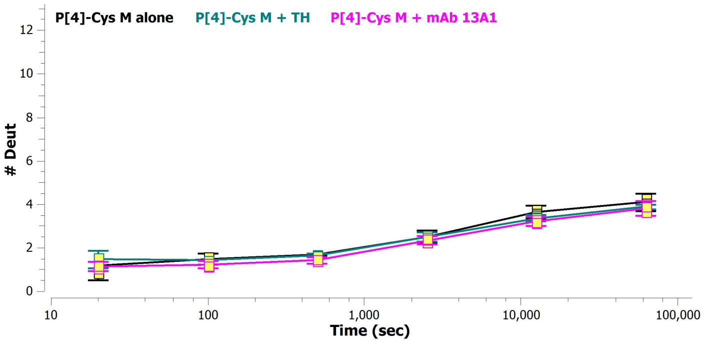


87-97: NVENNSDKWKF (#47)

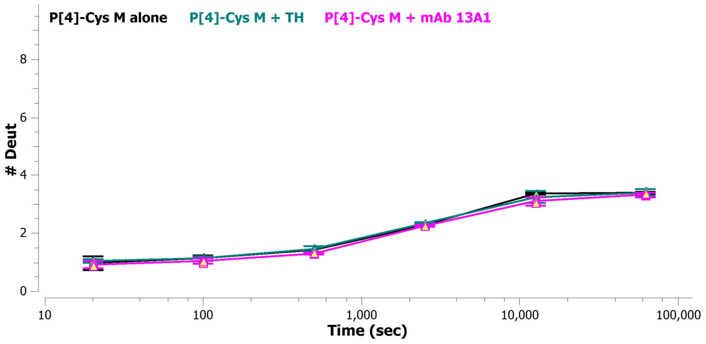


98-100: FEM (#48)

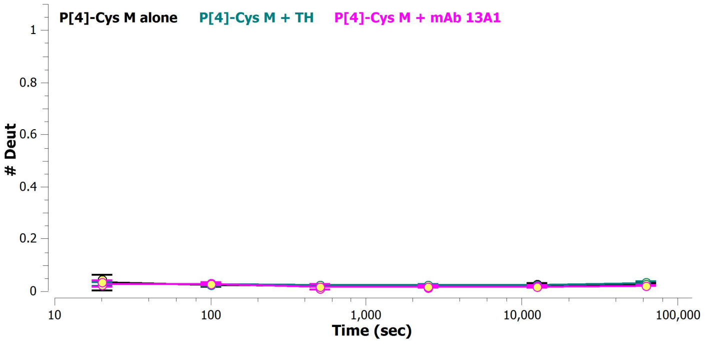


98-101: FEMF (#49)

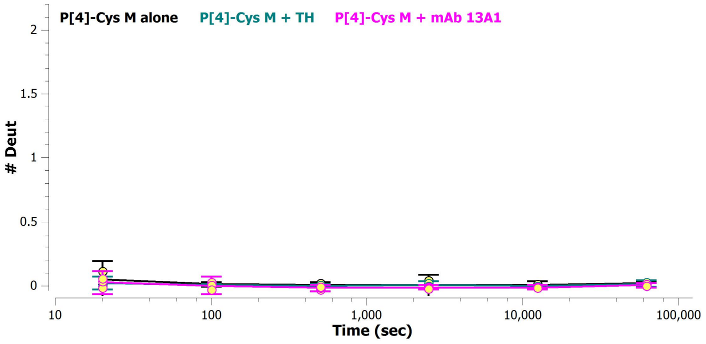


98-121: FEMFKGSSQGDFSNRRTLTSSNRL (#50)

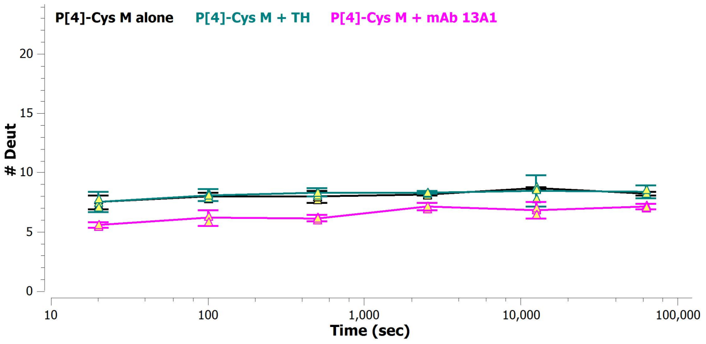

### 98-123: FEMFKGSSQGDFSNRRTLTSSNRLVG (#51)

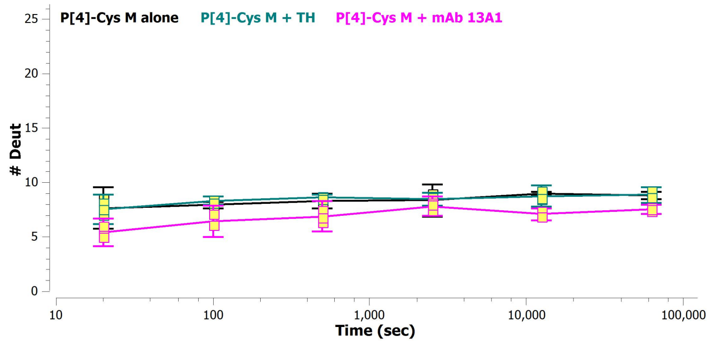


### 101-109: FKGSSQGDF (#52)

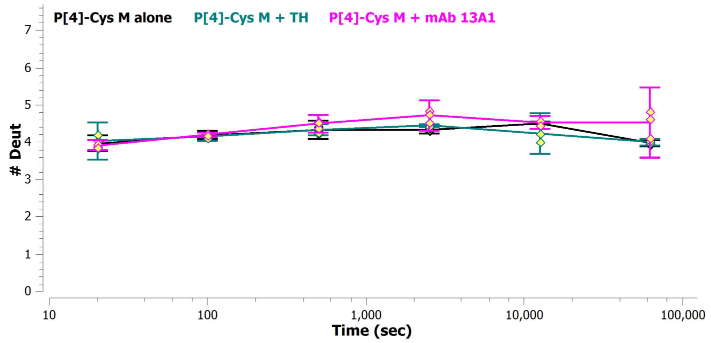


### 101-121: FKGSSQGDFSNRRTLTSSNRL (#53)

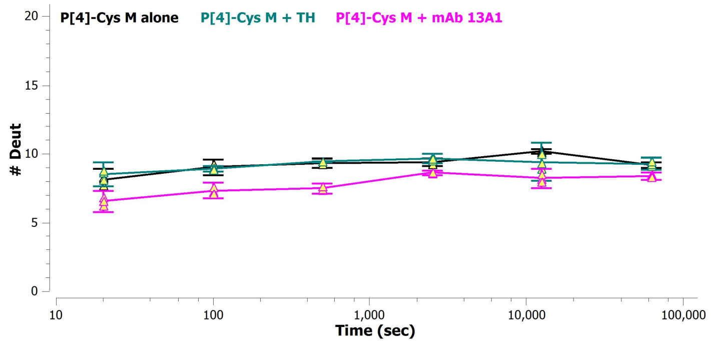


### 101-123: FKGSSQGDFSNRRTLTSSNRLVG (#54)

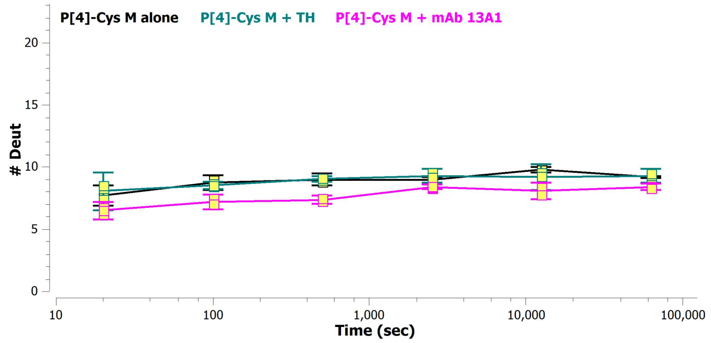


### 101-125: FKGSSQGDFSNRRTLTSSNRLVGML (#55)

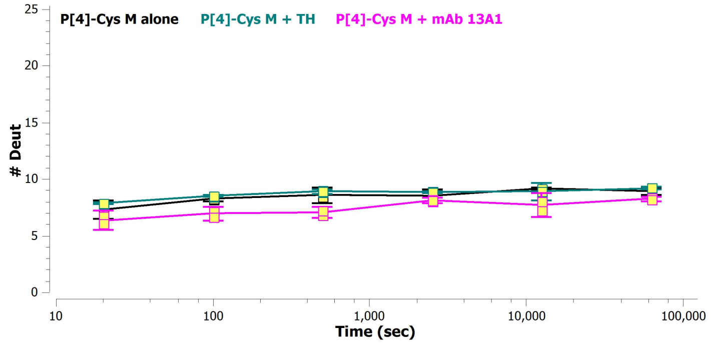


### 102-121: KGSSQGDFSNRRTLTSSNRL (#56)

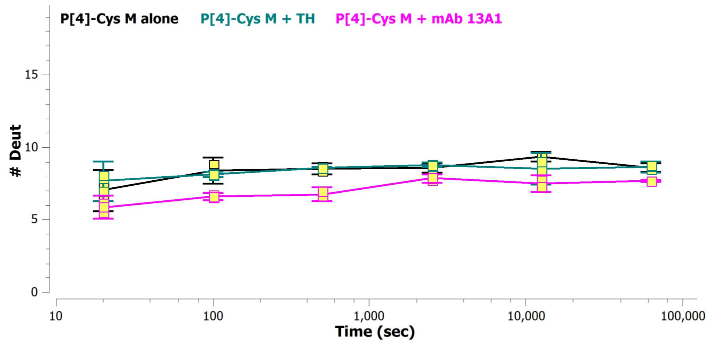


### 122-124: VGM (#57)

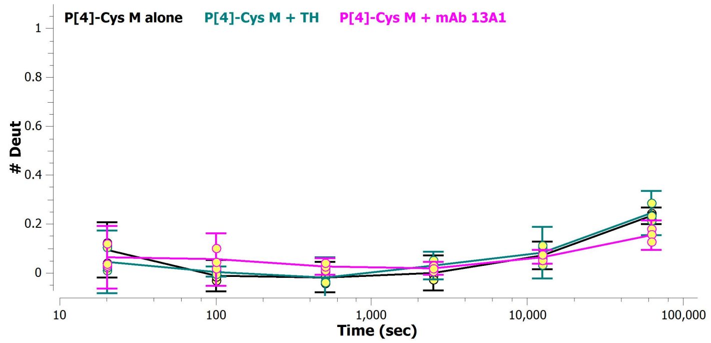


### 122-125: VGML (#58)

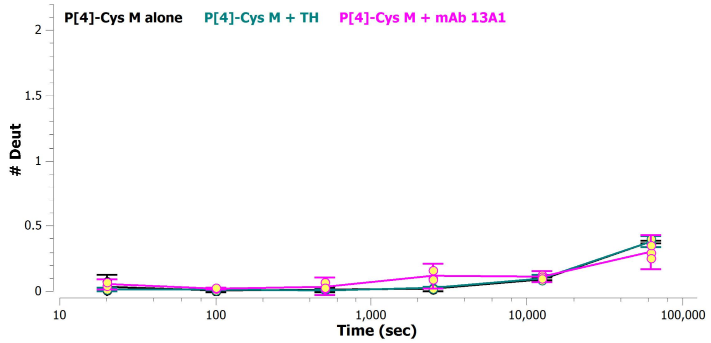


### 122-134: VGMLKYGGRVWTF (#59)

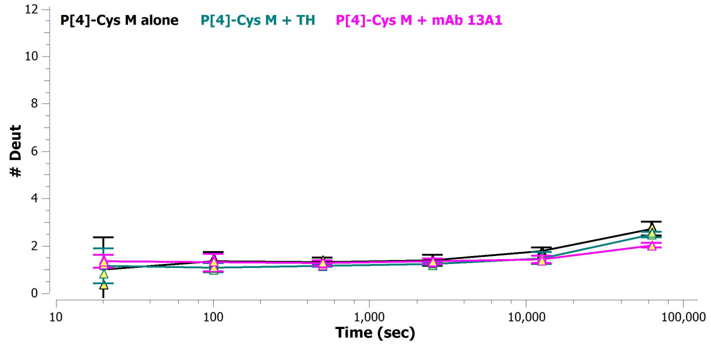


### 122-147: VGMLKYGGRVWTFHGETPRATTDSSN (#60)

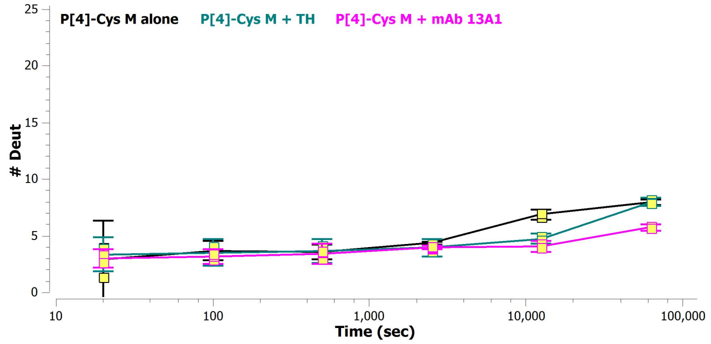

**122-153: VGMLKYGGRVWTFHGETPRATTDSSNTADLNN (#61)**

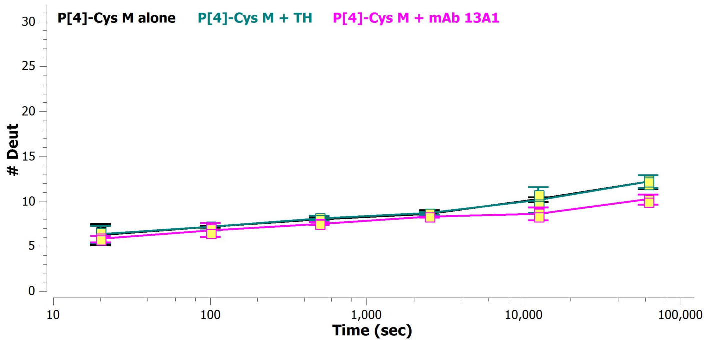


**122-155: VGMLKYGGRVWTFHGETPRATTDSSNTADLNNIS (#62)**

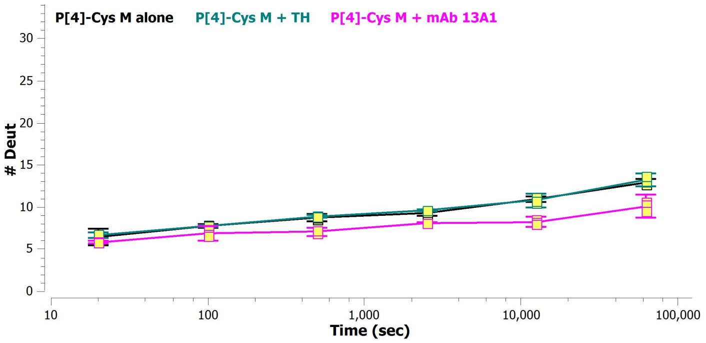


**124-134: MLKYGGRVWTF (#63)**

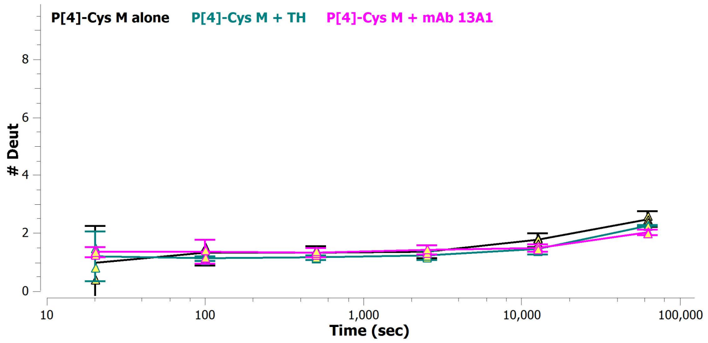


**124-151: MLKYGGRVWTFHGETPRATTDSSNTADL (#64)**

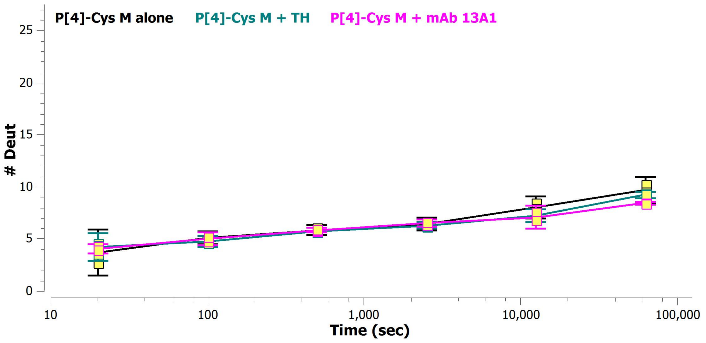


**124-155: MLKYGGRVWTFHGETPRATTDSSNTADLNNIS (#65)**

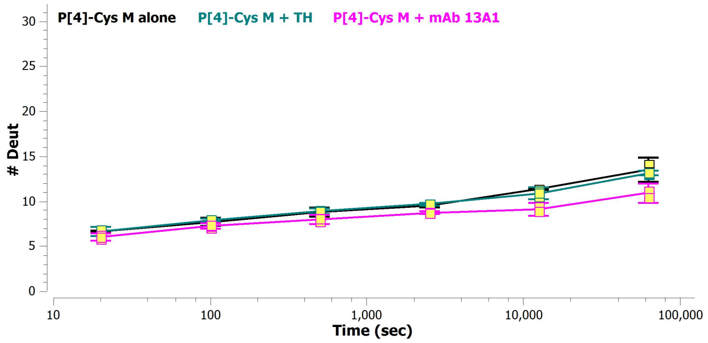


**125-134: LKYGGRVWTF (#66)**

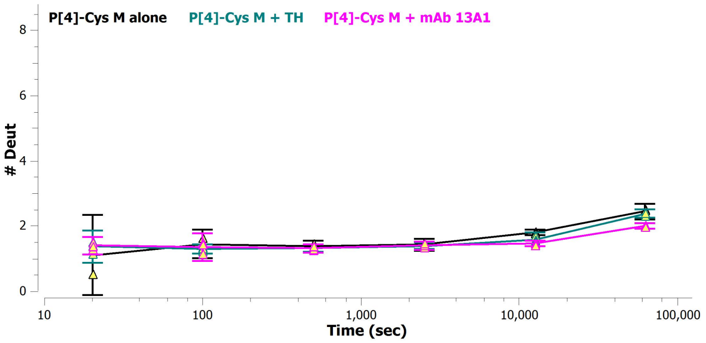


**125-151: LKYGGRVWTFHGETPRATTDSSNTADL (#67)**

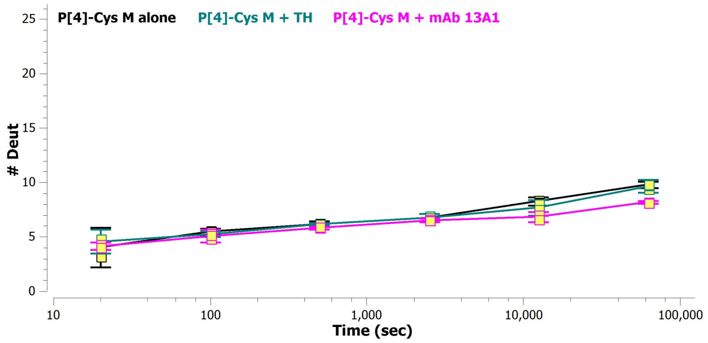


**125-153: LKYGGRVWTFHGETPRATTDSSNTADLNN (#68)**

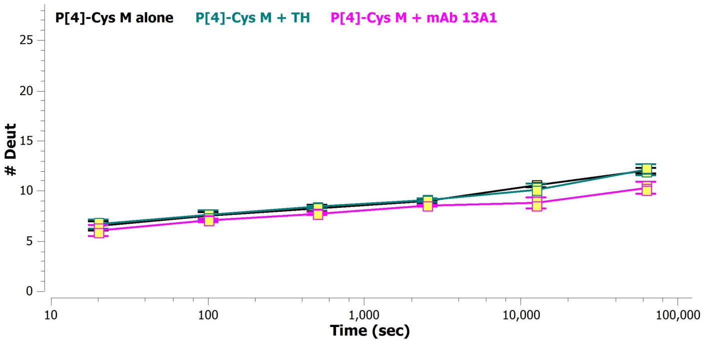


**125-155: LKYGGRVWTFHGETPRATTDSSNTADLNNIS (#69)**

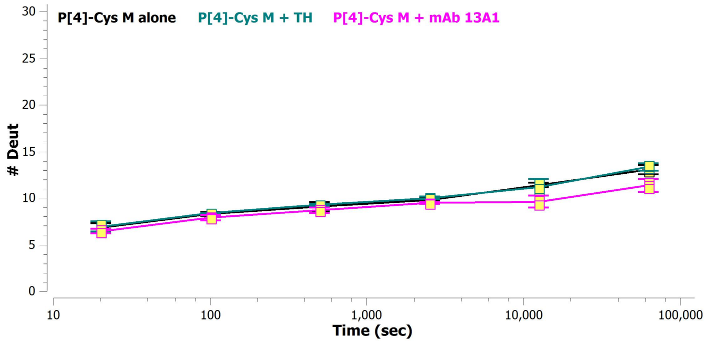


**126-155: KYGGRVWTFHGETPRATTDSSNTADLNNIS (#70)**

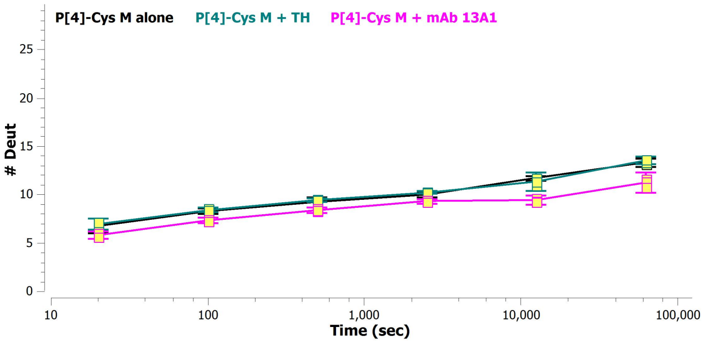

### 135-151: HGETPRATTDSSNTADL (#71)

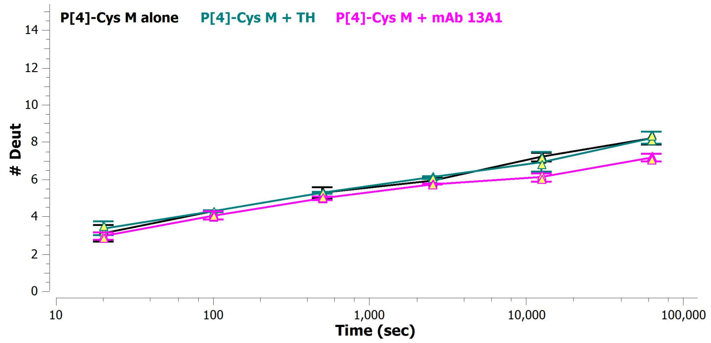


### 135-155: HGETPRATTDSSNTADLNNIS (#72)

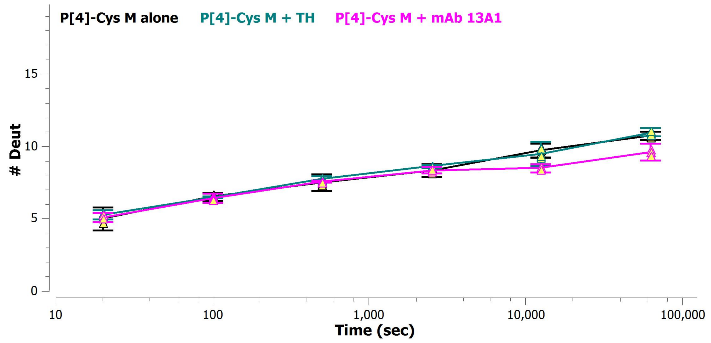


### 154-162: ISIIIHSEF (#73)

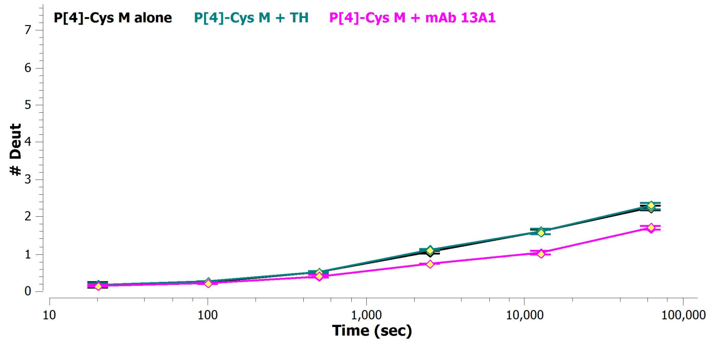


### 156-161: IIIHSE (#74)

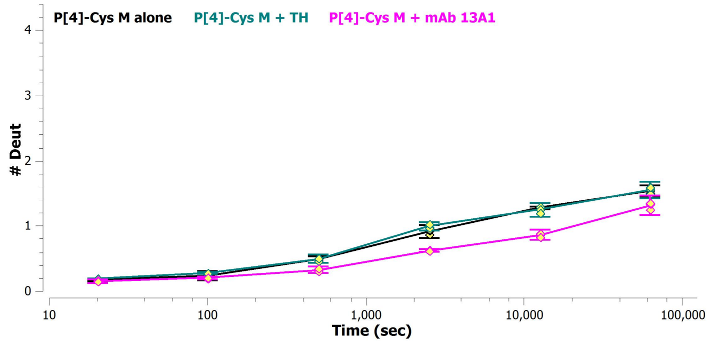


### 156-162: IIIHSEF (#75)

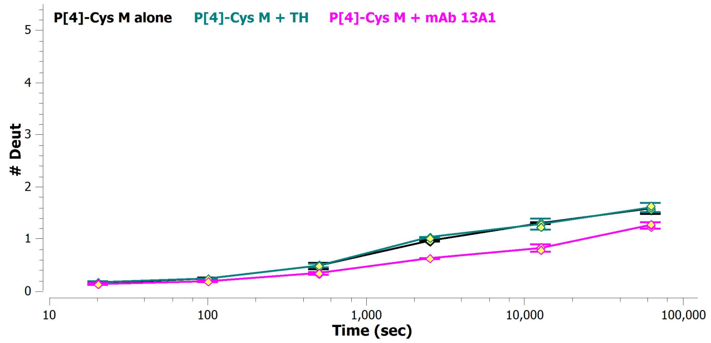


### 157-162: IIHSEF (#76)

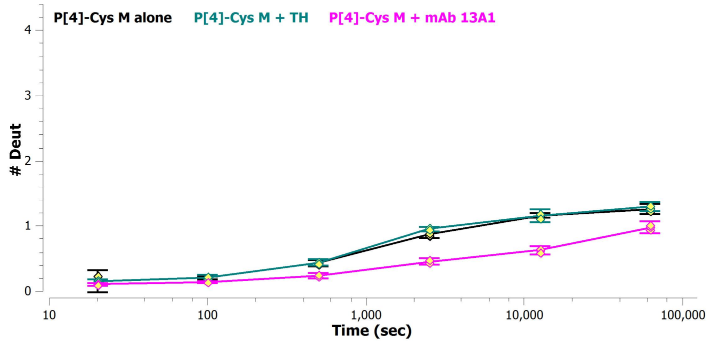


### 162-175: FYIIPRSQESKSNE (#77)

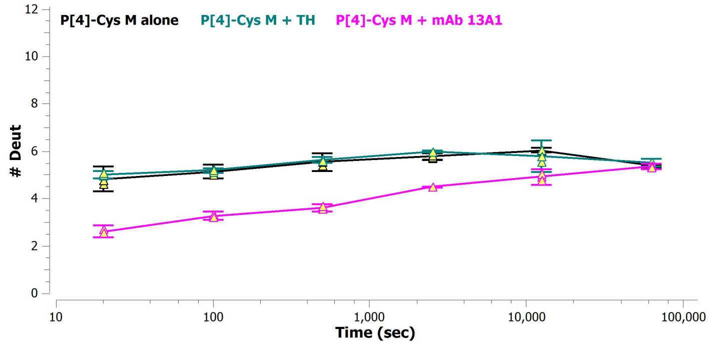


### 163-170: YIIPRSQE (#78)

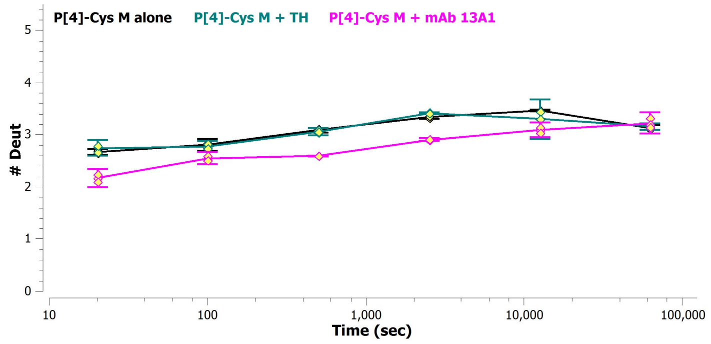


### 163-175: YIIPRSQESKSNE (#79)

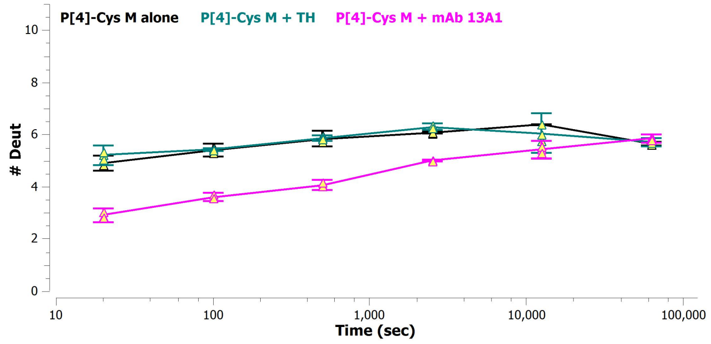


### 163-181: YIIPRSQESKSNEYINNGL (#80)

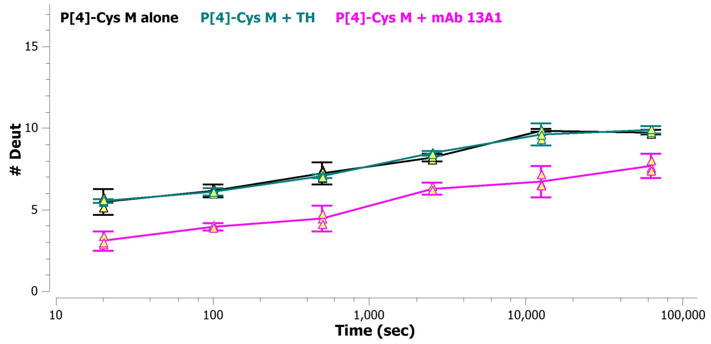

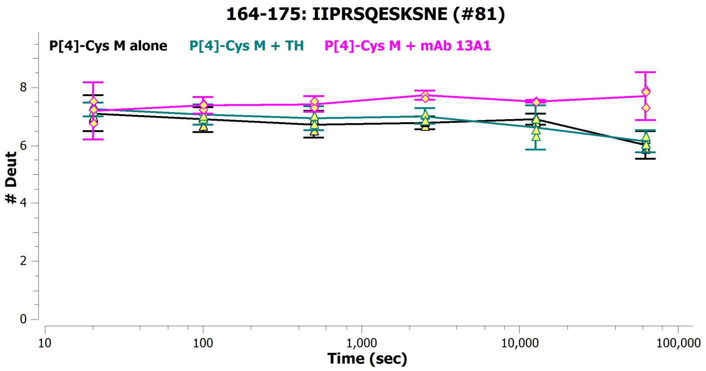
164-175: IIPRSQESKSNE (#81)
P[4]-Cys M alone
P[4]-Cys M + TH
P[4]-Cys M + mAb 13A1
# Deut
0
2
4
6
8
10
100
1,000
10,000
100,000
Time (sec)

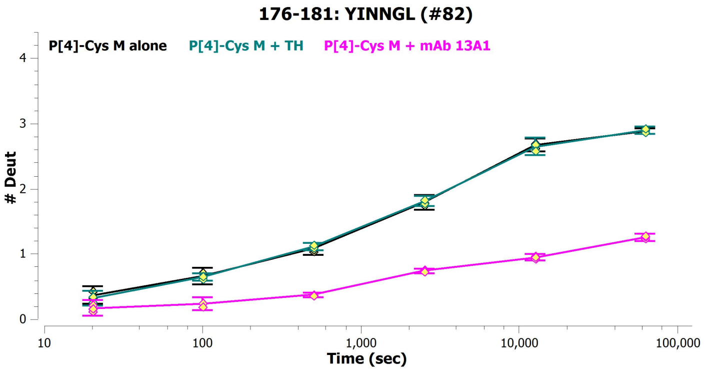
176-181: YINNGL (#82)
P[4]-Cys M alone
P[4]-Cys M + TH
P[4]-Cys M + mAb 13A1
# Deut
0
1
2
3
4
10
100
1,000
10,000
100,000
Time (sec)
